# Direct Synthesis of α‐Aryl‐α‐Trifluoromethyl Alcohols via Nickel Catalyzed Cross‐Electrophile Coupling

**DOI:** 10.1002/anie.202211732

**Published:** 2022-10-18

**Authors:** Lorenzo Lombardi, Alessandro Cerveri, Riccardo Giovanelli, Marta Castiñeira Reis, Carlos Silva López, Giulio Bertuzzi, Marco Bandini

**Affiliations:** ^1^ Dipartimento di Chimica “Giacomo Ciamician” Alma Mater Studiorum— Università di Bologna Via Selmi 2 40126 Bologna Italy; ^2^ Center for Chemical Catalysis—C^3^ Alma Mater Studiorum— Università di Bologna Via Selmi 2 40126 Bologna Italy; ^3^ Universidade de Vigo AS Lagoas (Marcosende) s/n 36310 Vigo Spain

**Keywords:** Cross-Electrophile Coupling, DFT Calculation, Hydrogen Atom Transfer, Nickel Catalysis, Trifluoromethylation

## Abstract

A nickel‐catalyzed reductive cross‐electrophile coupling between the redox‐active *N‐*trifluoroethoxyphthalimide and iodoarenes is documented. The protocol reproduces a formal arylation of trifluoroacetaldehyde under mild conditions in high yields (up to 88 %) and with large functional group tolerance (30 examples). A combined computational and experimental investigation revealed a pivotal solvent assisted 1,2‐Hydrogen Atom Transfer (HAT) process to generate a nucleophilic α‐hydroxy‐α‐trifluoromethyl C‐centered radical for the Csp^2^−Csp^3^ bond forming process.

## Graphical abstract

A nickel catalyzed synthesis of α‐aryl‐α‐trifluoromethyl alcohols **B** is presented that exploits the condensation of iodoarenes and the redox active ether **A** via cross‐electrophile coupling. A mechanistic study was conducted using a comprehensive computational investigation that was also supported by *ad hoc* control experiments and showed that the key 1,2‐HAT (Hydrogen Atom Transfer) event results in the formation of a C‐centered radical **C′** that mimics the reactivity of trifluoroacetaldehyde.

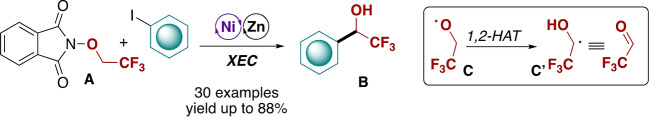

The incorporation of fluorine into organic compounds can dramatically tune/modify the overall chemical, physical and biological properties of molecular and polymeric architectures.^[1]^ Accordingly, the development of sustainable and selective synthetic methodologies for the introduction of fluorine‐based functional groups in carbon skeletons keeps receiving growing attention by the entire chemical community.^[2]^

In this scenario, α‐aryl‐α‐trifluoromethyl alcohols are of utmost importance, constituting a volume of biologically and pharmacologically active compounds (Figure 1a).^[3]^ The development of sustainable synthetic routes to these scaffolds has fascinated organic chemists during the past decades resulting predominantly in two disconnecting approaches. In particular, the direct nucleophilic trifluoromethylation of carbonyl compounds (mainly aldehydes) has been extensively investigated by means of the Ruppert‐Prakash reagent (TMSCF_3_) and other analogous systems (i.e. ICF_3_/TDAE (tetrakis(dimethylamino)ethylene), HCF_3_/base, B−CF_3_ adducts, CF_3_CHO hydrate, hemiaminals and S‐based transfer reagents, Figure 1b, path i).^[4]^ However, the relatively high cost and limited choice of “F_3_C^−^” synthons still represent major shortcomings, especially towards large scale applications. In addition, several of these protocols employ strongly basic conditions, incompatible with acidic substrates and may require cryogenic temperatures due to the instability of trifluoromethide anion. A complementary electrophilic approach, involving the addition of organometallic reagents to trifluoroacetates, has also been explored (Figure 1b, pathway ii).^[5]^ However, the limited scope, stringent substrate‐depending conditions and the requirement of highly reactive organometallics in stoichiometric amount do affect the generality and feasibility of the latter method. Finally, direct Friedel–Crafts‐like protocols involving CF_3_CHO derivatives as alkylating agents are limited to electron‐rich arenes.[^5d^, ^5e^, ^5f^, ^5g^]

**Figure 1:**
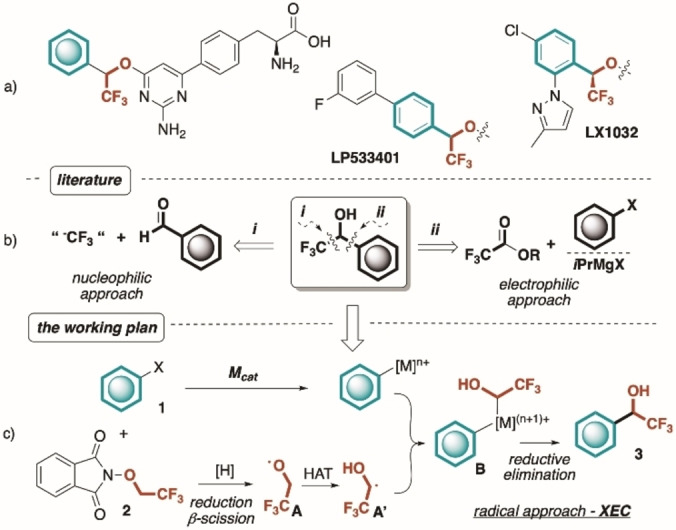
a) Representative examples of bioactive α‐aryl‐α‐trifluoromethyl alcohols. b) Known synthetic nucleophilic and electrophilic approaches to α‐aryl‐α‐trifluoromethyl alcohols. c) Present working idea.

Aiming to expand the synthetic routes towards α‐aryl‐α‐trifluoromethyl carbinols **3**, we envisioned that the emerging area of metal catalyzed cross‐electrophilic coupling (XEC)^[6]^ could be a valuable alternative to the common pitfalls of stochiometric variants.

To this end, we hypothesized that the readily available and poorly explored redox‐active ether *N*‐trifluoroethoxyphthalimide **2**,^[7]^ could formally turn inexpensive CF_3_CH_2_OH (TFE), into a potential chemical analog of CF_3_CHO. In particular, under reductive conditions, **2** is known to deliver the highly electrophilic oxygen centered radical **A**, capable of performing HAT processes on unactivated C(sp^3^)−H bonds.[^8^, ^9^] However, in the absence of suitable H donors, we speculated that the conversion of **A** into the corresponding carbon centered analogue **A′** (via intramolecular 1,2‐HAT) could occur in synthetically useful amounts.[^10^, ^11^] Eventually, the trapping of **A′** by a metal‐activated aryl moiety (Ar‐[M]) and subsequent reductive elimination (RE) of the resulting organometallic species **B**, would result into the desired fluorinated motif **3** (Figure 1c).

In conjunction with our ongoing research interest on nickel catalyzed coupling reactions^[12]^ and due to its great efficiency in XEC, we decided to employ Ni^II^ pre‐catalysts for accessing this C(sp^2^)−C(sp^3^) bond‐forming protocol under reductive conditions.

It is worth noting that the MacMillan group has shown the utilization of α‐hydroxy C‐centered radicals in a Ni‐catalyzed α‐arylation of alcohols under photochemical regime.^[6j]^ However, TFE was not engaged in the transformation, perhaps due to the slow kinetics of HAT processes with electrophilic abstractors on fluorinated alcohols/alkoxides.[^9b^, ^9c^] Therefore, the present methodology would represent the first catalytic strategy to access **3** from aryl halides.

At the outset of our investigation, we considered the condensation of *p*‐iodotoluene **1 a** with **2** in the presence of a range of [Ni^II^] complexes. A collection of salient results is summarized in Table 1 (see Supporting Information for further and exhaustive list of attempts). Interestingly, in the presence of 10 mol% of [Ni(**L1**)Cl_2_] (**L1**=phen), TMSCl as an additive, Zn as the reductant and DMA as the reaction medium ([**1 a**]=0.2 M) the desired trifluoromethyl benzyl alcohol **3 a** was obtained in 36 % yield (entry 1), demonstrating the feasibility of our approach. Under similar conditions, other metal (i.e. Mn) and organic (i.e. TDAE) reductants were tested, however, no improvements with respect to Zn were recorded (entries 2,3). The addition of TMSCl guaranteed better reproducibility and faster triggering of the [Ni^II^] reduction by Zn. As expected, both Ni catalyst and reductant (Zn) proved mandatory for the formation of product (see Supporting Information).

**Table 1:** Optimization of the reaction conditions.

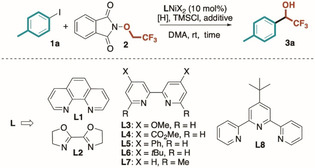				
Run^[a]^	**L**	[H]/additive	Time [h]	Yield **3 a** [%]^[b]^
1	[Ni(**L1**)Cl_2_]	Zn/–	16	36
2	[Ni(**L1**)Cl_2_]	Mn/–	16	16
3	[Ni(**L1**)Cl_2_]	TDAE/–	16	0
4^[c,d]^	[Ni(**L2**)Cl_2_]	Zn/–	16	0
5^[c]^	[Ni(**L3**)Cl_2_]	Zn/–	16	55
6	[Ni(**L3**)Br_2_]	Zn/–	18	54
7^[c,d]^	[Ni(**L4**)Cl_2_]	Zn/–	16	8
8	[Ni(**L5**)Cl_2_]	Zn/–	16	59
9^[c]^	[Ni(**L6**)Cl_2_]	Zn/–	16	48
10^[c,d]^	[Ni(**L7**)Cl_2_]	Zn/–	16	0
11^[c]^	[Ni(**L8**)Cl_2_]	Zn/–	16	5
12	[Ni(**L5**)Cl_2_]	Zn/NaI	2	95 (88)
13	[Ni(**L5**)Cl_2_]	Zn/–	2	30
14^[e]^	[Ni(**L5**)Cl_2_]	Zn/NaI	2	60

[a] All reactions were carried out under N_2_ in dry DMA ([**1 a**]: 0.2 M, **2**: 3 eq., [H]: 2 eq., TMSCl: 1 eq.; additive: 1 eq., cat: 10 mol%, unless otherwise specified). [b] Determined by ^19^F NMR on the reaction crude with an internal standard (CF_3_C_6_H_5_). In brackets, isolated yields after flash chromatography. [c] The catalytic complex was prepared in situ (**L**/NiCl_2_ ⋅ glyme: 15/10 mol%). [d] Dehalogenative homocoupling of **1 a** was determined as the major product. [e] With reagent grade DMA and under air. [H]: reductant. NR: no reaction. TMSCl: chlorotrimethylsilane.

Subsequently, a range of chelating 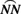 and 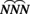 ligands **L2**–**8** was tested in the model transformation. While bi‐oxazoline **L2** and tridentate pyridyl ligand **L8** proved ineffective in promoting the reductive coupling (entries 4, 11), bipyridyl ligands displayed variable performance, strongly influenced by electronic as well as steric properties. In particular, the introduction of methyl groups at the 6,6′‐positions resulted in the exclusive dehalogenative homocoupling product of **1 a**. On the contrary, the introduction of electron‐releasing substituents at the 4,4′‐positions (i.e. Ph, OMe and *t*Bu) led to an overall increase of chemical yields regardless the type of counterion on the nickel complex (48–59 %, entries 5,6,8,9). Based on these findings, we selected **L5** as the optimal ligand for this reductive coupling.

An extensive list of additives was surveyed to further increase the chemical performance of the catalytic process (see Supporting Information). Interestingly, the addition of NaI (1 eq.) sped up the reaction enabling **3 a** to be obtained in almost quantitative yield in only 2 h when preformed [Ni(**L5**)Cl_2_] complex^[13]^ was utilized as the catalyst (yield=88 %, entry 12). Interestingly, the protocol resulted also robust to open flask conditions and reagent grade DMA, providing **3 a** in synthetically useful 60 % yield (2 h, entry 14).

The beneficial role of halide salts is well documented in reductive Ni‐based cross‐coupling reactions, and although the origin of this effect is still under debate, several possibilities have been proposed.^[14]^ Halide ions could accelerate the reduction of Ni^II^ species by acting as a bridging ligand with the metal reductant,[^15a^, ^15b^] or by removing Zn^II^ salts from the metal surface.[^15d^, ^15e^] Promotion of ligand exchange processes with coordinating solvents,^[15c]^ and the formation of metal species with enhanced catalytic activity[^14e^, ^15b^, ^15f^] have been suggested as well.

With the optimized reaction conditions in hand ([Ni(**L5**)Cl_2_] 10 mol%, **2** 3 eq., Zn/NaI/TMSCl, DMA, rt), we assessed the generality of the protocol by condensing a range of iodoarenes to **2**. The data reported in Scheme 1 unequivocally account for the robustness of the catalytic protocol and the wide functional group tolerance. Remarkably, electron‐donating groups (*i.e*. OMe, OBn, OAllyl, OAc, Me, NBn_2_ NHCOCF_3_ and NPhth, **1 b**–**j**) could be adequately accommodated both at the *meta* and *para* positions with respect to the iodoarene substitution. In all cases, good yields were achieved (up to 74 %). The tolerance towards trifluoroacetamide moiety (**1 h**) underlined also the suitability of protic functional groups in the present methodology. Analogously, a wide range of electron‐withdrawing groups (i.e. halogens, CF_3_, ester, ketone, **3 k**–**s**) were effectively handled in *meta* and *para* positions. Interestingly, 4‐bromoiodobenzene **1 m** was tolerated (60 % yield for **3 m**) under reductive conditions, showing a notable I/Br selectivity (9 : 1) of our cross‐electrophile condensation. Simple iodobenzene **1 t** and benzofused 1‐iodonaphthalene **1 u** performed analogously well, providing the corresponding α‐aryl‐α‐trifluoromethyl alcohols **3 t**,**u** in 70 % and 61 % yield, respectively.

**Scheme 1:**
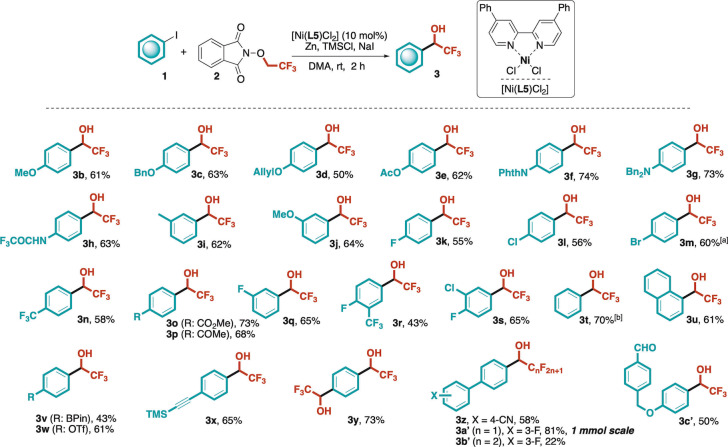
Generality of the present Ni‐catalyzed reductive cross‐electrophile‐coupling. Isolated yields after flash chromatography are provided, unless otherwise specified. ^[a]^ Isolated as a 9 : 1 mixture of Br/I containing alcohols. ^[b]^ ^19^F NMR yield based on internal standard (CF_3_C_6_H_5_). Product **3 t** resulted too volatile to be isolated. Bpin: boron pinacolate, Nphth: phthalimide, OTf: trifluoromethanesulfonate; TMS: trimethylsilyl.

Then, to properly assess the chemoselectivity and site‐specificity of our procedure, we subjected to optimal conditions iodobenzenes carrying cross‐coupling‐active functional groups such as: Bpin (**1 v**) and OTf (**1 w**). Remarkably, these substituents were nicely tolerated and the corresponding trifluoromethyl benzyl alcohols (**3 v**,**w**) were isolated in satisfactory yields (43–61 %). Moreover, the TMS‐protected alkynyl unit (**1 x**) did not perturbate the reaction course delivering the secondary alcohol **3 x** in 65 % yield. Finally, the possibility to carry out two consecutive formal reductive arylations with **2** was verified with 1,4‐diiodobenzene **1 y**. The corresponding dialkylated compound **3 y** was isolated in synthetically useful 73 % yield. We then focused our attention on 1,4‐biaryl scaffolds due to their predominance in biologically active α‐aryl‐α‐trifluoromethyl alcohols. In particular, the recorded efficiency on 1,4‐biaryl scaffold **1 z** (58 % yield) led us to extend our methodology to 3‐fluoro‐4′‐iodo‐1,1′‐biphenyl **1 a′**, whose product **3 a′** constitutes one of the key building blocks for the preparation of pharmacologically active **LP533401**. Interestingly, when optimal conditions were applied to readily available **1 a′** (*1 mmol scale*), the resulting benzyl alcohol **3 a′** was isolated in 81 % yield. In order to assess the extendibility of the process to other perfluoroalkyl chains, *N*‐pentafluoropropoxyphthalimide **2′** was prepared and reacted with substrate **1 a′**. Thus, **3 b′**, a homologue of the pharmaceutical building block **3 a′**, was obtained in a modest 22 % yield (unoptimized).

Finally, the methodology was tested on a formyl‐containing substrate that would result unsuitable in a classic trifluoromethylation protocol under Ruppert‐Prakash conditions. Here, the complementarity of our methodology *vs* TMSCF_3_‐based approaches was verified by subjecting iodoarene **1 c′** to optimal conditions effectively delivering **3 c′** in 50 % isolated yield.

In parallel, and encouraged by these results, we decided to carry out molecular modelling studies to gain a deeper understanding of the reaction mechanism operating in this catalytic system (Scheme 2).^[16]^ For this purpose, we adopted [Ni(**L3**)Br_2_] (**I**) as the catalyst and compounds **1 p** and **2** as the model substrates.^[17]^ We have found that, in the presence of Zn, the Ni^II^ pre‐catalyst is reduced to Ni^I^,^[18]^ yielding the active catalyst (**III**), which is capable of complexing both iodoarene and **2**. While the coordination of the latter is endergonic by 5.9 kcal mol^−1^, the coordination of the former renders a complex that is slightly more stable than **III** (see Scheme 2, right and Figure S2). Hence, the formation of **IV** will predominate in solution. Subsequently, the system can further progress via the oxidative addition of iodoarene, rendering **V**, followed by a SET process between Zn and intermediate **V** to yield Ni^II^‐aryl intermediate **VI**. Then, trapping of nucleophilic C‐centered radical species **VIII** (see below) by **VI** would deliver the Ni^III^‐alkyl‐aryl adduct **XIII**, that undergoes a very facile reductive elimination (with a barrier of only 5.5 kcal mol^−1^) yielding the observed product and the simultaneous recovery of the active catalyst **III**.

**Scheme 2:**
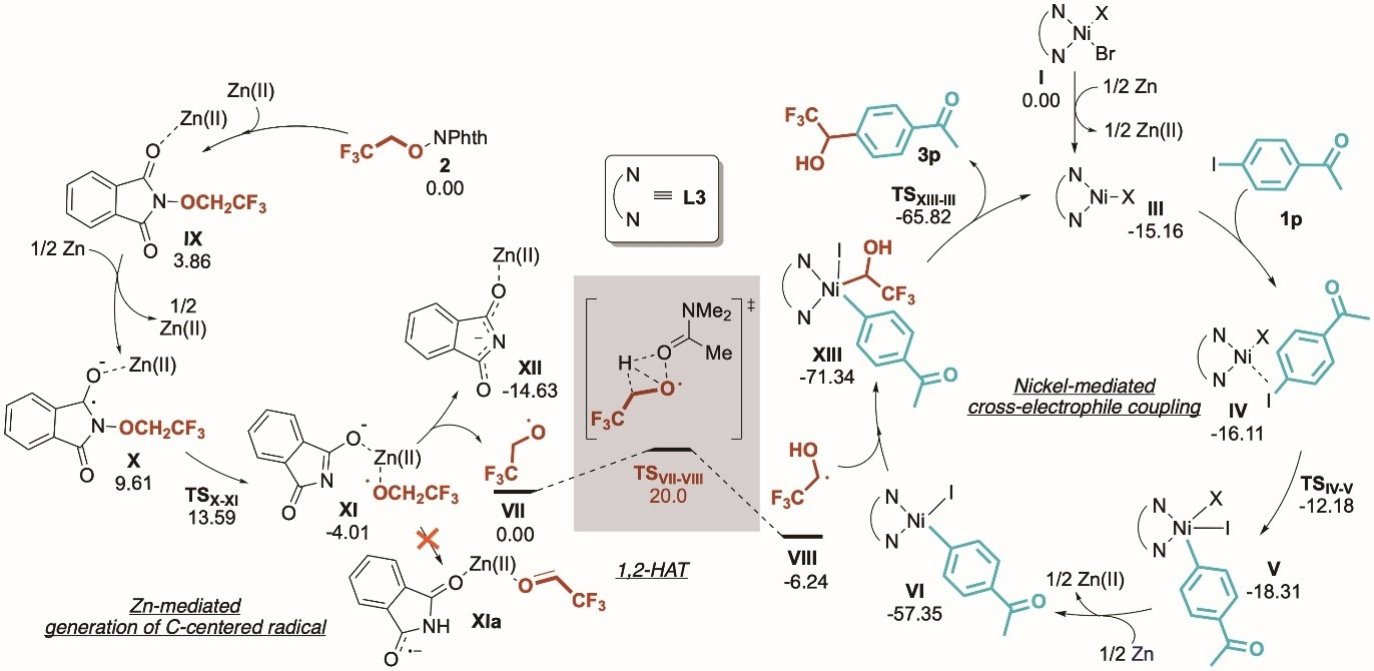
Proposed mechanistic paths for: cross‐electrophile coupling (right) and 1,2‐HAT process to deliver the postulated C‐centered radical **VIII** (NPhth: phthalimide, left). Computations have been performed at the PCM(*N,N*‐dimethylacetamide)^[19]^‐M06/def2svpp^[20]^ computational level. Counterions have been disregarded in this scheme for the sake of clarity but considered in the simulations (see Supporting Information for further details).

In addition, the generation of key radical **VIII** was investigated computationally (Scheme 2, left). Interestingly, the role of Zn goes beyond mere ligand exchange and reducing tasks, and we have found it to be also responsible for activating and promoting the N−O bond cleavage of the phthalimide core, resulting in the facile release of the alkoxy radical **VII**. At this stage, the oxygen‐centered radical **VII** can evolve via a solvent promoted 1,2‐HAT, furnishing the pivotal species **VIII**. Here, it could be argued that **XI** could also evolve via a hydrogen transfer, forming **XIa**. Computations suggest that this formal 1,2‐H migration on the alkoxide is energetically very demanding and therefore non‐competitive (see Figure S8).

To support the involvement of the radical intermediate **VIII** in the C−C bond forming event, the model reaction was carried out in the presence of radical trapping agents such as *tert‐*butyl acrylate **4 a** and TEMPO **4 b** (Scheme 3). As expected, when **4 a** was utilized as a Michael acceptor the trapping of **VIII** occurred at the β‐position (35 % isolated yield).^[21]^ Analogously, the addition of 1 eq. of TEMPO generated small amount of the silylated acetal **5 b** that was detected in the reaction crude (see Supporting Information for details).

**Scheme 3:**
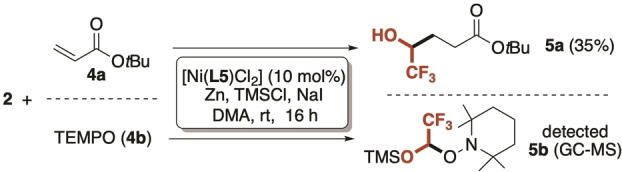
Control experiments addressing the key role of the carbon‐centered radical (**VIII**). TEMPO: 2,2,6,6‐tetramethylpiperidine‐1‐oxyl.

Finally, the synthetic significance of the methodology was tested on the late‐stage functionalization of derivatized biologically relevant scaffolds **6 a**–**c** (Scheme 4). Synthetically useful reductive cross‐electrophile coupling was obtained with *N*‐(4‐iodobenzoyl) phenyl alanine **6 a** that delivered the corresponding trifluoromethyl alcohol **7 a** in 65 % yield. Additionally, highly lipophilic scaffolds such as menthol‐ester **6 b** and functionalized pregnenolone **6 c** proved suitable as well, providing the desired alcohols **7 b** and **7 c** in 46 % and 32 % yield, respectively.

**Scheme 4:**
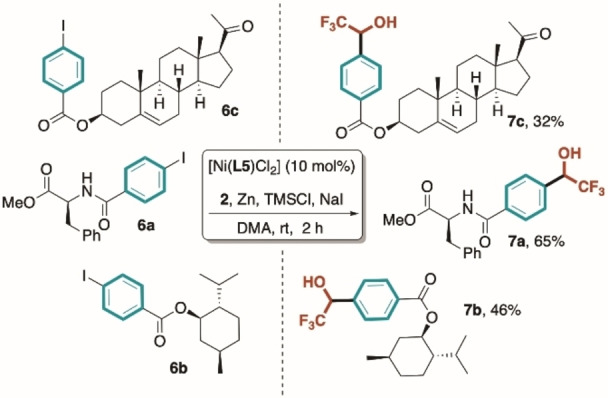
Late‐stage functionalization of biologically relevant iodoarene derivatives (**6 a**–**c**).

In conclusion, we are reporting a new radical approach for the synthesis of α‐aryl‐α‐trifluoromethyl alcohols, bypassing the use of toxic and gaseous trifluoroacetaldehyde via nickel catalyzed cross‐electrophilic coupling. The procedure exploits the *in situ* generation of a key C‐centered α‐hydroxy radical that undergoes efficient cross‐electrophile coupling mediated by Ni‐catalysis. In addition, the entire mechanistic profile was studied through a comprehensive computational investigation that was also supported by *ad hoc* control experiments. Finally, the protocol was found to be suitable for the preparation of biologically relevant building blocks and late‐stage functionalization of naturally occurring motifs.

## Conflict of interest

The authors declare no conflict of interest.

## Supporting information

As a service to our authors and readers, this journal provides supporting information supplied by the authors. Such materials are peer reviewed and may be re‐organized for online delivery, but are not copy‐edited or typeset. Technical support issues arising from supporting information (other than missing files) should be addressed to the authors.

Supporting Information

## Data Availability

The data that support the findings will be available in IRIS at https://cris.unibo.it/ following an embargo from the date of publication to allow for commercialization of research findings.
