# Supplementary material for: Direct Synthesis of α‐Aryl‐α‐Trifluoromethyl Alcohols via Nickel Catalyzed Cross‐Electrophile Coupling
Source: Angew Chem Int Ed Engl. 2022 Oct 18;61(47):e202211732. doi: 10.1002/anie.202211732 (PMC9828748; doi:10.1002/anie.202211732)
Supplement: Supplementary file 1 — Supporting Information [file ANIE-61-0-s001.pdf]

Angewandte Chemie
Eine Zeitschrift der Gesellschaft Deutscher Chemiker

Supporting Information

Direct Synthesis of α-Aryl-α-Trifluoromethyl Alcohols via Nickel Catalyzed Cross-Electrophile Coupling

L. Lombardi, A. Cerveri, R. Giovanelli, M. Castiñeira Reis, C. Silva López, G. Bertuzzi*, M. Bandini**

Table of Contents

1. General experimental methods 4

2. General computational details and considerations 5

3. Synthesis of starting materials 7

3.1 Synthesis of *N*-trifluoroethoxyphthalimide **2** 7

3.2 Synthesis of *N*-pentafluoropropoxyphthalimide **2’** 9

3.3 Synthesis or aryl iodides 10

3.4 Synthesis of Ligand **L5** and nickel complex [Ni(**L5**)Cl_2]. 11

4. Additional optimization data 12

4.1. **Table S2**: Initial screening on substrate **1p** 12

4.2. **Table S3**: Screening on substrate **1u** 14

4.3. **Table S4**: Additional screening data on substrate **1a** 16

4.4. **Table S5**: Additional screening data on substrate **1a** with **L5** 17

4.5 Explorative screening of chiral ligands 19

5. Ni catalyzed cross-coupling 20

5.1 General procedure for the Ni catalyzed cross-coupling reaction 20

5.2. Unsuccessful substrates 23

5.3 Characterization data of products **3** and **7** 24

6. Computational mechanistic studies 34

7. Radical trapping experiments 46

7.1 Trapping with tert-butylacrylate **4a** 46

7.1 Trapping with TEMPO radical **4b** 47

8. Control experiments 49

8.1 Experiment without Ni catalyst 49

8.2 Experiment without Zn 50

8.3 Experiment for Zn insertion with aryl iodide **1u** 51

8.4 Experiment with Trifluoroacetaldehyde ethyl hemiacetal 52

9. ^{1}H-, ^{19}F-, ^{13}C-NMR Spectra 53

10. Cartesians 119

11. References 175

1. General experimental methods

^{1}H NMR, ^{13}C NMR and ^{19}F NMR spectra were recorded on Varian 400-MR (400 MHz) (equipped with autoswitchable PFG probe) and Bruker Avance Neo 600 MHz (equipped with CryoProbe Prodigy Broadband 5mm) spectrometers. Data are reported as follows: chemical shift, multiplicity (s = singlet, d = doublet, dd= double doublet, t = triplet, td = triple doublet, dt = double triplet, q = quartet, sext = sextet, sept = septet, p = pseudo, b = broad, m = multiplet), coupling constants (Hz). Chemical shifts are reported in ppm from TMS with the solvent resonance as the internal standard.
HRMS spectra were obtained with a G2XS QTof mass spectrometer using either ESI or APCI ionization techniques, as specified case by case.
Melting points were determined with a Büchi Melting Point B-540 apparatus and are not corrected.
Chromatographic purification was done with 240-400 mesh silica gel.
Anhydrous solvents, except for DMA, were supplied by Sigma Aldrich in Sureseal® bottles and used without any further purification. Reagent grade DMA was purchased from Fluorochem and dried as follows: DMA was stirred overnight with CaO at r.t., then the mixture was refluxed for 1h, followed by distillation under reduced pressure (ca. 50 mbar, 50°C). The first 5% head distillate was discarded, the rest collected in a receiving flask with activated 4Å MS and then degassed by sparging N_2 for 15 minutes.
Room temperature (r.t.) refers to the ambient temperature of the laboratory, ranging from 22 °C to 26 °C.
Commercially available chemicals and (non-anhydrous) solvents were purchased from Sigma Aldrich, Fluorochem and TCI Chemicals and used without any further purification.
Zn dust refers to a particle size <10 µm and was purchased from Sigma Aldrich, having ≥98% purity.

2. General computational details and considerations

We have used the Density Functional Theory (DFT) in the Kohn-Sham formulation to optimize all the stationary points presented in this manuscript. Geometries of all the stationary points were fully optimized at the M06[1]/def2svpp[2,3] computational level. The effect of solvent (DMA) was modelled using the polarizable continuum model (PCM)[4] with the default parameters implemented in the Gaussian 09 package.[5] Explicit solvation was also included in some instances since the solvent has the potential ability to coordinate the metallic center and even aid in hydrogen transferences.

All geometry optimizations have been performed using tight convergence criteria in the SCF and requesting a pruned (99.590) grid to guarantee the accuracy of the reported results. Moreover, calculations were performed considering 1.0 atm and 298.1 K to properly simulate the reaction conditions.

Frequency analysis was used to establish the nature of all optimized structures as either minima or transition structures. For all stationary points, the stability of the wave function was examined.[6,7,8] When different spin-states are possible for a stationary point those states were explored by running single point energy calculations starting from the optimized structure with the expected multiplicity. Single point energies for the different possible multiplicities are specified in the **Cartesian section**.

IRC calculations[9,10] were conducted for important transition states to ensure their connectivity with the expected reactants and products. The nudged elastic band method was used to locate difficult transition states[11] When the substrates showed conformational freedom, conformational analysis was performed manually, it must be indicated that only the most stable conformer of each stationary point was considered and reported unless otherwise indicated. The visualization of the reported structures was performed using MOLDEN.[12] The representation of the structures here presented were generated using CYLView.[13]

The reduction steps constitute a troublesome point in this research since they involve metallic Zn. We have worked here under the consideration that in the presence of such a coordinating solvent such as DMA part of the metallic Zn will be efficiently solvated and leached in the form of $Zn(DMA)_3$. Different number of solvent molecules coordinated to the metal center has been explored obtaining that three is the enthalpically preferred coordination.

Table S1. Data on the solvation of metallic Zn.[a]

ID	Im Freqs	Stable	SCF	SCF+ZPVE	H[b]	G[c]
**DMA**	-	Yes	-287.4093664	-287.280614	-287.272025	-287.312298
**Zn**	-	Yes	-1779.098406	-1779.098406	-1779.096045	-1779.114283
**Zn(DMA)**	-	Yes	-2066.517272	-2066.387867	-2066.377097	-2066.425476
**$Zn(DMA)_2$**	-	Yes	-2353.937241	-2353.676407	-2353.657118	-2353.723942
**$Zn(DMA)_3$**	-	Yes	-2641.356003	-2640.965713	-2640.937012	-2641.026676

[a] SCF energies correspond to the electronic energies expressed in a.u. Imaginary frequencies are expressed in cm^{-1}. [b] H denotes the Sum of electronic and thermal Enthalpies. [c] G denotes the Sum of electronic and thermal Free Energies.

3. Synthesis of starting materials

3.1 Synthesis of *N*-trifluoroethoxyphthalimide **2**

Compound **2** was synthesized from *N*-hydroxyphthalimide (NHPI) and 2,2,2-trifluoroethyl trifluoromethanesulfonate according to the literature.[14] The latter was prepared from trifluoroethanol (TFE) and triflic anhydride following a described procedure.[15] Please note that the compound is also commercialy available (CAS No. 6226-25-1, Sigma Aldrich Cat. No. 752924).

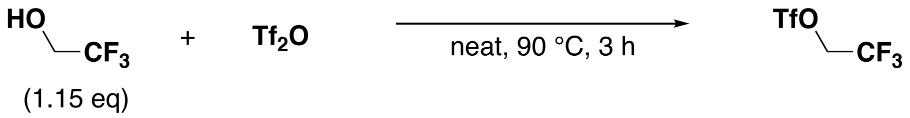

In a heat-gun dried, 25 mL two-necked round bottom flask under N_2 atmosphere equipped with reflux condenser were added TFE (4.17 g, 3.0 mL, 41.7 mmol, 1.15 eq) and triflic anhydride (10.00 g, 6 mL, 35.7 mmol, 1 eq). The mixture was stirred for 15 minutes at rt (**CAUTION! Heat is produced when mixing the two liquids**) and then refluxed for 3 hours at 90 °C.

Under N_2 flow, the reflux condenser was removed, and the flask connected to a previously heat-gun dried distillation apparatus equipped with two receiving flasks also under N_2 flow. Fractionate distillation under N_2 atmosphere at atmospheric pressure was carried out (oil bath temperature ca. 120 °C), discarding the head distillate (ca. 0.5 mL, containing residual TFE) to obtain pure 2,2,2-trifluoroethyl trifluoromethanesulfonate (7.46 g, 32.1 mmol, 90 % yield). Spectral data are in accordance with the literature.[15]

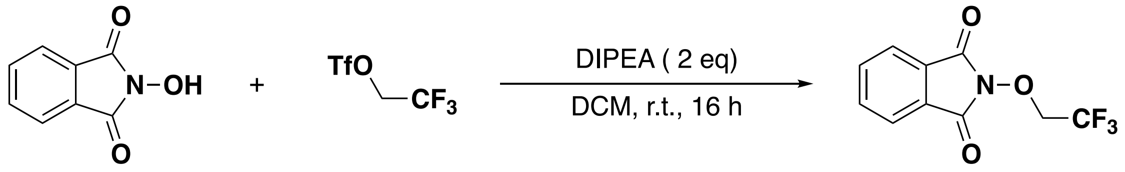

2

A 250 mL three -necked round bottom flask was evacuated and back-filled with N_2 for three times. NHPI (2.90 g, 17.8 mmol, 1 eq) was added to the flask, followed by DCM (50 mL). DIPEA (4.60 g, 6.2 mL, 35.6 mmol, 2 eq) was added slowly to the suspension, immediately

producing a color change to orange/red, indicating formation of NHPI anion. The mixture was stirred until homogeneous (5 to 10 minutes). At this point 2,2,2-trifluoroethyl trifluoromethanesulfonate (4.53 g, 19.5 mmol, 1.1 eq) was added in one portion, and the mixture was stirred overnight under N_2 atmosphere.

The reaction was quenched with H_2O, transferred to a separatory funnel, the aqueous phase extracted with DCM, the combined organic phases dried on anhydrous Na_2SO_4 and the solvent removed under reduced pressure. Flash chromatography on silica gel (5:1 *n*Hex/EtOAc until a 365 nm light absorbing impurity is eluted, then 3.5:1 *n*Hex/EtOAc) furnished *N*-trifluoroethoxyphthalimide as a white, fluffy solid. (3.14 g, 12.8 mmol, 72 % yield). Spectral data are in accordance with the literature.[14]

Additional notes:

1. Special care must be taken in properly sealing all the glass joints before the distillation. This is to avoid any leakage of TfOH that would react with grease in the joints, making the system not well sealed, ultimately leading to very slow distillation, loss of product as well as posing safety issues.

2. We noticed that compound **2** is not stable in the reaction crude upon dryness, probably due to sensitivity to strongly basic conditions. Therefore, solvent should be evaporated only right before flash chromatography. Anyway, compound **2** resulted stable in the reaction mixture in the presence of solvent for at least one day.

3.2 Synthesis of *N*-pentafluoropropoxyphthalimide **2'**

Compound **2'** was synthesized from *N*-hydroxyphthalimide (NHPI) and 2,2,3,3,3-pentafluoro-1-propanol via an adapted literature procedure described for the synthesis of **2**.[16] The corresponding triflate ester was prepared in situ and reacted with *N*-hydroxyphthalimide in the presence of base.

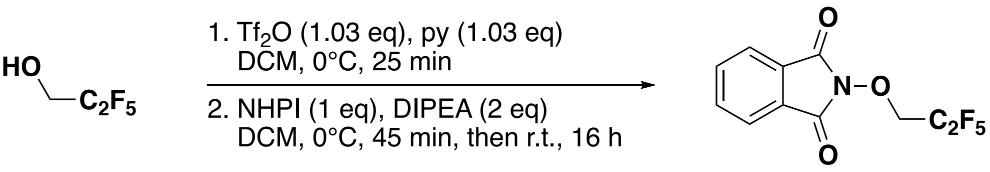

(1.03 eq) **2'**

In a heat-gun dried, 25 mL three-necked round bottom flask equipped with a dropping funnel under N_2 atmosphere were added Tf_2O (5.13 mmol, 1.45 g, 0.86 mL) and DCM (8 mL). After cooling to 0°C, a solution of 2,2,3,3,3-pentafluoro-1-propanol (5.13 mmol, 0.77 g, 0.64 mL) and pyridine (5.13 mmol, 0.41 g, 0.41 mL) in DCM (2 mL) was added dropwise. After stirring at 0°C for 25 minutes, a solution of NHPI (5 mmol, 0.81 g) and DIPEA (10 mmol, 1.29 g, 1.8 mL) in DCM (5.75 mL) was added dropwise (this solution was prepared in a heat-gun dried two neck round bottom flask and transferred via syringe). The mixture was stirred at 0°C for 45 minutes and then at r.t. for 16 h. The reaction was quenched with 2 N HCl (*ca.* 15 mL), transferred to a separatory funnel, the aqueous phase extracted with DCM, the combined organic phases washed with H_2O and brine, dried on anhydrous Na_2SO_4 and the solvent removed under reduced pressure. Flash chromatography on silica gel (5:1 *n*Hex/EtOAc) furnished *N*-trifluoropropoxyphthalimide as a white solid. (398 mg, 1.35 mmol, 27 % yield).

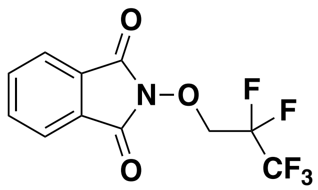

2'. White solid. **MP** = 89 – 91 °C. **^{1}H NMR** (400 MHz, $CDCl_3$) δ = 7.88 – 7.83 (m, 2H), 7.80 – 7.75 (m, 2H), 4.64 (t, *J* = 12.9 Hz, 2H); **^{13}C NMR** (100 MHz, $CDCl_3$) δ = 162.4 (2C), 134.9 (2C), 128.6 (2C), 123.9 (2C), 72.4 (t, *J* = 25.4 Hz), the CF_2 and CF_3 carbons of the pentafluoroethyl group were not detected; **^{19}F NMR** (377 MHz, $CDCl_3$) δ = -83.73 (s, 3F), -124.26 (t, *J* = 12.9 Hz, 2F). **GC-MS**: 295 (13), 276 (4), 176 (3), 147 (12), 105 (100).

3.3 Synthesis or aryl iodides

Aryl iodides **1a**, **1b**, **1i-1n**, **1p-1v**, **1y** are commercially available and were used without as received.

Aryl iodides **1c**[17], **1d**[18], **1e**[19], **1w**[20] were prepared from 4-iodophenol according to the literature.

Aryl iodides **1f**[21], **1g**[22], **1h**[23] were prepared from 4-iodoaniline according to the literature.

Aryl iodide **1o**[24] was prepared from 4-iodobenzoic acid according to the literature.

Aryl iodide **1x**[25] was prepared from 1,4-diiodobenzene according to the literature.

Aryl iodides **1z**[26] and **1a'**[27] were prepared following a literature procedure by a two-step sequence: Suzuki cross coupling between 4-(trimethylsilyl)phenylboronic acid and 4-iodobenzonitrile (**1z**) or 3-fluoroiodobenzene (**1a'**) followed by *ipso*-iodination with ICl.[27]

Aryl iodide **1c'** was prepared from 4-iodobenzyl bromide and 4-hydroxybenzaldehyde according to the literature.[28]

Aryl iodides **6a**[29], **6b**[30] and **6c**[31] were prepared from 4-iodobenzoyl chloride and the corresponding alcohol or amine according to the literature.

3.4 Synthesis of Ligand **L5** and nickel complex [Ni(**L5**)Cl_2].

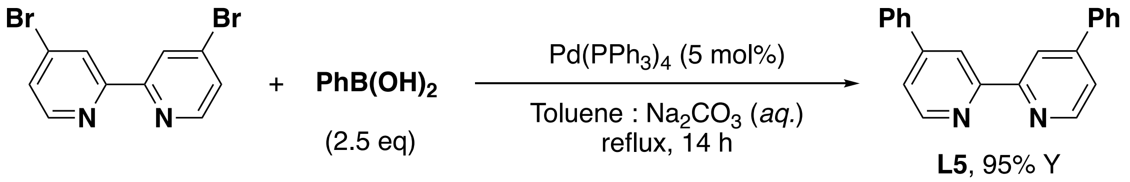

Ligand **L5** was prepared according to the literature from 4,4'-dibromo-2,2'-bipyridine and phenyl boronic acid via Suzuki Cross Coupling.[32]

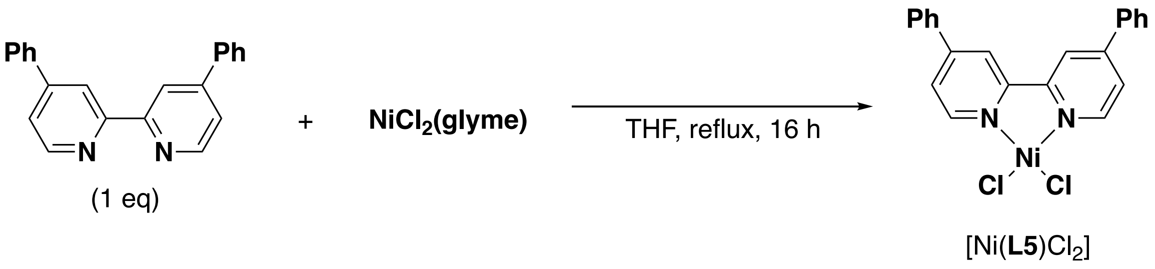

Nickel complex [Ni(**L5**)Cl_2] was prepared via modification of a described procedure for the synthesis of [Ni(dtbbpy)Cl_2],[33] as follows: in a heat-gun dried Schlenk tube were added **L5** (0.3 mmol, 92.4 mg, 1 eq), $NiCl_2$(glyme) (0.3 mmol, 65.9 mg, 1 eq) and dry THF (2 mL). The heterogeneous mixture was refluxed for 18 h, cooled to room temperature and diluted with EtOAc (6 mL). The solid was separated by centrifugation, washed with EtOAc and dried under vacuum to obtain [Ni(**L5**)Cl_2] as a pale green solid (0.285 mmol, 124.8 mg, 95% yield).

4. Additional optimization data

4.1. **Table S2**: Initial screening on substrate **1p**

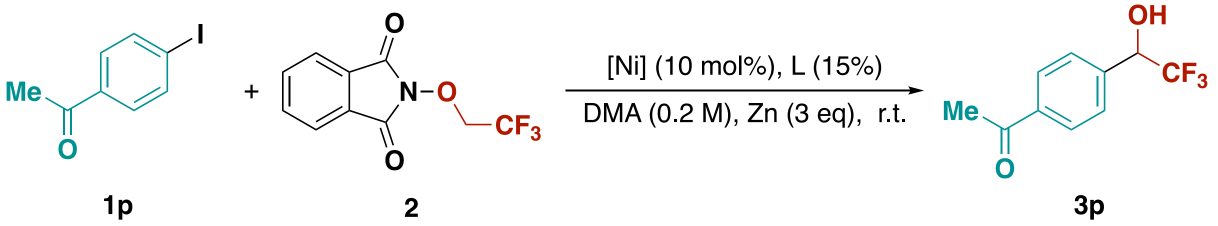

Entry[a]	**1p** : **2**	[Ni]	**L**	Other variations	**3p** Yield (%)[b]
1	1 : 1.5	$NiCl_2$(glyme)	**L6**	/	50
2	1.5 : 1	$NiCl_2$(glyme)	**L6**	/	28
3	1 : 1.5	NiI_2	**L6**	/	36
4	1 : 1.5	$NiCl_2$(glyme)	**L6**	$MgCl_2$ (1.5 eq)	traces
5	1 : 3	$NiCl_2$(glyme)	**L6**	/	72
6	1 : 3	$NiCl_2$(glyme)	**L3**	/	82
7	1 : 3	$NiCl_2$(glyme)	**L3**	DMF as solvent	55
8	1 : 3	$NiCl_2$(glyme)	**L3**	TBAI (50 mol%)	76
9	1 : 3	[Ni(**L3**)Cl_2][c]	/	2 eq of Zn	93

[a] All reactions were conducted on 0.1 mmol scale of the limiting reagent (**1p** or **2a** depending on the ratio chosen) and checked after 16 h. [b] Yields determined on the crude mixture by ^{1}H NMR with 1,3-dimethoxybenzene as internal standard after work up. [c] 10 mol% of preformed metal complex was used.

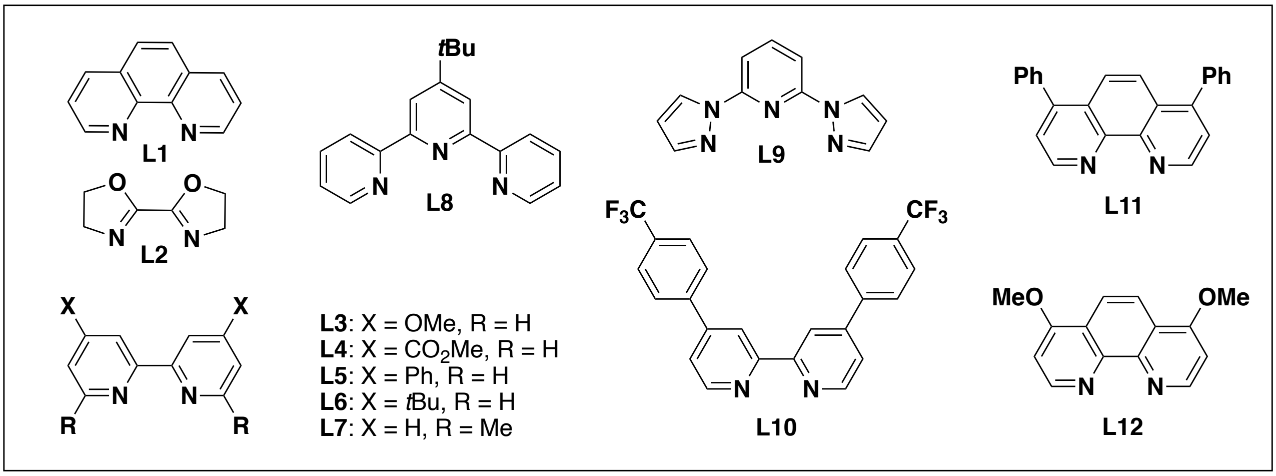

Optimal conditions for substrate **1p** (entry 8) turned out not to be general for other substrates. These conditions appear to be limited to substrates carrying EWGs, and more electron-rich aryl iodides failed to provide satisfactory conversions. Therefore, we further explored conditions choosing **1u** as model substrate (*i.e.* non-volatile and easily detectable product) and the results are summarized in the **Table S3**.

4.2. **Table S3**: Screening on substrate **1u**

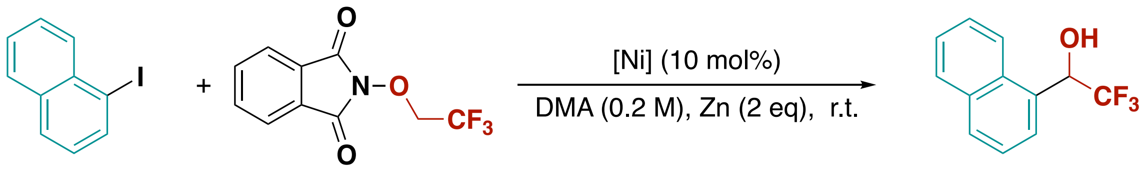

1u **2** **3u**

Entry[a]	[Ni]	Other variations	**3u** Yield (%)[b]
1	[Ni(**L3**)Cl_2]	/	14
2	[Ni(**L3**)Cl_2]	TMSCl (1 eq)	32
3	[Ni(**L1**)Cl_2]	/	18
4	[Ni(**L1**)Cl_2]	DMSO as solvent	14
5	[Ni(**L1**)Cl_2]	DMF as solvent	10
6	[Ni(**L1**)Cl_2]	NMP as solvent	11
7	[Ni(**L1**)Cl_2]	DMA:dioxane (2:3) as solvent	18
8	[Ni(**L1**)Cl_2]	TMSCl (1 eq)	61
9	[Ni(**L1**)Cl_2]	TMSCl (2 eq)	29
10	[Ni(**L1**)Cl_2]	TMSCl (0.5 eq)	50
11	[Ni(**L1**)Cl_2]	TMSCl (0.75 eq)	56

[a] All reactions were conducted on 0.1 mmol scale of **1u** (**1u**:**2** = 1:3) and checked after 16 h. [b] Yields determined on the crude mixture by ^{19}F NMR with trifluorotoluene as an internal standard, after quenching.

TMSCl was identified as a useful additive to ensure reproducibility, as some reactions showed sluggish initiation periods that resulted in low yields and incomplete conversion. In each case it was used, its addition provided similar or higher yields than the reaction run in its absence.

Again, optimal conditions for substrate **1u** (entry 8) demonstrated to be specific for this substrate and proved low-yielding for several others. For example, electron-rich substrate **1b** bearing a 4-OMe group was unreactive under these optimized conditions, as well as with optimal conditions for substrate **1p**.

Therefore, we chose substrate **1a** as unbiased model iodide for the final optimization, and the most significant results are summarized in the main text (**Table 1**). The following two tables present additional optimization data.

4.3. **Table S4**: Additional screening data on substrate **1a**

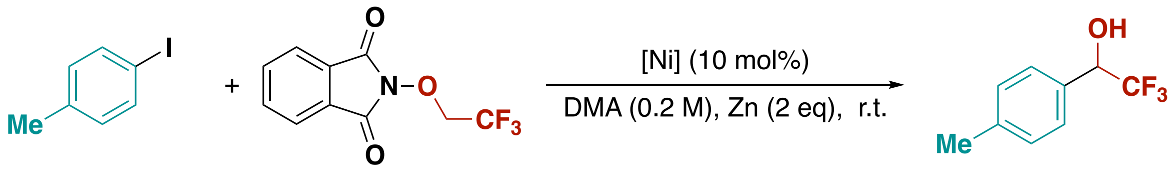

1a **2** **3a**

Entry[a]	[Ni]	L	Other variations	**3a** Yield (%)[b]
1	[Ni(**L1**)Cl_2][c]	/	/	36
2	[Ni(**L1**)Cl_2][c]	/	40 °C	22
3	[Ni(**L1**)Cl_2][c]	/	50 °C	16
4[d]	[Ni(**L1**)Cl_2][c]	/	TMSCl (1 eq)	36
5	[Ni(**L1**)Cl_2][c]	/	TMSCl (1 eq) and HNPhth (1 eq)	16
6	[Ni(**L1**)Cl_2][c]	/	TMSCl (1 eq) and KNPhth (1 eq)	NR
7	[Ni(**L1**)Cl_2][c]	/	TMSCl (1 eq) and TMG (2 eq)	0
8	[Ni(**L1**)Cl_2][c]	/	TMSCl (1 eq) and 20% of [Ni]	33
9	[Ni(**L1**)Cl_2][c]	/	TMSCl (1 eq) and NaI (1 eq)	40
10	$NiCl_2$(glyme)	**L12**	/	21
11	$NiCl_2$(glyme)	**L9**	TMSCl (1 eq)	3
12	$NiCl_2$(glyme)	**L10**	TMSCl (1 eq) and NaI (1 eq)	9
13	$NiCl_2$(glyme)	**L11**	TMSCl (1 eq)	38
14	$NiCl_2$(glyme)	**L11**	TMSCl (1 eq) and NaI (1 eq)	62
15	$NiCl_2$(glyme)	**L3**	TMSCl (1 eq) and NaI (1 eq)	53
16	[Ni(**L3**)Cl_2][c]	/	TMSCl (1 eq) and NaI (1 eq)	77

[a] All reactions were conducted on 0.1 mmol scale of **1a** (**1a**:**2** = 1:3) and checked after 16 h. [b] Yields determined on the crude mixture by ^{19}F NMR with trifluorotoluene as the internal standard, after quenching. [c] 10 mol% of preformed metal complex was used. [d] Entry 1 in **Table 1** in the main text. NR = no reaction.

4.4. **Table S5**: Additional screening data on substrate **1a** with **L5**

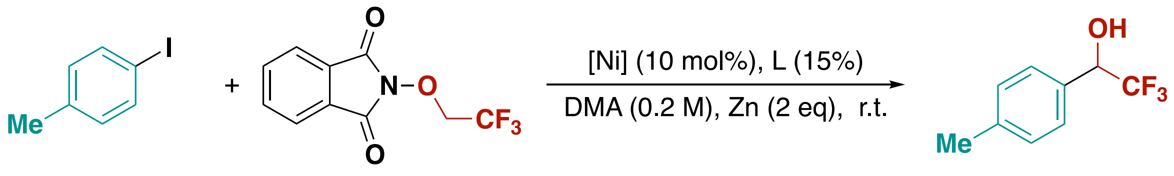

Entry[a]	[Ni]	L	Other variations	3a Yield (%)[b]
1	$NiCl_2$(glyme)	**L5**	/	22
2	$NiCl_2$(glyme)	**L5**	TMSCl (1 eq)	55
3	$NiCl_2$(glyme)	**L5**	TMSCl (1 eq) and NaI (1 eq)	74
4	$NiCl_2$(glyme)	**L5**	TMSCl (1 eq) and NaI (1.5 eq)	63
5	$NiCl_2$(glyme)	**L5**	activated Zn[c] and NaI (1 eq)	66
6	$NiCl_2$(glyme)	**L5**	TMSCl (1 eq), NaI (1 eq) and 20 mol% **L5**	67
7	$NiCl_2$(glyme)	**L5**	TMSCl (1 eq) and NaI (1 eq), and 0.1 M	71
8	$NiBr_2$(glyme)	**L5**	TMSCl (1 eq) and NaI (1 eq)	54
9	NiI_2	**L5**	TMSCl (1 eq) and NaI (1 eq)	71
10	$NiCl_2$(glyme)	**L5**	TMSCl (1 eq) and KI (1 eq)	54
11	$NiCl_2$(glyme)	**L5**	TMSCl (1 eq) and TBAI (1 eq)	20
12	$NiCl_2$(glyme)	**L5**	TMSCl (1 eq) and NaBr (1 eq)	59
13	$NiCl_2$(glyme)	**L5**	TMSCl (1 eq) and $MgBr_2 \cdot (Et_2O)$ (1 eq)	28
14	$NiCl_2$(glyme)	**L5**	TMSCl (1 eq) and LiCl (1 eq)	traces
15	$NiCl_2$(glyme)	**L5**	TMSCl (1 eq), NaI (1 eq) and pyridine (25 mol%)	72

16	$NiCl_2$(glyme)	**L5**	TMSCl (1 eq), NaI (1 eq) and 1,5-hexadiene (75 mol%)	75
17[d,e]	[Ni(**L5**)Cl_2][f]	/	TMSCl (1 eq) and NaI (1 eq)	95

[a] All reactions were conducted on 0.1 mmol scale **1a** (**1a**:**2** = 1:3) and checked after 16 h. [b] Yields determined on the crude mixture by ^{19}F NMR with trifluorotoluene as internal standard, after quenching. [c] 1.6 g of commercial Zn dust were stirred with 10 mL aqueous 2% HCl for 4 minutes, washed with 20 mL H_2O (x3) by decanting the solution each time, filtered and washed with EtOH (5 mL), Acetone (10 mL), Et_2O (5 mL). The solid was dried under vacuum at 90°C for 10 minutes and stored under N_2. [d] Entry 12 in Table 1 in the main text. [e] Reaction time = 2h. [f] 10 mol% of preformed metal complex was used.

4.5 Explorative screening of chiral ligands

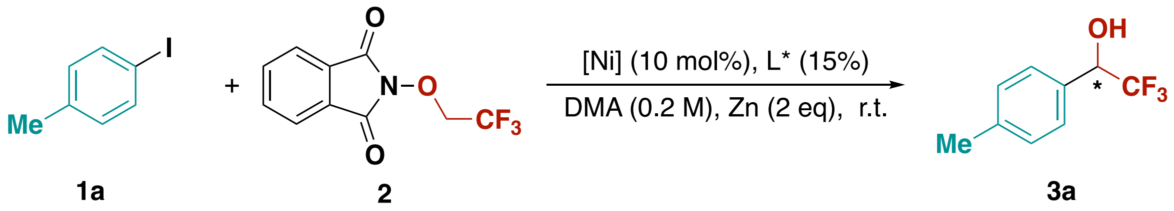

After optimization with ligand **L5**, we tested several chiral ligands to assess the feasibility of an enantioselective version of the protocol. Unfortunately, we did not record any encouraging outcome, as the product was always obtained in low yields and almost racemic form.

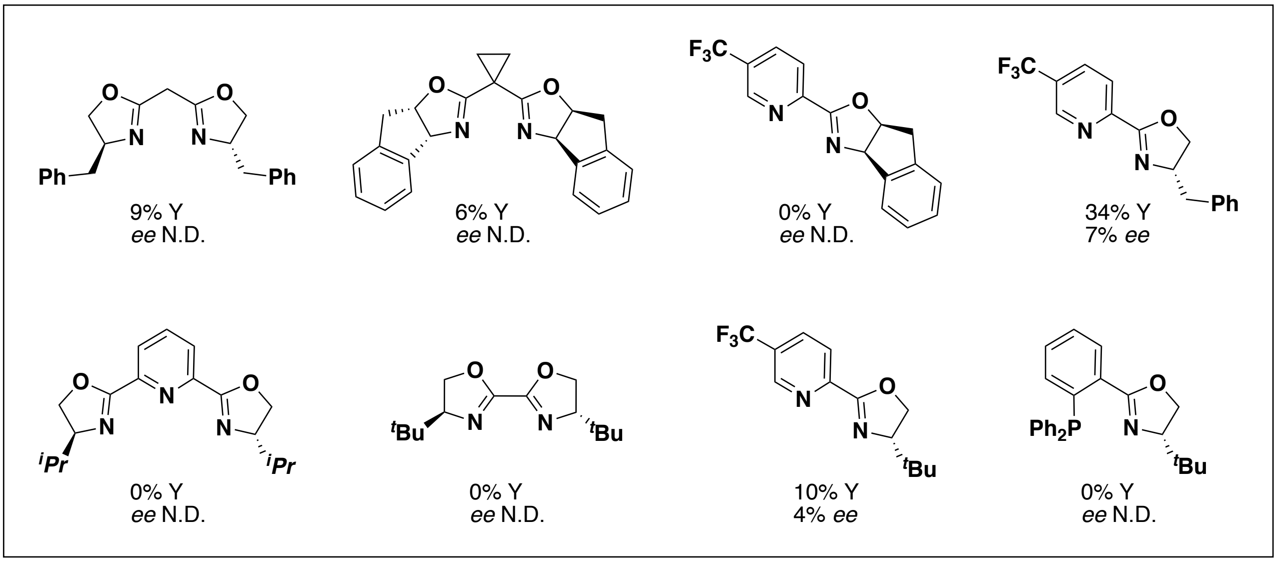

5. Ni catalyzed cross-coupling

5.1 General procedure for the Ni catalyzed cross-coupling reaction

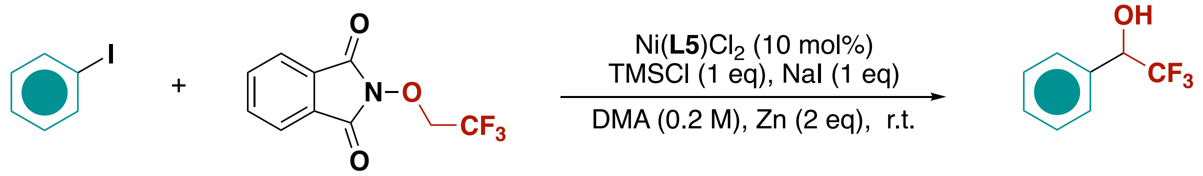

1 2 3

A heat-gun dried pressure Schlenk under N_2 atmosphere was charged with [Ni(**L5**)Cl_2] (4.4 mg, 0.01 mmol, 0.1 eq), and dry/degassed DMA (0.5 mL). The mixture was stirred until complete dissolution of the metal complex to yield an emerald-green solution (few minutes) (**Figure S1**, top left).

N-trifluoroethoxyphthalimide **2** (73.5 mg, 0.3 mmol, 3 eq), Zn dust (13 mg, 0.2 mmol, 2 eq), NaI (15 mg, 0.1 mmol, 1 eq) and, if solid, aryl iodide **1** (0.1 mmol, 1 eq) were added at once (due to its hygroscopic nature, NaI was weighted last). For liquid aryl iodides, they were added with a 50 µL Hamilton syringe after the solids (**Figure S1**, top right). The heterogeneous mixture appears olive green and turbid (**Figure S1**, bottom left). TMSCl (12.5 µL, 0.1 mmol, 1 eq) was added, and within seconds, a sudden change of color to deep red occurs (**Figure S1**, bottom right).

The Schlenk was sealed and the mixture stirred @ 1250 rpm for the indicated time (until **2** disappeared, judged by TLC). See section 4.3 for the time employed for each substrate.

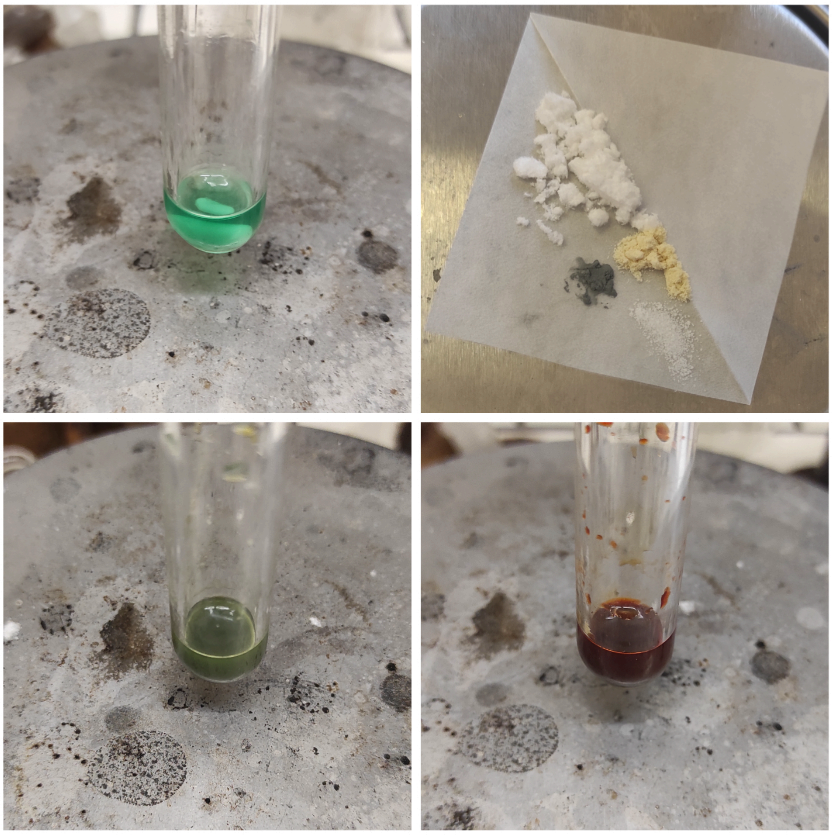

Figure S1. Top Left: [Ni(**L5**)Cl_2] solution in DMA. Top right: Solid reagents (**2**, **1p**, Zn, NaI). Bottom left: Reaction mixture after addition of all solids. Bottom right: Reaction mixture few seconds after the addition of TMSCl.

After the indicated time, the Schlenk was opened to air and EtOAc was added, followed by 2N HCl, and the biphasic mixture was shaken and stirred until all Zn was consumed (roughly 5 minutes). The red color of the organic phase gradually disappears to give a pale-yellow solution. For ^{19}F-NMR yield determination, $PhCF_3$ was added to the reaction mixture as an internal standard and an aliquot was taken without solvent evaporation. To isolate the product, the biphasic mixture was directly transferred to a separatory funnel, the aqueous phase extracted with EtOAc (10 mL x 3), and then the combined organic phases were washed twice with diluted HCl (ca 0.1 N, 10 mL x 2). The organic layer was separated, dried on anhydrous Na_2SO_4, and the solvent removed under reduced pressure. Flash chromatography on silica gel with the appropriate eluent yields the product.

Additional notes:

1. In our experience, the observation of the color change towards red does not guarantee high yields, but usually indicates initiation of the reaction and full or almost full conversion of **2** is observed when it occurs. When it does not occur, the reaction provides low yields

and low conversion. Addition of TMSCl is not mandatory to observe this change in color, but we noticed that in its presence it always occurs readily, while when it is not added, this phenomenon seems to be more substrate- and conditions dependent.

2. Vigorous stirring (1250 rpm) is beneficial given the heterogeneous nature of the reductant used, and the reaction should not be run at lower stirring speeds.[34]

3. For product **3g**, due to its basic nature, the aqueous phase was brought to pH = 10 by adding saturated Na_2CO_3 solution prior to extraction.

4. For products **3e**, **3f**, **3h**, **3p**, **3y**, **3z**, **3a'**, **3c'**, **7a**, **7c** chromatographic separation from phthalimide coproduct was troublesome, therefore after FC a basic wash (aqueous 1N NH_3 / Et_2O) was carried out to obtain the pure compounds.

5. Products **3a**, **3b**, **3d**, **3i**, **3j**, **3k**, **3l**, **3n**, **3q**, **3r**, **3s** are volatile, therefore special care must be taken when removing the solvent. In particular, products **3k**, **3n**, **3q**, **3r** were prepared on 0.2 mmol scale and Et_2O was used in place of EtOAc for extraction and FC to allow product isolation without significant loss.

6. Product **7c** is scarcely soluble in EtOAc, therefore *i*PrOAc was used for extraction and chromatographic purification.

7. For product **3y** the General Procedure was slightly modified by doubling the amount of [Ni(**L5**)Cl_2], Zn and **2**.

8. For product **3b'**, *N*-pentafluoropropoxyphthalimide **2'** (88.5 mg, 0.3 mmol, 3 eq) was used instead of **2**.

5.2. Unsuccessful substrates

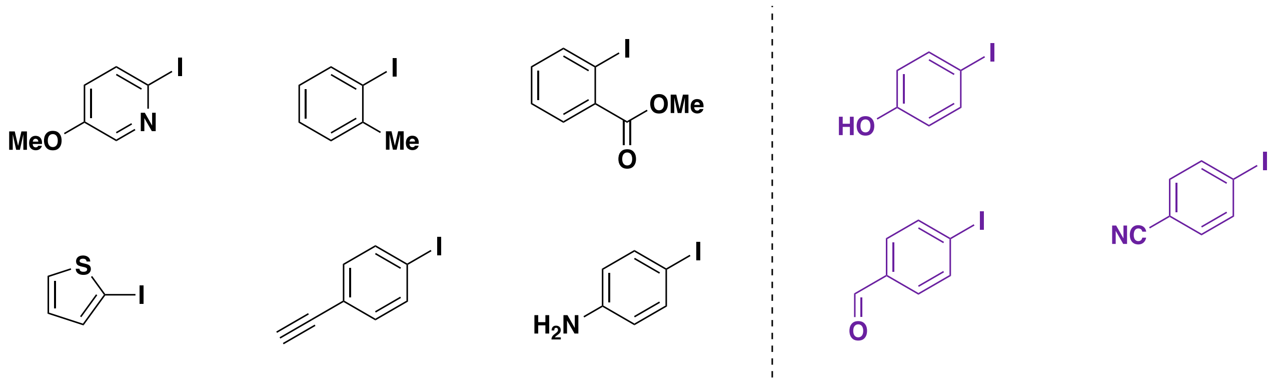

Substrates on the left (in black) gave the corresponding products in less than 15% yield. Substrates on the right (in purple) could give the product in more than 50% yield (based on ^{19}F-NMR) but it resulted impossible to separate from phthalimide by flash chromatography and resulted unstable to basic washes. Therefore, they were not included in the scope.

5.3 Characterization data of products **3** and **7**

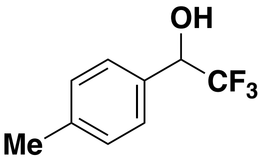

3a. Viscous colorless oil. Reaction time: 2 h. FC eluent: *n*Hex/EtOAc: 7:1. Yield = 88%, (0.088 mmol, 16.7 mg). **^{1}H NMR** (400 MHz, $CDCl_3$) δ = 7.38 – 7.31 (m, 2H), 7.23 – 7.18 (m, 2H), 4.97 (q, *J* = 6.8 Hz, 1H), 2.54 (bs, 1H), 2.36 (s, 3H); **^{13}C NMR** (100 MHz, $CDCl_3$) δ = 139.5, 131.0, 129.3 (2C), 127.3 (q, *J* = 1.0 Hz, 2C), 124.3 (q, *J* = 282.0 Hz), 72.7 (q, *J* = 31.9 Hz), 21.2; **^{19}F NMR** (377 MHz, $CDCl_3$) δ = -78.47 (d, *J* = 6.7 Hz, 3F). This is a known compound and spectral data are in accordance with the literature.[35]

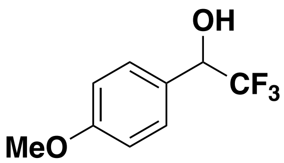

3b. Viscous colorless oil. Reaction time: 3 h. FC eluent: *n*Hex/EtOAc: 6:1. Yield = 61%, (0.061 mmol, 12.6 mg). **^{1}H NMR** (400 MHz, $CDCl_3$) δ = 7.42 – 7.33 (m, 2H), 6.97 – 6.88 (m, 2H), 4.95 (qd, *J* = 6.7, 4.2 Hz, 1H), 3.81 (s, 3H), 2.53 (d, *J* = 4.3 Hz, 1H); **^{13}C NMR** (100 MHz, $CDCl_3$) δ = 160.5, 128.7 (q, *J* = 1.0 Hz, 2C), 126.1 (q, *J* = 1.1 Hz), 124.3 (q, *J* = 281.9 Hz), 114.0 (2C), 72.5 (q, *J* = 32.0 Hz), 55.3; **^{19}F NMR** (377 MHz, $CDCl_3$) δ = -78.60 (d, *J* = 6.8 Hz, 3F). This is a known compound and spectral data are in accordance with the literature.[36]

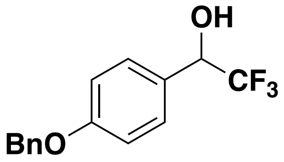

3c. White solid. Reaction time: 5 h FC eluent: *n*Hex/EtOAc: 6:1. Yield = 63%, (0.063 mmol, 17.8 mg). **MP** = 101 – 103 °C. **^{1}H NMR** (400 MHz, $CDCl_3$) δ = 7.45 – 7.29 (m, 7H), 7.03 – 6.96 (m, 2H), 5.07 (s, 2H), 4.97 – 4.91 (m, 1H), 2.56 (d, *J* = 4.1 Hz, 1H); **^{13}C NMR** (100 MHz, $CDCl_3$) δ ^{13}C NMR (101 MHz, $CDCl_3$) δ 159.7, 136.6, 128.8 (2C), 128.6 (2C), 128.1, 127.5 (2C), 126.3, 124.30 (q, *J* = 282.0 Hz), 114.9 (2C), 72.5 (q, *J* = 32.0 Hz), 70.1; **^{19}F NMR** (377 MHz, $CDCl_3$) δ = -78.54 (d, *J* = 6.8 Hz, 3F). This is a known compound and spectral data are in accordance with the literature.[37]

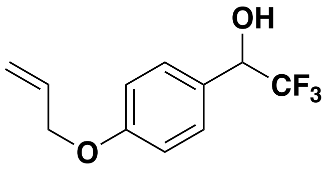

3d. White solid. Reaction time: 5 h. FC eluent: *n*Hex/EtOAc: 6:1. Yield = 50%, (0.050 mmol, 11.8 mg). **MP** = 51 – 53 °C. **^{1}H NMR** (400 MHz, $CDCl_3$) δ = 7.41 – 7.36 (m, 2H), 6.99 – 6.92 (m, 2H), 6.05 (ddt, *J* = 17.3, 10.5, 5.3 Hz, 1H), 5.42 (dq, *J* = 17.3, 1.7 Hz, 1H), 5.30 (dq, *J* = 10.5, 1.4 Hz, 1H), 4.96 (qd, *J* = 6.6, 3.6 Hz, 1H), 4.55 (dt, *J* = 5.3, 1.6 Hz, 2H), 2.47 (d, *J* = 4.3 Hz, 1H); **^{13}C NMR** (100 MHz, $CDCl_3$) δ = 158.5, 131.9, 127.7 (2C), 125.2, 123.3 (q, *J* = 282.2 Hz), 116.9, 113.8 (2C), 71.46 (q, *J* = 32.1 Hz), 67.8; **^{19}F NMR**

(377 MHz, $CDCl_3$) δ = -78.53 (d, *J* = 6.8 Hz, 3F). This is a known compound and spectral data are in accordance with the literature.[38]

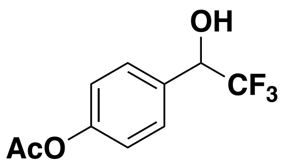

3e. White solid. Reaction time: 5 h. FC eluent: *n*Hex/EtOAc: 4.5:1. Yield = 62%, (0.062 mmol, 14.5 mg). **MP** = 95 – 98 °C. **^{1}H NMR** (400 MHz, $CDCl_3$) δ = 7.52 – 7.45 (m, 2H), 7.17 – 7.08 (m, 2H), 5.01 (q, *J* = 6.7 Hz, 1H), 2.66 (bs, 1H), 2.29 (s, 3H); **^{13}C NMR** (100 MHz, $CDCl_3$) δ = 169.2, 151.5, 131.5, 128.6 (2C), 124.1 (q, *J* = 282.2 Hz), 121.8 (2C), 72.3 (q, *J* = 32.2 Hz), 21.1; **^{19}F NMR** (377 MHz, $CDCl_3$) δ = -78.46 (d, *J* = 6.8 Hz, 3F). This is a known compound and spectral data are in accordance with the literature.[39]

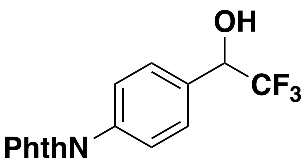

3f. White solid. Reaction time: 16 h. FC eluent: *n*Hex/EtOAc: 2:1. Yield = 74%, (0.074 mmol, 23.8 mg). **MP** = 202 – 204 °C. **^{1}H NMR** (400 MHz, acetone-*d*$_6$) δ = 7.97 – 7.93 (m, 2H), 7.93 – 7.90 (m, 2H), 7.74 – 7.68 (m, 2H), 7.61 – 7.54 (m, 2H), 5.97 (d, *J* = 5.4 Hz, 1H), 5.32 (qd, *J* = 7.1, 5.2 Hz, 1H); **^{13}C NMR** (100 MHz, acetone-*d*$_6$) δ = 166.8 (2C), 135.3 (q, *J* = 1.6 Hz), 134.5 (2C), 133.0, 132.0 (2C), 128.1 (q, 0.8 Hz, 2C), 126.8 (2C), 125.0 (q, *J* = 281.9 Hz), 123.3 (2C), 71.2 (q, *J* = 31.2 Hz); **^{19}F NMR** (377 MHz, acetone-*d*$_6$) δ = -78.64 (d, *J* = 7.0 Hz, 3F); **HRMS (APCI)** m/z: $[M+H]^+$ calcd. for $C_6H_{11}F_3NO_3$ 322.0691; found 322.0682.

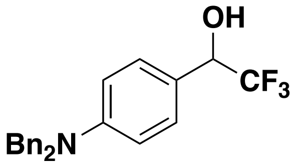

3g. White solid. Reaction time: 3 h. FC eluent: *n*Hex/EtOAc: 4:1. Yield = 73%, (0.073 mmol, 27.1 mg). **MP** = 152 – 154 °C. **^{1}H NMR** (400 MHz, $CDCl_3$) δ = 7.32 – 7.23 (m, 4H), 7.22 – 7.14 (m, 8H), 6.68 – 6.64 (m, 2H), 4.79 (q, *J* = 7.0 Hz, 1H), 4.60 (s, 4H), 2.27 (bs, 1H); **^{13}C NMR** (150 MHz, $CDCl_3$) δ = 149.0, 137.0 (2C), 127.7 (4C), 127.6 (2C), 126.0 (2C), 125.5 (4C), 123.5 (q, *J* = 281.8 Hz), 120.8, 111.1 (2C), 71.70 (q, *J* = 32.0 Hz), 53.1 (2C); **^{19}F NMR** (565 MHz, $CDCl_3$) δ = -78.3 (d, *J* = 6.9 Hz, 3F); **HRMS (ESI)** m/z: $[M+H]^+$ calcd. for $C_{22}H_{20}F_3NO$ 372.1575; found 372.1567.

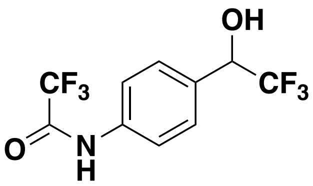

3h. White solid. Reaction time: 16 h. FC eluent: *n*Hex/Acetone: 3:1. Yield = 63%, (0.063 mmol, 18.1 mg). **MP** = 147 – 150 °C. **^{1}H NMR** (400 MHz, acetone-*d*$_6$) δ = 10.38 (bs, 1H), 7.80 – 7.74 (m, 2H), 7.61 – 7.54 (m, 2H), 5.97 (d, *J* = 5.5 Hz ,1H), 5.26 – 5.16 (m, 1H), peaks at 10.38 ppm and 5.97 ppm show an integral value lower than unity, probably due to partial

H-D exchange with the solvent; **^{13}C NMR** (100 MHz, acetone-d_6) δ = 154.9 and 154.8 (q, *J* = 37.6 Hz, two isotopomeric signals), 137.1 and 137.0 (two isotopomeric signals), 133.1 (two isotopomeric signals), 128.3 (2C), 125.0 (q, *J* = 281.9 Hz), 120.5 and 120.4 (2C, two isotopomeric signals), 116.0 (q, *J* = 287.9 Hz), 71.1 and 70.9 (q, *J* = 31.2 Hz, two isotopomeric signals), some signals appear split due to partial H-D exchange with the solvent giving rise to two isotopomers; **^{19}F NMR** (377 MHz, $CDCl_3$) δ = -76.23 (s, 3F), -78.83 (d, *J* = 7.3 Hz, 3F); **HRMS (ESI)** m/z: $[M-H]^-$ calcd. for $C_{10}H_6F_6NO_2$ 286.0308; found 286.0323.

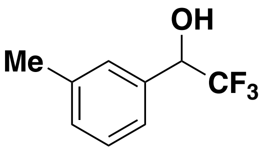

3i. Viscous colorless oil. Reaction time: 3 h. FC eluent: *n*Hex/EtOAc: 7:1. Yield = 62%, (0.062 mmol, 11.8 mg). **^{1}H NMR** (400 MHz, $CDCl_3$) δ = 7.31 – 7.25 (m, 3H), 7.23 – 7.19 (m, 1H), 4.97 (q, *J* = 6.7 Hz, 1H), 2.51 (bs, 1H), 2.37 (s, 3H); **^{13}C NMR** (100 MHz, $CDCl_3$) δ = 138.4, 133.9, 130.3, 128.5, 128.0 (q, *J* = 1.4 Hz), 124.5 (q, *J* = 1.1 Hz), 124.2 (q, *J* = 282.1 Hz), 72.9 (q, *J* = 31.9 Hz), 21.4; **^{19}F NMR** (377 MHz, $CDCl_3$) δ = -78.32 (d, *J* = 6.8 Hz, 3F). This is a known compound and spectral data are in accordance with the literature.[35]

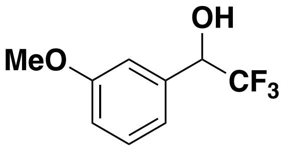

3j. Colorless oil. Reaction time: 6 h. FC eluent: *n*Hex/EtOAc: 6:1. Yield = 64%, (0.064 mmol, 13.2 mg). **^{1}H NMR** (400 MHz, $CDCl_3$) δ = 7.35 – 7.28 (m, 1H), 7.07 – 6.99 (m, 2H), 6.93 (ddd, *J* = 8.4, 2.6, 1.1 Hz, 1H), 4.98 (q, *J* = 6.7 Hz, 1H), 3.81 (s, 3H), 2.63 (bs, 1H); **^{13}C NMR** (100 MHz, $CDCl_3$) δ = 159.7, 135.4 (q, *J* = 1.2 Hz), 129.7, 124.2 (q, *J* = 282.1 Hz), 119.7 (q, *J* = 0.8 Hz), 115.09, 112.9 (q, *J* = 1.0 Hz), 72.7 (q, *J* = 32.0 Hz), 55.3; **^{19}F NMR** (377 MHz, $CDCl_3$) δ = -78.38 (d, *J* = 6.7 Hz, 3F). This is a known compound and spectral data are in accordance with the literature.[40]

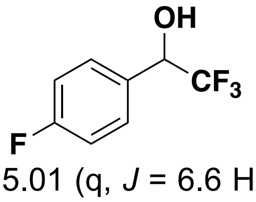

3k. Colorless oil. Reaction time: 16 h. FC eluent: *n*Hex/Et_2O: 4.5:1. Yield = 55%, (0.11 mmol, 21.40 mg, reaction performed on 0.2 mmol scale). **^{1}H NMR** (400 MHz, $CDCl_3$) δ = 7.49 – 7.41 (m, 2H), 7.14 – 7.04 (m, 2H), 5.01 (q, *J* = 6.6 Hz, 1H), the OH signal was not detected; **^{13}C NMR** (100 MHz, $CDCl_3$) δ = 163.4 (d, *J* = 248.5 Hz), 129.7 – 129.6 (m), 129.3 (dq, *J* = 8.6, 0.9 Hz, 2C), 124.1 (qd, *J* = 281.8, 1.1 Hz), 115.6 (d, *J* = 21.8 Hz, 2C), 72.2 (q, *J* = 32.2 Hz); **^{19}F NMR** (377 MHz, $CDCl_3$) δ = -76.93 (d, *J* = 6.6 Hz, 3F), -111.81 – -111.89 (m, 1F). This is a known compound and spectral data are in accordance with the literature.[35]

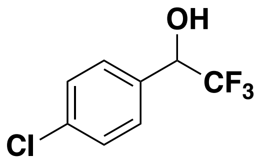

3l. Colorless oil. Reaction time: 16 h. FC eluent: *n*Hex/EtOAc: 10:1. Yield = 56%, (0.056 mmol, 11.8 mg). **^{1}H NMR** (400 MHz, $CDCl_3$) δ = 7.44 – 7.39 (m, 2H), 7.39 – 7.35 (m, 2H), 5.00 (q, *J* = 6.2 Hz, 1H), 2.69 (s, 1H); **^{13}C NMR** (100 MHz, $CDCl_3$) δ = 135.5, 132.3 (q, *J* = 1.0 Hz), 128.8 (2C), 128.8 (q, *J* = 0.9 Hz, 2C) 124.0 (q, *J* = 281.9 Hz), 72.1 (q, *J* = 32.1 Hz); **^{19}F NMR** (377 MHz, $CDCl_3$) δ = -78.59 (d, *J* = 6.4 Hz, 3F). This is a known compound and spectral data are in accordance with the literature.[35]

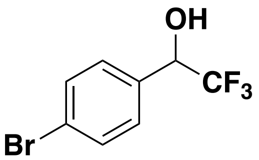

3m. Colorless oil. Reaction time: 16 h. FC eluent: *n*Hex/EtOAc: 10:1. Yield = 60%, (0.060 mmol, 15.2 mg). **^{1}H NMR** (600 MHz, $CDCl_3$) δ = 7.50 – 7.46 (m, 2H), 7.31 – 7.26 (m, 2H), 4.93 (qd, *J* = 6.7, 3.8 Hz, 1H), 2.86 (bs, 1H); **^{13}C NMR** (150 MHz, $CDCl_3$) δ = 136.7, 130.8 (2C), 128.1 (2C), 123.0 (q, *J* = 282.3 Hz), 122.7, 71.2 (q, *J* = 32.2 Hz); **^{19}F NMR** (565 MHz, $CDCl_3$) δ = -78.52 (d, *J* = 6.8 Hz, 3F). This is a known compound and spectral data are in accordance with the literature.[33] This compound was isolated as an inseparable 9:1 mixture with the corresponding *p*-iodo derivative. Relevant signals in the ^{1}H NMR spectrum for this compound are: 7.69 – 7.66 (m, 2H) and 7.16 – 7.14 (m, 2H). The ^{19}F NMR spectrum for this compound shows a doublet at -78.49 ppm. This is a known compound and spectral data are in accordance with the literature.[41]

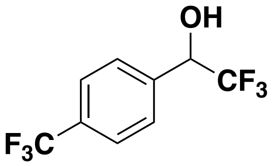

3n. Colorless oil. Reaction time: 16 h. FC eluent: *n*Hex/Et_2O: 4.5:1. Yield = 58%, (0.116 mmol, 28.4 mg, reaction performed on 0.2 mmol scale). **^{1}H NMR** (400 MHz, $CDCl_3$) δ = 7.70 – 7.64 (m, 2H), 7.64 – 7.57 (m, 2H), 5.15 – 5.04 (m, 1H), 2.82 (d, *J* = 3.9 Hz, 1H); **^{13}C NMR** (100 MHz, $CDCl_3$) δ = 137.6 – 137.5 (m), 131.7 (q, *J* = 32.6 Hz), 127.9 (q, *J* = 0.9 Hz, 2C), 125.5 (q, *J* = 3.8 Hz, 2C), 123.9 (q, *J* = 282.4 Hz) partially overlapped with 123.8 (q, *J* = 272.3 Hz), 72.2 (q, *J* = 32.2 Hz); **^{19}F NMR** (377 MHz, $CDCl_3$) δ = -62.90 (s, 3F), -78.59 (d, *J* = 6.4 Hz, 3F). This is a known compound and spectral data are in accordance with the literature.[40]

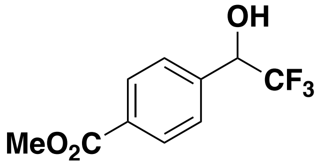

3o. White solid. Reaction time: 16 h. FC eluent: *n*Hex/EtOAc: 5:1. Yield = 73%, (0.073 mmol, 17.1 mg). **MP** = 46 – 49 °C. **^{1}H NMR** (400 MHz, $CDCl_3$) δ = 8.08 – 8.01 (m, 2H), 7.58 – 7.51 (m, 2H), 5.08 (q, *J* = 6.6 Hz, 1H), 3.91 (s, 3H), 3.02 (bs, 1H); **^{13}C NMR** (100

MHz, $CDCl_3$) δ = 166.6, 138.6 (q, *J* = 1.2 Hz), 131.1, 129.7 (2C), 127.5 (q, *J* = 0.9 Hz, 2C), 124.0 (q, *J* = 282.3 Hz), 72.3 (q, *J* = 32.1 Hz), 52.29; **^{19}F NMR** (377 MHz, $CDCl_3$) δ = -78.24 (d, *J* = 6.7 Hz, 3F). This is a known compound and spectral data are in accordance with the literature.[42]

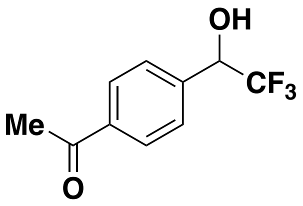

3p. White solid. Reaction time: 16 h. FC eluent: *n*Hex/EtOAc: 5:1. Yield = 68%, (0.068 mmol, 14.8 mg). **MP** = 97 – 99 °C. **^{1}H NMR** (400 MHz, $CDCl_3$) δ = 8.01 – 7.93 (m, 2H), 7.61 – 7.55 (m, 2H), 5.10 (q, *J* = 6.7 Hz, 1H), 2.88 (bs, 1H), 2.60 (s, 3H); **^{13}C NMR** (100 MHz, $CDCl_3$) δ = 197.7, 138.8 (q, *J* = 1.1 Hz), 138.0, 128.6 (2C), 127.8 (q, *J* = 0.9 Hz, 2C), 124.0 (q, *J* = 282.3 Hz), 72.3 (q, *J* = 32.2 Hz), 26.7; **^{19}F NMR** (377 MHz, $CDCl_3$) δ = -78.23 (d, *J* = 6.7 Hz, 3F). This is a known compound and spectral data are in accordance with the literature.[43]

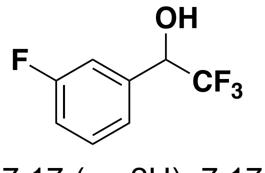

3q. Viscous colorless oil. Reaction time: 16 h. FC eluent: *n*Hex/Et_2O: 4.5:1. Yield = 65%, (0.130 mmol, 25.2 mg, reaction performed on 0.2 mmol scale). **^{1}H NMR** (400 MHz, $CDCl_3$) δ = 7.42 – 7.32 (m, 1H), 7.27 – 7.17 (m, 2H), 7.17 – 7.04 (m, 1H), 5.07 – 4.97 (m, 1H), 2.70 (d, *J* = 4.5 Hz, 1H); **^{13}C NMR** (100 MHz, $CDCl_3$) δ = 162.8 (d, *J* = 246.9 Hz), 136.2 (d, *J* = 8.7 Hz), 130.2 (d, *J* = 8.1 Hz), 124.0 (q, *J* = 282.1 Hz), 123.2 – 123.1 (m), 116.5 (d, *J* = 21.2 Hz), 114.5 (dq, *J* = 22.8, 1.0 Hz), 72.2 (qd, *J* = 32.2, 1.9 Hz); **^{19}F NMR** (377 MHz, $CDCl_3$) δ = -78.45 (d, *J* = 6.5 Hz, 3F), -112.18 (td, *J* = 9.0, 5.6 Hz, 1F). This is a known compound and spectral data are in accordance with the literature.[44]

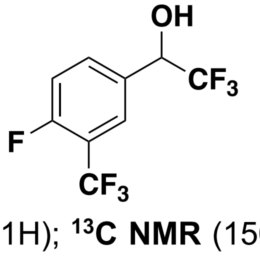

3r. Viscous colorless oil. Reaction time: 16 h. FC eluent: *n*Hex/Et_2O: 4:1. Yield = 43%, (0.086 mmol, 22.6 mg, reaction performed on 0.2 mmol scale). **^{1}H NMR** (400 MHz, $CDCl_3$) δ = 7.75 (d, *J* = 6.7 Hz, 1H), 7.73 – 7.65 (m, 1H), 7.26 (t, *J* = 9.6 Hz, 1H), 5.09 (q, *J* = 6.5 Hz, 1H), 2.88 (bs, 1H); **^{13}C NMR** (150 MHz, $CDCl_3$) δ = 159.3 (dq, *J* = 259.0, 2.1 Hz), 132.0 (d, *J* = 8.9 Hz), 129.1 (d, *J* = 3.8 Hz), 125.5 (q, *J* = 5.2 Hz), 122.8 (q, *J* = 282.0 Hz) partially overlapped with 121.2 (q, *J* = 272.4 Hz), 117.8 (qd, *J* = 33.4, 12.9 Hz), 116.3 (d, *J* = 20.9 Hz), 70.5 (q, *J* = 32.4 Hz); **^{19}F NMR** (377 MHz, $CDCl_3$) δ = -61.61 (d, *J* = 12.7 Hz, 3F), -78.76 (d, *J* = 6.3 Hz, 3F), -112.95 – -113.14 (m, 1F); **HRMS (ESI)** m/z: $[M+HCOO]^-$ calcd. for $C_{10}H_6F_7O_3$ 307.0211; found 307.0216.

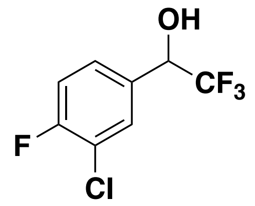

3s. Viscous colorless oil. Reaction time: 16 h. FC eluent: *n*Hex/EtOAc: 10:1. Yield = 65%, (0.065 mmol, 14.8 mg). **^{1}H NMR** (600 MHz, $CDCl_3$) δ = 7.76 (dd, *J* = 6.7, 2.3 Hz, 1H), 7.71 – 7.66 (m, 1H), 7.26 (t, *J* = 9.2 Hz, 1H), 5.09 (q, *J* = 6.5 Hz, 1H), 2.94 (bs, 1H); **^{13}C NMR** (150 MHz, $CDCl_3$) δ = 157.7 (d, *J* = 251.3 Hz), 129.8 (d, *J* = 4.0 Hz), 128.9, 126.3 (d, *J* = 7.6 Hz), 122.9 (q, *J* = 282.1 Hz), 120.5 (d, *J* = 18.1 Hz), 115.8 (d, *J* = 21.6 Hz), 70.6 (q, *J* = 32.4 Hz); **^{19}F NMR** (565 MHz, $CDCl_3$) δ = -78.62 (d, *J* = 6.8 Hz, 3F), -113.87 – -113.93 (m, 1F);). This is a known compound and spectral data are in accordance with the literature.[45]

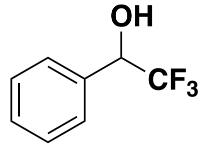

3t. Reaction time: 4 h. Compound **3t** was found to be too volatile to be isolated on 0.1 mmol scale. The yield given (70%) was determined by ^{19}F NMR analysis on the crude reaction mixture. This is a known compound and spectral data observed in the crude is in accordance with the literature.[36]

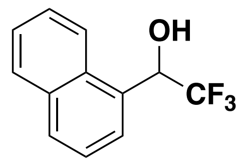

3u. Colorless oil. Reaction time: 16 h. FC eluent: *n*Hex/EtOAc: 9:1. Yield = 61%, (0.061 mmol, 13.8 mg). **^{1}H NMR** (400 MHz, $CDCl_3$) δ = 8.05 (d, *J* = 8.4 Hz, 1H), 7.95 – 7.87 (m, 2H), 7.83 (d, *J* = 7.3 Hz, 1H), 7.60 – 7.42 (m, 3H), 5.89 (q, *J* = 6.5 Hz, 1H), 2.84 (bs, 1H); **^{13}C NMR** (100 MHz, $CDCl_3$) δ = 133.7, 131.1, 130.1, 130.0 (q, *J* = 0.9 Hz), 129.0, 126.8, 125.9, 125.8 (q, *J* = 1.3 Hz), 125.2, 124.7 (q, *J* = 282.6 Hz), 122.8 (q, *J* = 1.4 Hz), 69.0 (q, *J* = 32.3 Hz); **^{19}F NMR** (377 MHz, $CDCl_3$) δ = -76.93 (d, *J* = 6.6 Hz, 3F). This is a known compound and spectral data are in accordance with the literature.[36]

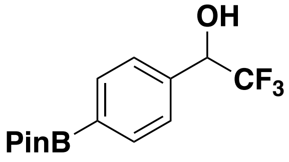

3v. White solid. Reaction time: 16 h. FC eluent: *n*Hex/EtOAc: 5:1. Yield = 43%, (0.043 mmol, 7.5 mg). **MP** = 84 – 87 °C. **^{1}H NMR** (400 MHz, $CDCl_3$) δ = 7.86 – 7.80 (m, 2H), 7.49 – 7.43 (m, 2H), 5.02 (qd, *J* = 6.7, 4.6 Hz, 1H), 2.59 (d, *J* = 4.6 Hz, 1H), 1.33 (s, 12 H); **^{13}C NMR** (100 MHz, $CDCl_3$) δ = 136.7 (q, *J* = 1.0 Hz), 135.0 (2C), 126.7 (2C), 124.1 (q, *J* = 282.3 Hz), 84.0 (2C), 72.8 (q, *J* = 31.9 Hz), 24.8 (4C), the quaternary carbon connected to the B atom is too broad and was not detected; **^{19}F NMR** (377 MHz, $CDCl_3$) δ = -78.27 (d, *J* = 6.7 Hz, 3F); **HRMS (ESI)** m/z: $[M+HCOO]^-$ calcd. for $C_{15}H_{19}BF_3O_5$ 347.1283; found 347.1291.

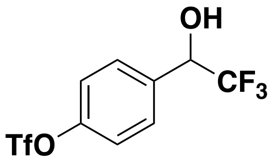

3w. Viscous colorless oil. Reaction time: 7 h. FC eluent: *n*Hex/EtOAc: 7:1. Yield = 61%, (0.061 mmol, 19.8 mg). **^{1}H NMR** (400 MHz, $CDCl_3$) δ = 7.61 – 7.55 (m, 2H), 7.36 – 7.27 (m, 2H), 5.07 (q, *J* = 6.5 Hz, 1H), the OH signal was not detected; **^{13}C NMR** (100 MHz, $CDCl_3$) δ = 150.1, 134.3 (q, *J* = 1.0 Hz), 129.5 (q, *J* = 0.8 Hz, 2C), 123.8 (q, *J* = 282.2 Hz), 121.6 (2C), 118.7 (q, *J* = 320.6 Hz), 71.8 (q, *J* = 32.4 Hz); **^{19}F NMR** (377 MHz, $CDCl_3$) δ = -72.85 (s, 3F), -78.54 (d, *J* = 6.3 Hz, 3F); **HRMS (ESI)** m/z: $[M+HCOO]^-$ calcd. for $C_{10}H_7F_6O_6S$ 368.9873; found 368.9889.

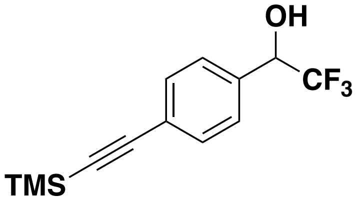

3x. Yellow solid. Reaction time: 16 h. FC eluent: *n*Hex/EtOAc: 8:1. Yield = 65%, (0.065 mmol, 17.7 mg). **MP** = 58 – 60 °C. **^{1}H NMR** (400 MHz, $CDCl_3$) δ = 7.52 – 7.45 (m, 2H), 7.43 – 7.36 (m, 2H), 5.00 (q, *J* = 6.7 Hz, 1H), 2.66 (bs, 1H), 0.24 (s, 9H); **^{13}C NMR** (100 MHz, $CDCl_3$) δ = 133.9 (q, *J* = 1.1 Hz), 132.1 (2C), 127.2 (q, *J* = 0.8 Hz, 2C), 124.5, 124.0 (q, *J* = 282.2 Hz), 104.2, 95.5, 72.5 (q, *J* = 32.1 Hz), -0.14 (3C); **^{19}F NMR** (377 MHz, $CDCl_3$) δ = -78.38 (d, *J* = 6.7 Hz, 3F);). This is a known compound and spectral data are in accordance with the literature.[46]

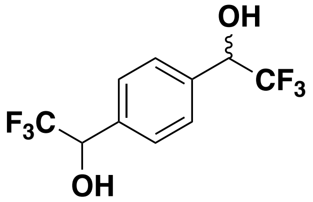

3y. White solid. Reaction time: 16 h. FC eluent: *n*Hex/EtOAc: 8:1. Yield = 73%, (0.073 mmol, 20.0 mg), 1:1 *dr*. **^{1}H NMR** (400 MHz, $CDCl_3$) δ = 7.53 (s, 4H), 5.05 (q, *J* = 6.7 Hz, 2H), 2.65 (bs, 2H), the signals of the two diastereoisomers overlap completely, appearing as a single compound; **^{13}C NMR** (100 MHz, $CDCl_3$) δ = 135.2 (2C), 127.7 (4C), 124.1 (q, *J* = 282.0 Hz, 2C), 72.4 (q, *J* = 32.3 Hz, 2C, two diastereomeric signals), The signals of the two diastereoisomers overlap in some cases, appearing as a single compound, in other cases (as specified in the list) they split; **^{19}F NMR** (377 MHz, $CDCl_3$) δ = -78.38 (d, *J* = 6.5 Hz, 6F), the signals of the two diastereoisomers overlap completely, appearing as a single compound. This is a known compound and spectral data are in accordance with the literature.[47]

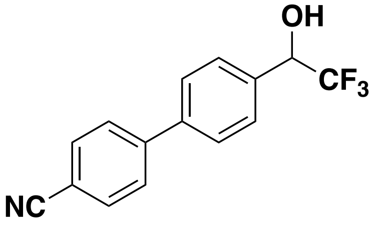

3z. White solid. Reaction time: 16 h. FC eluent: *n*Hex/EtOAc: 5:1. Yield = 58% (0.058 mmol, 16.1 mg). **MP** = 152 – 154 °C. **^{1}H NMR** (400 MHz, $CDCl_3$) δ = 7.75 – 7.70 (m, 2H), 7.69 – 7.65 (m, 2H), 7.64 – 7.56 (m, 4H), 5.09 (q, *J* = 6.7 Hz, 1H), 2.79 (bs,

1H); **^{13}C NMR** (100 MHz, $CDCl_3$) δ = 144.8, 140.3, 134.3, 132.7 (2C), 128.2 (q, *J* = 1.0 Hz, 2C), 127.8 (2C), 127.4 (2C), 124.1 (q, *J* = 282.2 Hz), 118.8, 111.3, 72.4 (q, *J* = 32.1 Hz); **^{19}F NMR** (377 MHz, $CDCl_3$) δ = -78.33 (d, *J* = 6.6 Hz); **HRMS (ESI)** m/z: $[M+HCOO]^-$ calcd. for $C_{16}H_{11}F_3NO_3$ 322.0697; found 322.0700.

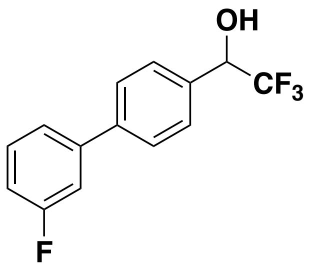

3a'. White solid. Reaction time: 16 h. FC eluent: *n*Hex/EtOAc: 8:1. Yield = 75% (0.075 mmol, 20.3 mg) reaction run on 0.1 mmol scale. Yield = 81% (0.81 mmol, 218.7 mg) reaction run on 1.0 mmol scale (unmodified protocol with the exception of 4.0 mL of DMA employed as the solvent). **MP** = 67 – 70 °C. **^{1}H NMR** (400 MHz, $CDCl_3$) δ = 7.63 – 7.58 (m, 2H), 7.57 – 7.52 (m, 2H), 7.44 – 7.33 (m, 2H), 7.27 (ddd, *J* = 10.2, 2.5, 1.6 Hz, 1H), 7.09 – 7.02 (m, 1H), 5.07 (q, *J* = 6.7 Hz, 1H), 2.75 (s, 1H); **^{13}C NMR** (100 MHz, $CDCl_3$) δ = 163.2 (d, *J* = 246.0 Hz), 142.6 (d, *J* = 7.6 Hz), 141.2 (d, *J* = 2.3 Hz), 133.4 (q, *J* = 1.1 Hz), 130.3 (d, *J* = 8.5 Hz), 128.0 (q, *J* = 0.9 Hz, 2C), 127.3 (2C), 124.2 (q, *J* = 281.9 Hz), 122.8 (d, *J* = 3.0 Hz), 114.5 (d, *J* = 21.2 Hz), 114.0 (d, *J* = 22.1 Hz), 72.5 (q, *J* = 32.1 Hz); **^{19}F NMR** (377 MHz, $CDCl_3$) δ = -78.32 (d, *J* = 6.7 Hz), -112.89 (ddd, *J* = 10.0, 8.3, 5.5 Hz). This is a known compound and spectral data are in accordance with the literature.[48]

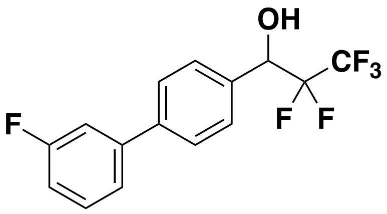

3b'. White solid. Reaction time: 16 h. FC eluent: *n*Hex/EtOAc: 10:1. Yield = 22% (0.022 mmol, 7.0 mg). **MP** = 65 – 68 °C. **^{1}H NMR** (400 MHz, $CDCl_3$) δ = 7.66 – 7.60 (m, 2H), 7.59 – 7.52 (m, 2H), 7.45 – 7.36 (m, 2H), 7.30 (dt, *J* = 10.1, 2.1 Hz, 1H), 7.10 – 7.02 (m, 1H), 5.19 (ddd, *J* = 16.6, 7.2, 4.9 Hz, 1H), 2.56 (d, *J* = 5.0 Hz, 1H); **^{13}C NMR** (100 MHz, $CDCl_3$) δ = 163.2 (d, *J* = 246.0 Hz), 142.5 (d, *J* = 7.6 Hz), 141.3 (d, *J* = 2.1 Hz), 133.4, 130.3 (d, *J* = 8.4 Hz), 128.4 (2C), 127.3 (2C), 122.8 (d, *J* = 3.0 Hz), 114.5 (d, *J* = 21.3 Hz), 114.1 (d, *J* = 22.1 Hz), 71.7 (dd, *J* = 28.1, 22.6 Hz), the CF_2 and CF_3 carbons of the pentafluoroethyl group were not detected; **^{19}F NMR** (377 MHz, $CDCl_3$) δ = -81.20 (s, 3F), -112.87 (td, *J* = 9.3, 5.6 Hz, 1F), -121.66 (dd, *J* = 275.9, 7.3 Hz, 1F), -129.27 (dd, *J* = 276.0, 16.6 Hz, 1F); **HRMS (ESI)** m/z: $[M+HCOO]^-$ calcd. for $C_{16}H_{11}F_6O_3$ 365.0618; found 365.0625.

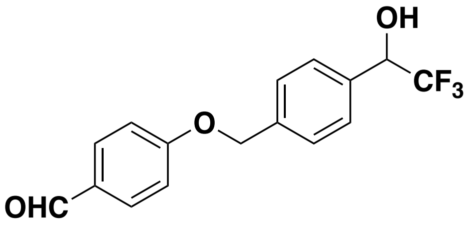

3c'. White solid. Reaction time: 16 h. FC eluent: *n*Hex/EtOAc: 3:1. Yield = 50% (0.050 mmol, 15.5 mg). **MP** = 114 – 117 °C. **^{1}H NMR** (400 MHz, $CDCl_3$) δ = 9.86 (s, 1H), 7.86 – 7.78 (m, 2H), 7.53 – 7.49 (m, 2H), 7.48 – 7.44 (m, 2H), 7.10 – 7.02 (m, 2H), 5.16 (s, 2H), 5.04 (q, *J* = 6.8 Hz, 1H), 2.90 (s, 1H); **^{13}C NMR** (100 MHz, $CDCl_3$) δ = 190.9, 163.5, 137.4, 134.1, 132.0 (2C), 130.2, 127.8 (2C), 127.5 (2C), 124.2 (q, *J* = 282.3 Hz), 115.1 (2C), 72.5 (q, *J* = 31.9 Hz), 69.7; **^{19}F NMR** (377 MHz, $CDCl_3$) δ = -78.4 (d, *J* = 6.7 Hz); **HRMS (ESI)** m/z: $[M+HCOO]^-$ calcd. for $C_{17}H_{14}F_3O_5$ 355.0799; found 355.0788.

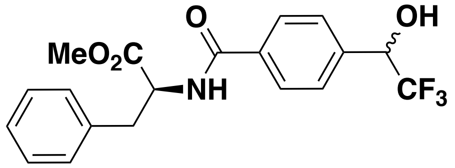

7a. White solid. Reaction time: 16 h. FC eluent: *n*Hex/EtOAc: 3:1. Yield = 65%, (0.065 mmol, 24.8 mg), 1:1 *dr*. **^{1}H NMR** (400 MHz, $CDCl_3$) δ = 7.70 – 7.63 (m, 2H), 7.50 – 7.45 (m, 2H), 7.33 – 7.20 (m, 3H), 7.14 – 7.07 (m, 2H), 6.55 (d, *J* = 7.6 Hz, 1H), 5.09 – 4.98 (m, 2H), 3.76 (s, 3H), 3.32 (bs, 1H) partially overlapped with 3.28 (dd, *J* = 13.9, 5.8 Hz, 1H), 3.20 (dd, *J* = 13.9, 5.6 Hz, 1H), the signals of the two diastereoisomers overlap completely, appearing as a single compound; **^{13}C NMR** (100 MHz, $CDCl_3$) δ = 172.0 (two diastereomeric signals), 166.4 (two diastereomeric signals), 137.8, 135.6, 134.7 (two diastereomeric signals), 129.3 (2C), 128.7 (2C), 127.7 (2C), 127.3, 127.1 (2C, two diastereomeric signals), 124.1 (q, *J* = 282.3 Hz), 72.10 (q, *J* = 31.8 Hz, two diastereomeric signals) 53.6 (two diastereomeric signals), 52.5, 37.8, the signals of the two diastereoisomers overlap in some cases, appearing as a single compound, in other cases (as specified in the list) they split; **^{19}F NMR** (377 MHz, $CDCl_3$) δ = -78.25 (d, *J* = 6.8 Hz, 3F), and -78.26 (d, *J* = 6.8 Hz, 3F), two partially overlapped diastereomeric signals; **HRMS (ESI)** m/z: $[M+Na]^+$ calcd. for $C_{19}H_{18}F_3NNaO_4$ 404.1086; found 404.1079.

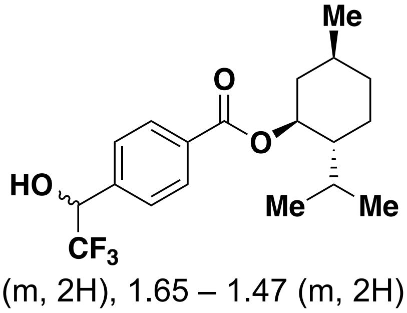

7b. White solid. Reaction time: 16 h. FC eluent: *n*Hex/EtOAc: 5:1. Yield = 46%, (0.046 mmol, 16.5 mg), 1:1 *dr*. **^{1}H NMR** (400 MHz, $CDCl_3$) δ = 8.09 – 8.02 (m, 2H), 7.58 – 7.51 (m, 2H), 5.08 (q, *J* = 6.6 Hz, 1H), 4.92 (td, *J* = 10.9, 4.4 Hz, 1H), 2.84 (bs, 1H), 2.15 – 2.05 (m, 1H), 1.98 – 1.85 (m, 1H), 1.77 – 1.67 (m, 2H), 1.65 – 1.47 (m, 2H), 1.18 – 1.02 (m, 2H), 0.92 (d, *J* = 4.8 Hz, 3H), 0.90 (d, *J* = 5.4 Hz, 3H) overlapped with 0.93 – 0.88 (m, 1H), 0.77 (d, *J* = 6.9 Hz, 3H), the signals of the two

diastereoisomers overlap completely, appearing as a single compound; **^{13}C NMR** (100 MHz, $CDCl_3$) δ = 165.6, 138.3, 131.9, 129.7 (2C), 127.4 (2C), 124.0 (q, *J* = 282.2 Hz), 75.2, 72.4 (q, *J* = 32.3, Hz) and 72.3 (q, *J* = 31.8 Hz, two diastereomeric signals), 47.2, 40.9, 34.3, 31.4, 26.5 (two diastereomeric signals), 23.6 (two diastereomeric signals), 22.0, 20.7 (two diastereomeric signals), 16.5 (two diastereomeric signals). The signals of the two diastereoisomers overlap in some cases, appearing as a single compound, in other cases (as specified in the list) they split; **^{19}F NMR** (377 MHz, $CDCl_3$) δ = -78.25 (d, *J* = 6.5 Hz, 3F) and -78.26 (d, *J* = 6.4 Hz, 3F), two partially overlapped diastereomeric signals; **HRMS (ESI)** m/z: $[M+HCOO]^-$ calcd. for $C_{20}H_{26}F_3O_5$ 403.1738; found 403.1741.

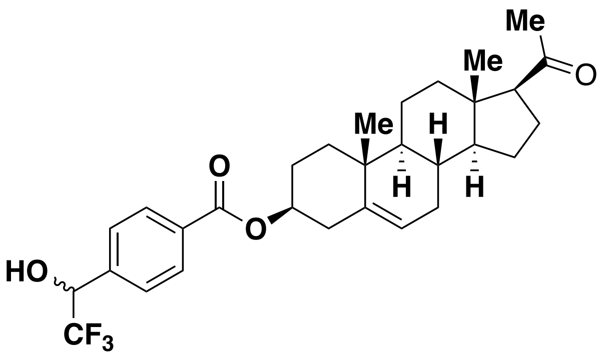

7c. White solid. Reaction time: 16 h. FC eluent: *n*Hex/*i*PrOAc: 2:1. Yield = 32%, (0.032 mmol, 16.6 mg), 1:1 *dr*. **^{1}H NMR** (400 MHz, $CDCl_3$) δ = 8.09 – 8.03 (m, 2H), 7.60 – 7.50 (m, 2H), 5.44 – 5.37 (m, 1H), 5.09 (q, *J* = 6.6 Hz, 1H), 4.85 (dtd, *J* = 16.3, 8.4, 4.5 Hz, 1H), 2.98 (s, 1H), 2.53 (t, *J* = 8.9 Hz, 1H), 2.45 (d, *J* = 7.2 Hz, 2H), 2.21 – 2.13 (m, 1H), 2.11 (s, 3H), 2.07 – 1.96 (m, 3H), 1.91 (dt, *J* = 13.4, 3.5 Hz, 1H), 1.80 – 1.39 (m, 7H), 1.31 – 1.13 (m, 4H), 1.05 (s, 3H) overlapped with 1.07 – 1.01 (m, 1H), 0.62 (s, 3H), the signals of the two diastereoisomers overlap completely, appearing as a single compound; **^{13}C NMR** (100 MHz, $CDCl_3$) δ = 209.8, 165.4, 139.5, 138.4, 131.8, 129.7 (2C), 127.4 (2C), 124.0 (q, *J* = 282.6 Hz), 122.6, 74.7, 72.4 (q, *J* = 32.1 Hz), 63.7, 56.8, 49.9, 44.0, 38.8, 38.1, 37.0, 36.6, 31.8, 31.8, 31.5, 27.8, 24.5, 22.8, 21.0, 19.3, 13.2, the signals of the two diastereoisomers overlap completely, appearing as a single compound; **^{19}F NMR** (377 MHz, $CDCl_3$) δ = -78.25 (d, *J* = 6.3 Hz, 3F), the signals of the two diastereoisomers overlap completely, appearing as a single compound; **HRMS (ESI)** m/z: $[M+HCOO]^-$ calcd. for $C_{31}H_{38}F_3O_6$ 563.2626; found 563.2633.

6. Computational mechanistic studies

To gain insights into the reaction mechanism behind the presented transformation we resorted to molecular modelling. We envisioned that the first step of this mechanism would involve participation of Zn(0) as a reducing agent ($\xi_{Zn(0)\text{-}Zn(II)}$ = 0.76 V). Specifically, we expected that Zn(0) would be responsible for the reduction of the pre-catalyst **I**. In principle, this reduction can render a Ni(0) species **XIV** or a Ni(I) species **III** as a matter of whether a total of two or one electrons are transferred from Zn(0) to the [Ni (II)] complex **I** (**Figure S2**). Starting with the double reduction of **I** towards **XIV**, we have found that this reduction is endergonic by 3.67 kcal/mol. Once **XIV** is formed it can be stabilized via solvation (**XV**) and/or evolve through the coordination of **II**. Then, the resulting Ni(0) complex (**XVI**) can progress via an oxidative addition involving the insertion of the metal in the C*sp*^{2}-I bond resulting in the formation of the Ni(II) complex **VI** (-57.35 kcal/mol). Alternatively, **XIV** can evolve through a SET process in which the Ni(0) complex releases an electron to **II** rendering the pair **XVIII/XIX**. **XIX** can subsequently reorganize towards **VI** in a process that involves two oxidative steps: the first one, consisting of an iodine abstraction at **XVIII** by **XIX,** forming **XXI** and **XXII** (-22.07 kcal/mol). The second oxidative step involves the collapse of the resulting radical **XXII** onto the Ni(I) complex, rendering **XVII**.

*These two paths can, in principle, operate under the reported reaction conditions. They involve an energy span of 5.11 kcal/mol (evolution of **XVI** to **VI**) and 3.67 kcal/mol (evolution of **I** to **XIV**), respectively.*

Alternatively, **I** can evolve via a monoelectronic reduction towards the formation **III** (**Figure S2**, top). We have found that against the direct reduction of Ni(II) to Ni(0), the reduction of Ni(II) to Ni(I) is exergonic by -15.16 kcal/mol. Hence, the formation of species **III** in the reaction process is more likely than the formation of **XIV**. Once **III** is formed it can coordinate **II** and subsequently engage in an intramolecular oxidative addition towards forming species **V** (-18.31 kcal/mol). **V** can then react with a second molecule of Zn(0) resulting in **XXIII** (-63.90 kcal/mol).

Overall, this latter path involving a Ni(II)-Ni(I)-Ni(III)-Ni(I) triple redox process involves an energy span of 3.58 kcal/mol (*corresponding to the conversion of **IV** to **V***) resulting competitive with the SET path. However, since the formation of **II** is endergonic and not so the formation of **XV**, together with the fact that we are in a large excess of iodine ions* we consider the triple redox path as the one operating.

**** Please note the beneficial effect of adding NaI described in the main text, we hypothesize that the addition of this counterion promotes this path over the radical counterpart hence preventing the initiation of other undesired radical mechanisms that could erode the yield of the reaction.***

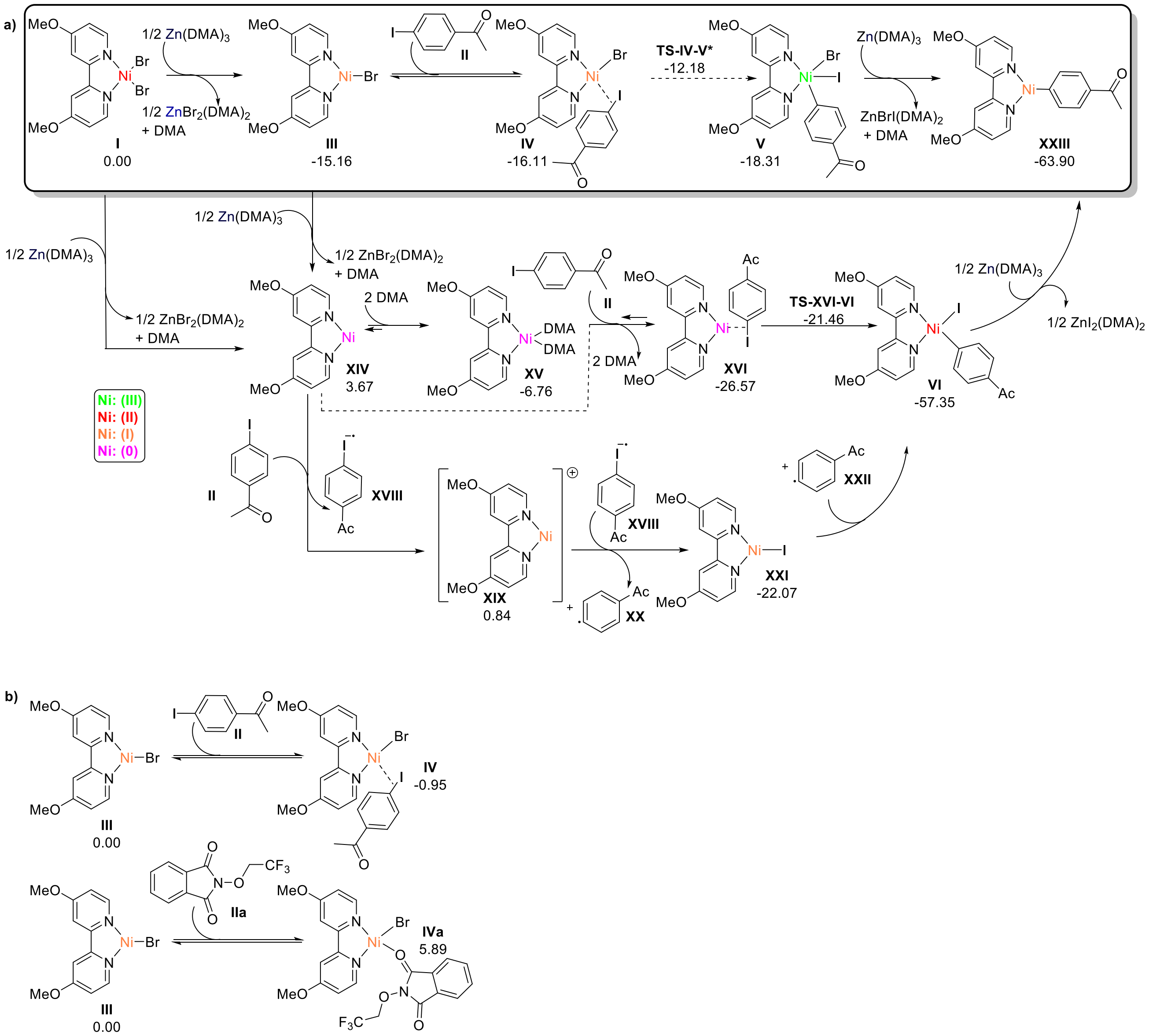

Figure S2. Explored paths for a) the reduction of Ni(II) to Ni(I) mediated by Zn(0) and b) potential equilibrium present in solution. With a star (*) we have marked those barriers estimated via application of the NEB method (see below).

Table S6. Energy data of those stationary points explored in **Figure S2.**[a]

ID	ImFreqs	Stable	SCF	SCF+ZPVE	H[b]	G[c]
**I**	-	Yes	-7379.142121	-7378.915506	-7378.895061	-7378.967796
**Zn(DMA)$_3$**	-	Yes	-2641.356003	-2640.965713	-2640.937012	-2641.026676

**III**	-	Yes	-4805.330318	-4805.105207	-4805.086799	-4805.153919
**II**	-	Yes	-681.484264	-681.357664	-681.347327	-681.394457
**IV**	-	Yes	-5486.839878	-5486.487277	-5486.458019	-5486.549899
**V**	-	Yes	-5486.842713	-5486.489363	-5486.459967	-5486.553394
**ZnBrI(DMA)$_3$**	-	Yes	-5513.041949	-5512.647318	-5512.615385	-5512.71261
**XXIII**	-	Yes	-2615.232346	-2614.882141	-2614.856593	-2614.940116
**XIV**	-	Yes	-2231.461627	-2231.241024	-2231.224297	-2231.285883
**DMA**	-	Yes	-287.4093664	-287.280614	-287.272025	-287.312298
**XV**	-	Yes	-2806.342546	-2805.860734	-2805.826508	-2805.9271
**XVI**	-	Yes	-2913.020057	-2912.66912	-2912.642069	-2912.728527
**TS-XVI-VI**	-90.87	Yes	-2914.5002434	-2912.662598	-2912.635958	-2912.721789
**VI**	-	Yes	-2913.070676	-2912.717788	-2912.69069	-2912.77759
**XVIII**	-	Yes	-756.723557	-756.595153	-756.582620	-756.639416
**XIX**	-	Yes	-2231.365831	-2231.126133	-2231.125189	-2231.185783
**XX**	-	Yes	-458.787541	-458.650230	-458.649286	-458.695118
**XXI**	-	Yes	-2529.341105	-2529.098591	-2529.097647	-2529.166601
**IIa**	-	Yes	-963.385476	-963.231759	-963.217049	-963.274797
**IVa**	-	Yes	-5768.73091	-5768.351523	-5768.318054	-5768.419329

[a] SCF energies correspond to the electronic energies expressed in a.u. Imaginary frequencies are expressed in cm^{-1}. [b] H denotes the Sum of electronic and thermal Enthalpies. [c] G denotes the Sum of electronic and thermal Free Energies.

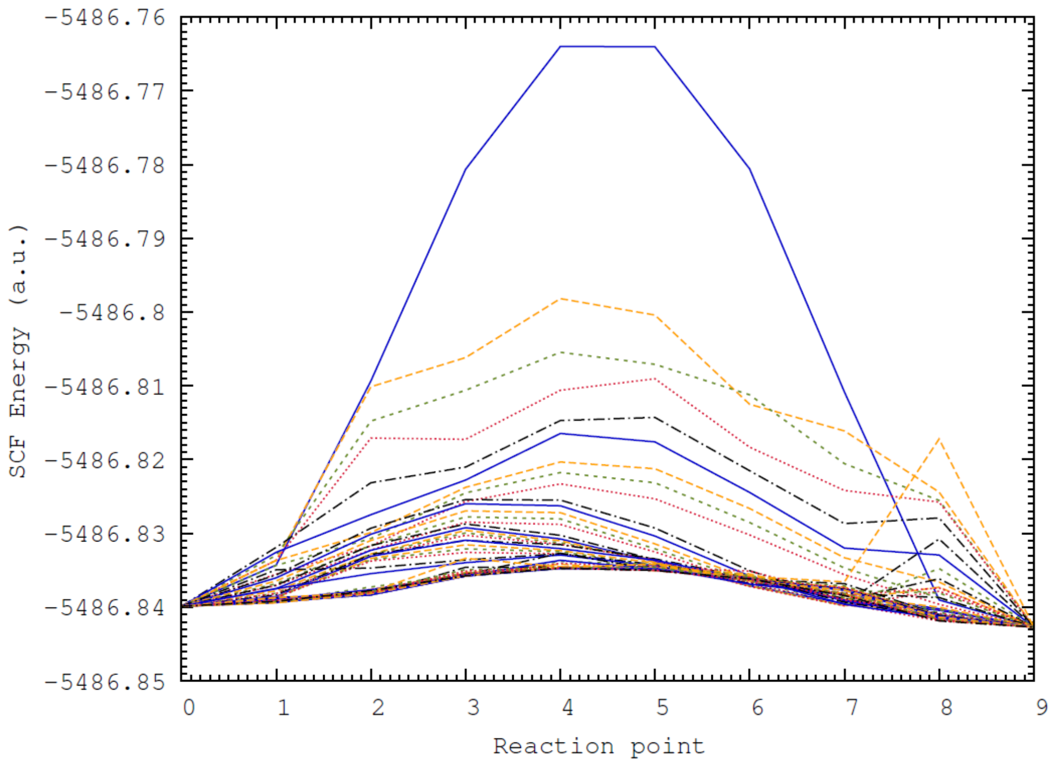

Figure S3. Exploration of the energy profile for the conversion of **IV** to **V** using the NEB. Y-axis represents the SCF energies expressed in a.u. and X-axis represents the reaction points.

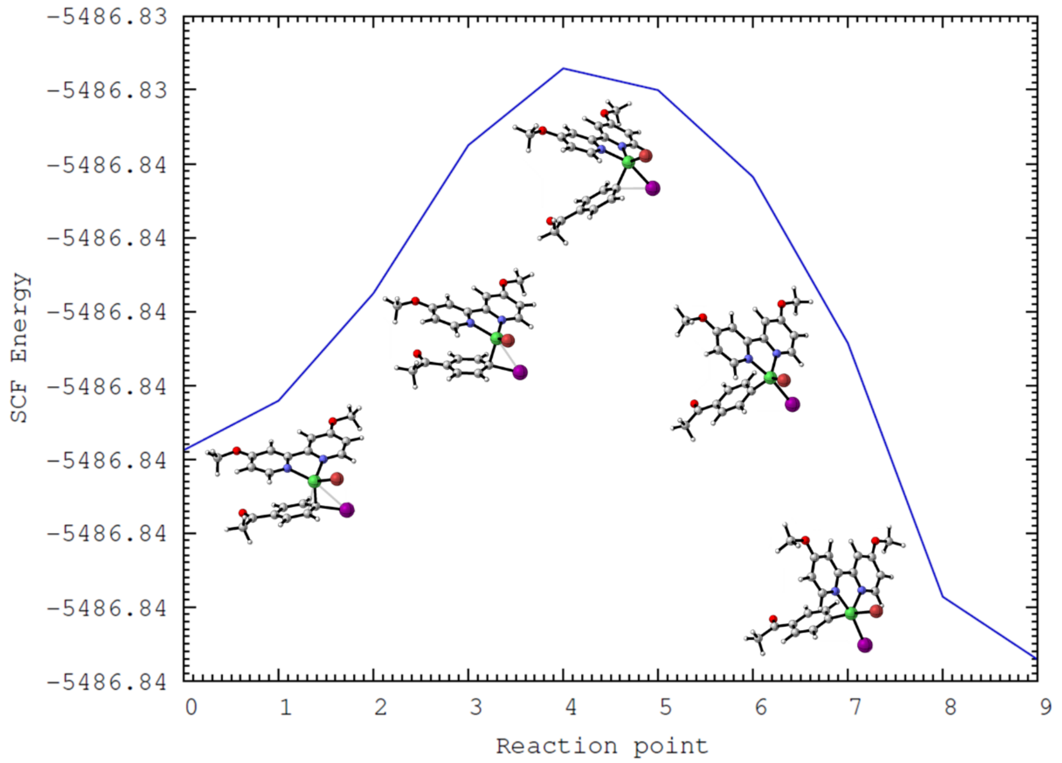

Figure S3a. MEP for the for the conversion of **IV** to **V** obtained using the NEB. Y-axis contains the SCF energies expressed in a.u. and X-axis represents the reaction points.

With this mechanistic picture, we wondered how the second component of the product, *i.e.* the alkoxy-radical, could be formed in solution. We hypothesized that the very Zn(II) released in the previous steps could be responsible for coordinating **2** and activating it to evolve via a SET in the presence of a second unit of Zn(0) and subsequently release the alkoxy-radical **VII**. Alternatively, we also acknowledge that the very Ni(I) complexes formed in previous reaction steps could account for an analogous intramolecular reductive chemistry. Hence, we embarked into exploring all these potential paths.

Starting with the Zn promoted transformation (**Figure S4a**), we have found that the initial coordination of $ZnBr_2$ to **2** is endergonic by 3.86 kcal/mol and its subsequent evolution via SET promoted by Zn(0) is also endergonic by 9.61 kcal/mol. However, the resulting complex **X** can easily reorganize towards **XI** and later evolve towards **XII** (**Figure S4a**). This overall transformation accounts for the release of an alkoxy molecule to the reaction medium. Then, **VII** can easily tautomerize to **VIII** in the presence of DMA (**Figure S4b**). All our attempts to find the direct release of the alkoxy-radical at these steps failed and resulted in its coordination to the metallic center. At this stage, we hypothesized that in the presence of such a coordinating solvent as DMA, perhaps the solvent could impede the coordination of the alkoxy radical and aid in its release. We have found that while solvent does prevent coordination of the alkoxy-radical to the Zn center it also imposes a high energy penalization for the process.

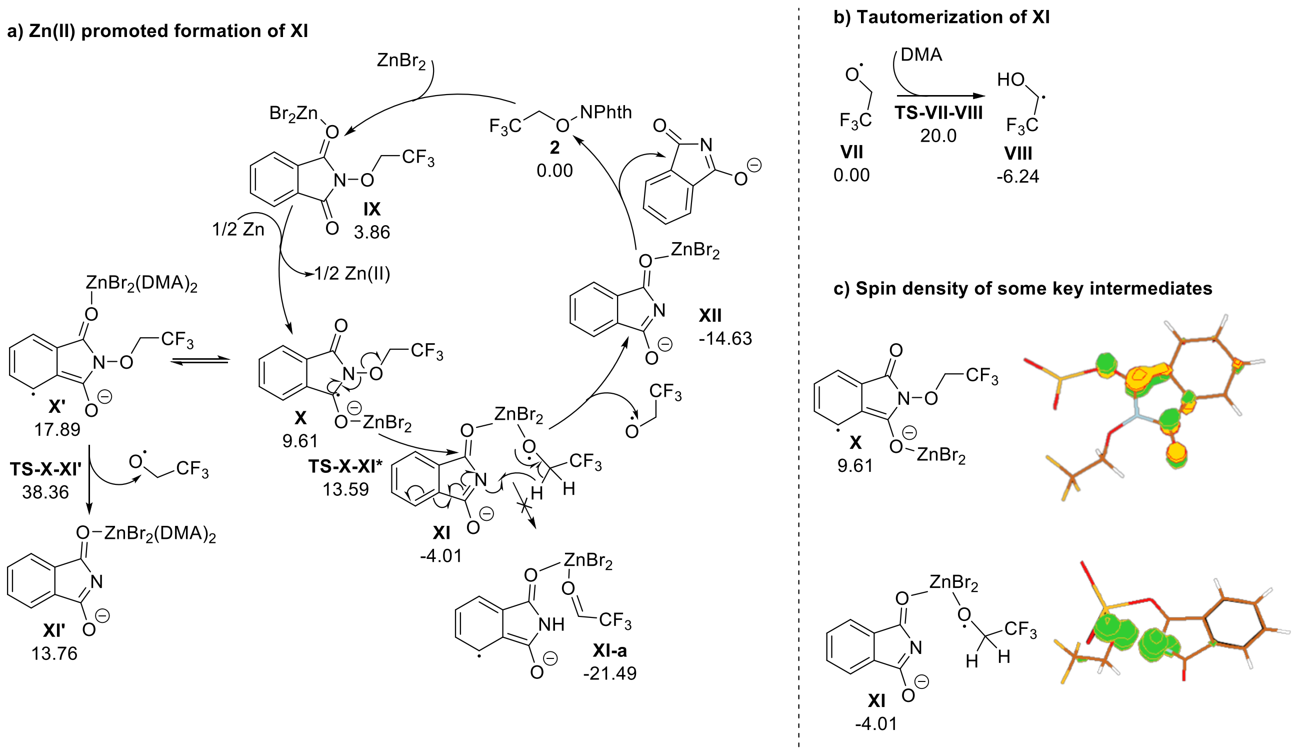

Figure S4. a) Zn promoted formation of an alkoxy-radical from **2**. b) Solvent promoted tautomerization of **VII**. c) Spin density obtained for some key intermediates. *The energy of this transition state was estimated via the use of the NEB method.

Table S7. Energy data of those stationary points explored in **Figure S4**.[a]

ID	ImFreqs	Stable	SCF	SCF+ZPVE	H[b]	G[c]
**2**	-	Yes	-964.489	-963.232	-963.217	-963.275
**IX**	-	Yes	-7892.14	-7889.98	-7889.96	-7890.03
**X**	-	Yes	-8465	-8464.58	-8464.54	-8464.65
**XI**	-	Yes	-7892.28	-7890.13	-7890.11	-7890.19
**XII**	-	Yes	-7440.17	-7438.59	-7438.58	-7438.64
**X’**	-	Yes	-8465.121	-8464.704	-8464.666	-8464.7767
**TS-X-XI’**	-1292.20	Yes	-8465.088	-8464.6732	-8464.635	-8464.7440
**XI’**	-	Yes	-8465.123	-8464.708	-8464.669	-8464.7832
**VII**	-	Yes	-452.115	-451.534	-451.528	-451.564
**TS-VII-VIII**	-437.56	Yes	-739.866	-738.802	-738.787	-738.845
**VIII**	-	Yes	-739.902	-738.843	-738.828	-738.887

[a] SCF energies correspond to the electronic energies expressed in a.u.. Imaginary frequencies are expressed in cm^{-1}. [b] H denotes the Sum of electronic and thermal Enthalpies. [c] G denotes the Sum of electronic and thermal Free Energies.

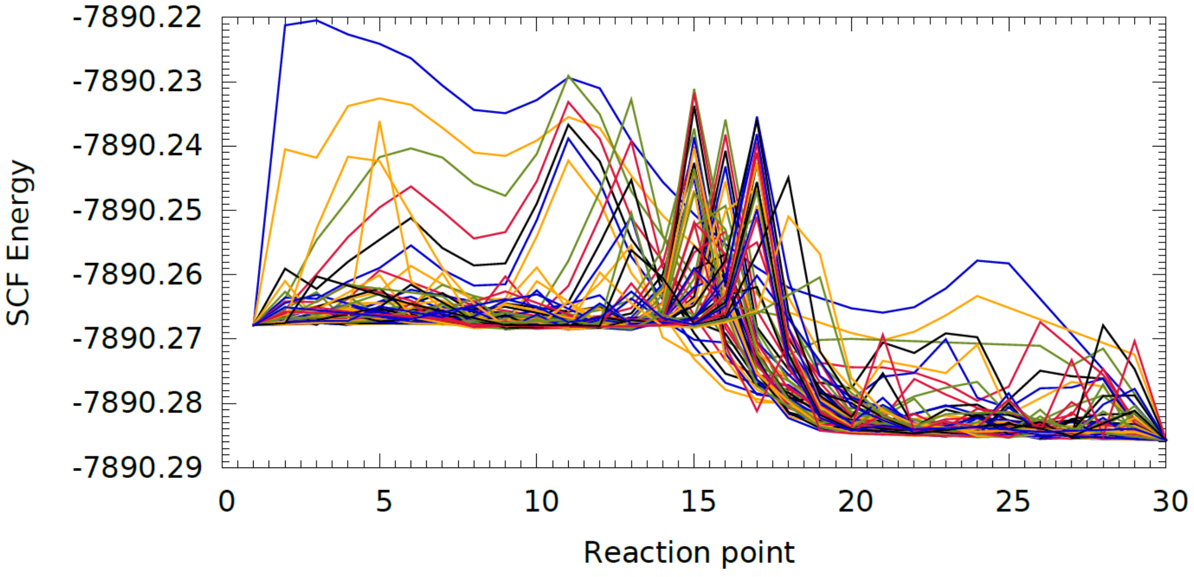

Figure S5. PES evaluation for the transformation of **X** to **XI** using the NEB method.

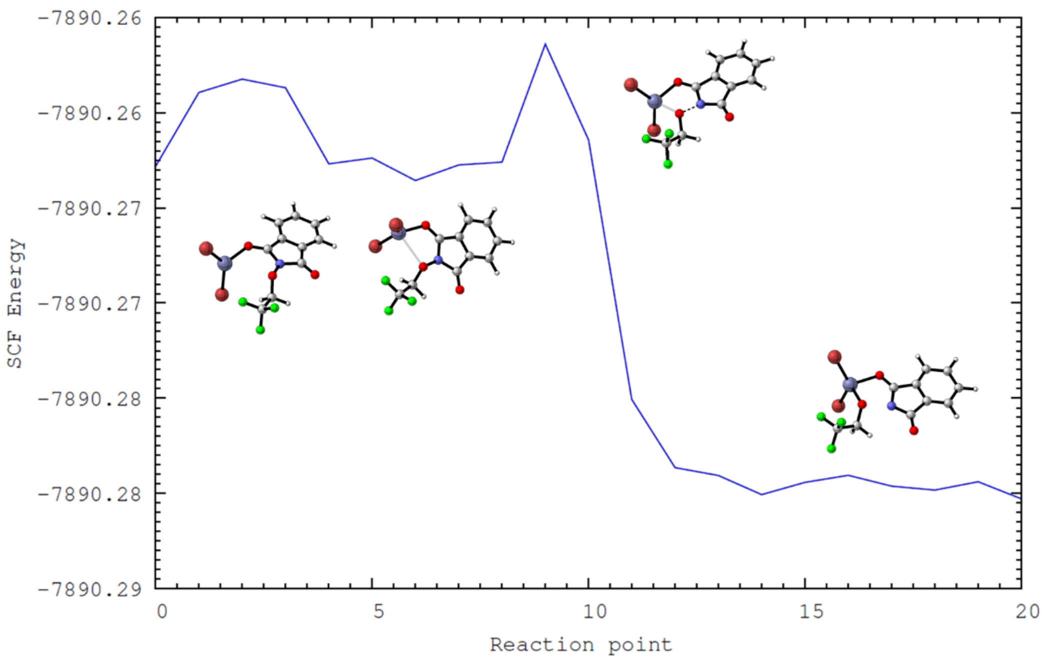

Figure S5a. MEP evaluation for the transformation of **X** to **XI**. This MEP is obtained with the NEB method.

Once **VIII** is formed it can collapse on different complexes already described, namely **XXIII** and **VI**.

Starting with the reactivity of **VIII** with **XXIII**, the interaction between these two fragments renders complex **XXIV** in a step that is exergonic by 36.15 kcal/mol. Then, **XXIV** can evolve via a reductive elimination and render the product **P** and a Ni(0) species (**Figure S6a**). This

Ni(0) species can alternatively engage in an oxidative addition with a second **III** molecule forming ultimately **XVII** that after reduction in the presence of Zn allows the recovery of the active catalyst (**Figure S2**).
Following with the collapse of **VIII** on **VI**, we found that the radical collapse on this Ni(II) complex is exergonic by 13.99 kcal/mol, and therefore less favorable than the previously described path. Were complex **XXV** formed it could also evolve towards **3e** via an easy reductive elimination (**Figure S6b**).

a) Collapse of the alcoxy-radical on XXIII

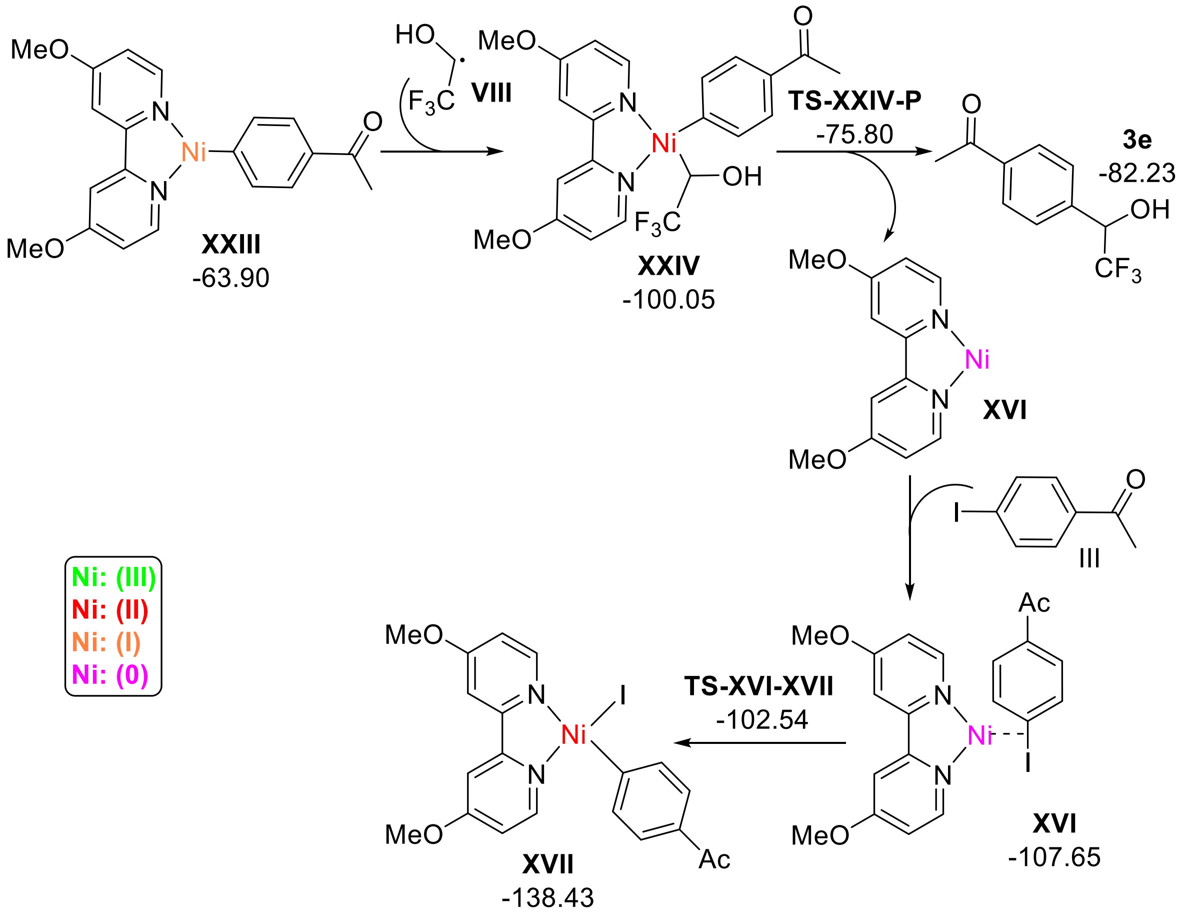

b) Collapse of the alcoxy-radical onVI

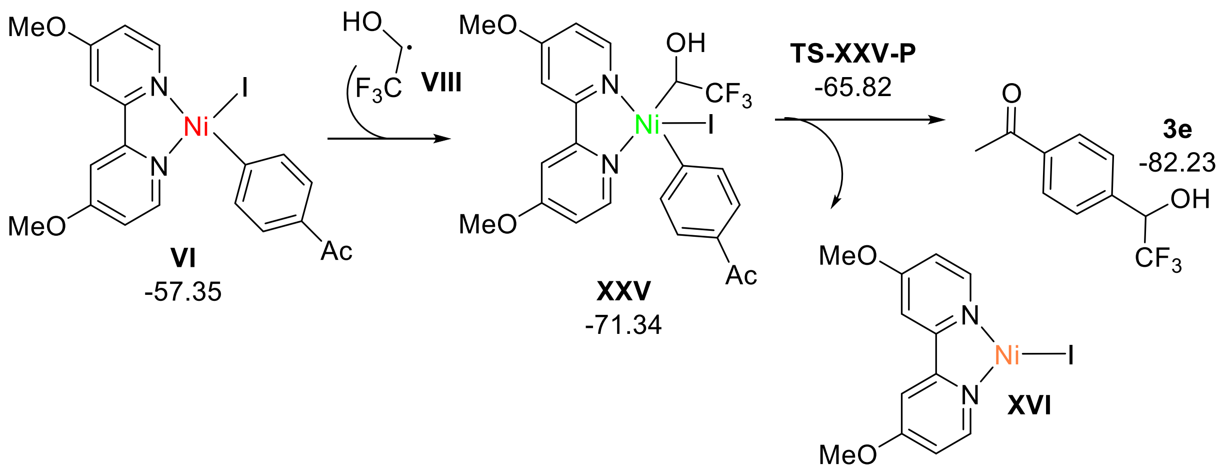

Figure S6. Study of the evolution of the complexes a) **XIII-2D** and b) **XVIII-1S** after collapse of the alkoxy-radical towards **3e**.

Table S8. Energy data of those stationary points explored in **Figure S6**.[a]

ID	ImFreqs	Stable	SCF	SCF+ZPVE	H[b]	G[c]
**XXIII**	-	Yes	-2615.232346	-2614.882141	-2614.856593	-2614.940116
**VIII**	-	Yes	-739.017868	-738.843239	-738.827689	-738.886553

**XXIV**	-	Yes	-3066.907832	-3066.508319	-3066.476921	-3066.571985
**TS-XXIV-P**	-412.282	Yes	-3066.866966	-3066.470081	-3066.43892	-3066.533343
**P(3e)**	-	Yes	-835.3916499	-835.216291	-835.201694	-835.257705
**XVI**	-	Yes	-2231.461627	-2231.241024	-2231.224297	-2231.285883
**III**	-	Yes	-681.484264	-681.357664	-681.347327	-681.394457
**XVI**	-	Yes	-2913.020057	-2912.66912	-2912.642069	-2912.728527
**TS-XVI-XVII**	-90.8689	Yes	-2913.013395	-2912.662598	-2912.635958	-2912.721789
**XVII**	-	Yes	-2913.070676	-2912.717788	-2912.69069	-2912.77759
**XXV**	-	Yes	-3364.696455	-3364.296235	-3364.26261	-3364.364129
**TS-XXV-P**	-256.665	Yes	-3364.695962	-3364.296883	-3364.263407	-3364.365343

[a] SCF energies correspond to the electronic energies expressed in a.u.. Imaginary frequencies are expressed in cm^{-1}. [b] H denotes the Sum of electronic and thermal Enthalpies.[c] G denotes the Sum of electronic and thermal Free Energies.

Alternatively, the formation of the alkoxy radical can be mediated by any of the Ni complexes formed in the reaction media that feature a coordination vacancy, namely **XXIII** and **III**. We have already pointed that the coordination of **2** is endergonic and therefore unlikely to be competitive, but we have found that the coordination of **2** to **XXIII** is exergonic and can facilitate the release of this radical (**Figure S7**). This path can continue evolving towards obtaining the product P, however the energy penalization associated with it is too high to be competitive at the working conditions.

Potential path towards forming XI and P

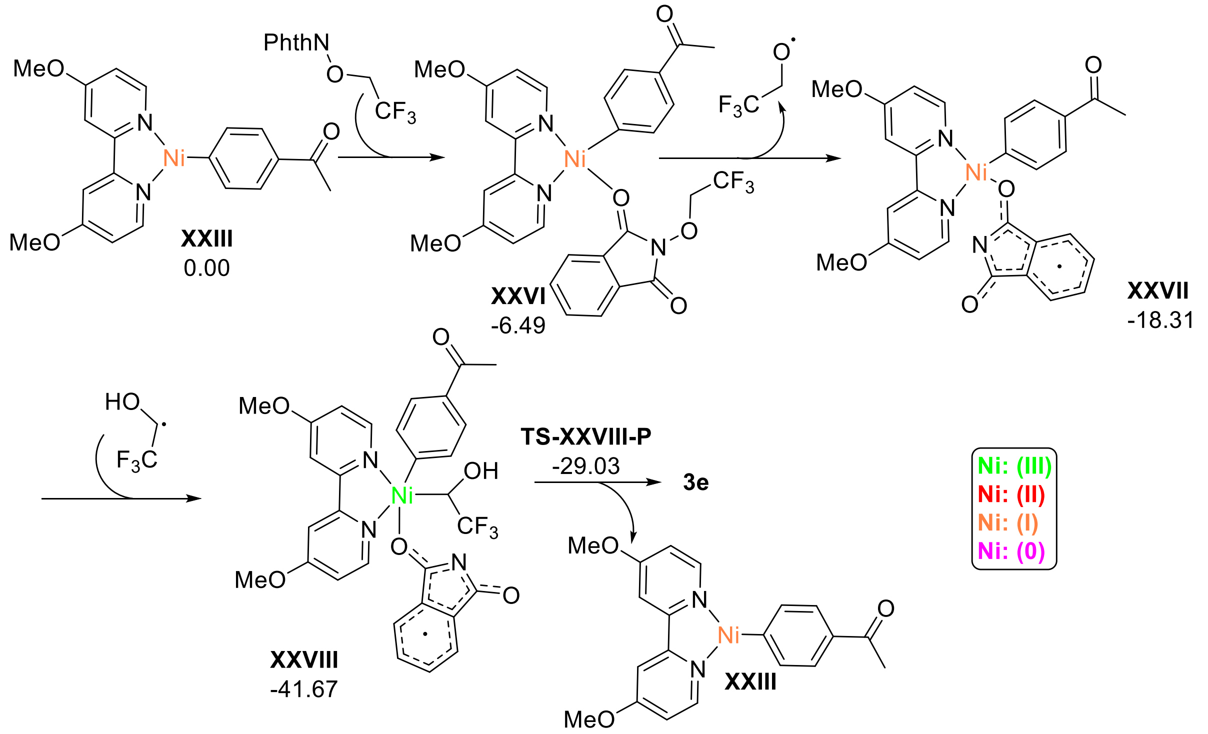

Figure S7. Alternative paths towards the formation of **3e**.

Table S9. Energy data of those stationary points explored in **Figure S7**.[a]

ID	ImFreqs	Stable	SCF	SCF+ZPVE	H[b]	G[c]
**XXVI**	-	Yes	-3578.66	-3578.15	-3578.11	-3578.23
**XXVII**	-	Yes	-3127.07	-3126.61	-3126.57	-3126.68
**XXVIII**	-	Yes	-3578.71	-3578.2	-3578.16	-3578.28
**TS-VIII-P**	-230.03	Yes	-3578.69	-3578.19	-3578.15	-3578.26

[a] SCF energies correspond to the electronic energies expressed in a.u.. Imaginary frequencies are expressed in cm^{-1}. [b] H denotes the Sum of electronic and thermal Enthalpies. [c] G denotes the Sum of electronic and thermal Free Energies.

At this point, we also evaluated the possibility of **XI** evolving towards **XIa**, we have found that this formal 1,2 migration is energetically quite demanding.

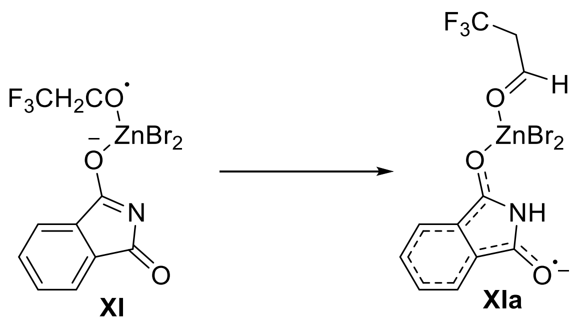

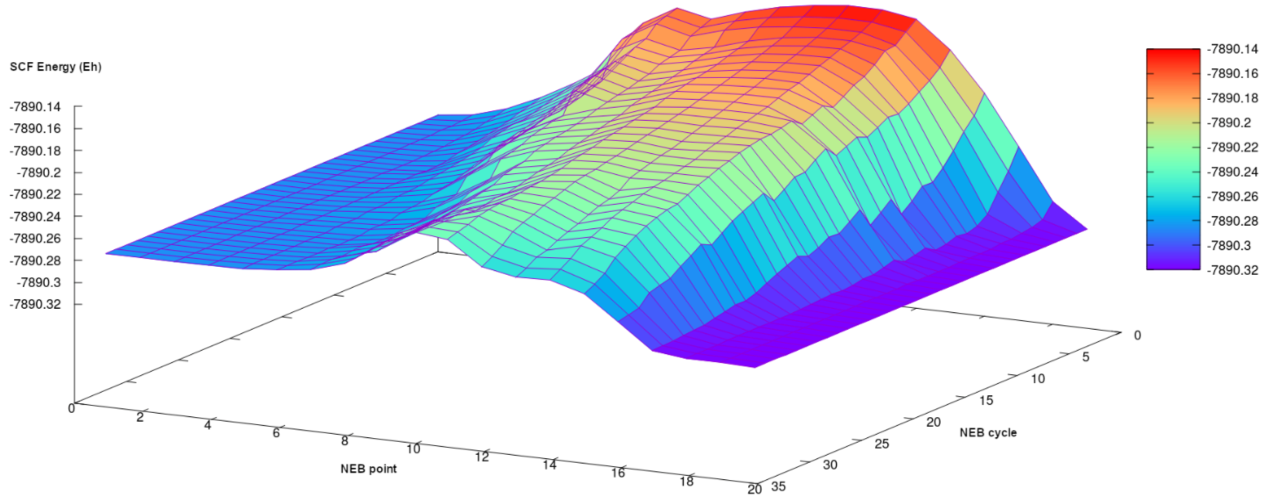

Figure S8. 3D representation of the explored reacting surface related to the H-shift step from **XI** to **Xia** via the NEB method. Arbitrary units used in the configuration space and Hartrees (Eh) used in the energy axis.

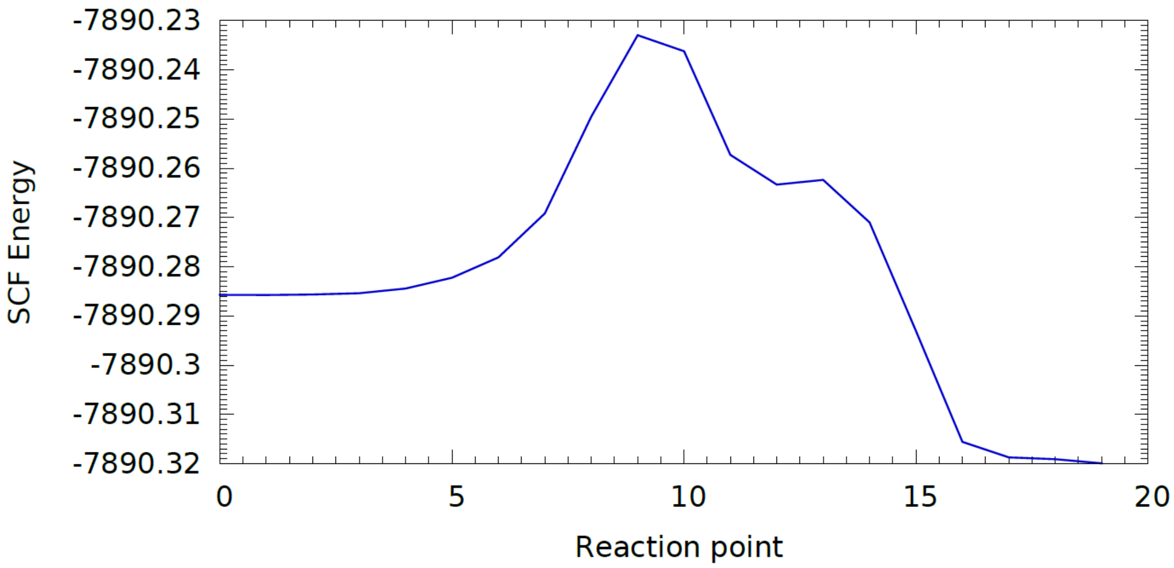

Figure S8a. MEP for the for the conversion of **XI** to **XIa** obtained using the NEB. Y-axis contains the SCF energies expressed in a.u. and X-axis represents the reaction points.

7. Radical trapping experiments

7.1 Trapping with tert-butylacrylate **4a**

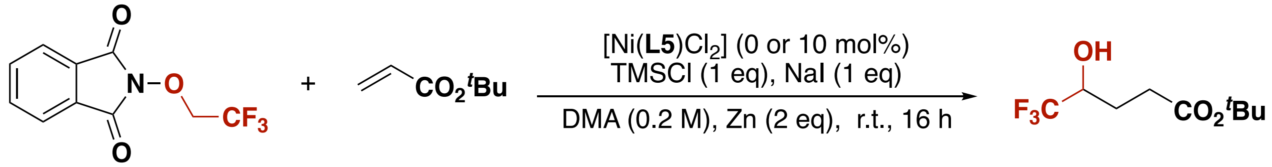

Radical trapping experiment with *tert*-butylacrylate in the presence of Ni catalyst was set up according to General procedure (see section 5.1), substituting aryl iodide **1** with *tert*-butylacrylate **4a** (1 eq). Adduct **5a** was isolated in 35% yield after work-up and flash chromatography as a colorless oil.

The same reaction was set up omitting the Ni catalyst and adduct **5a** was isolated in 12% yield. This confirms the presence of the C-centered radical and the fact that Ni is not necessary for the activation of N-trifluoroethoxyphthalimide nor for the isomerization of the O-centered radical.

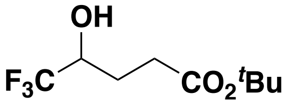

5a. Colourless oil. FC eluent: *n*Hex/EtOAc: 6:1. Yield = 35% (0.035 mmol, 8.0 mg). **^{1}H NMR** (400 MHz, $CDCl_3$) δ = 4.04 – 3.92 (m, 1H), 3.26 (d, *J* = 6.0 Hz, 1H), 2.59 – 2.39 (m, 2H), 2.05 – 1.94 (m, 1H), 1.94 – 1.83 (m, 1H), 1.44 (s, 9H); **^{13}C NMR** (100 MHz, $CDCl_3$) δ = 173.3, 125.0 (q, *J* = 281.9 Hz), 81.5, 70.0 (q, *J* = 31.1 Hz), 31.0, 28.0, 24.6 (q, *J* = 2.0 Hz); **^{19}F NMR** (377 MHz, $CDCl_3$) δ = -80.06 (d, *J* = 6.7 Hz); **HRMS (ESI)** m/z: [M+HCOO]- calcd. for $C_{10}H_{16}F_3O_5$ 273.0955; found 273.0960.

Additional notes:

1. Compound **5a** is fairly volatile, so that thorough drying resulted not possible, and some Et_2O can be observed in the NMR spectrum. Anyway, it is possible to isolate it via flash chromatography and remove the solvent on the rotavap.

7.1 Trapping with TEMPO radical **4b**

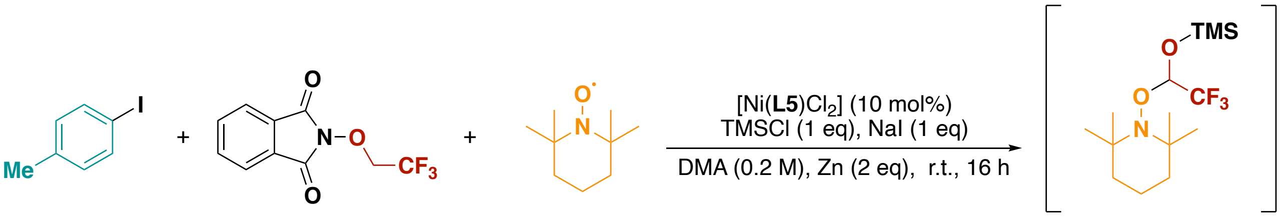

1a **2** **4b** **5b**

observed by GC-MS

Radical trapping experiment with TEMPO **4b** was set up with substrate **1a** according to General procedure (see section 5.1), simply by adding **4b** (2 eq, 0.2 mmol, 31.3 mg) to the reaction mixture right before TMSCl.

After addition of EtOAc the reaction was quenched using sat. aq. NH_4Cl instead of 2N HCl, and an aliquot of the organic phase was directly injected into GC-MS.

GC-MS shows that no product was formed, and the mixture consisted of unreacted **1a**, **2**, phthalimide, small amount of adduct **5b** and some TEMPO-derived byproducts (**Figure S9**).

```
File        :C:\msdchem\1\DATA\Smistamento\LL-1739GR.D
Operator    :
Acquired    : 30 Jun 2022  10:17     using AcqMethod AG.M
Instrument :     GC-MS
Sample Name:
Misc Info  :
Vial Number: 1
```

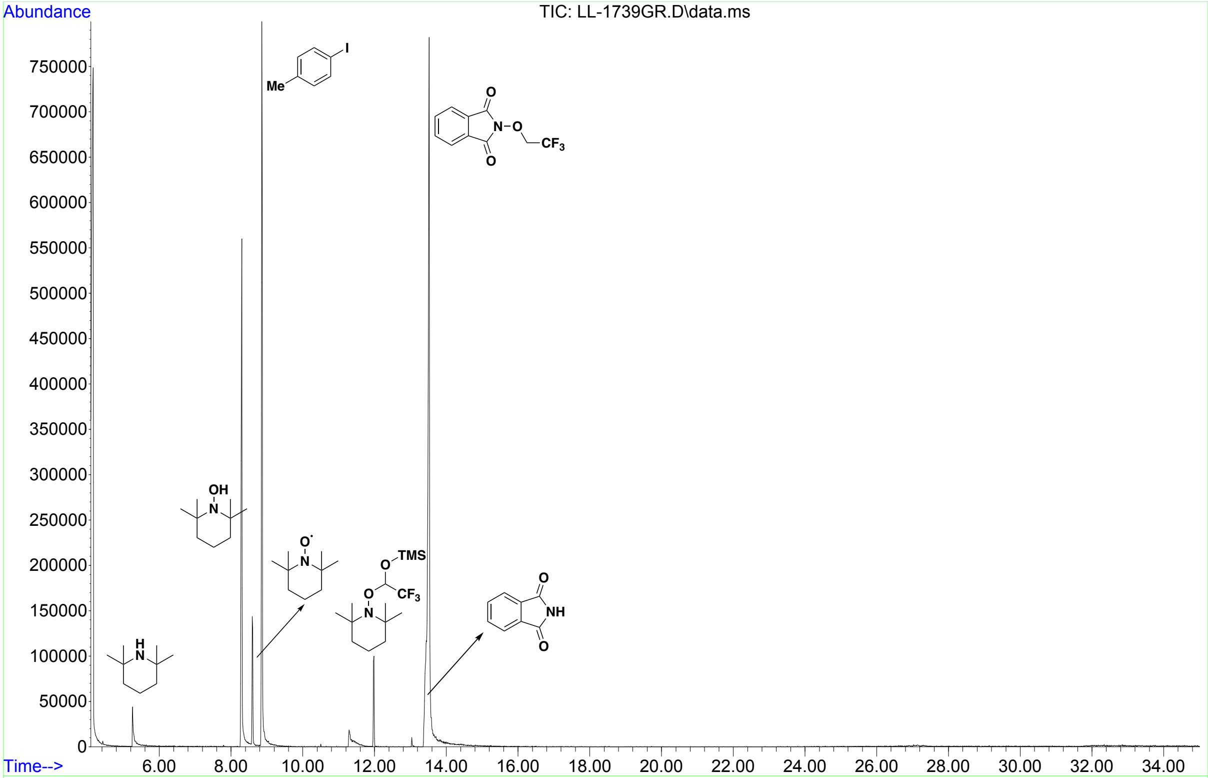

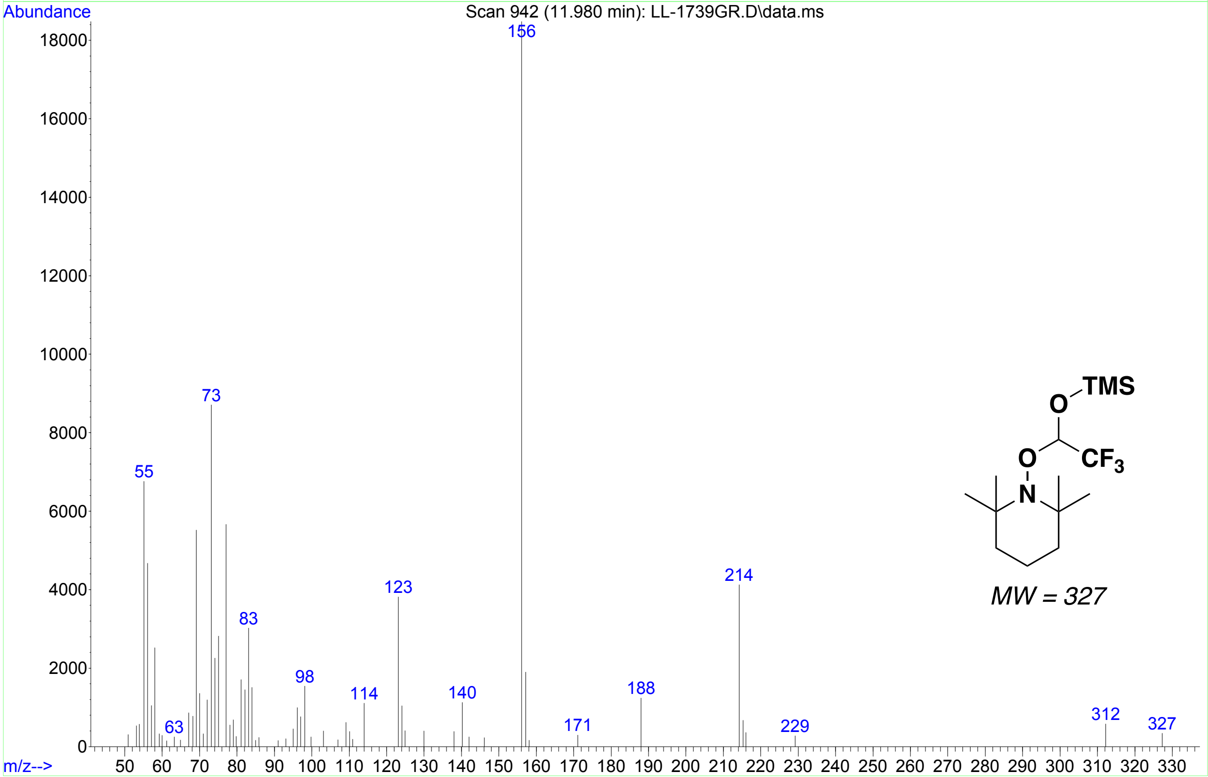

Figure S9: GC-MS analysis of the crude mixture for TEMPO radical trapping experiment. Mass spectrum of the observed adduct **5b** is given.

8. Control experiments

8.1 Experiment without Ni catalyst

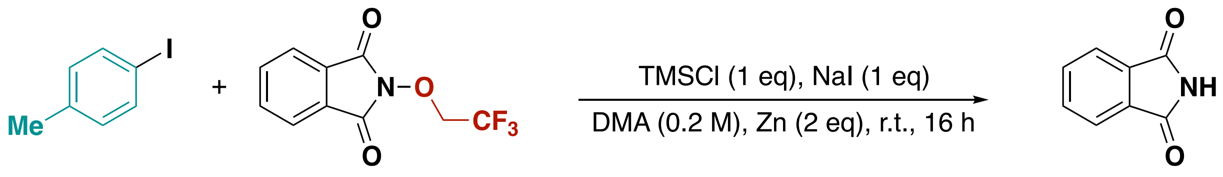

1a **2**

The reaction was set up with substrate **1a** according to General Procedure (see section 5.1), but omitting the catalyst [Ni(**L5**)Cl_2]. GC-MS and ^{19}F-NMR analysis of the mixture after 16 h show unreacted **1a**, complete conversion of **2** to phthalimide, but no product. This further confirms that activation of **2** is mediated by Zn and proves that Ni is essential for the formation of the product.

8.2 Experiment without Zn

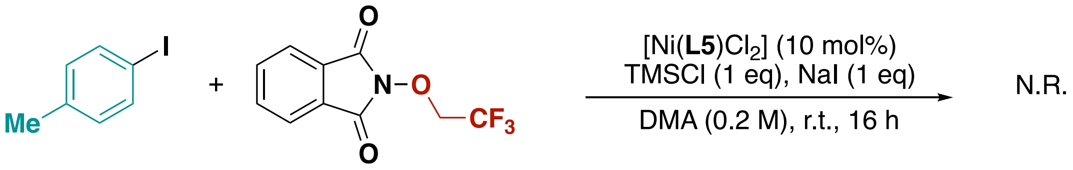

1a **2**

The reaction was set up with substrate **1a** according to General Procedure (see section 5.1) but omitting Zn dust. Both GC-MS and ^{19}F-NMR analysis of the mixture after 16 h only shows both unreacted starting materials.

8.3 Experiment for Zn insertion with aryl iodide **1u**

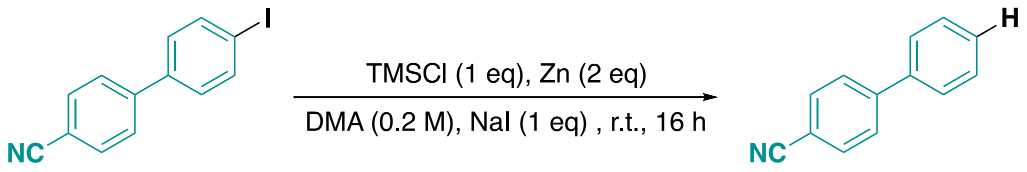

1z **1z(H)**

The reaction was set up with substrate **1z** according to General Procedure (see section 5.1), but omitting both **2** and Ni catalyst, and stopped after 16 h.

Given that homogeneous reductant TDAE proved unsuccessful in promoting any conversion of both starting materials, we considered appropriate to investigate whether Zn insertion into the C-I bond was occurring. This was done by quantifying the amount of dehalogenated starting material **1z(H)**, that would be arising from the protonation of the arylzinc derived from **1z** after work-up.

^{1}H-NMR analysis with mesitylene as an internal standard showed that **1z(H)** was formed in 22% yield, the rest being unreacted **1z**. The small extent of protodeiodination observed after 16 h indicates that insertion of Zn into the C-I occurs at a very low rate, leading us to consider unlikely the intermediacy of organozinc species in the present methodology.

<u>8.4 Experiment with Trifluoroacetaldehyde ethyl hemiacetal</u>

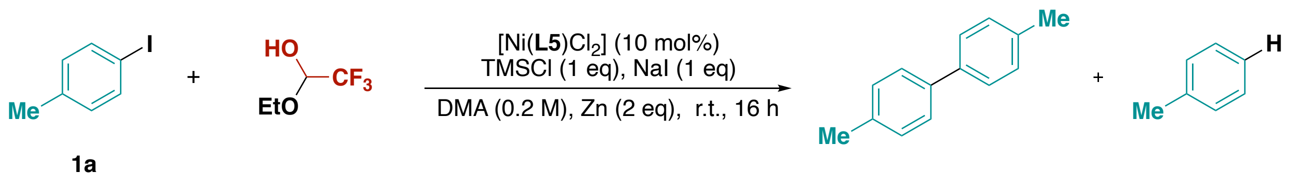

The reaction was set up with substrate **1a** according to General Procedure (see section 5.1), substituting **2** with Trifluoroacetaldehyde ethyl hemiacetal (3 eq). GC-MS, ^{1}H-NMR and ^{19}F-NMR analysis of the mixture after 16 h show complete conversion of **1** to 4,4′-dimethylbiphenyl and toluene (via Ni catalyzed homocoupling and protodehalogenation, respectively), but no product.

9. ^{1}H-, ^{19}F-, ^{13}C-NMR Spectra

2' ^{1}H NMR (400 MHz, $CDCl_3$)

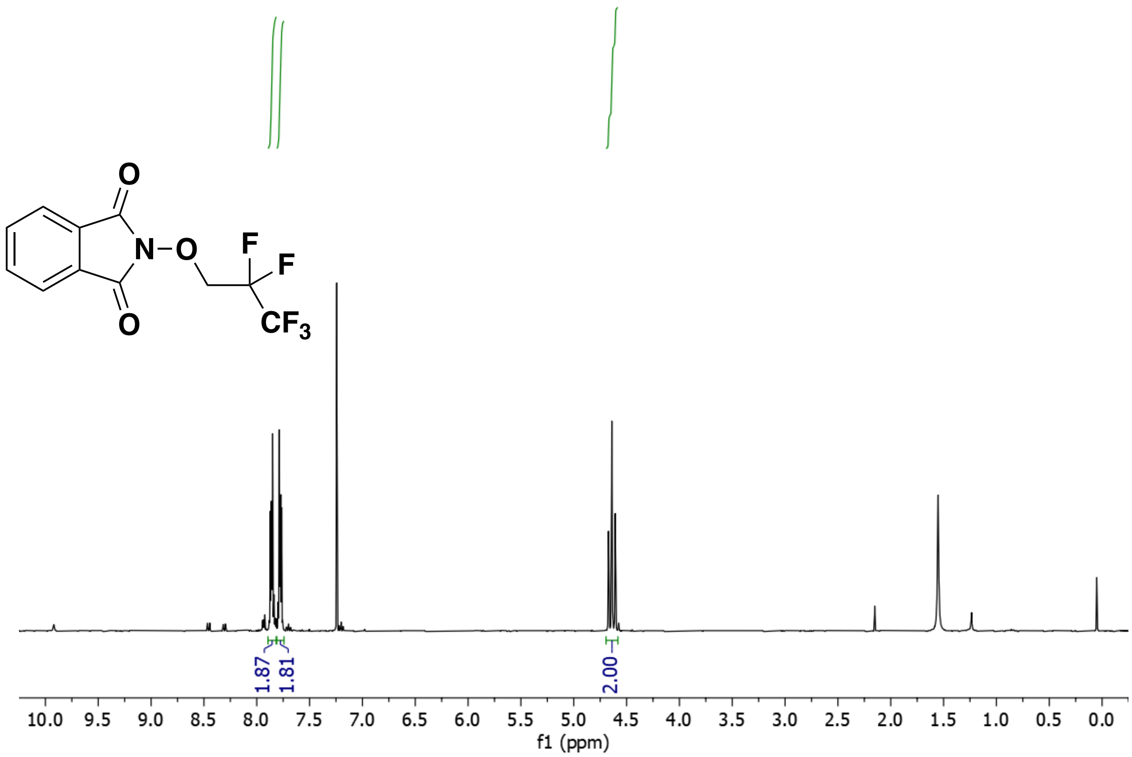

2' ^{13}C NMR (100 MHz, $CDCl_3$)

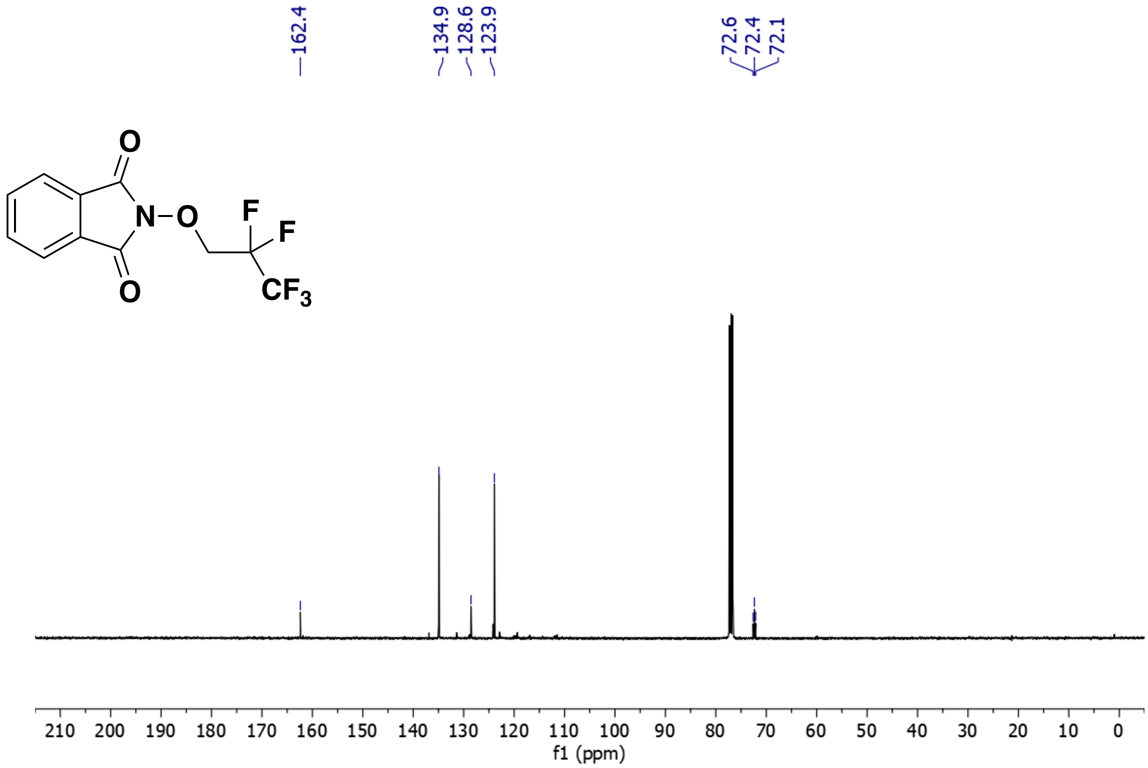

2’ ^{19}F NMR (377 MHz, $CDCl_3$)

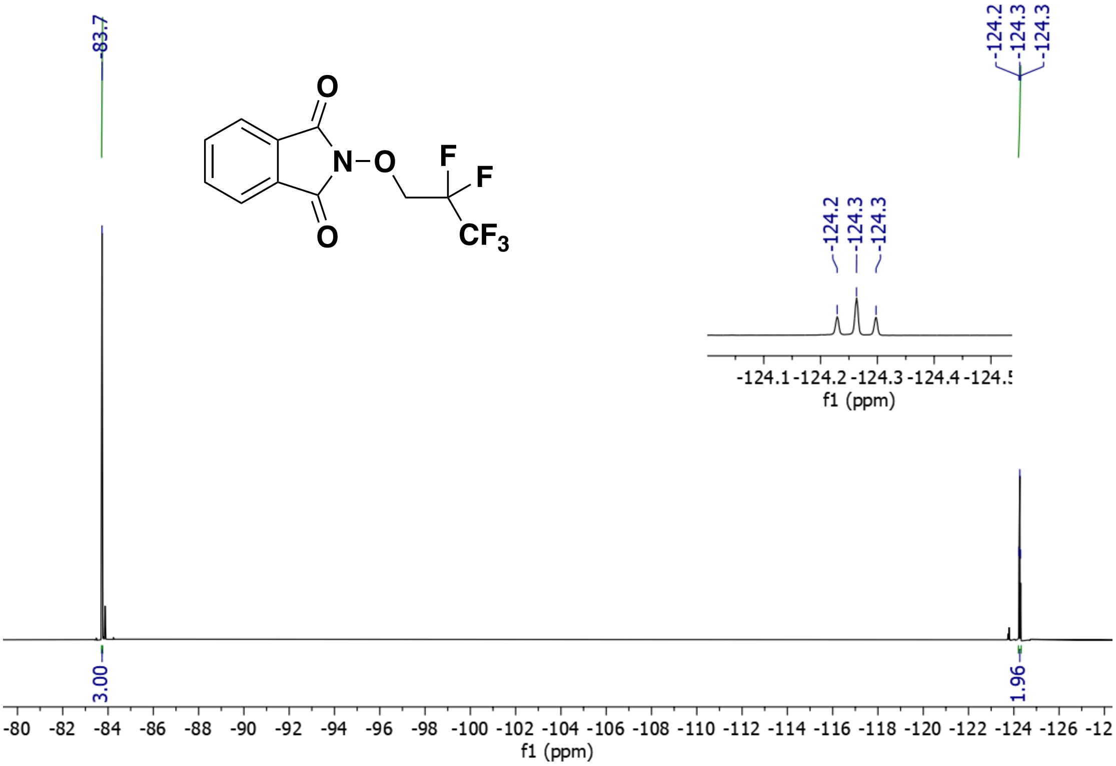

3a ^{1}H NMR (400 MHz, $CDCl_3$)

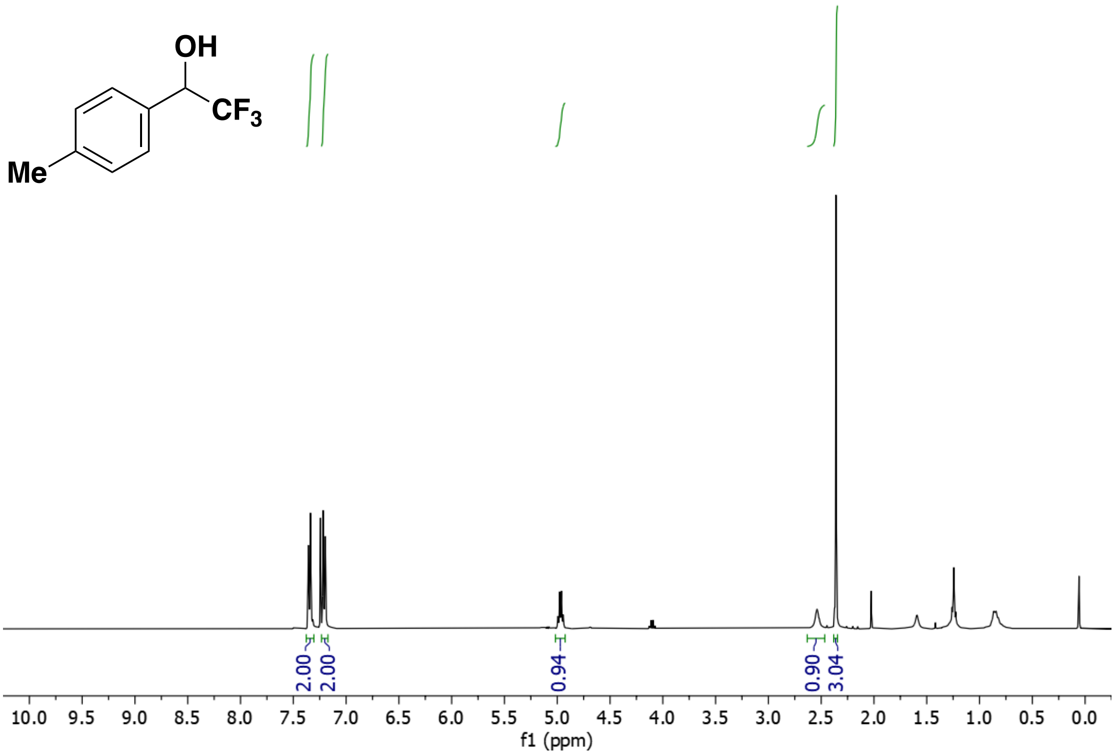

3a ^{13}C NMR (100 MHz, $CDCl_3$)

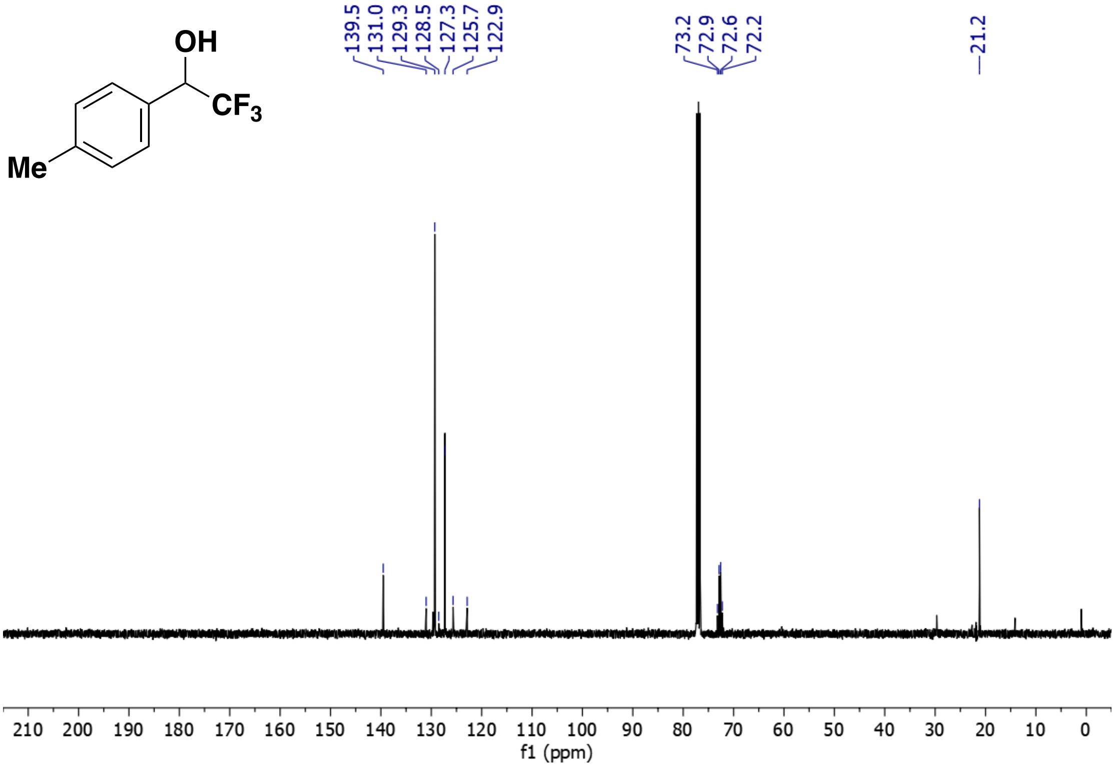

3a ^{19}F NMR (377 MHz, $CDCl_3$)

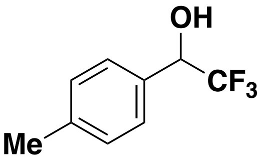

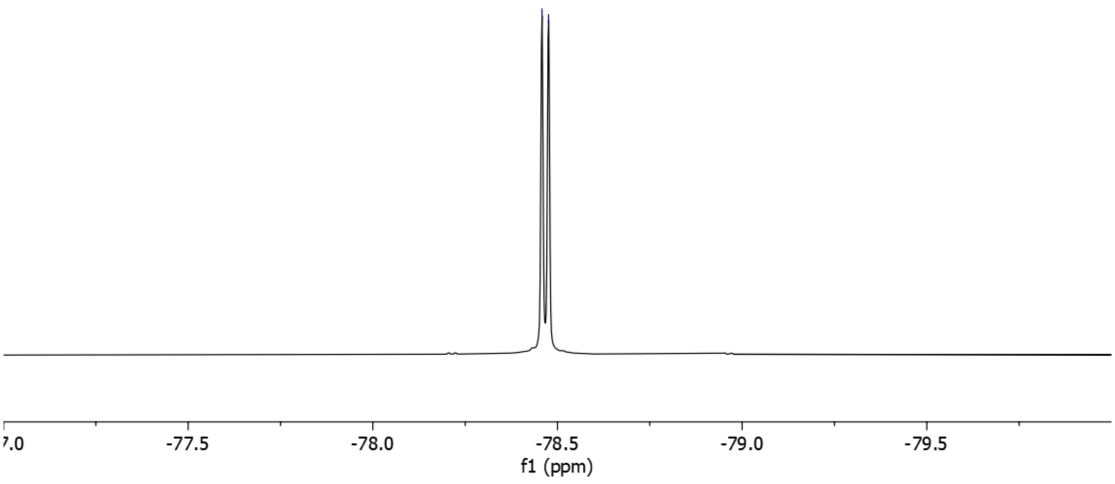

3b ¹H NMR (400 MHz, CDCl₃)

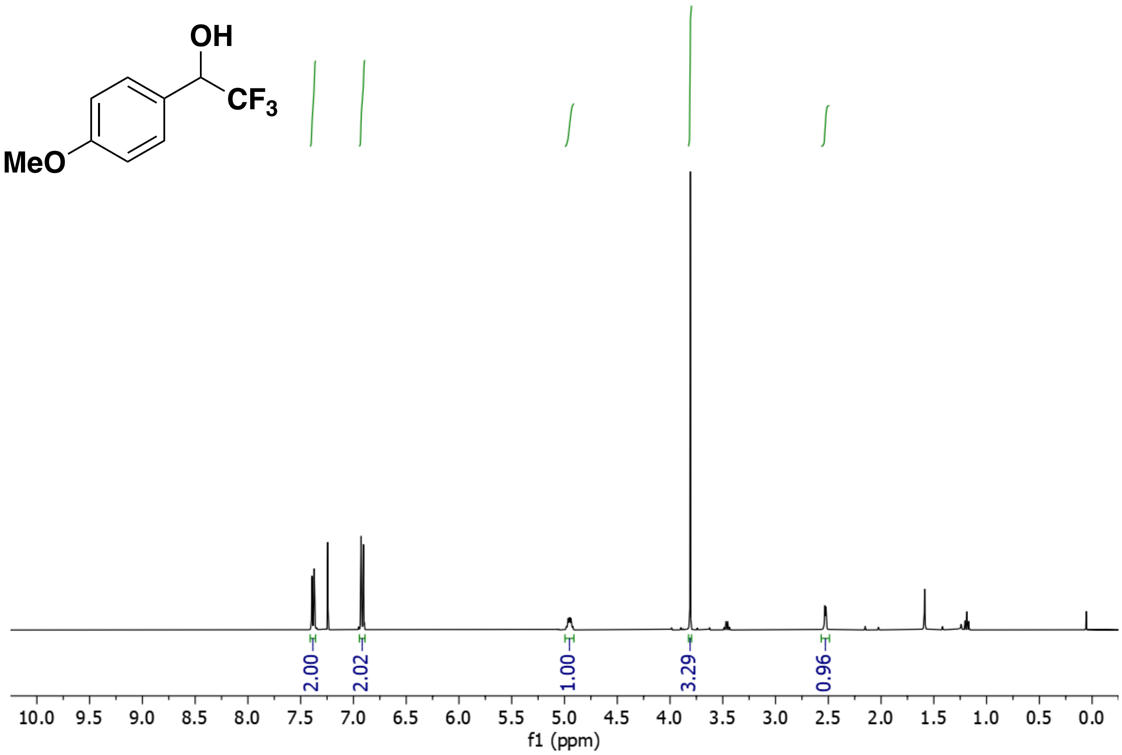

3b ¹³C NMR (100 MHz, CDCl₃)

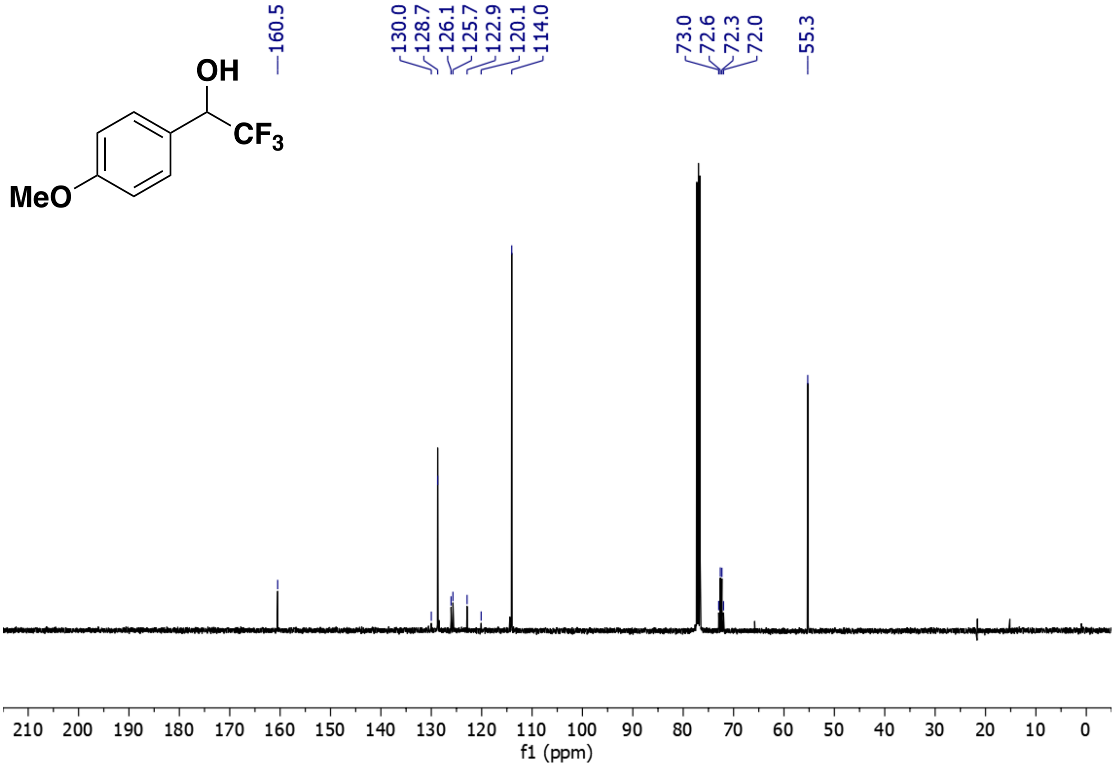

3b ^{19}F NMR (377 MHz, $CDCl_3$)

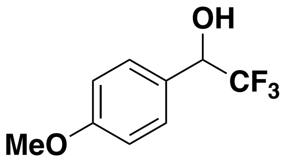

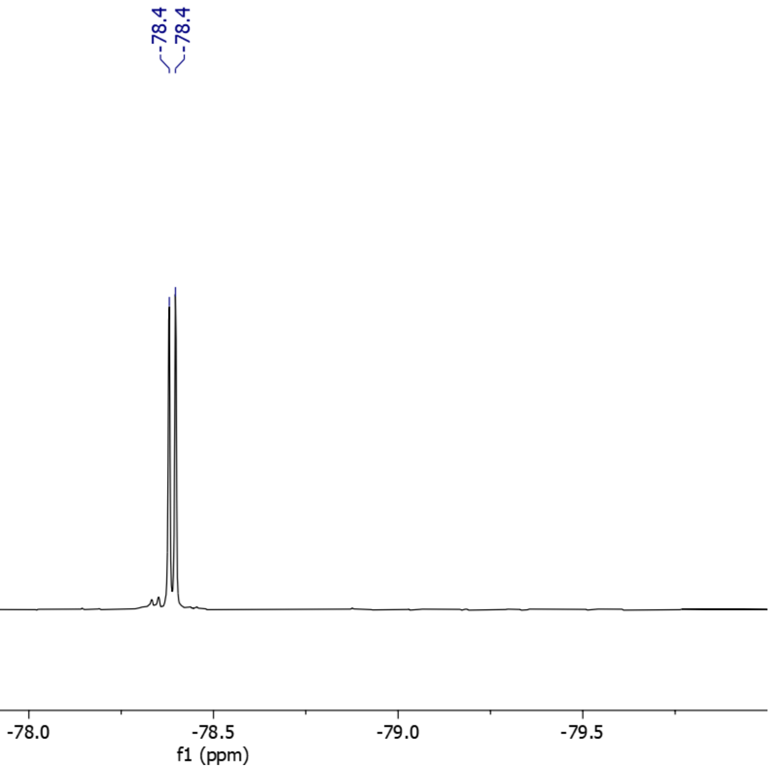

3c ^{1}H NMR (400 MHz, $CDCl_3$)

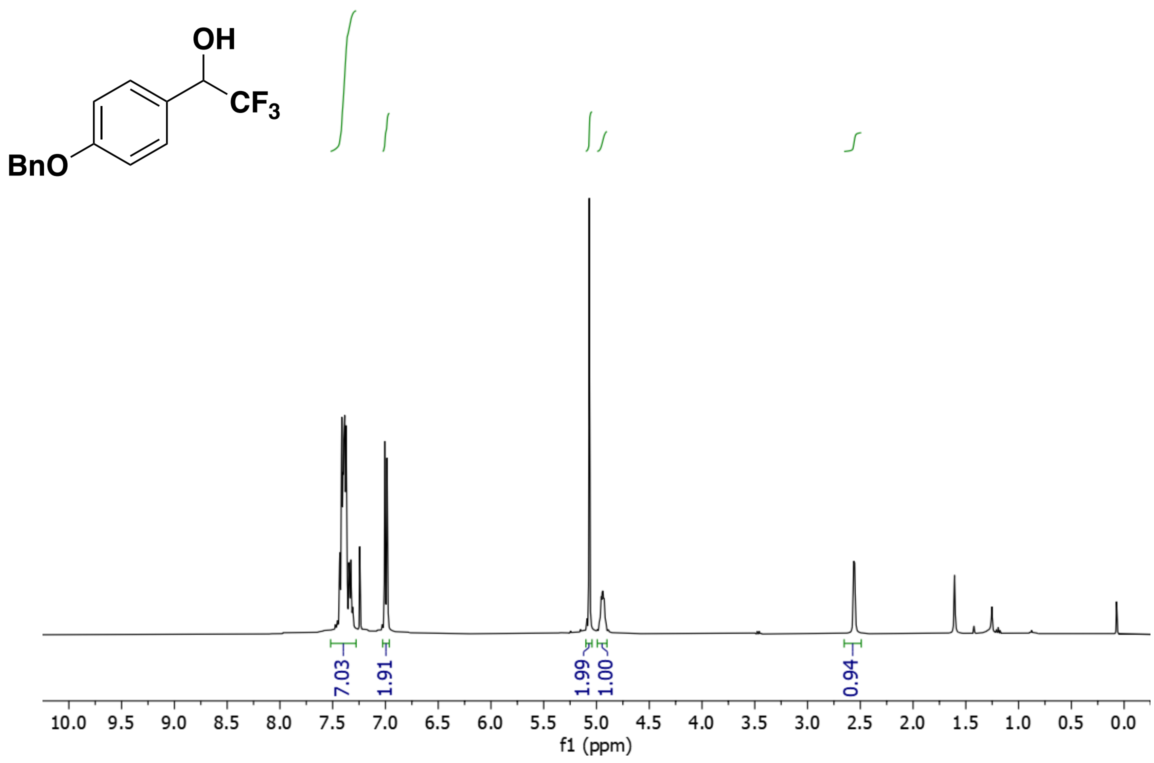

3c ^{13}C NMR (100 MHz, $CDCl_3$)

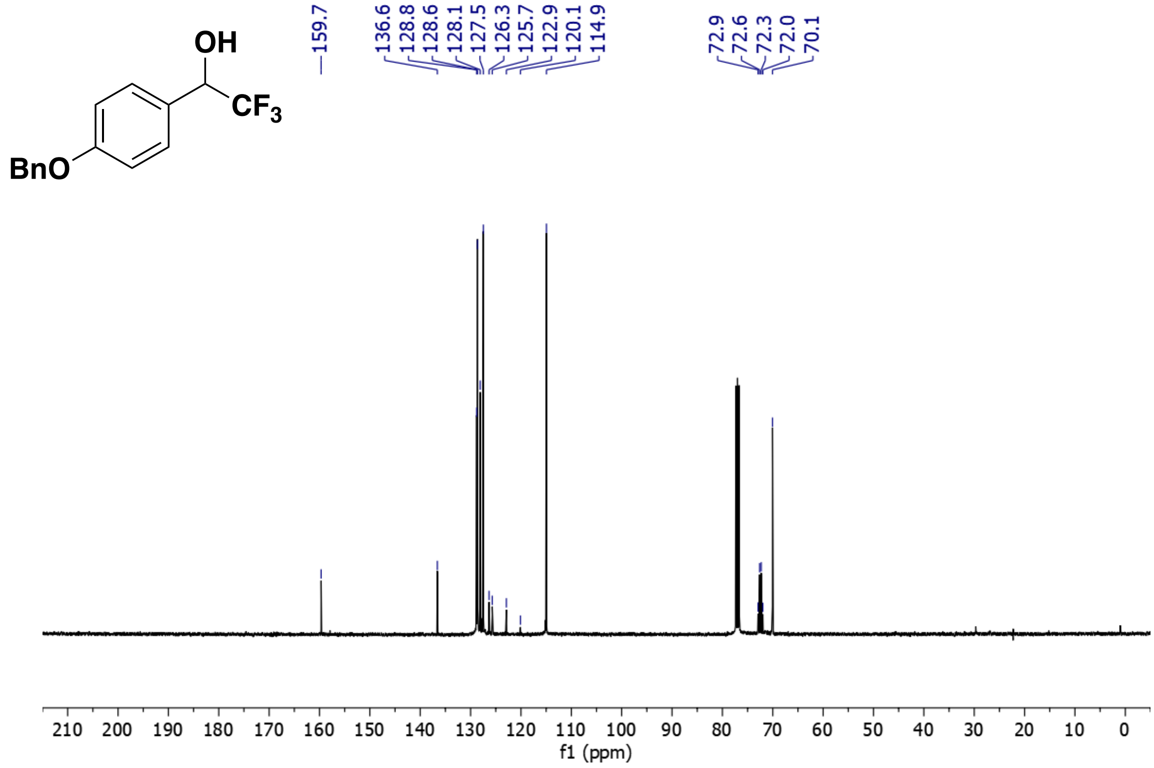

3c ^{19}F NMR (377 MHz, $CDCl_3$)

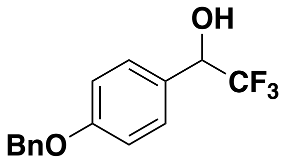

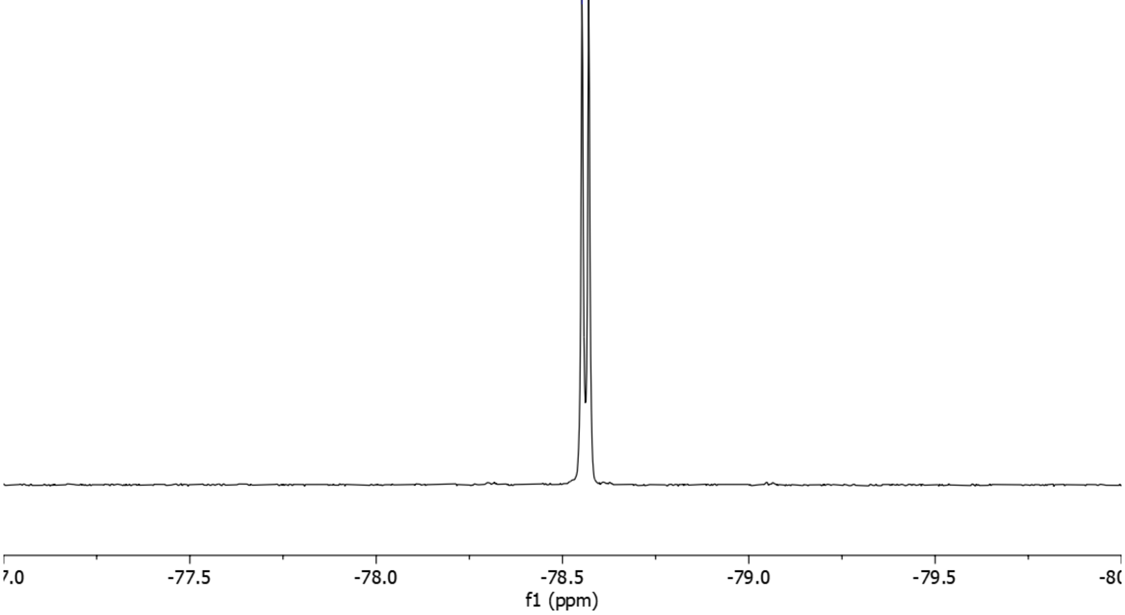

3d ¹H NMR (400 MHz, CDCl₃)

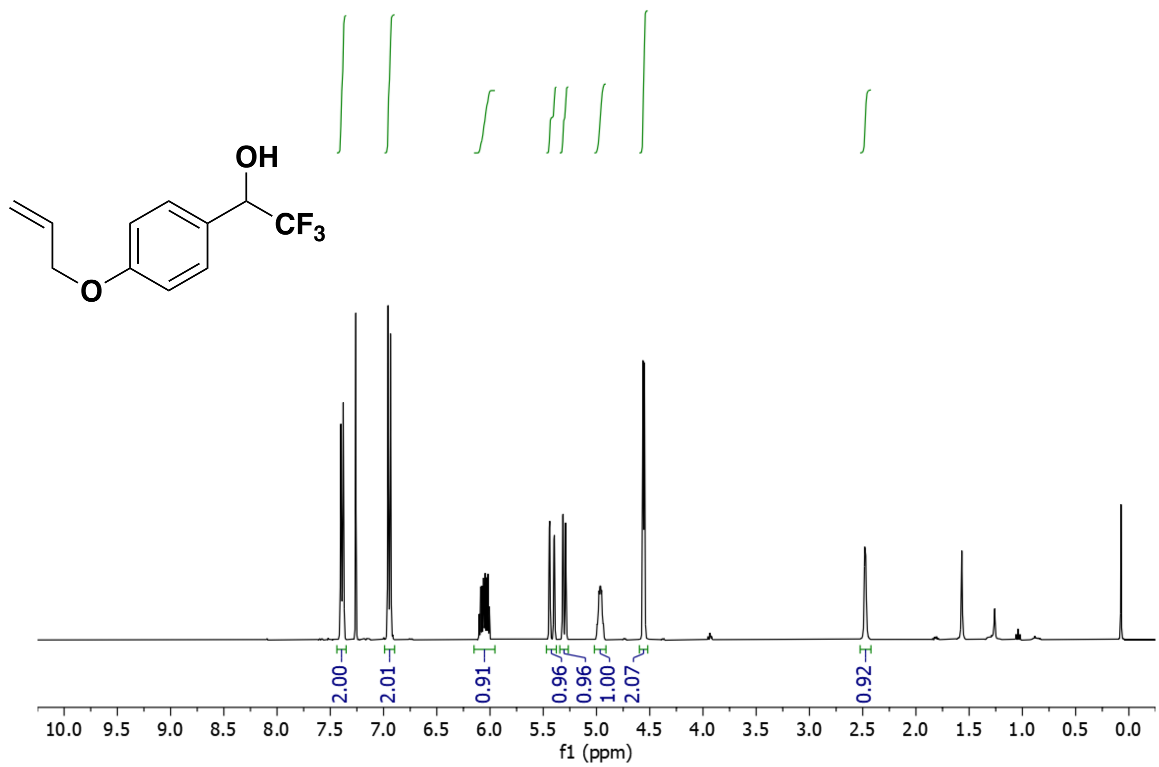

3d ¹³C NMR (100 MHz, CDCl₃)

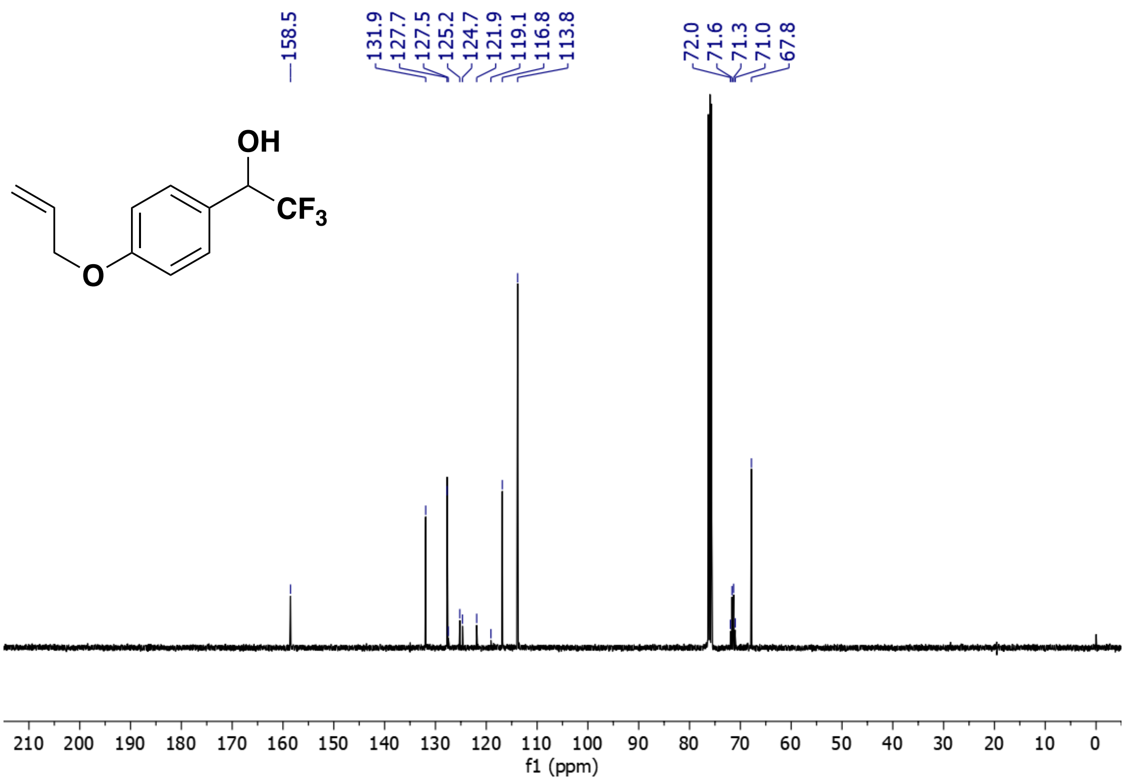

3d ^{19}F NMR (377 MHz, $CDCl_3$)

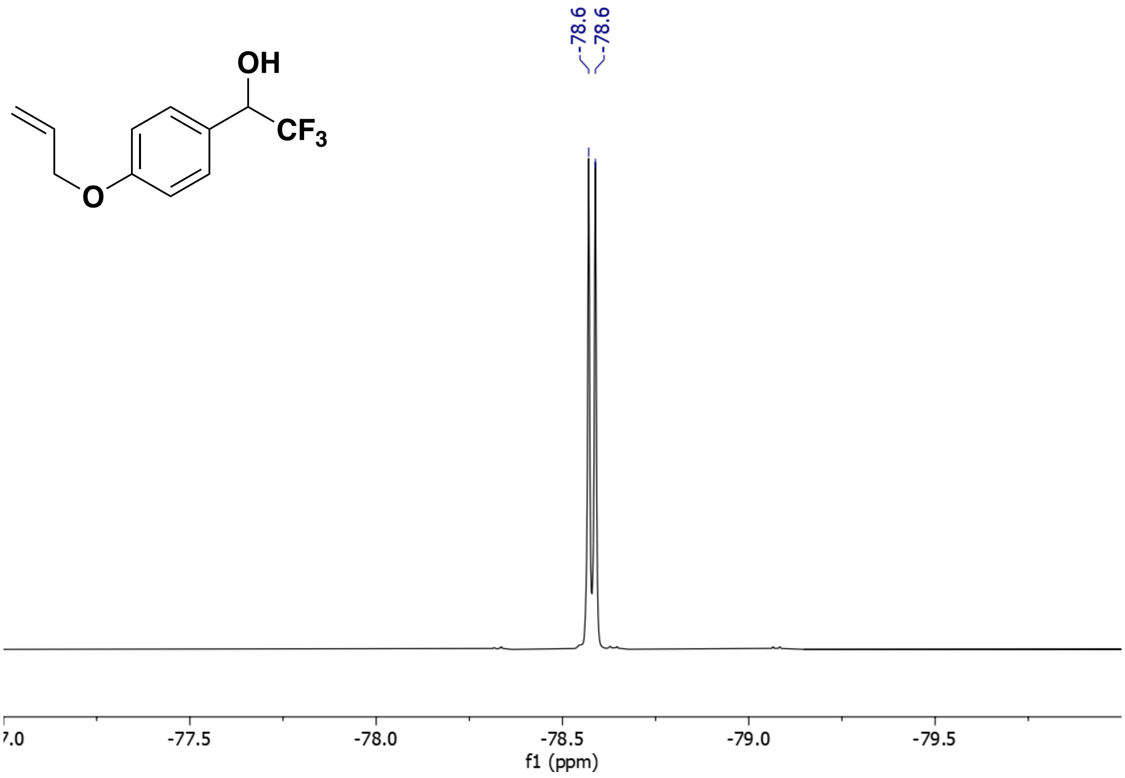

3e ^{1}H NMR (400 MHz, CDCl$_3$)

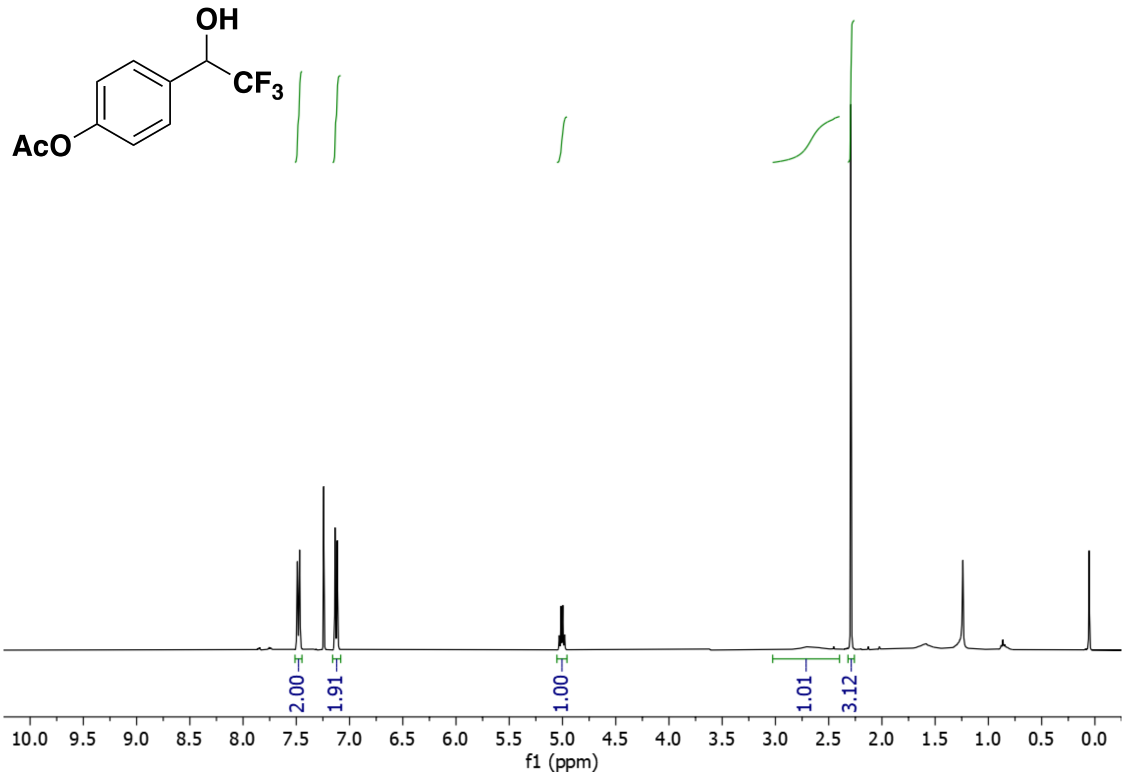

3e ^{13}C NMR (100 MHz, CDCl$_3$)

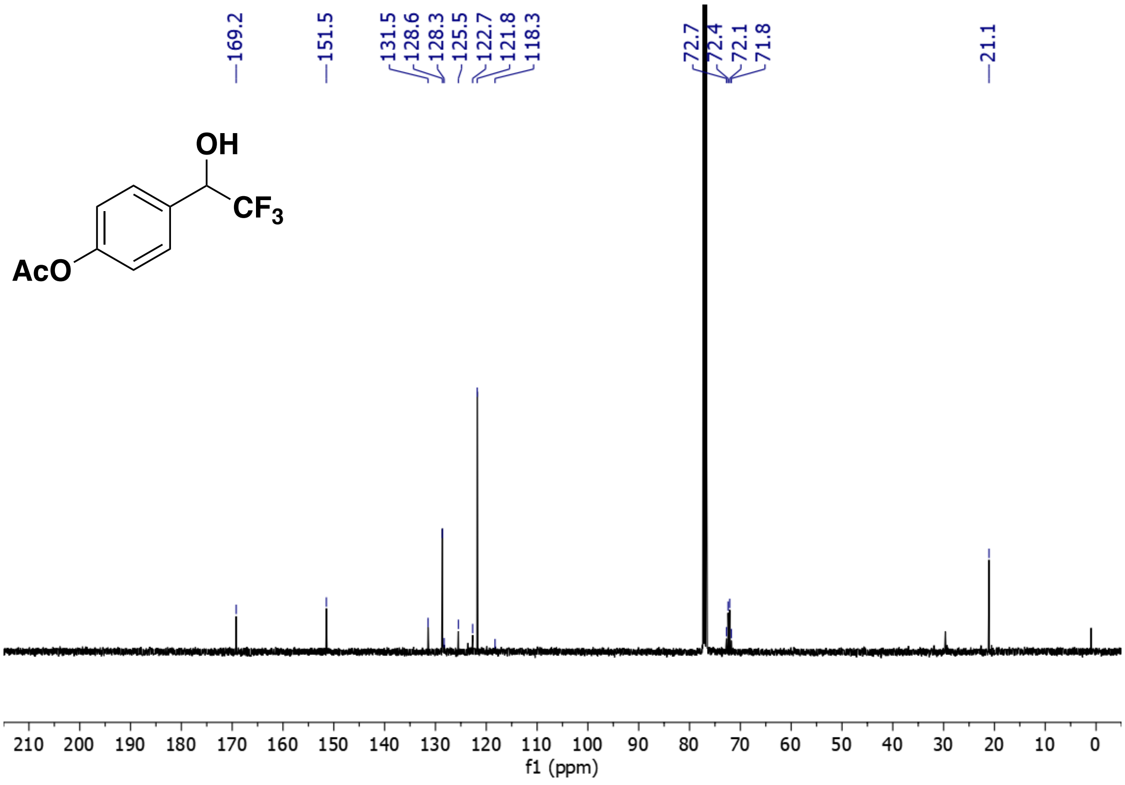

3e ^{19}F NMR (377 MHz, $CDCl_3$)

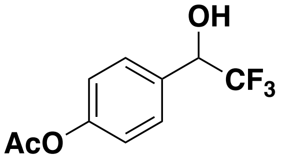

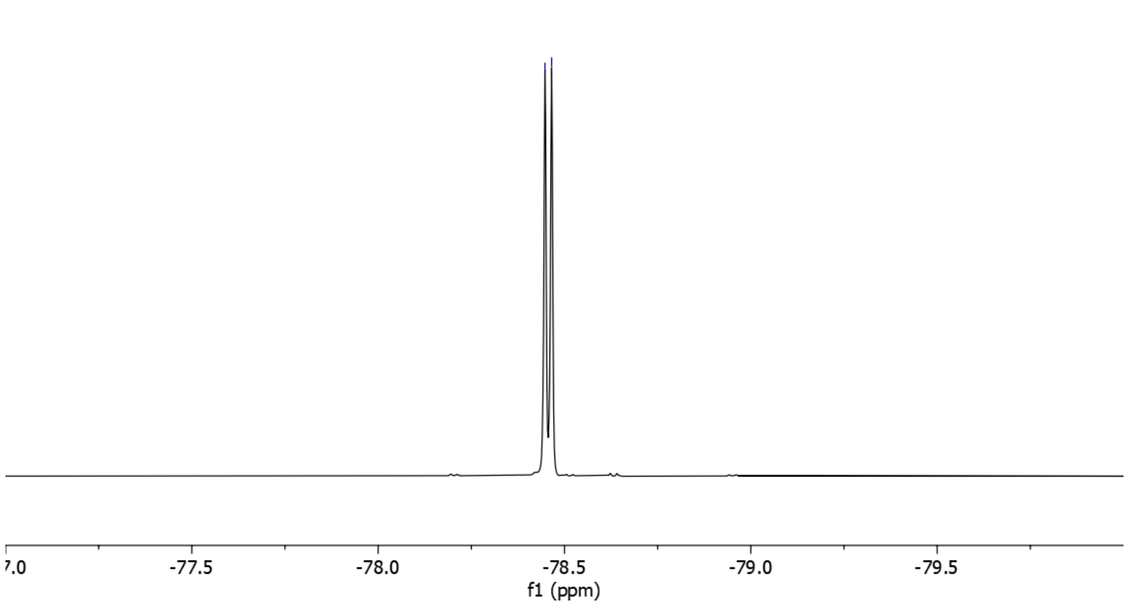

3f ^{1}H NMR (400 MHz, acetone-d_6)

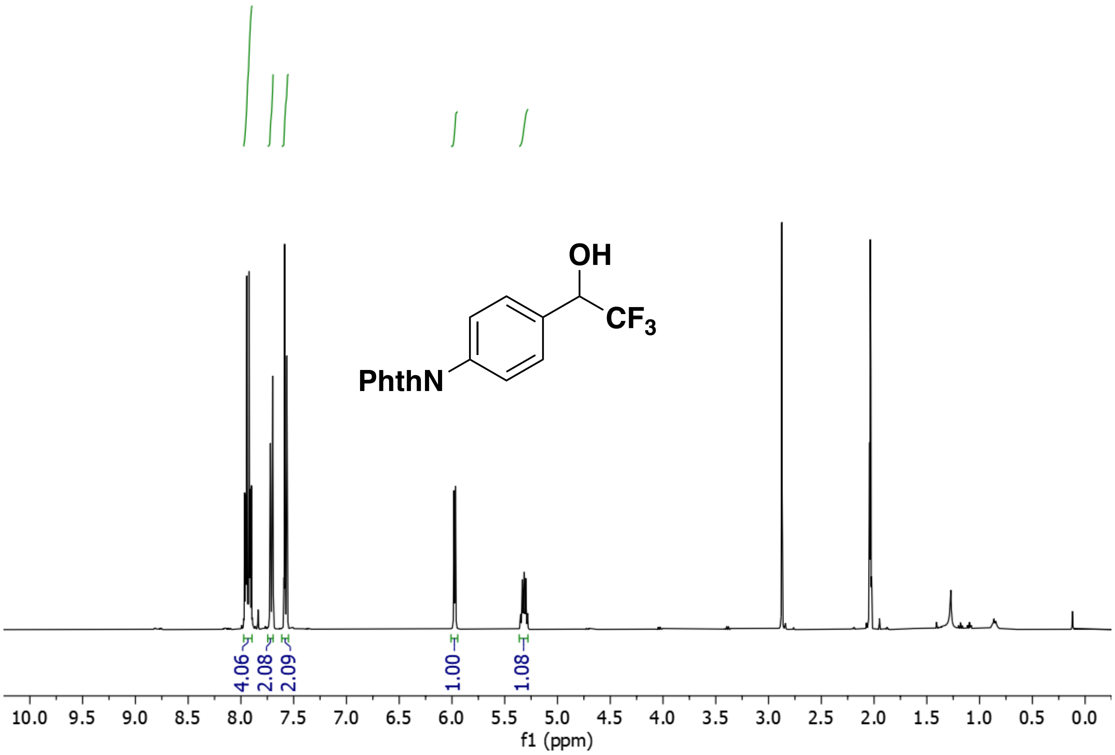

3f ^{13}C NMR (100 MHz, acetone-d_6)

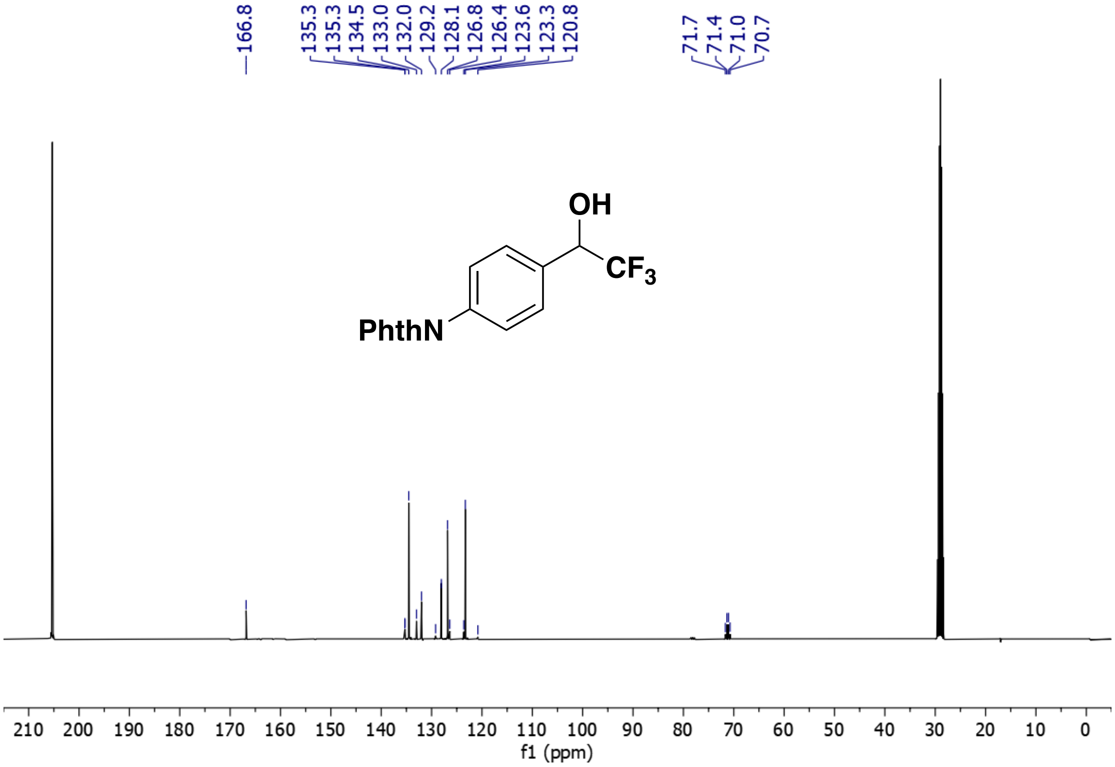

3f ^{19}F NMR (377 MHz, acetone-d_6)

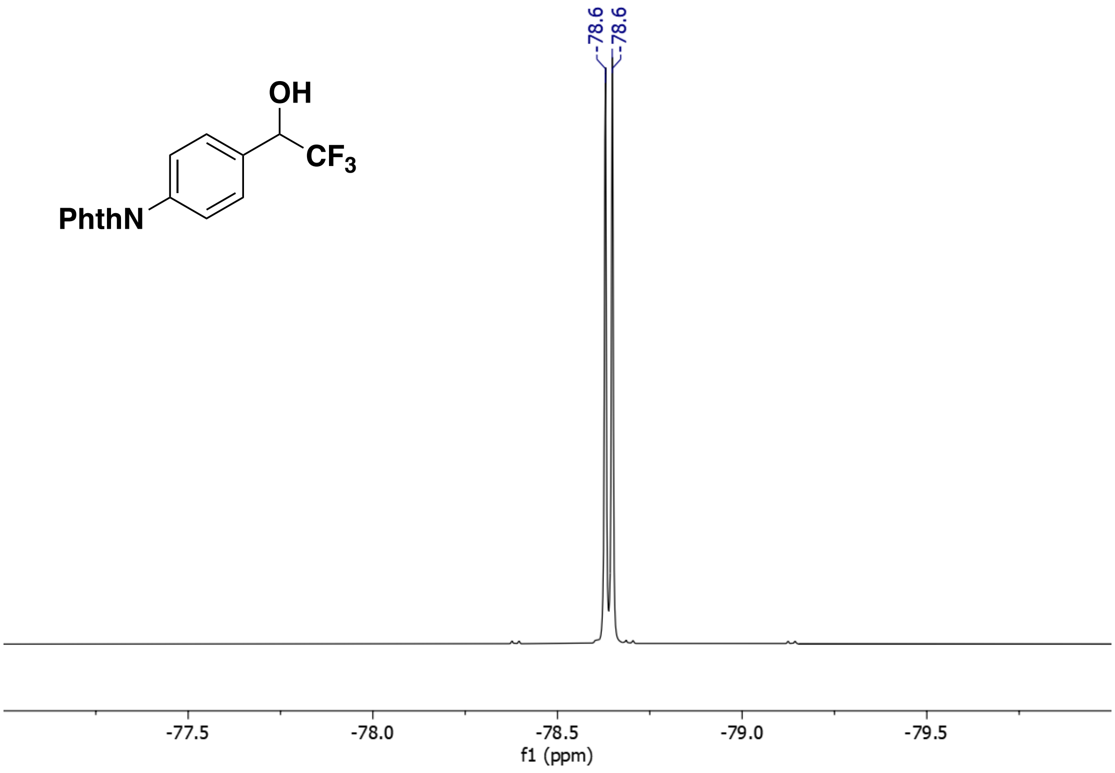

3g ^{1}H NMR (400 MHz, $CDCl_3$)

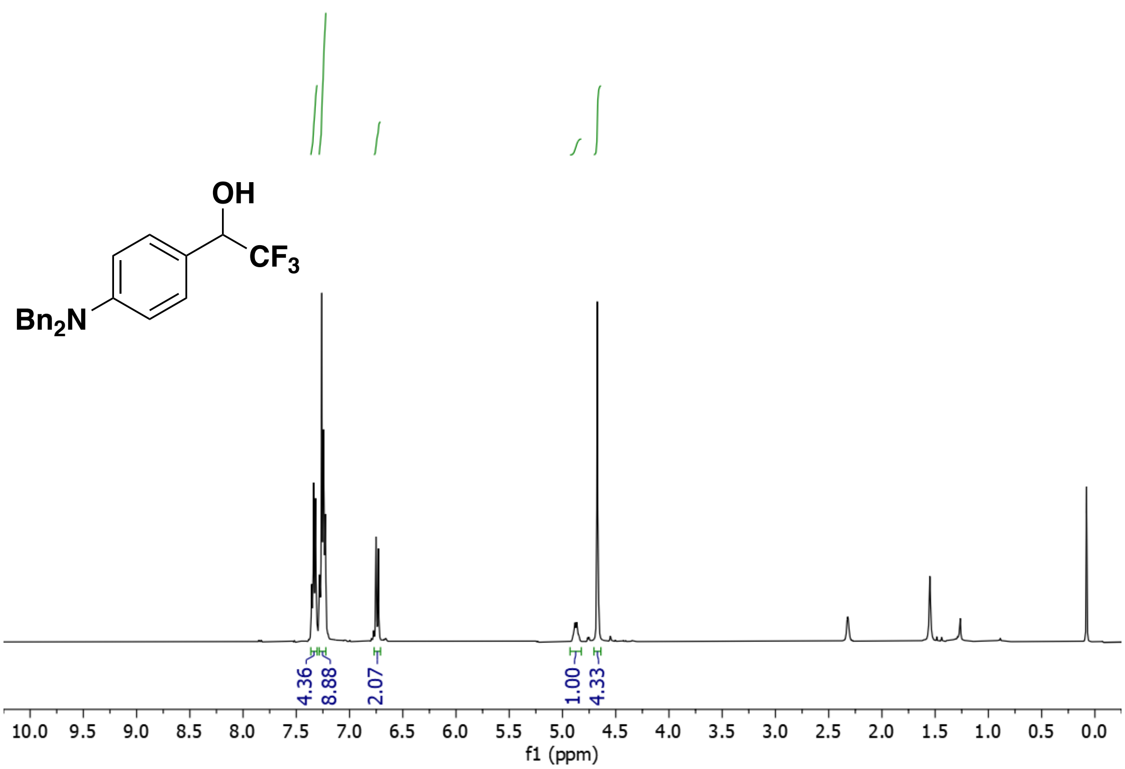

3g ^{13}C NMR (150 MHz, $CDCl_3$)

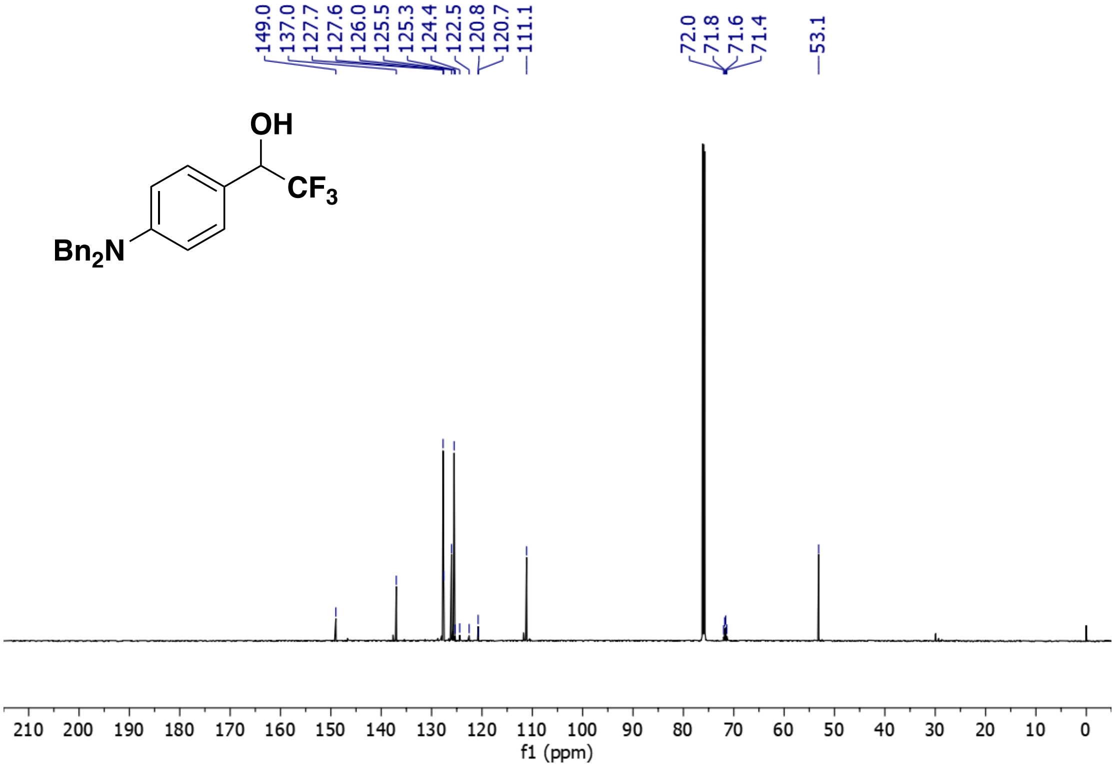

3g ^{19}F NMR (565 MHz, $CDCl_3$)

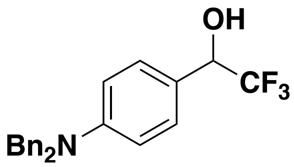

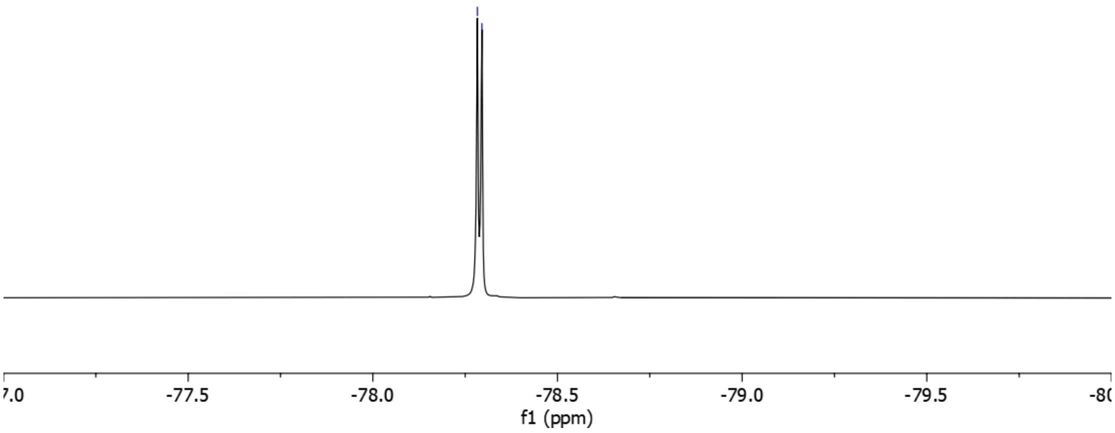

3h ^{1}H NMR (400 MHz, acetone-d_6)

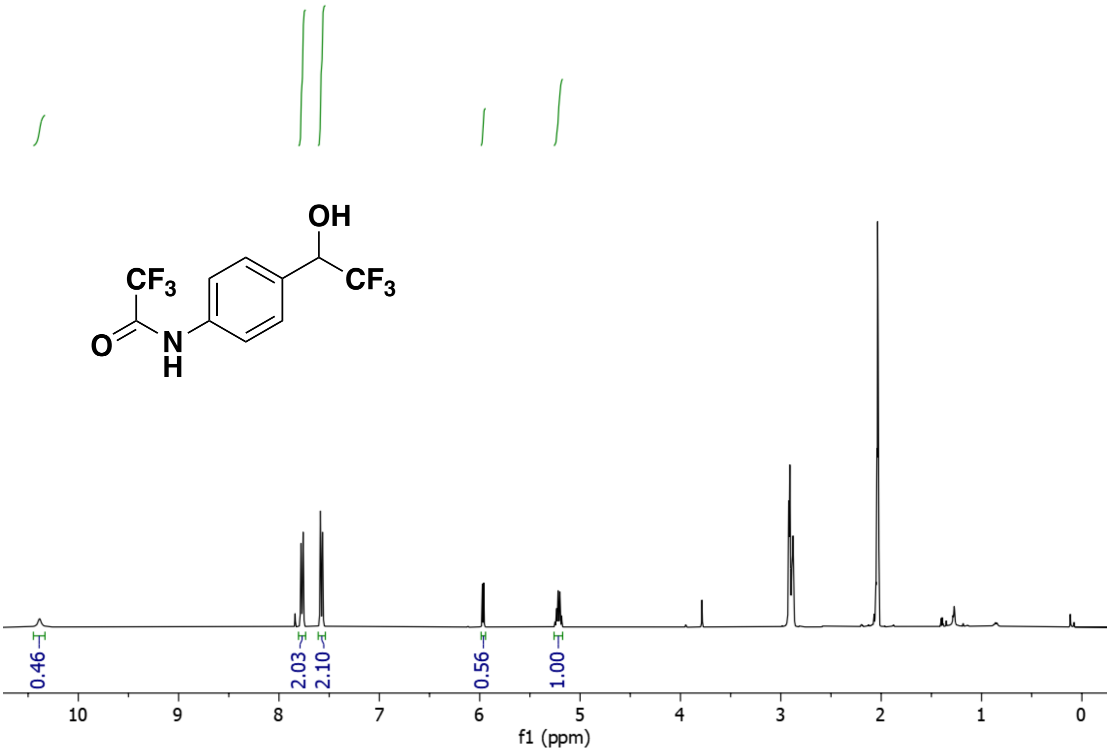

3h ^{13}C NMR (100 MHz, acetone-d_6)

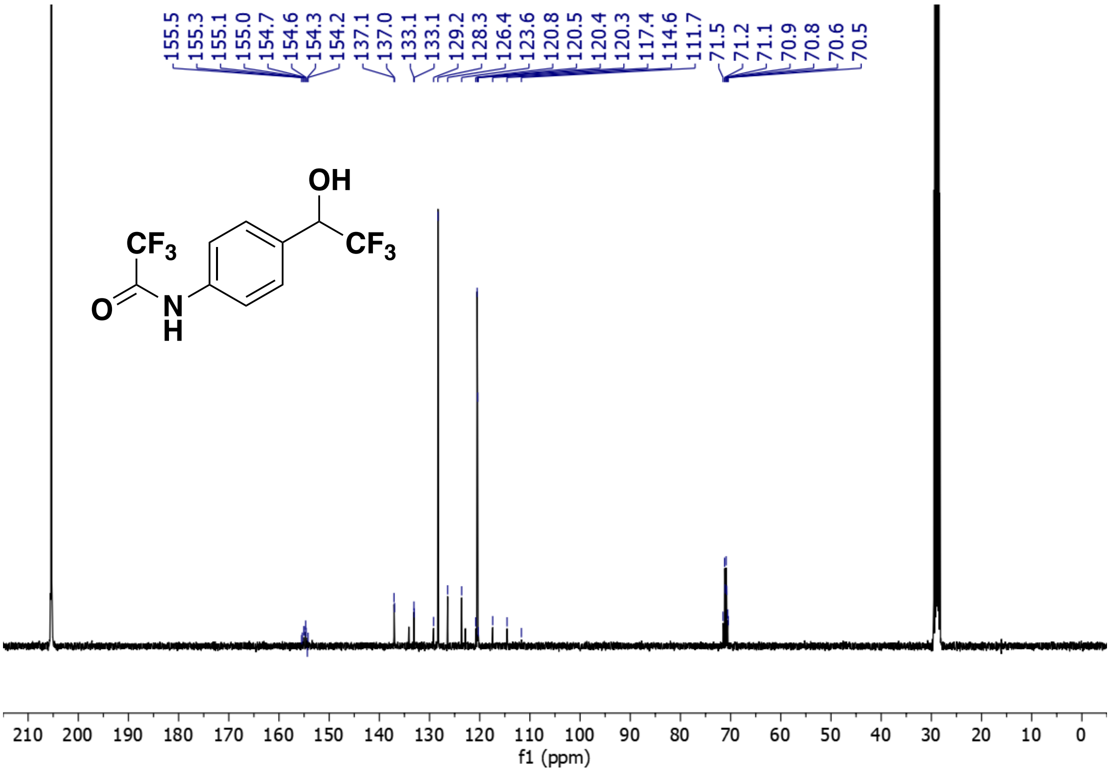

3h ^{19}F NMR (377 MHz, acetone-d_6)

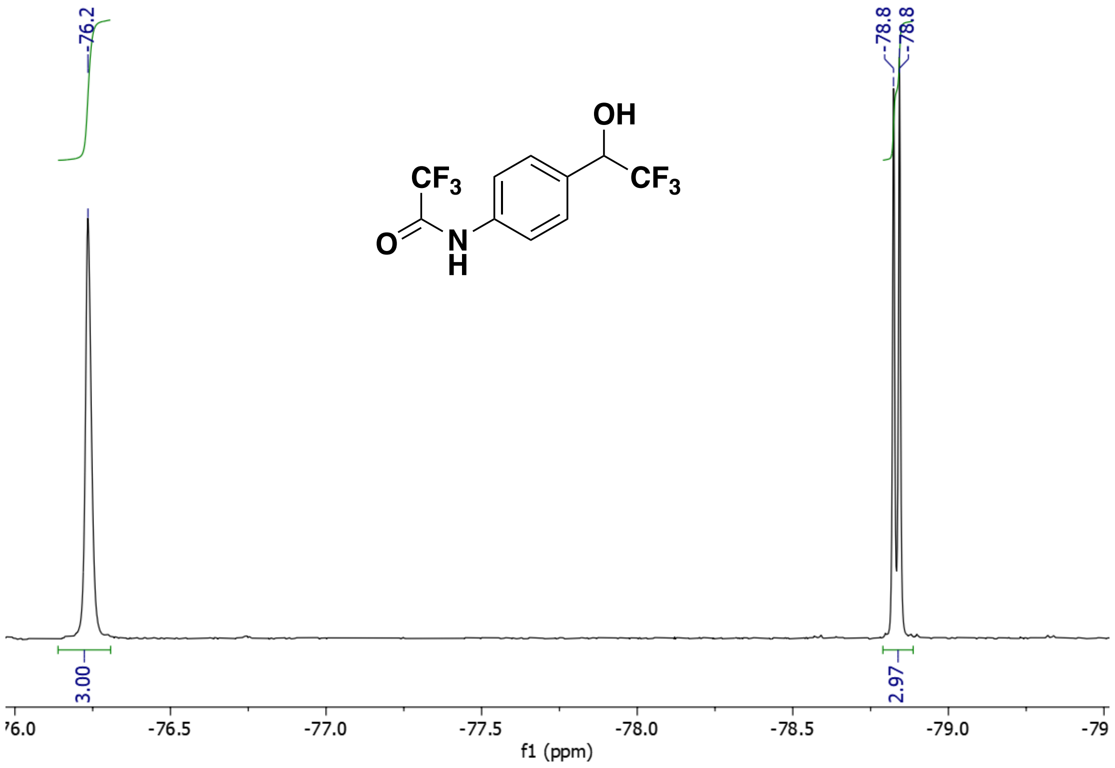

3i ^{1}H NMR (400 MHz, $CDCl_3$)

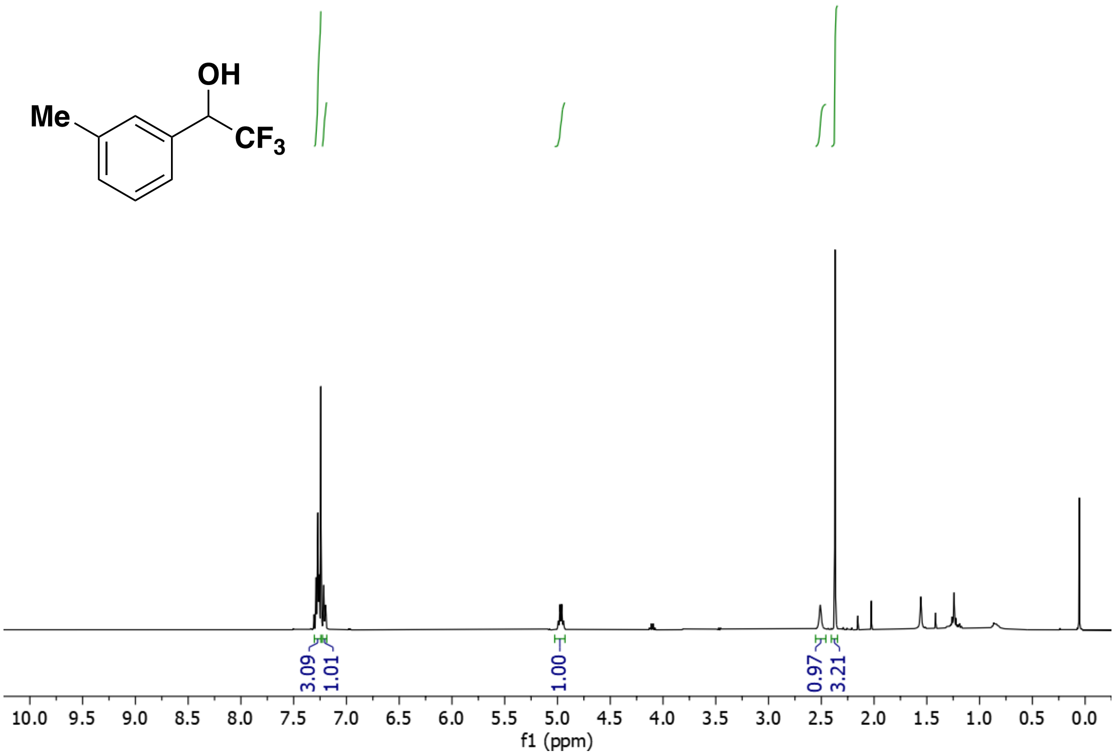

3i ^{13}C NMR (100 MHz, $CDCl_3$)

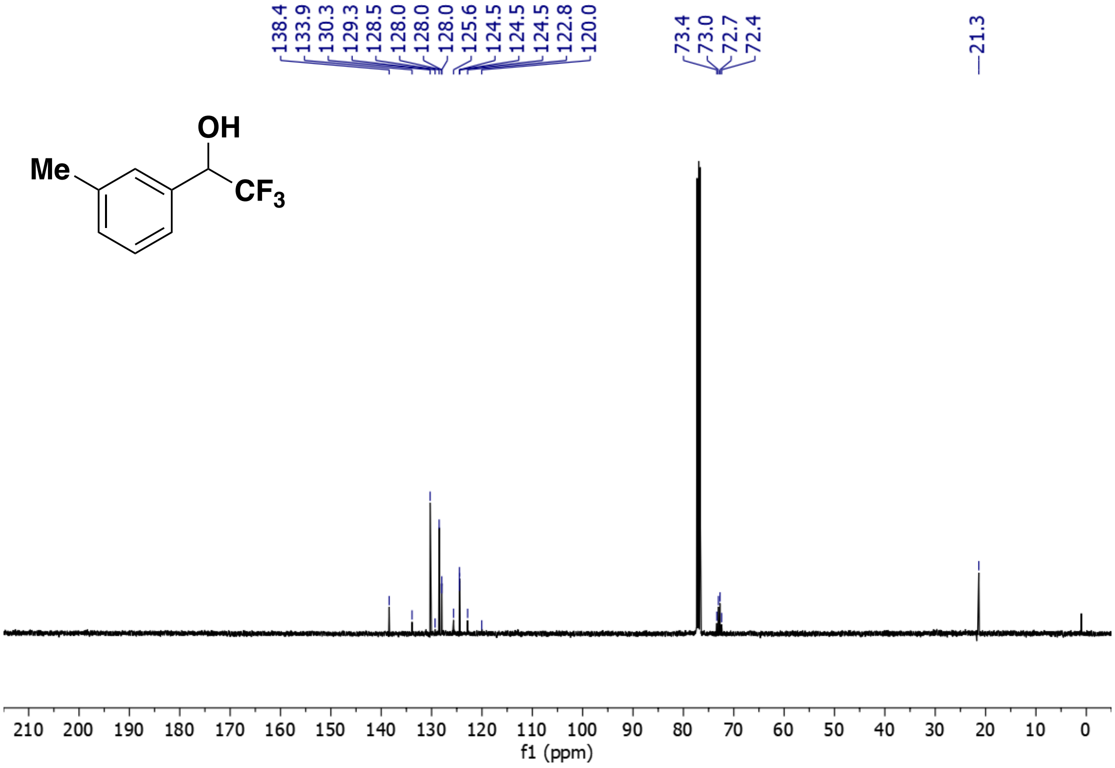

3i ^{19}F NMR (377 MHz, $CDCl_3$)

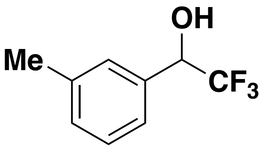

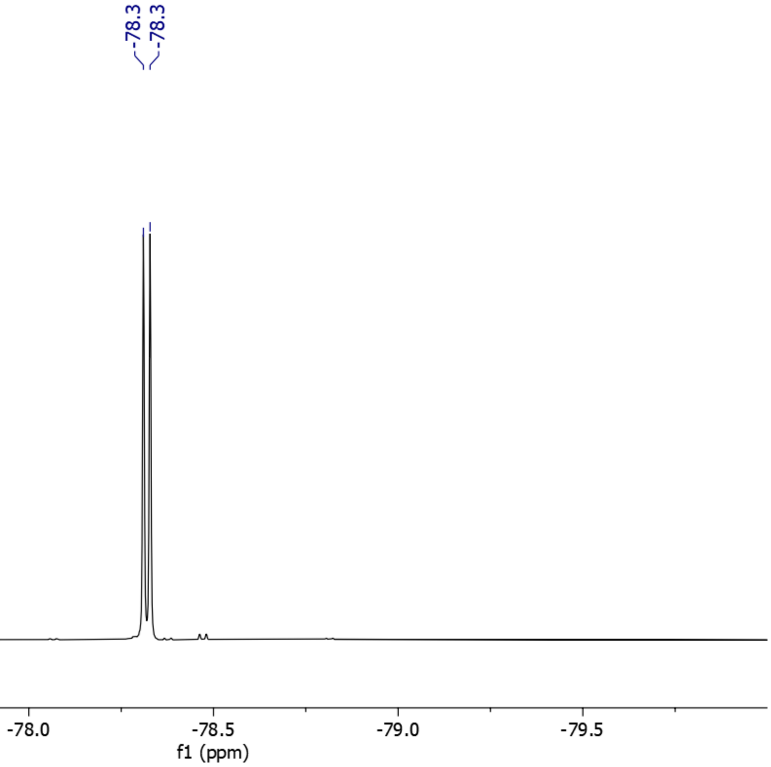

3j ^{1}H NMR (400 MHz, $CDCl_3$)

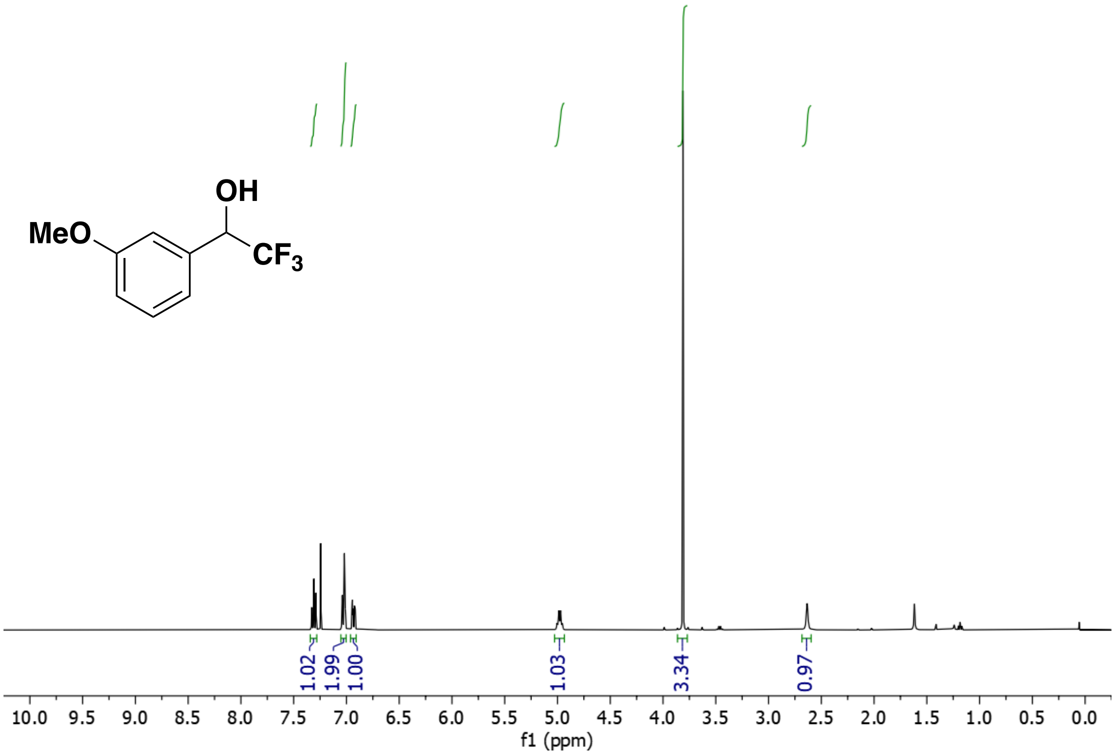

3j ^{13}C NMR (100 MHz, $CDCl_3$)

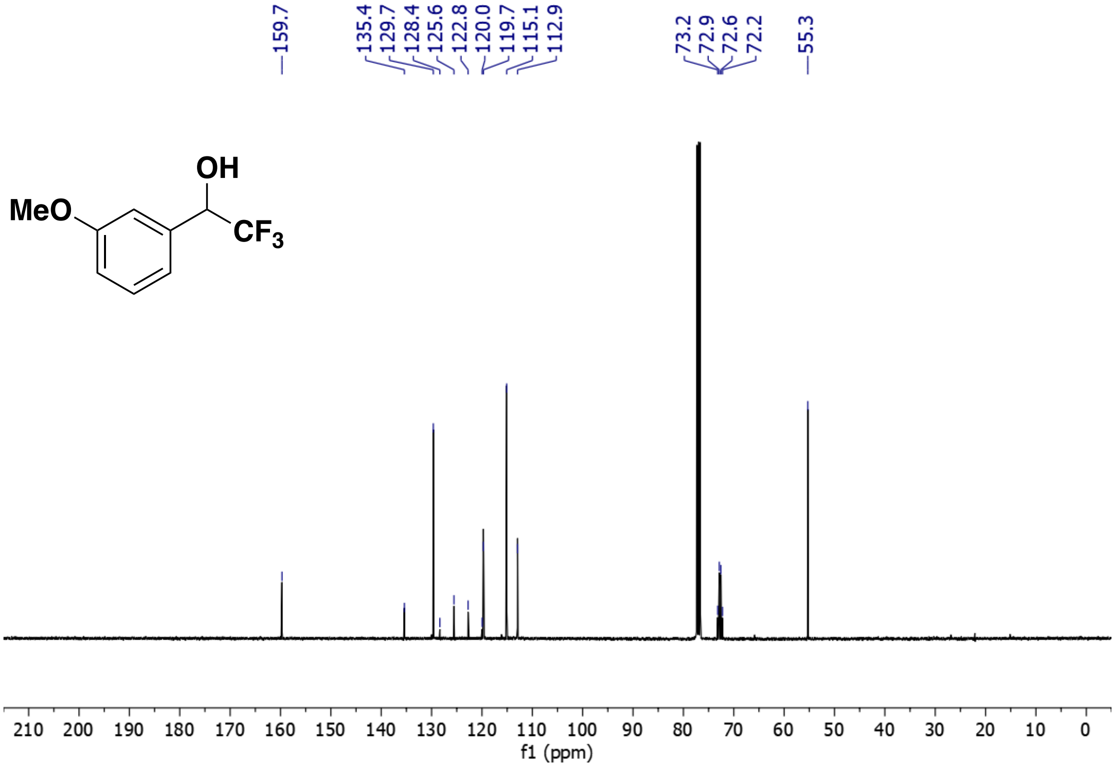

3j ^{19}F NMR (377 MHz, $CDCl_3$)

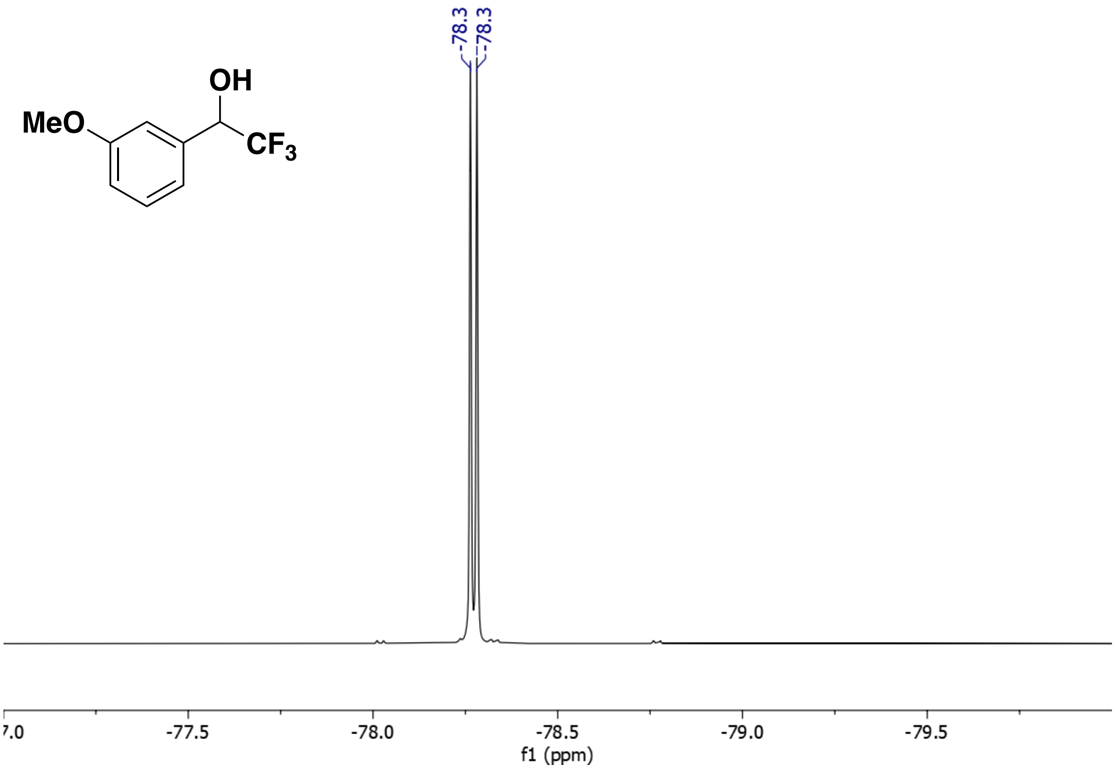

3k ^{1}H NMR (400 MHz, $CDCl_3$)

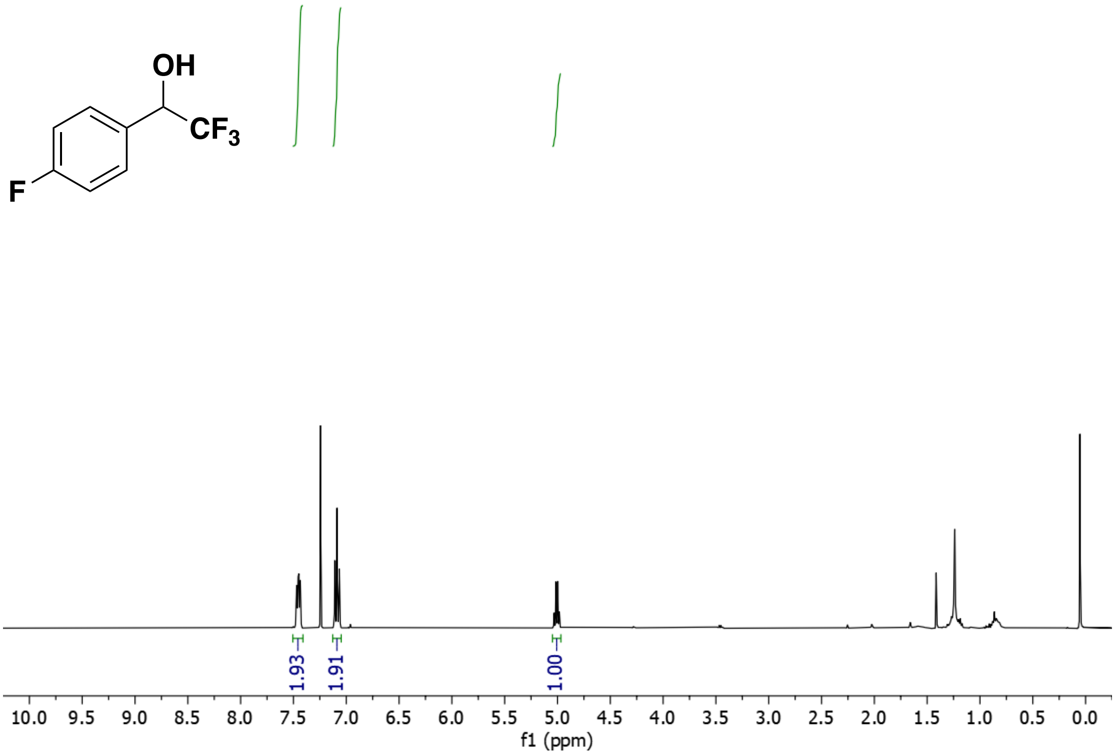

3k ^{13}C NMR (100 MHz, $CDCl_3$)

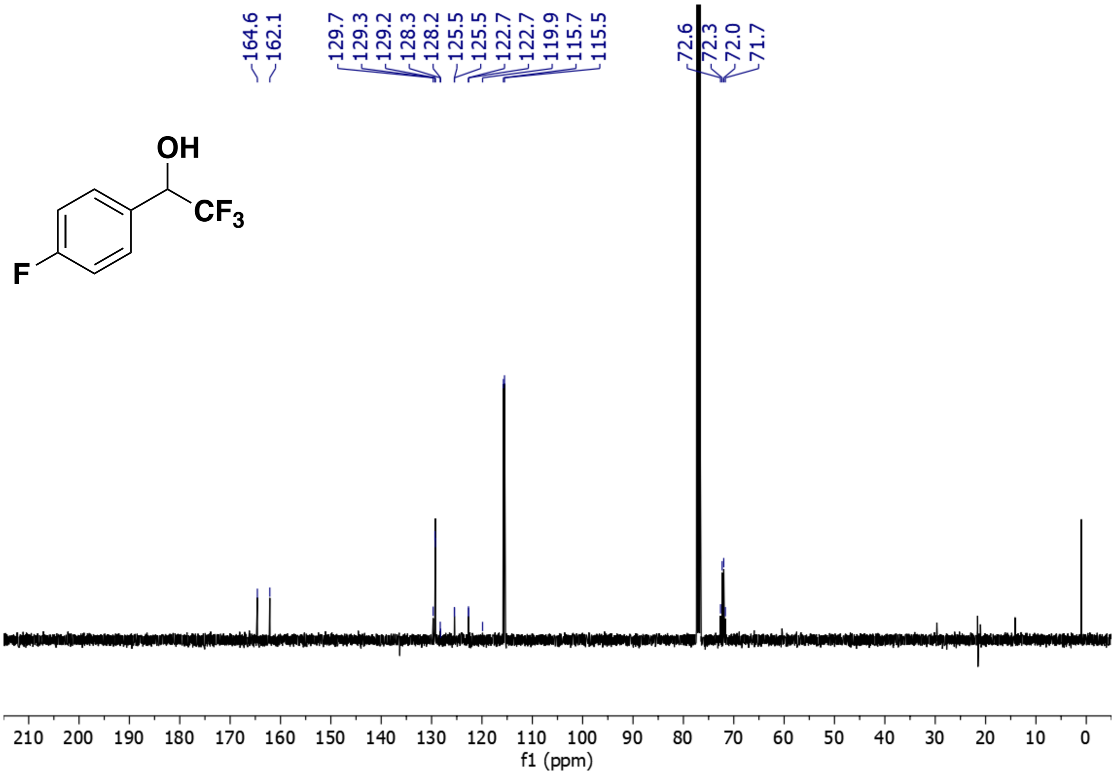

3k ^{19}F NMR (377 MHz, $CDCl_3$)

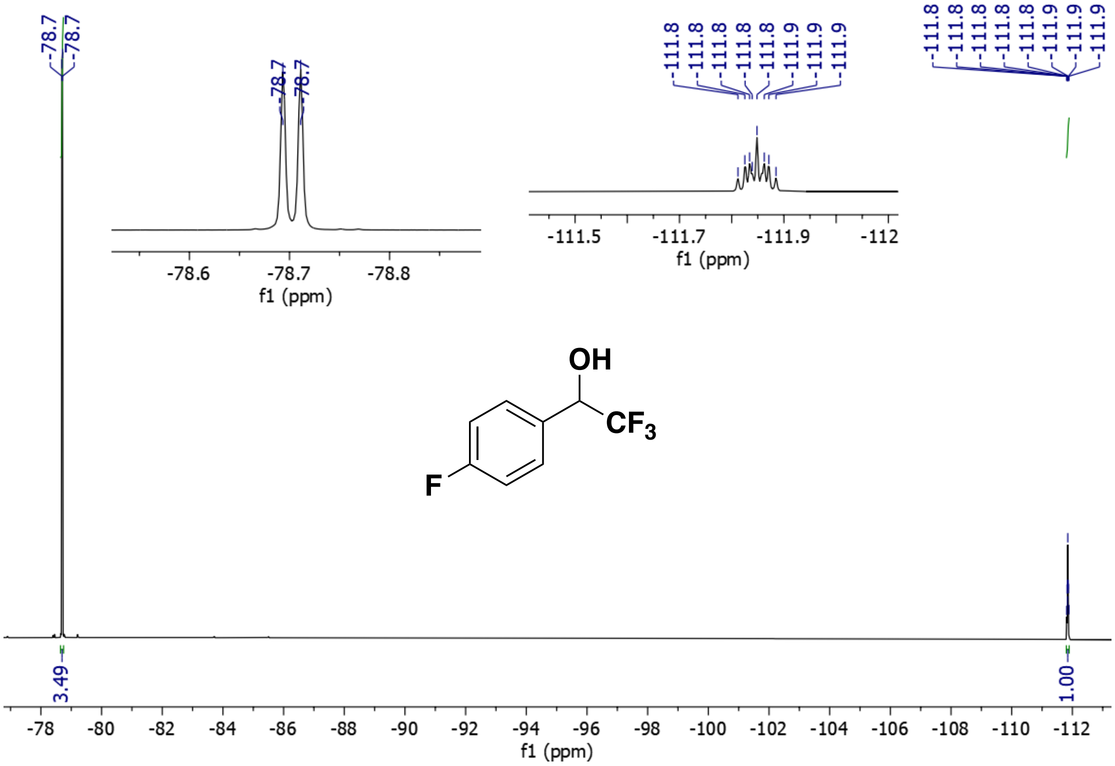

3l ^{1}H NMR (400 MHz, $CDCl_3$)

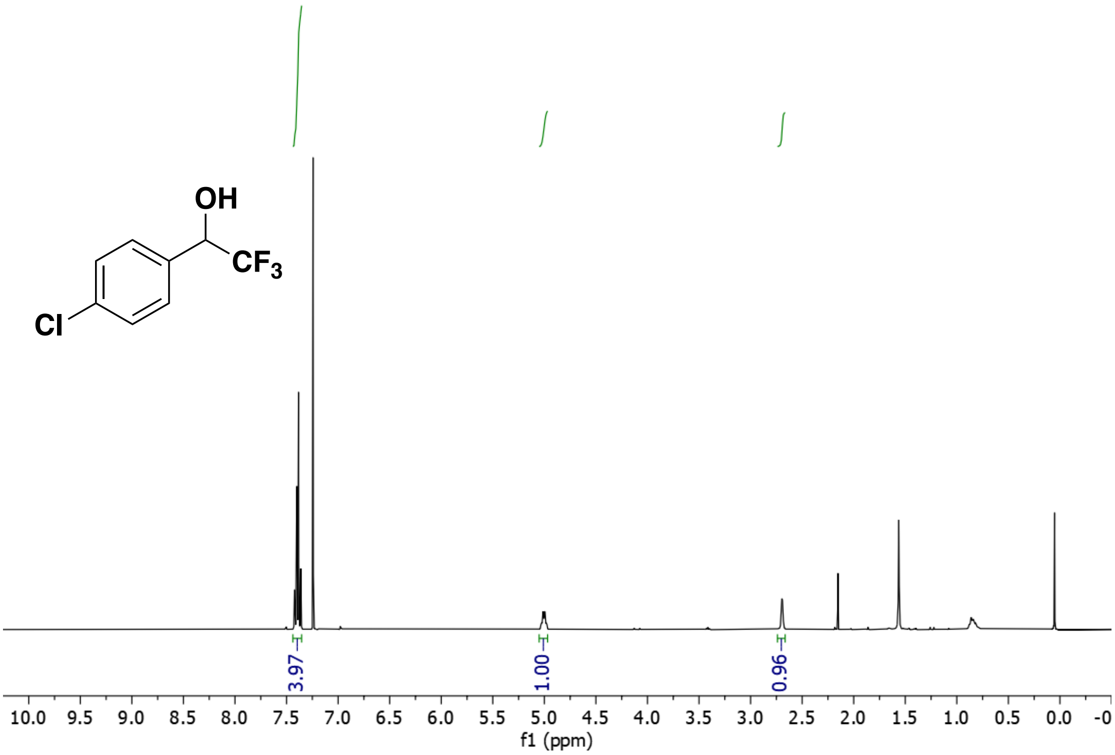

3l ^{13}C NMR (100 MHz, $CDCl_3$)

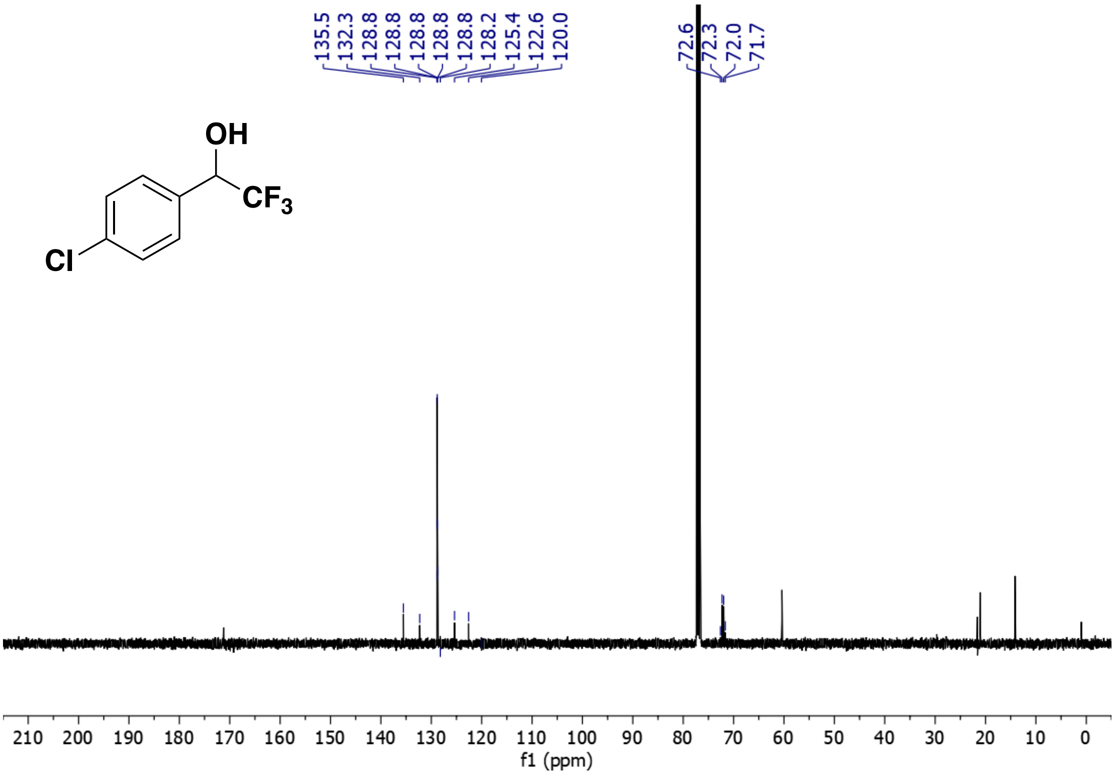

3l ^{19}F NMR (377 MHz, $CDCl_3$)

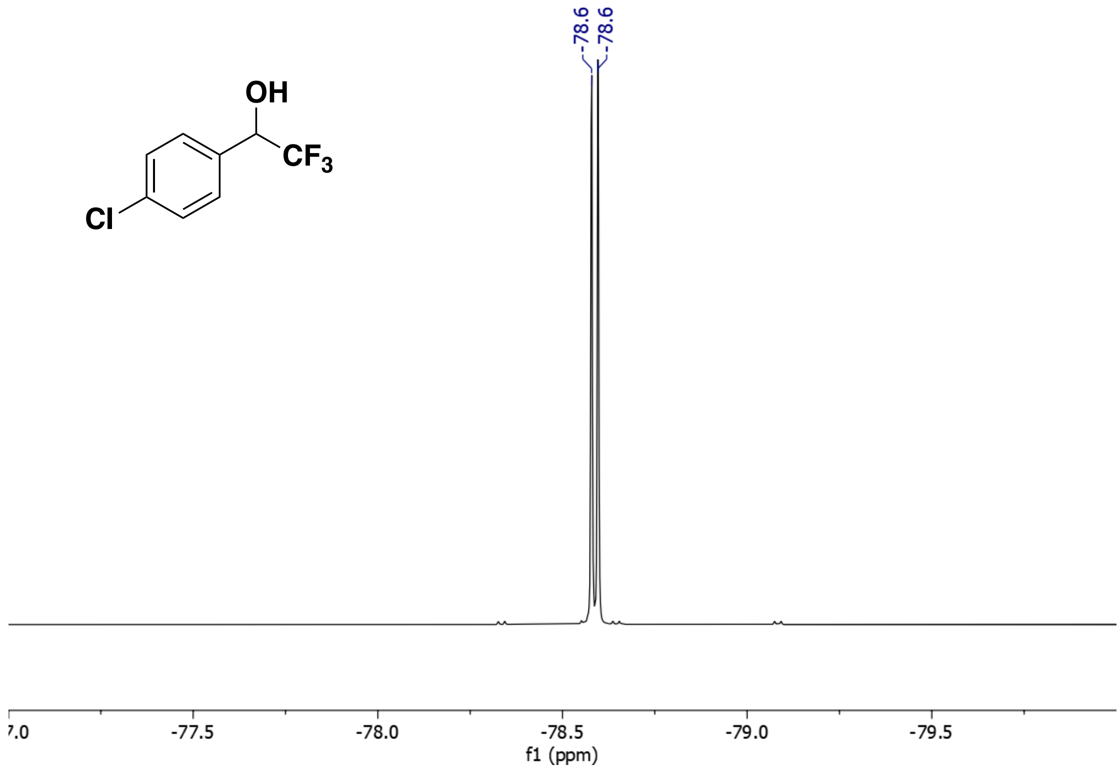

3m ^{1}H NMR (600 MHz, $CDCl_3$)

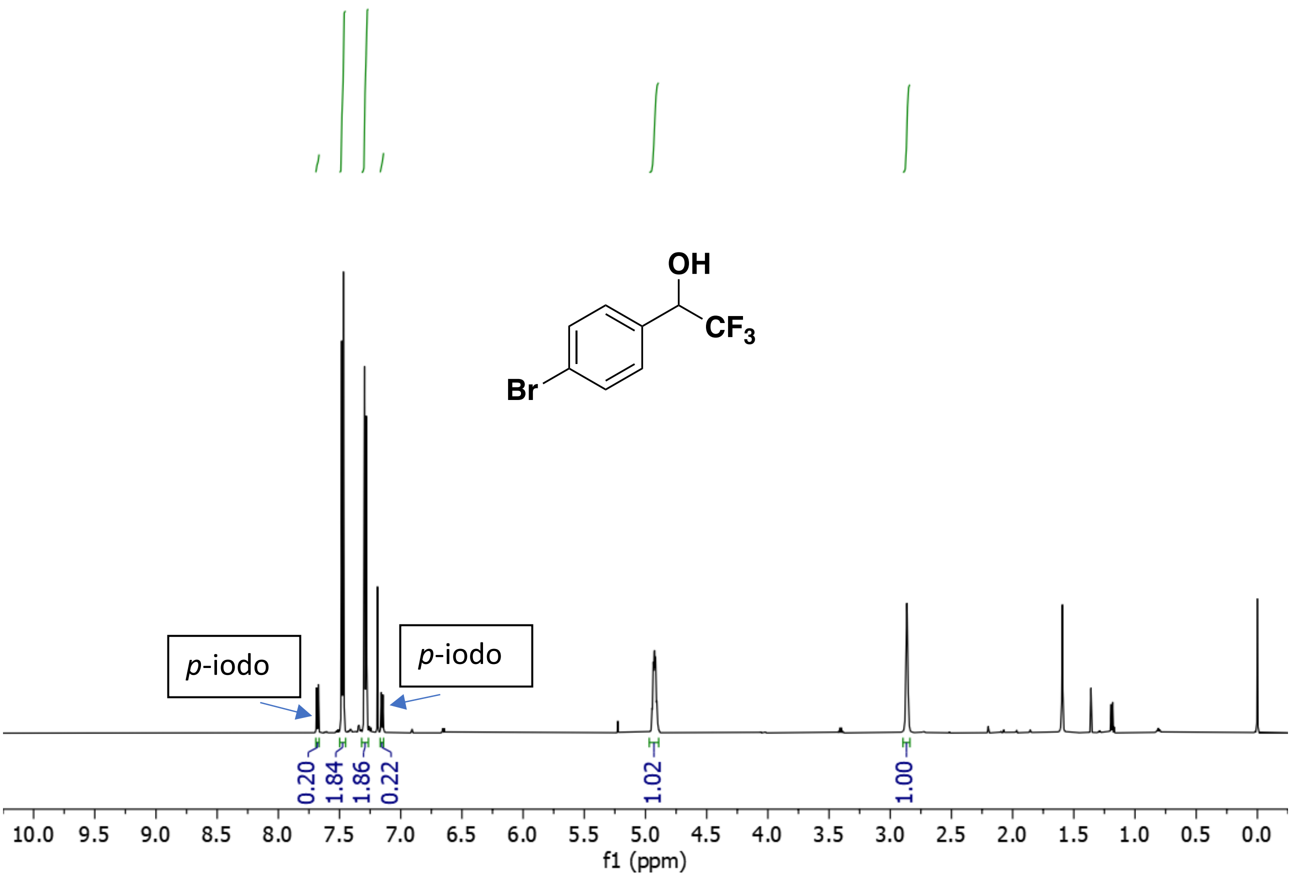

3m ^{13}C NMR (150 MHz, $CDCl_3$)

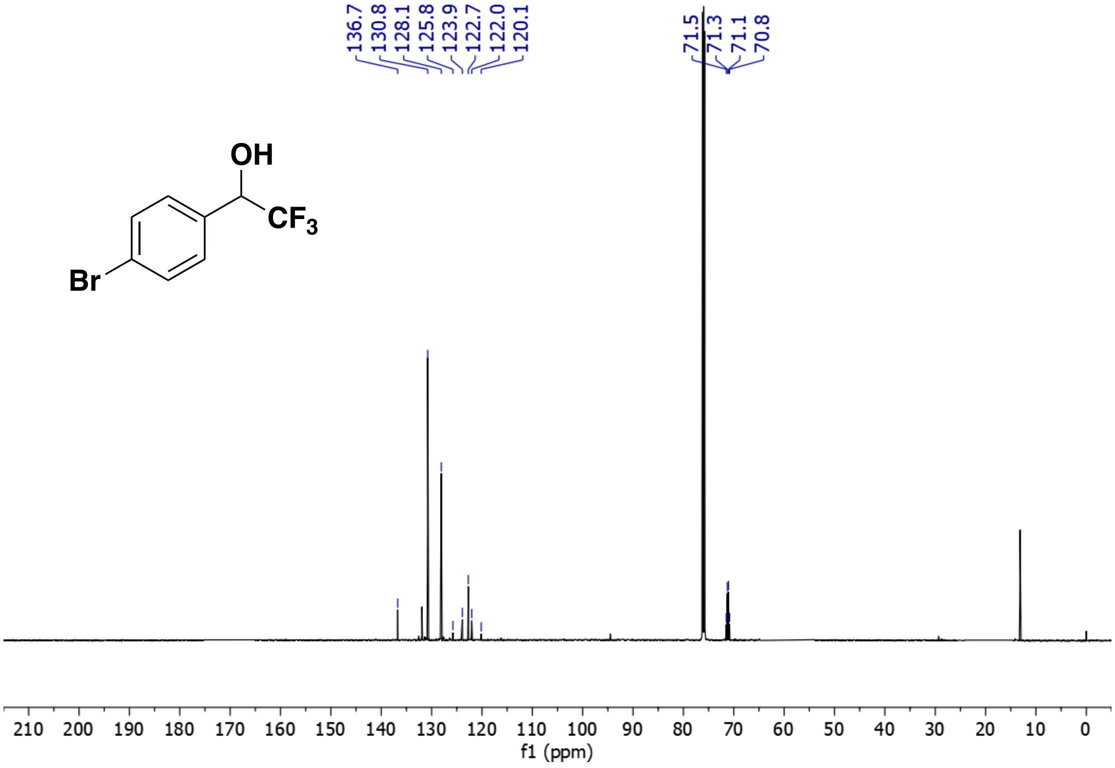

3m ^{19}F NMR (565 MHz, CDCl$_3$)

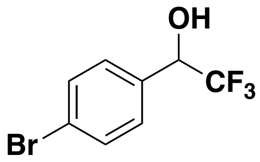

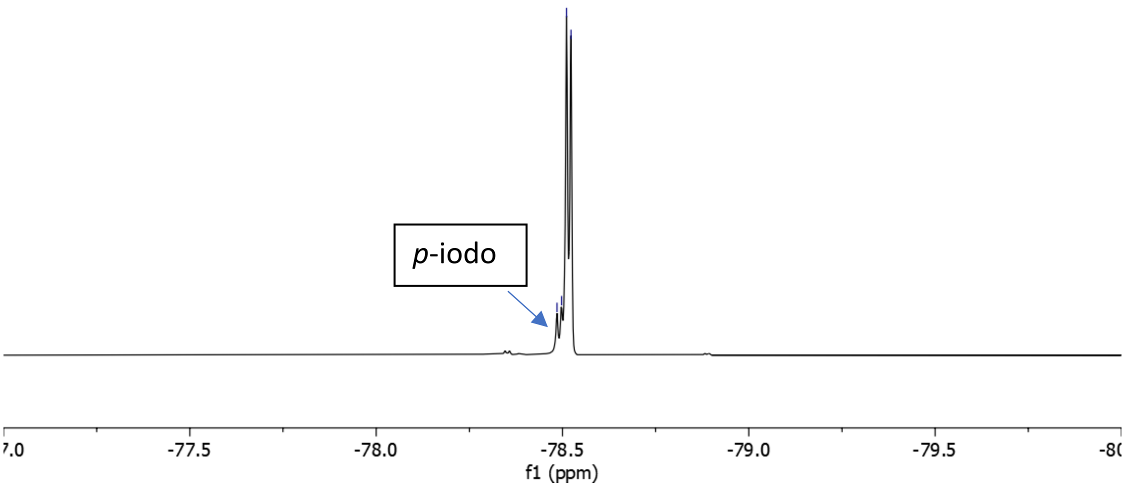

3n ^{1}H NMR (400 MHz, $CDCl_3$)

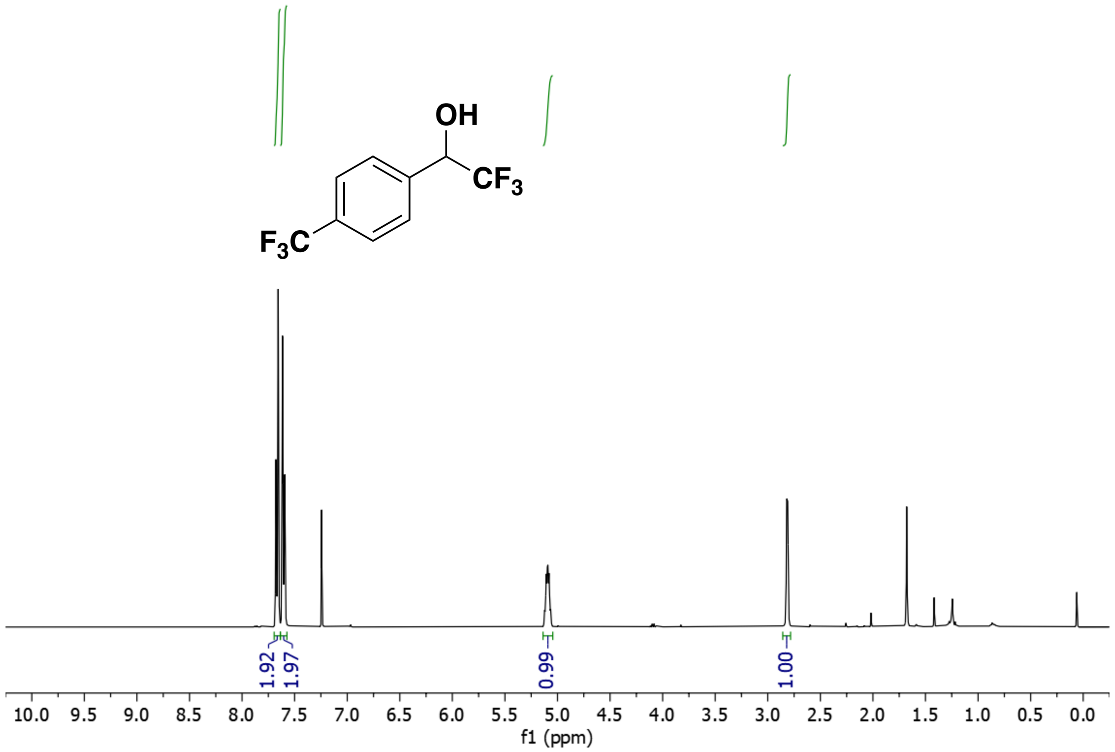

3n ^{13}C NMR (100 MHz, $CDCl_3$)

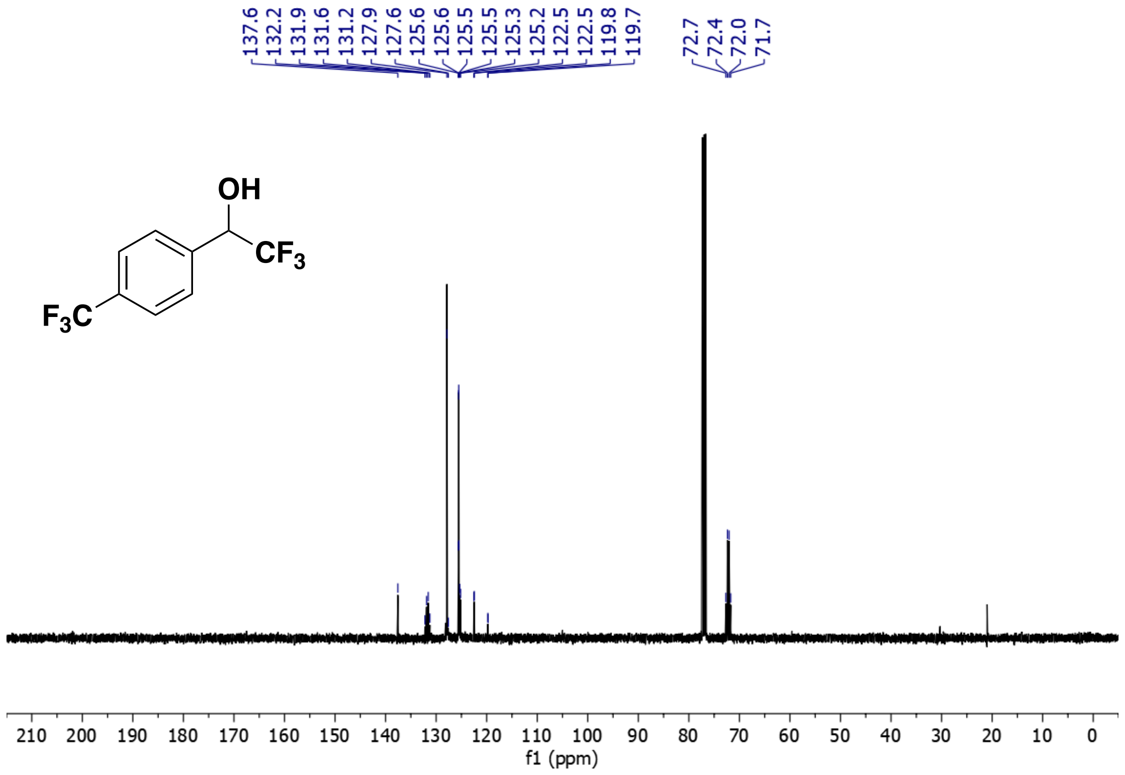

3n ^{19}F NMR (377 MHz, $CDCl_3$)

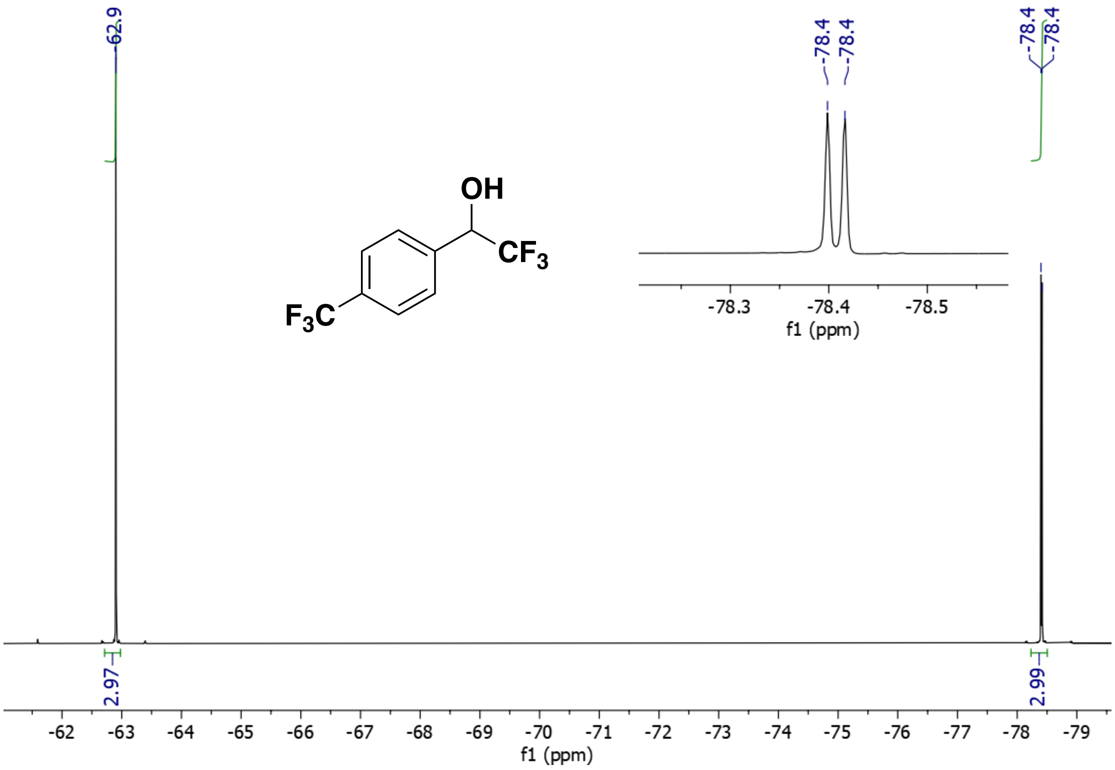

3o ^{1}H NMR (400 MHz, $CDCl_3$)

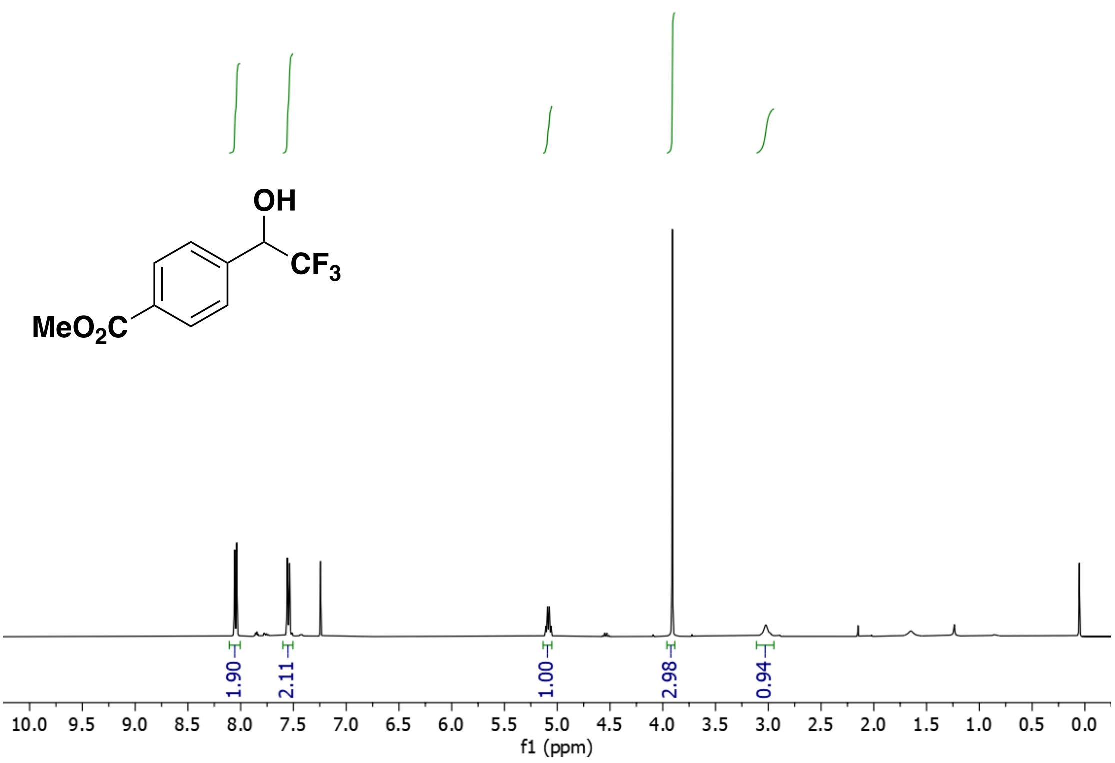

3o ^{13}C NMR (100 MHz, $CDCl_3$)

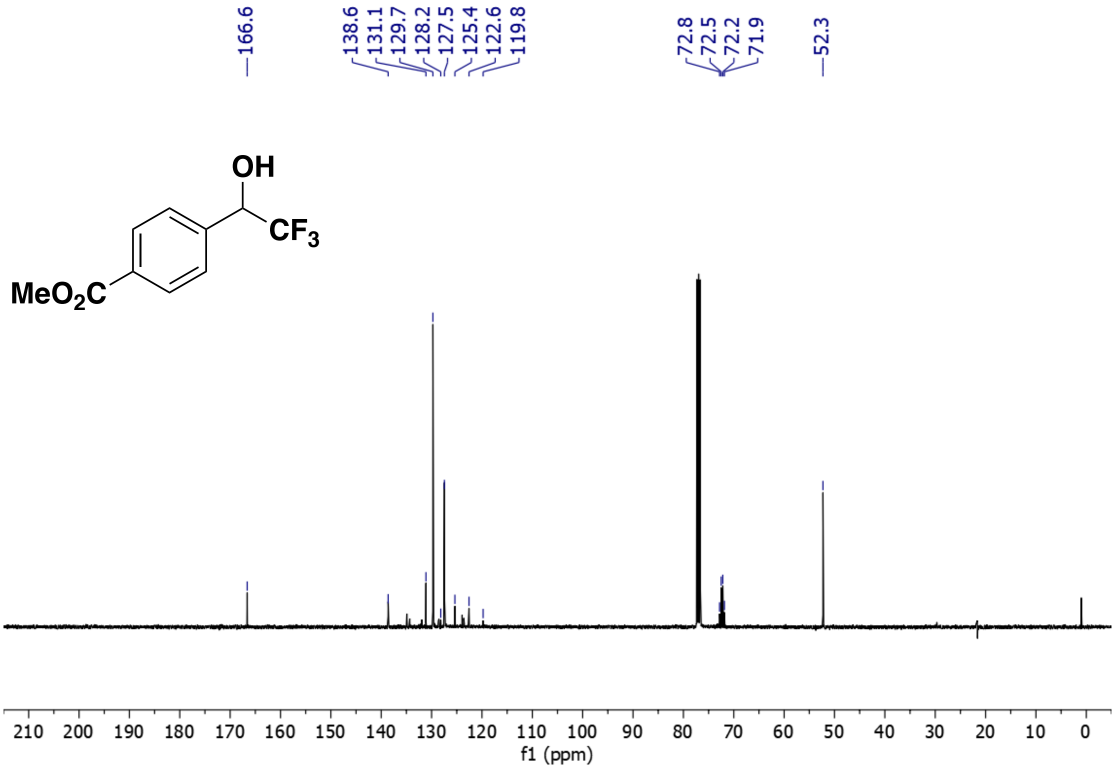

3o ^{19}F NMR (377 MHz, $CDCl_3$)

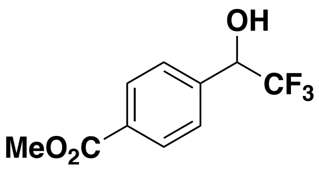

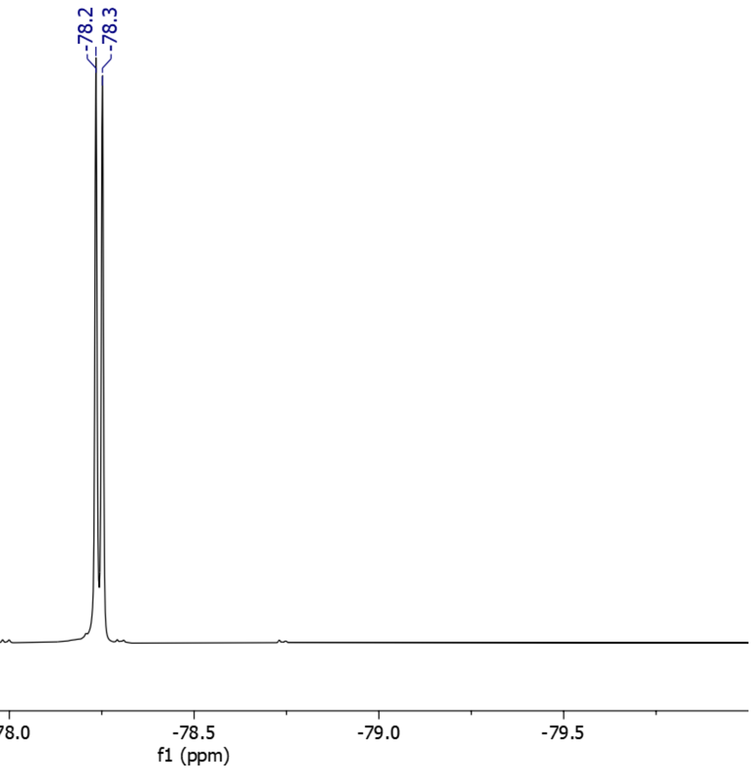

3p ^{1}H NMR (400 MHz, $CDCl_3$)

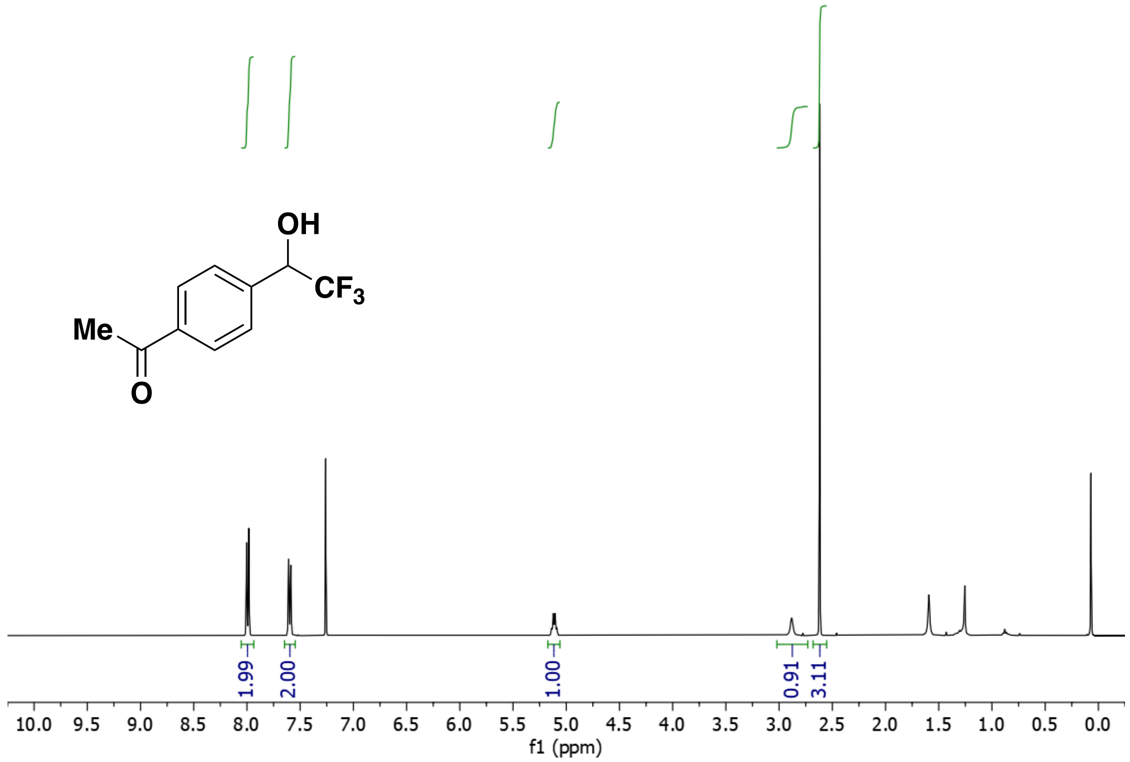

3p ^{13}C NMR (100 MHz, $CDCl_3$)

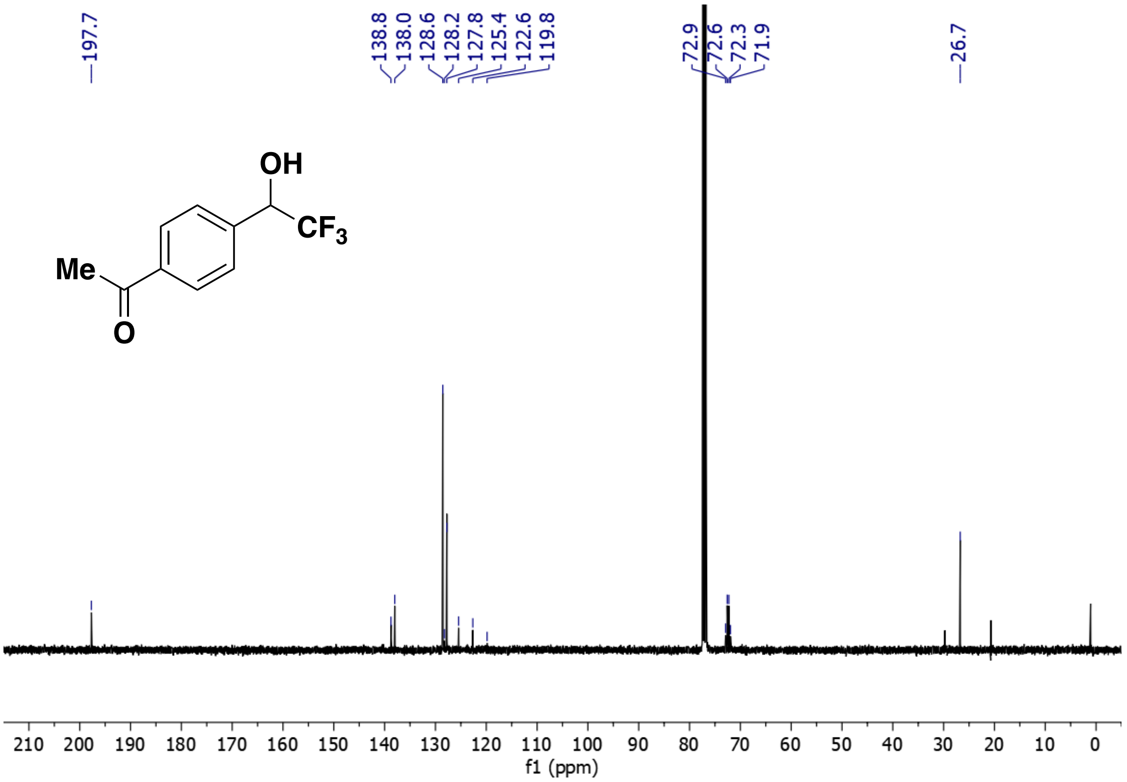

3p ^{19}F NMR (377 MHz, $CDCl_3$)

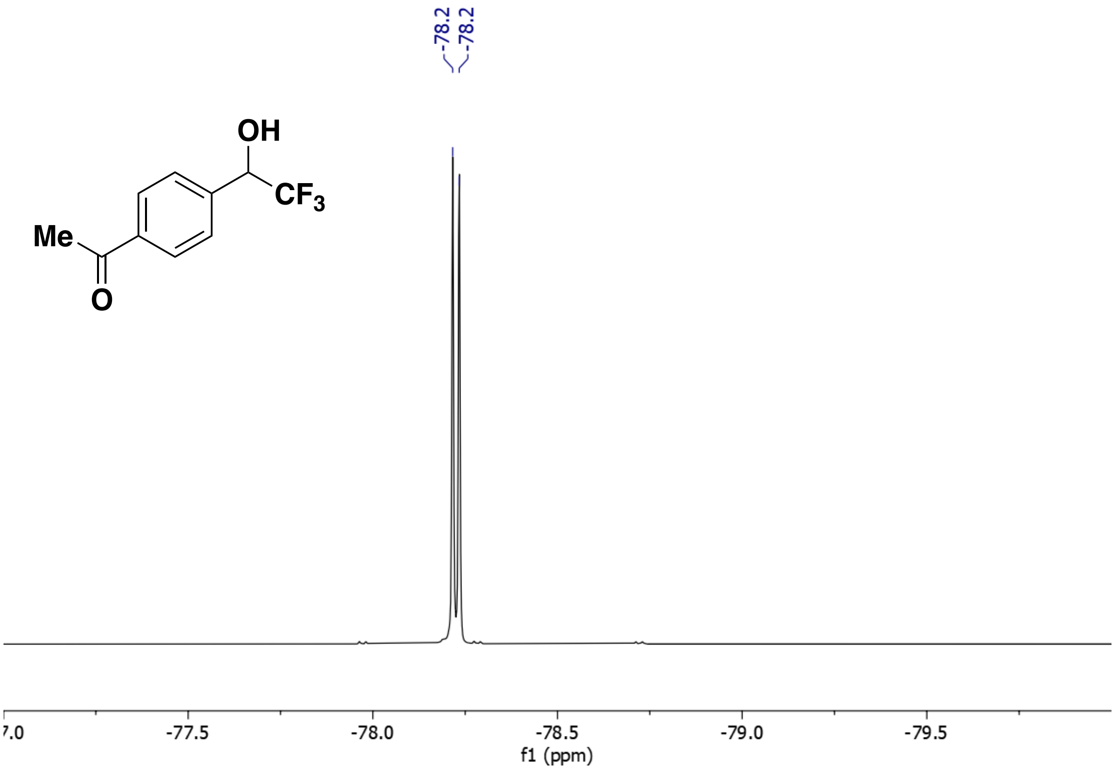

3q ^{1}H NMR (400 MHz, CDCl$_3$)

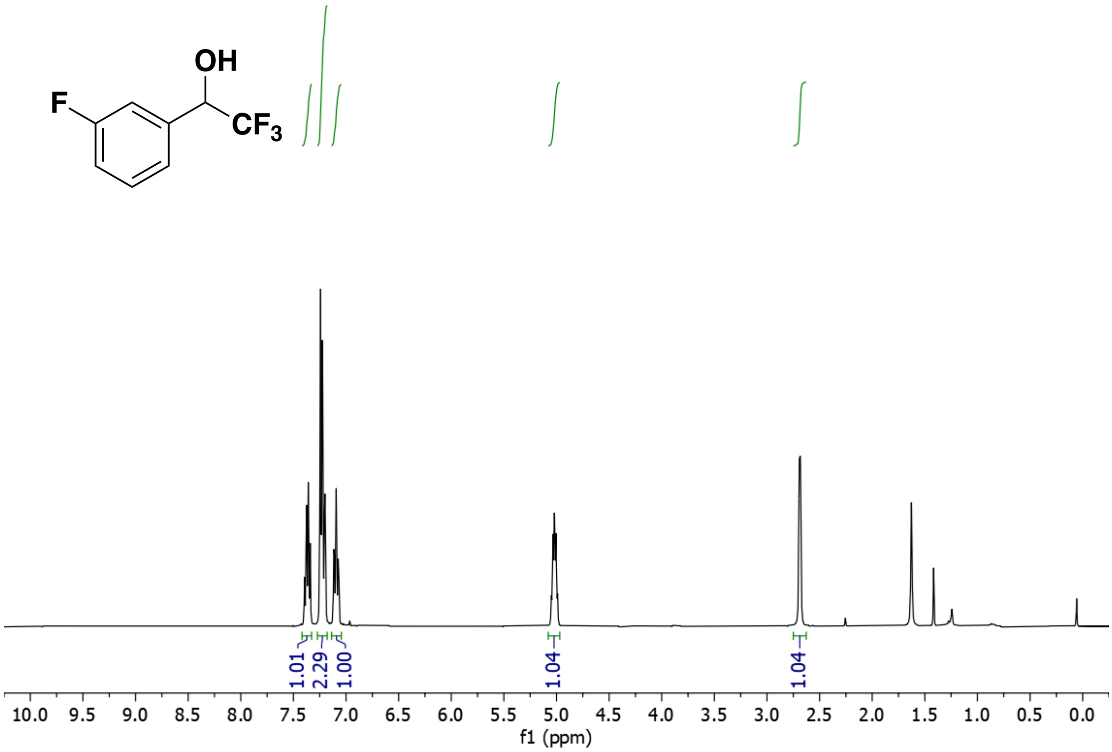

3q ^{13}C NMR (100 MHz, CDCl$_3$)

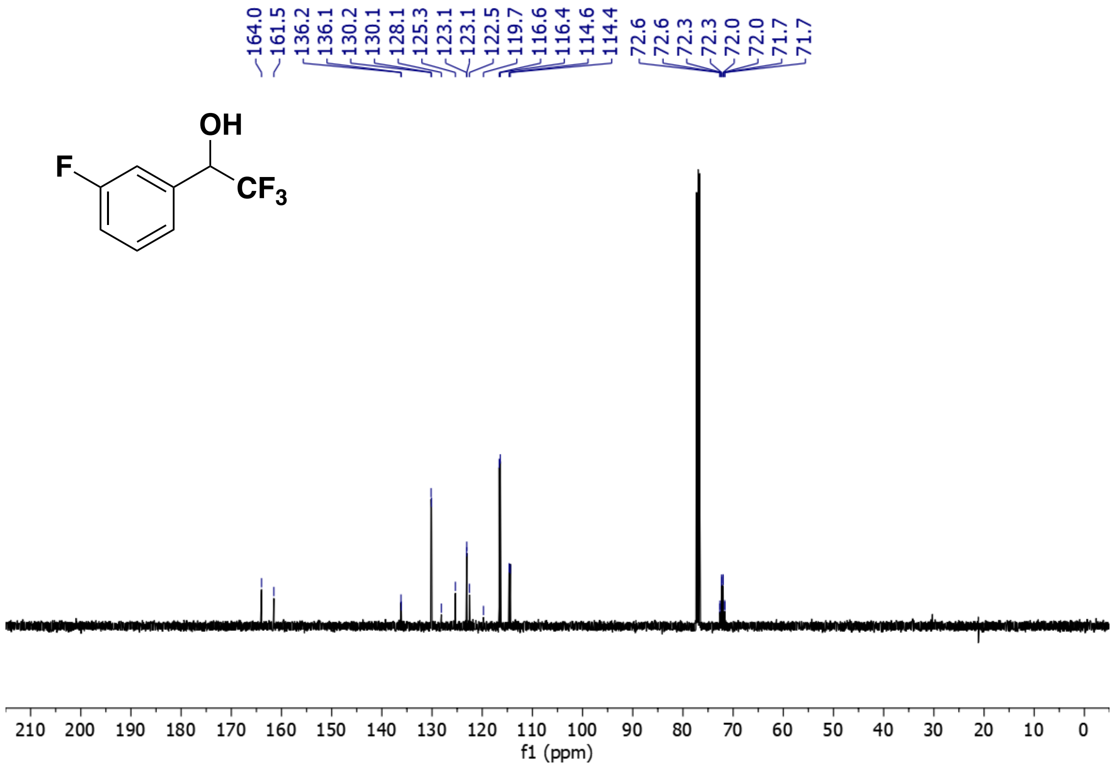

3q ^{19}F NMR (377 MHz, $CDCl_3$)

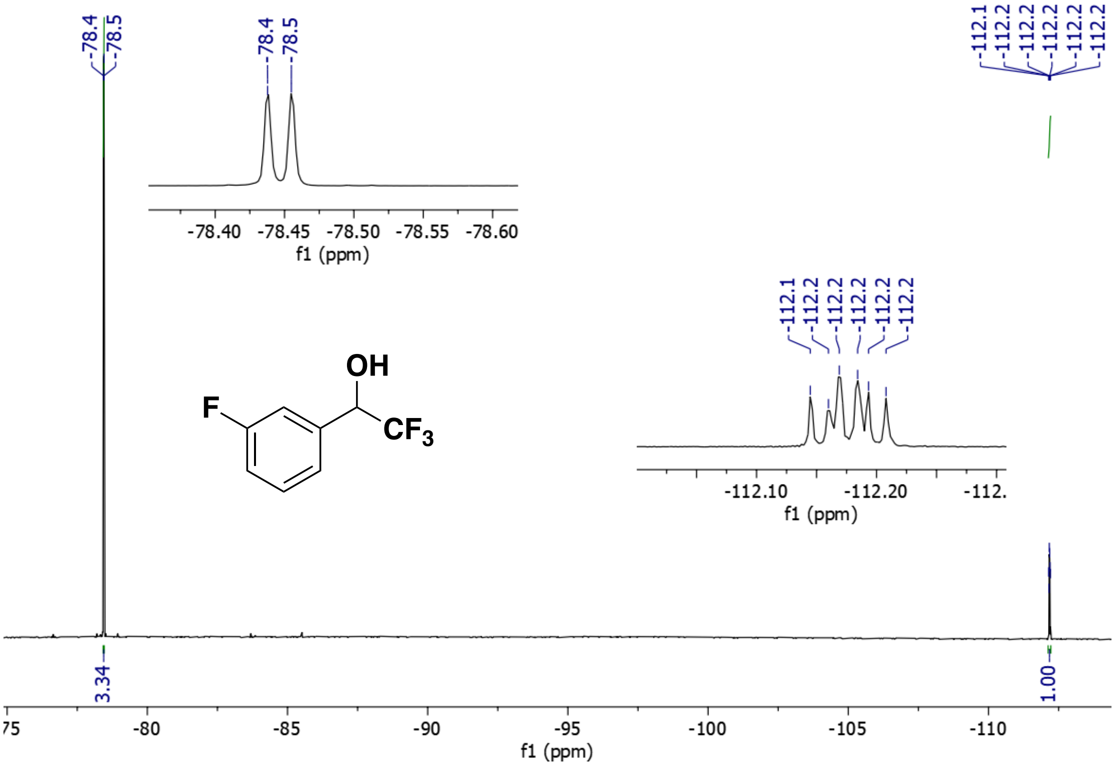

3r ^{1}H NMR (400 MHz, $CDCl_3$)

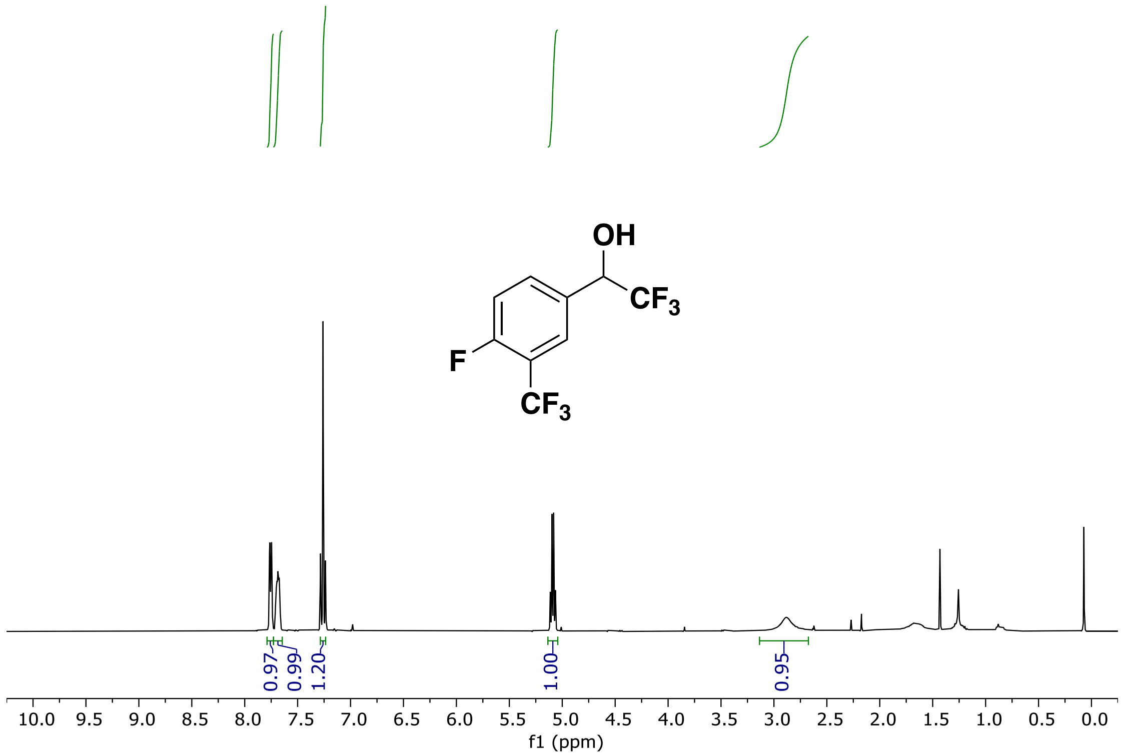

3r ^{13}C NMR (150 MHz, $CDCl_3$)

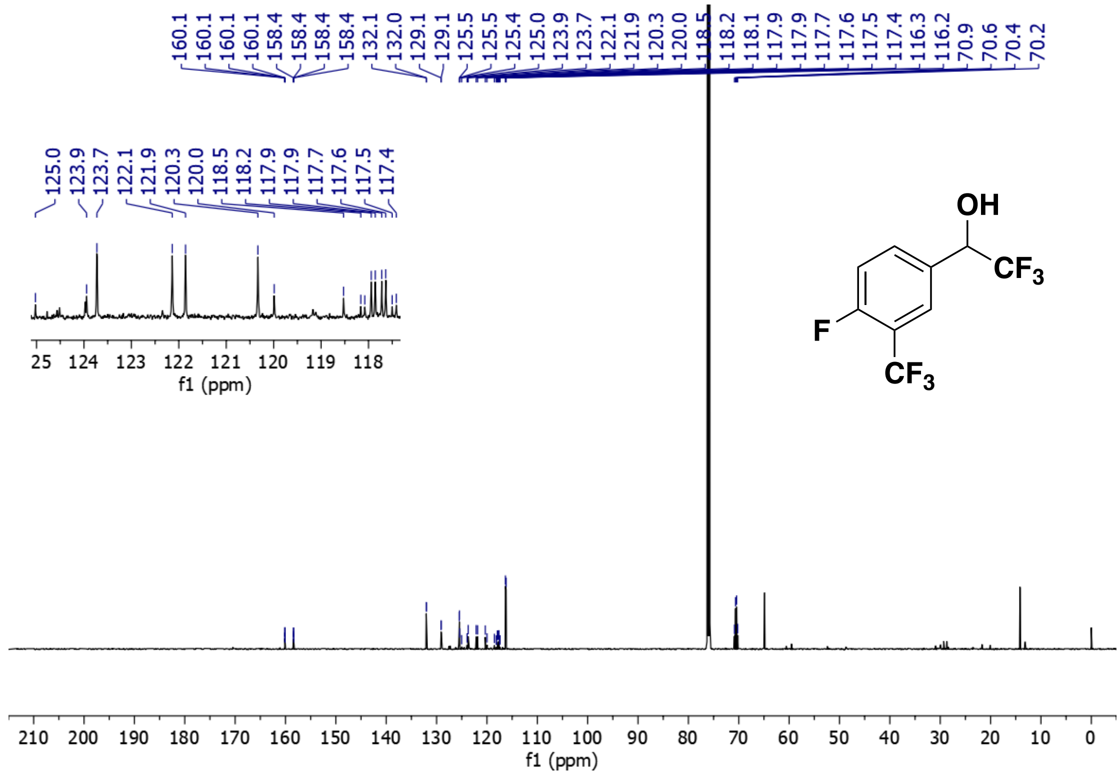

3r ^{19}F NMR (377 MHz, $CDCl_3$)

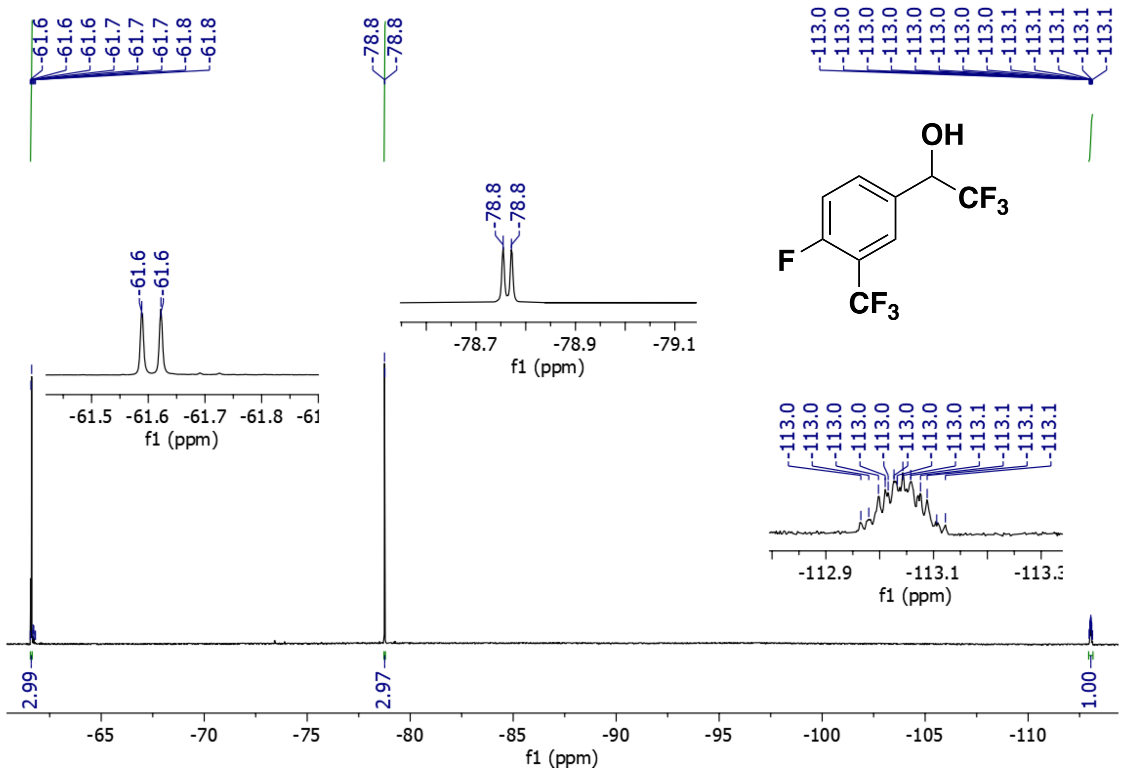

3s ^{1}H NMR (600 MHz, $CDCl_3$)

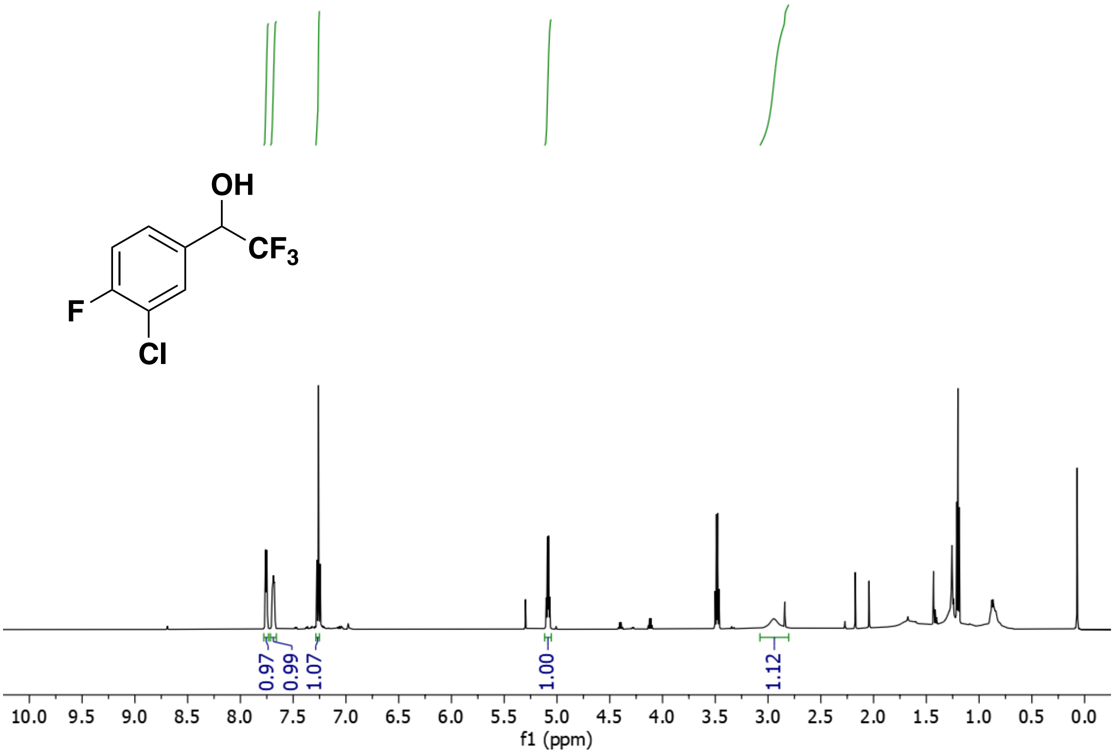

3s ^{13}C NMR (150 MHz, $CDCl_3$)

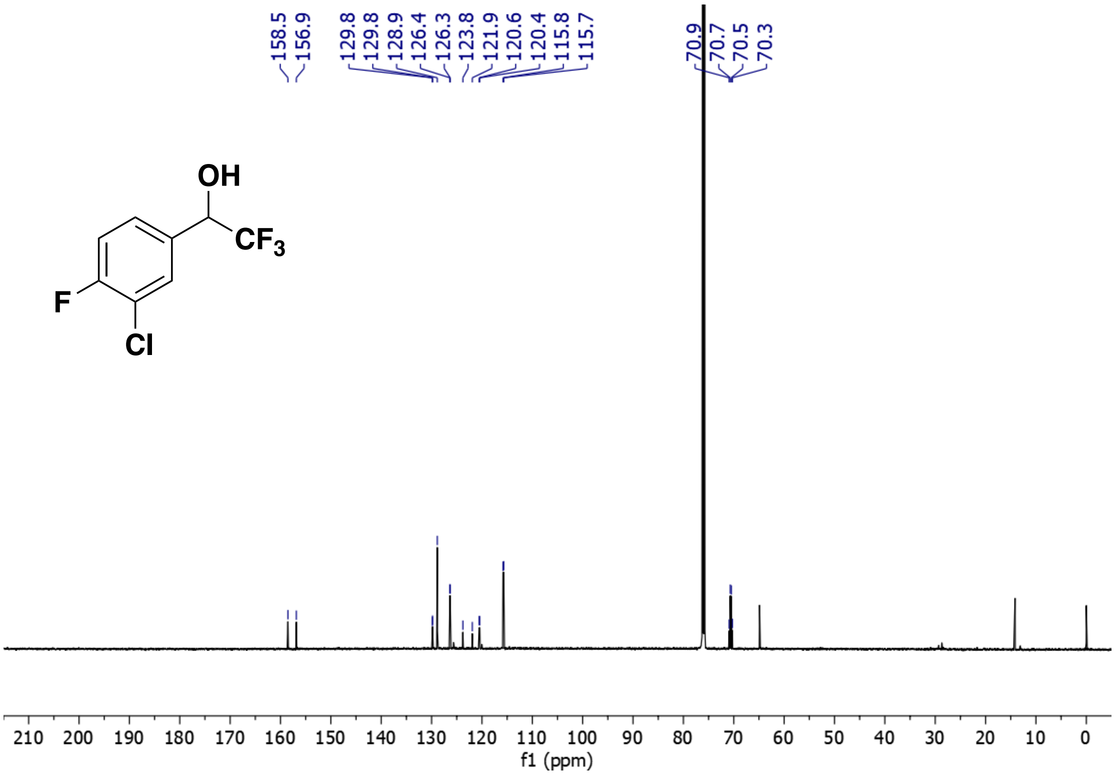

3s ^{19}F NMR (565 MHz, $CDCl_3$)

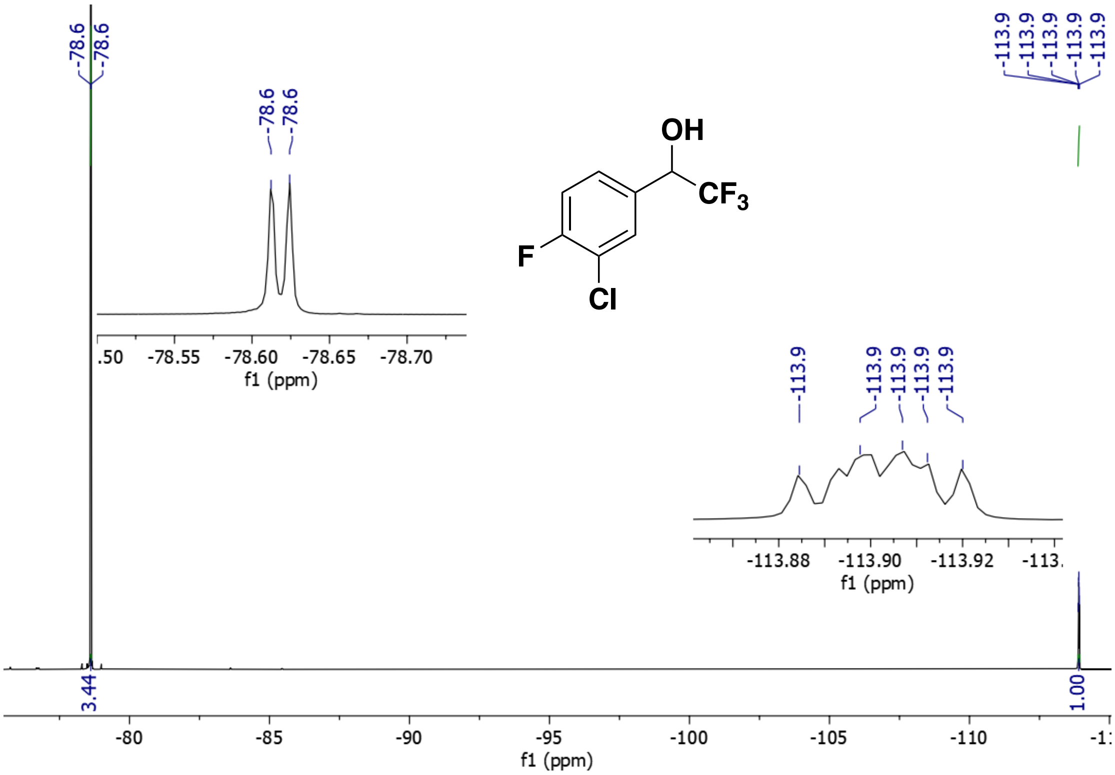

3u ^{1}H NMR (400 MHz, CDCl$_3$)

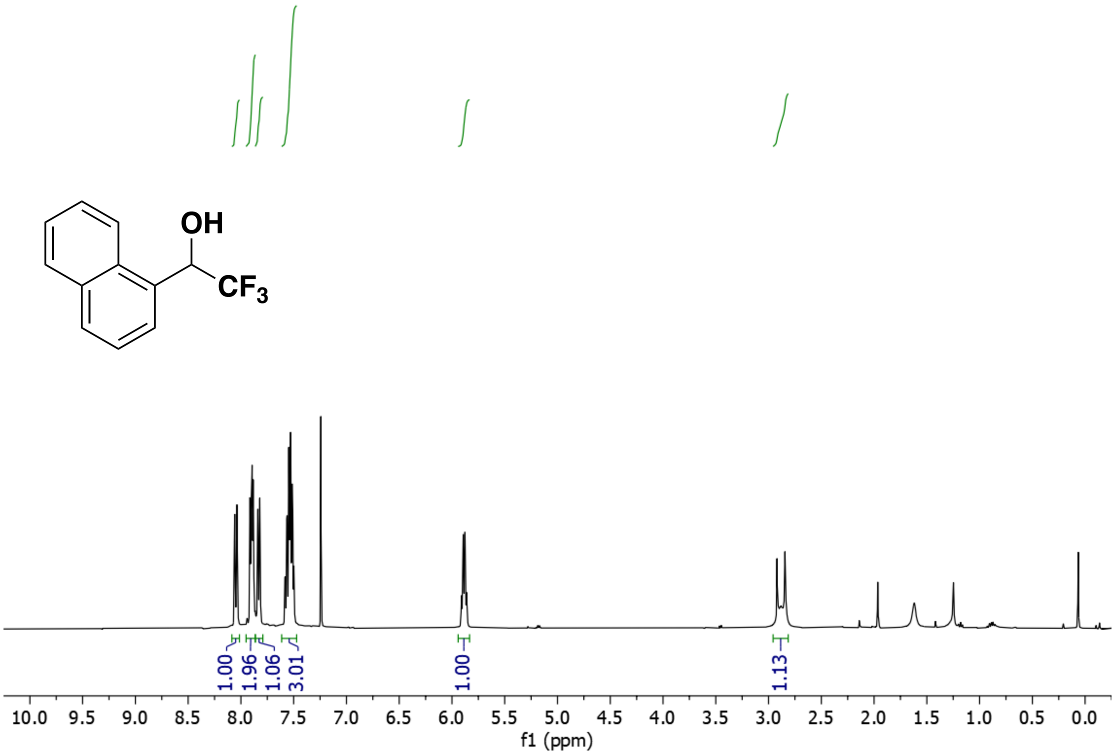

3u ^{13}C NMR (100 MHz, CDCl$_3$)

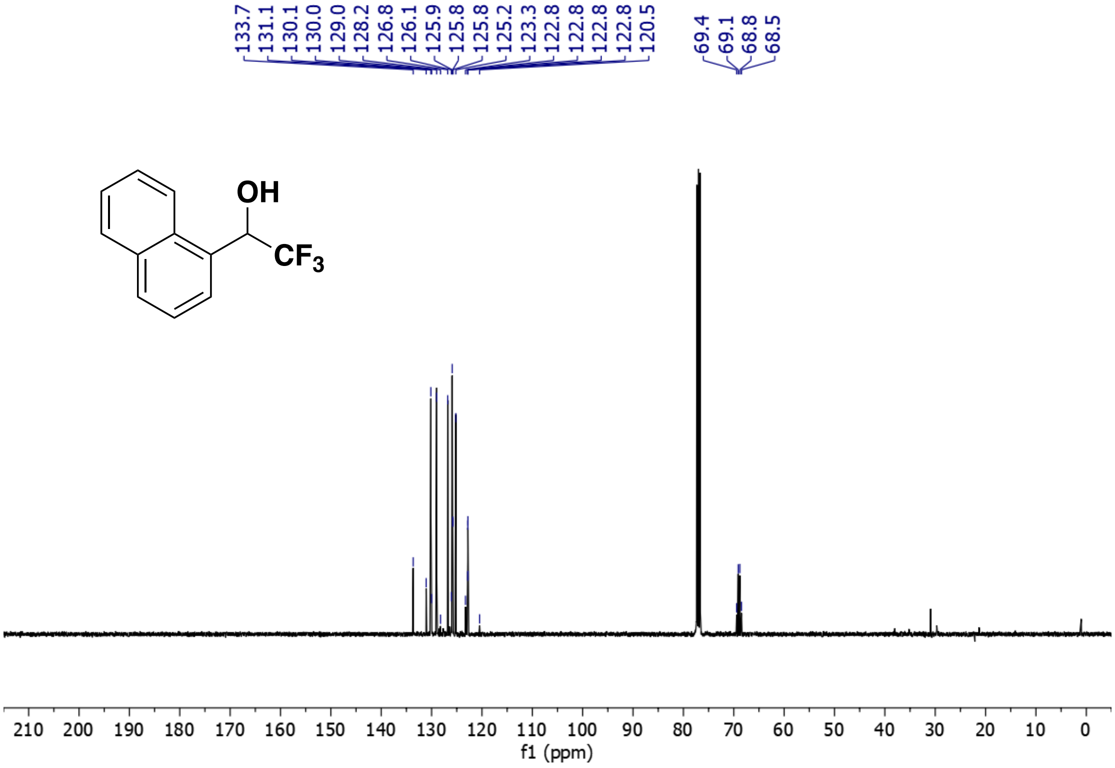

3u ^{19}F NMR (377 MHz, $CDCl_3$)

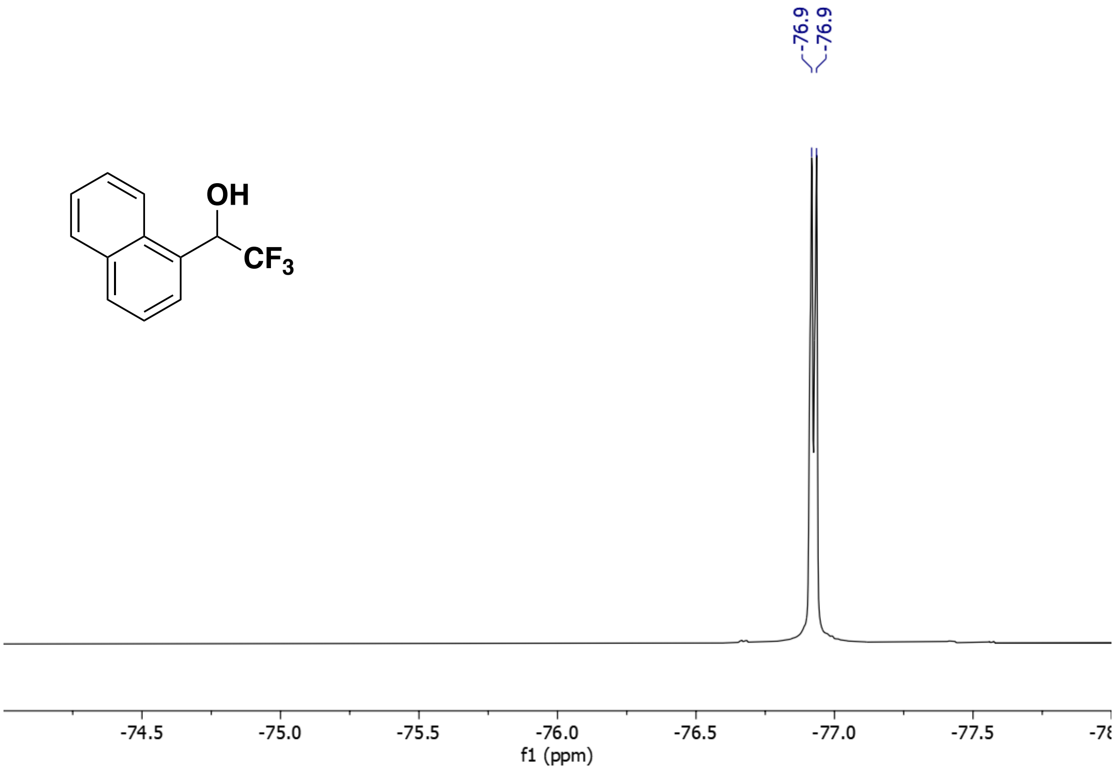

3v ^{1}H NMR (400 MHz, CDCl$_3$)

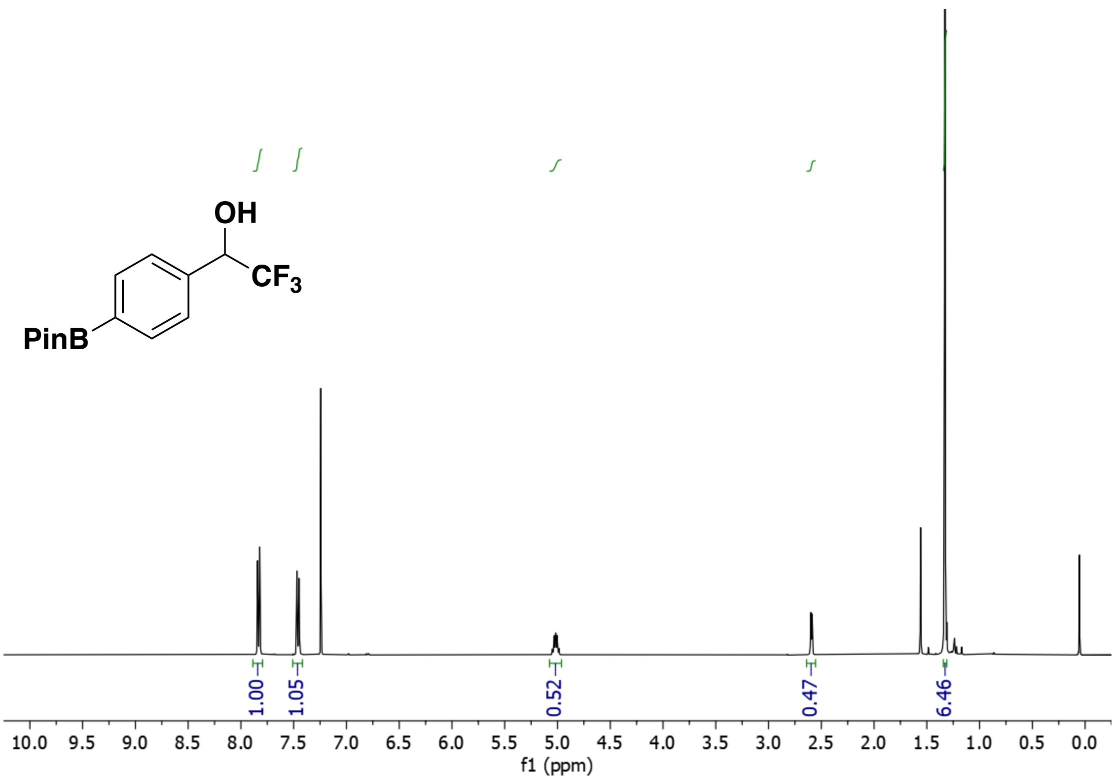

3v ^{13}C NMR (100 MHz, CDCl$_3$)

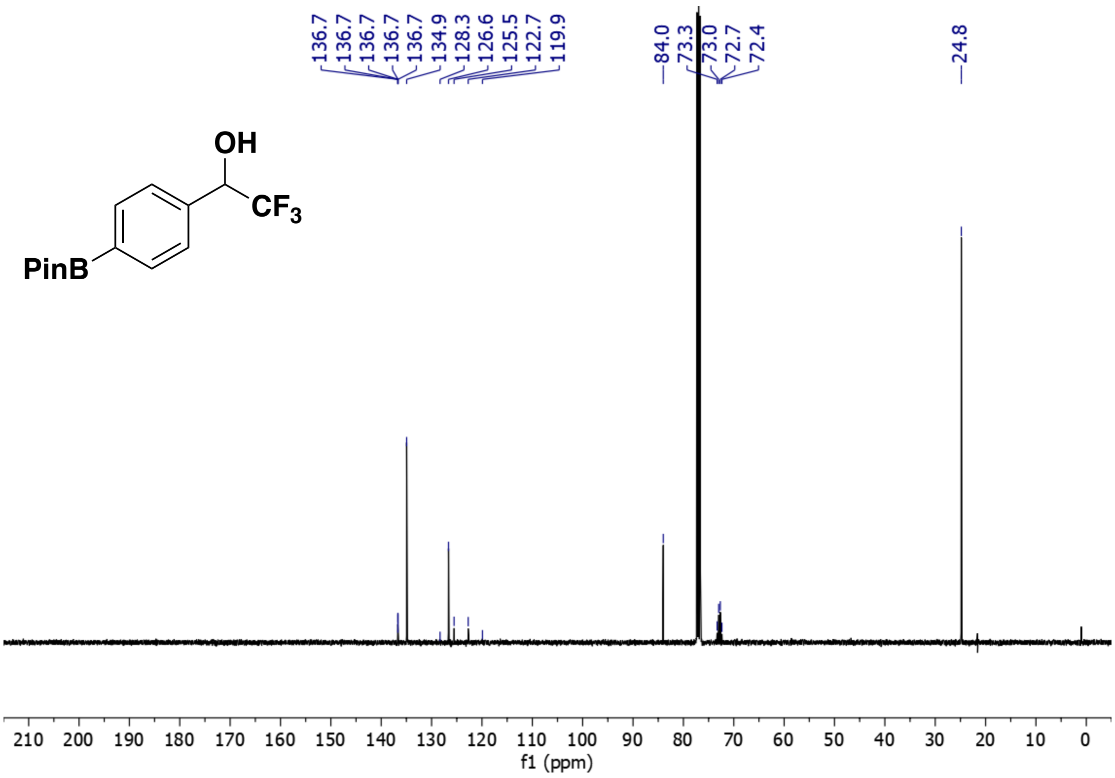

3v ^{19}F NMR (377 MHz, CDCl$_3$)

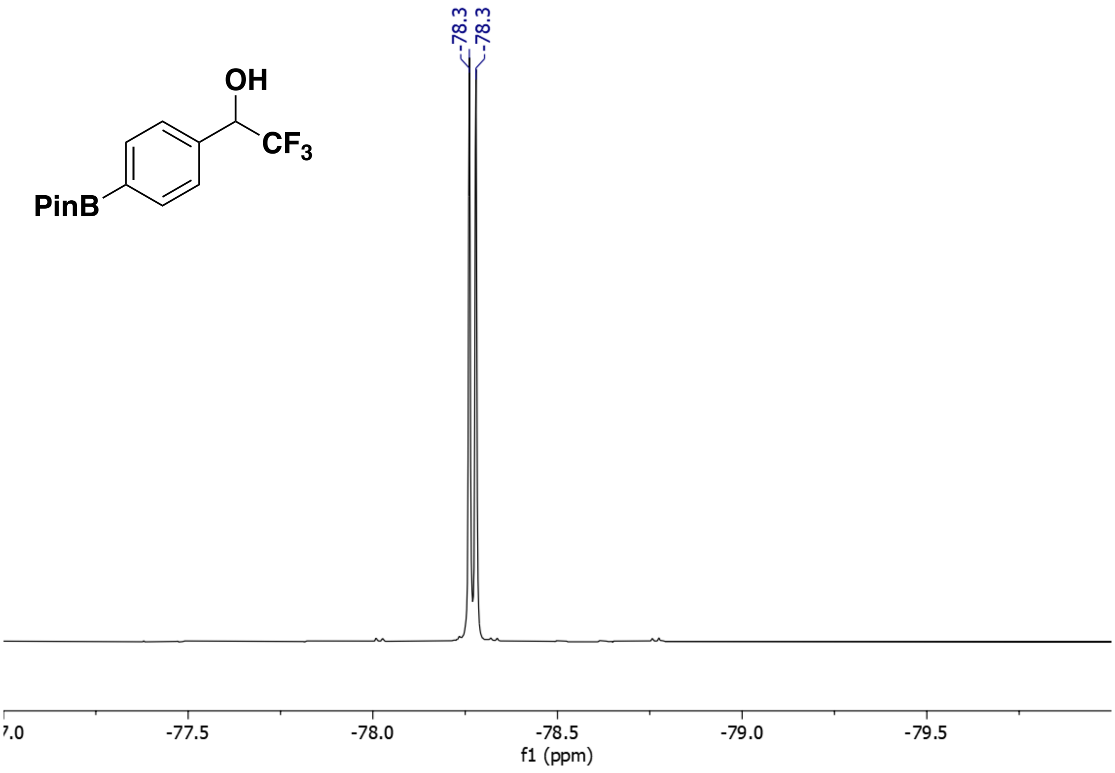

3w ^1H NMR (400 MHz, $CDCl_3$)

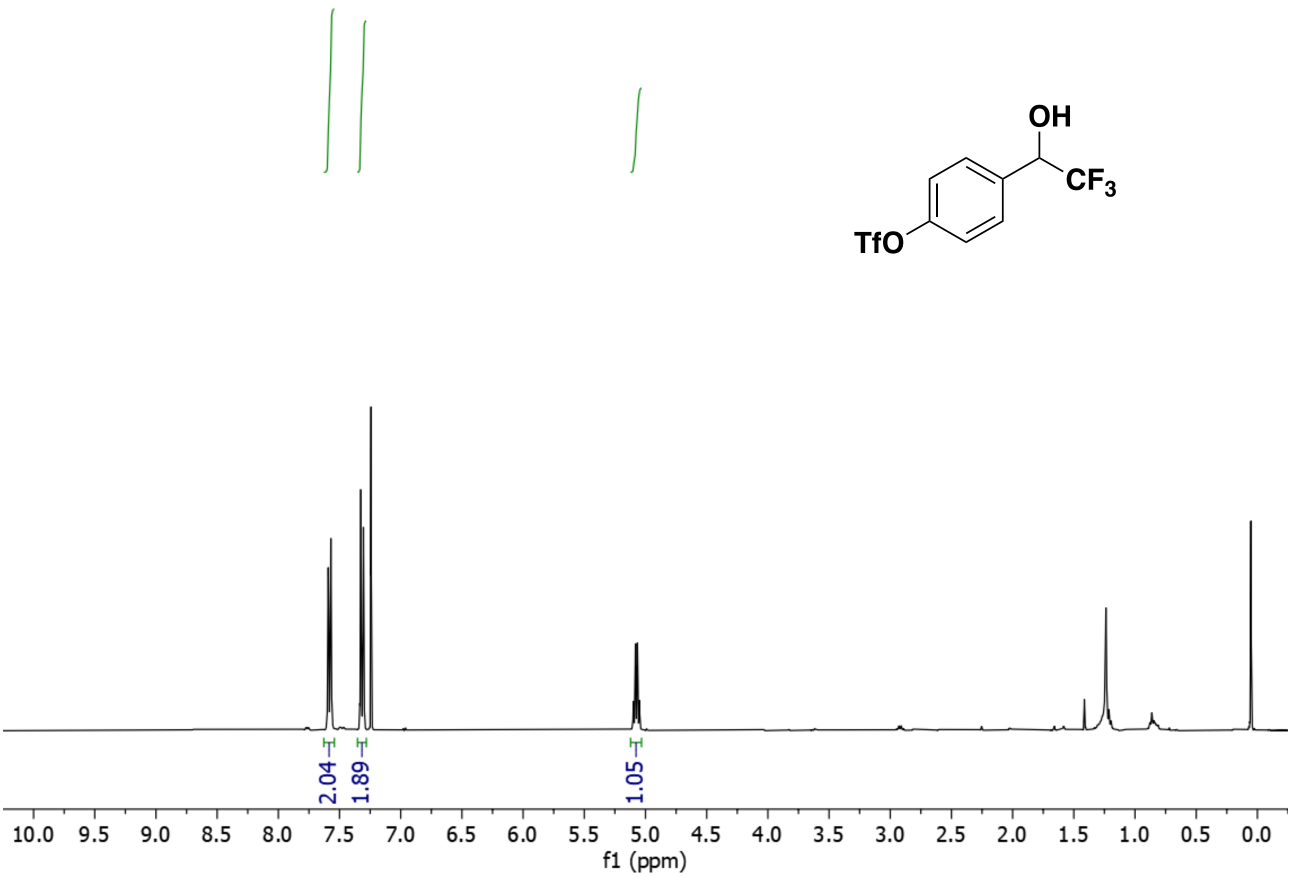

3w ^{13}C NMR (100 MHz, $CDCl_3$)

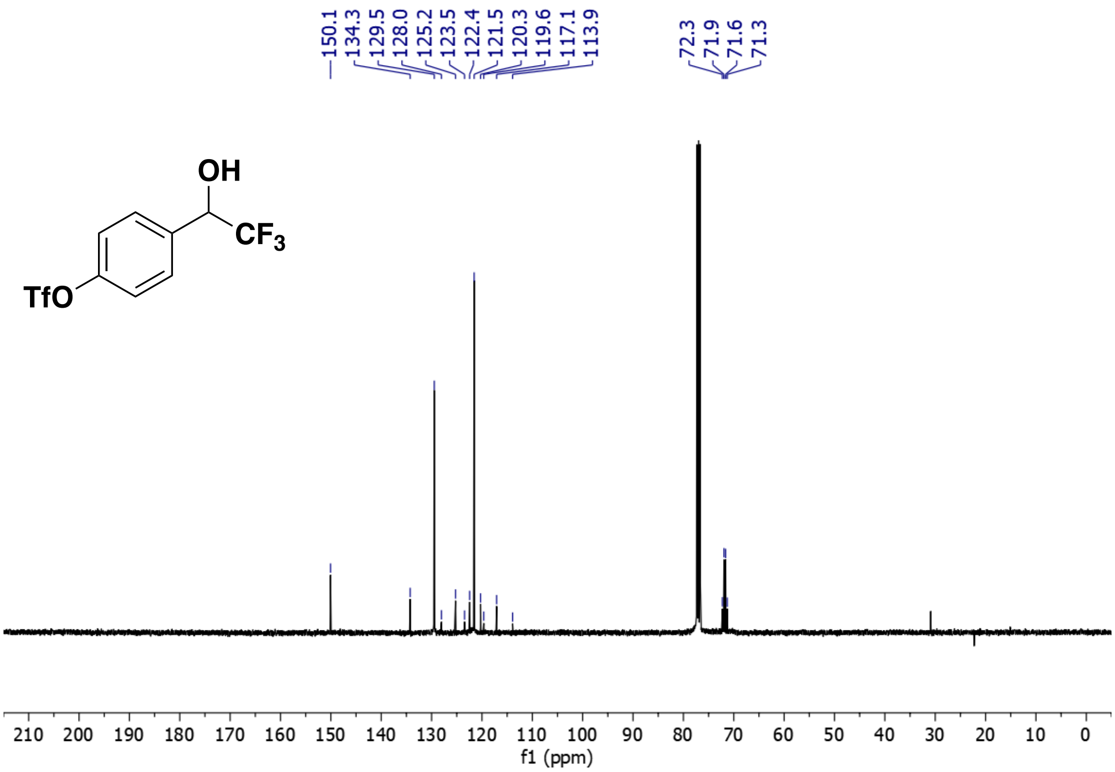

3w ^{19}F NMR (377 MHz, $CDCl_3$)

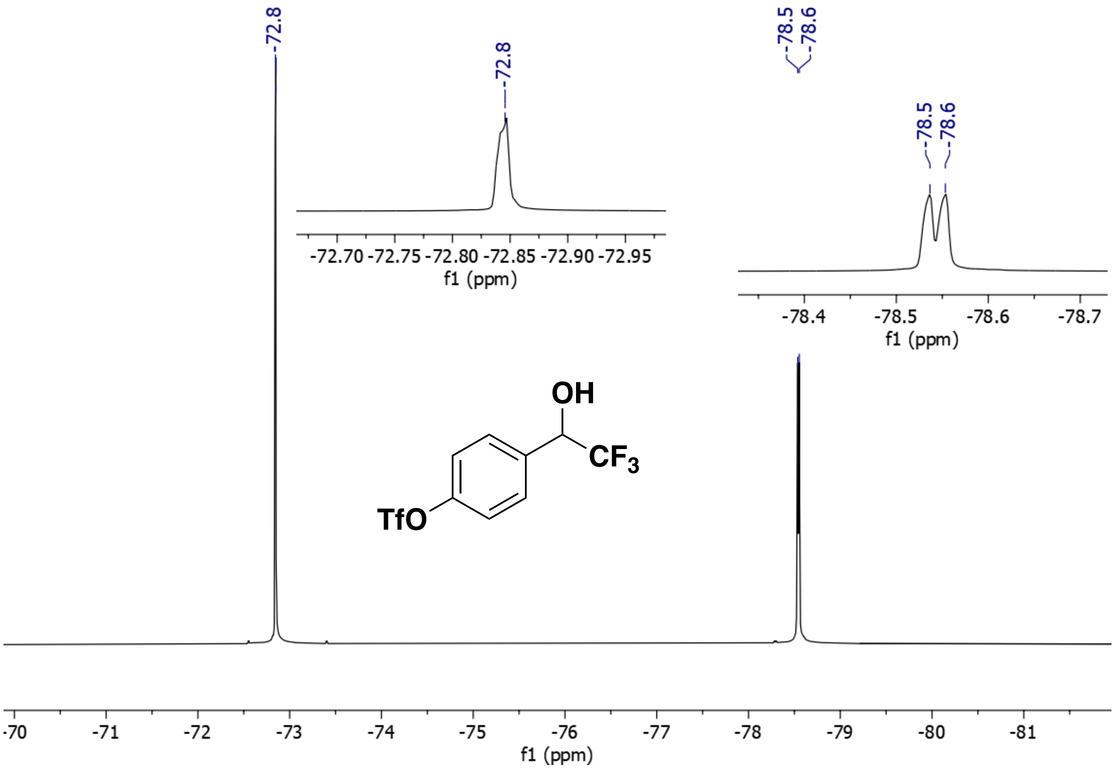

3x ^{1}H NMR (400 MHz, $CDCl_3$)

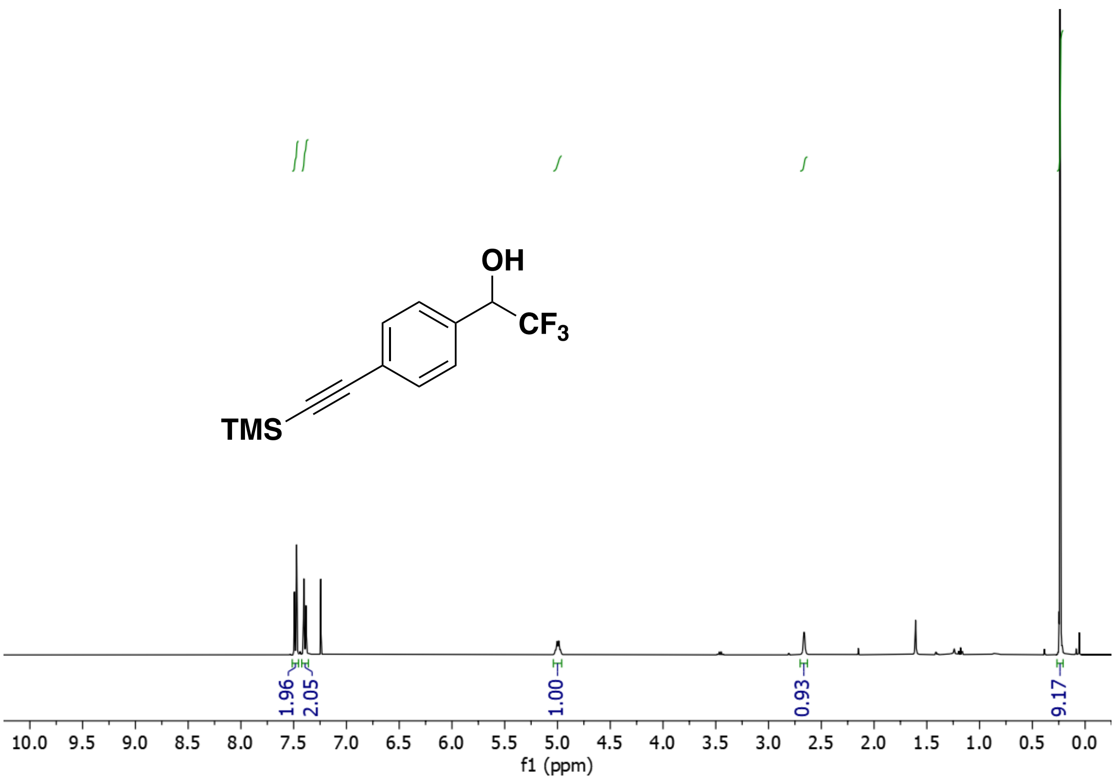

3x ^{13}C NMR (100 MHz, $CDCl_3$)

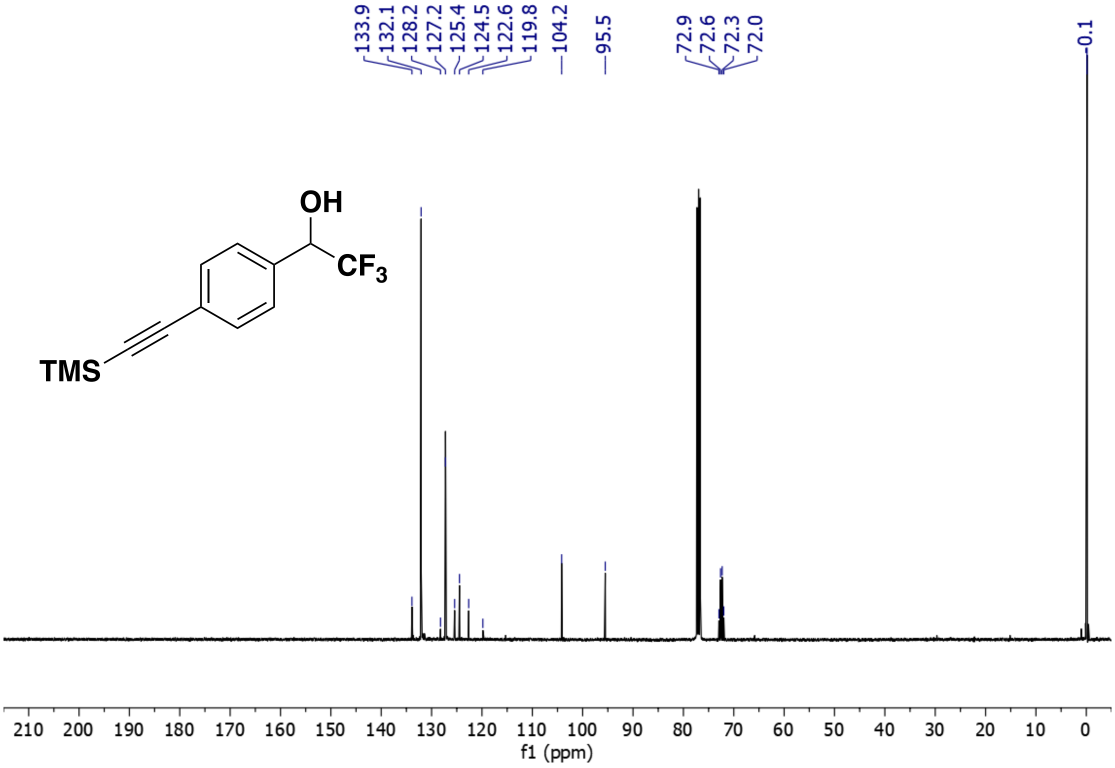

3x ^{19}F NMR (377 MHz, $CDCl_3$)

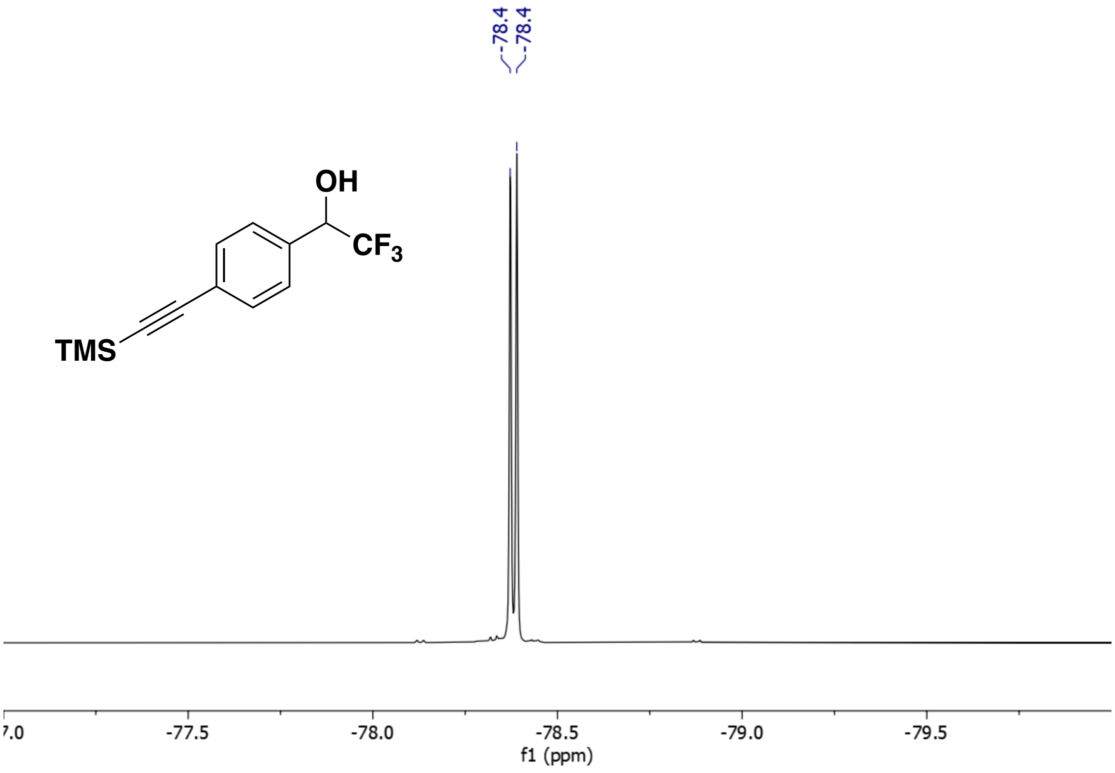

3y ^{1}H NMR (400 MHz, $CDCl_3$)

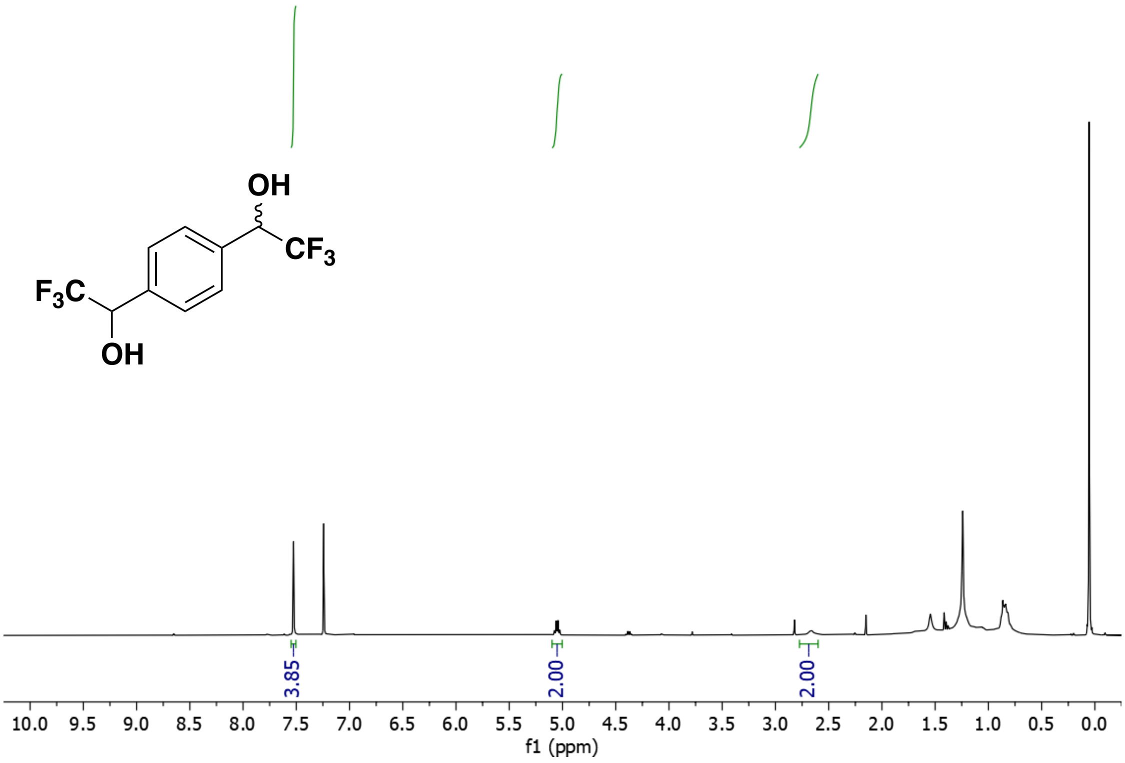

3y ^{13}C NMR (100 MHz, $CDCl_3$)

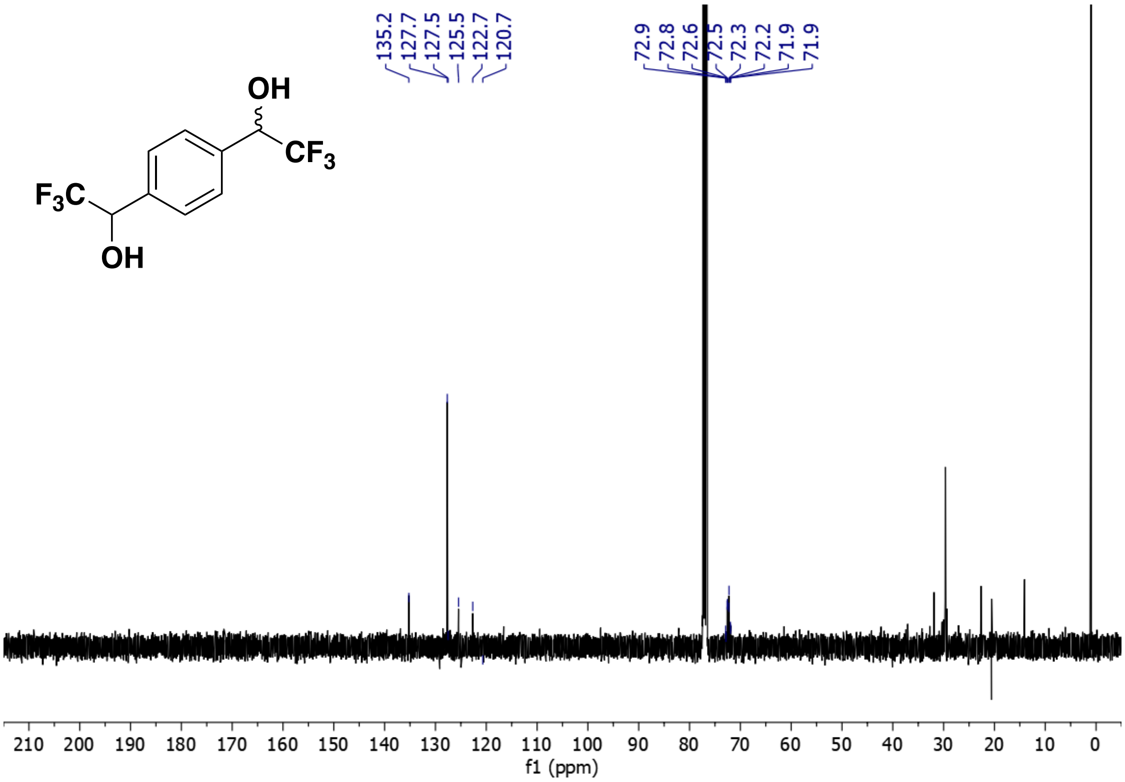

3y ^{19}F NMR (377 MHz, $CDCl_3$)

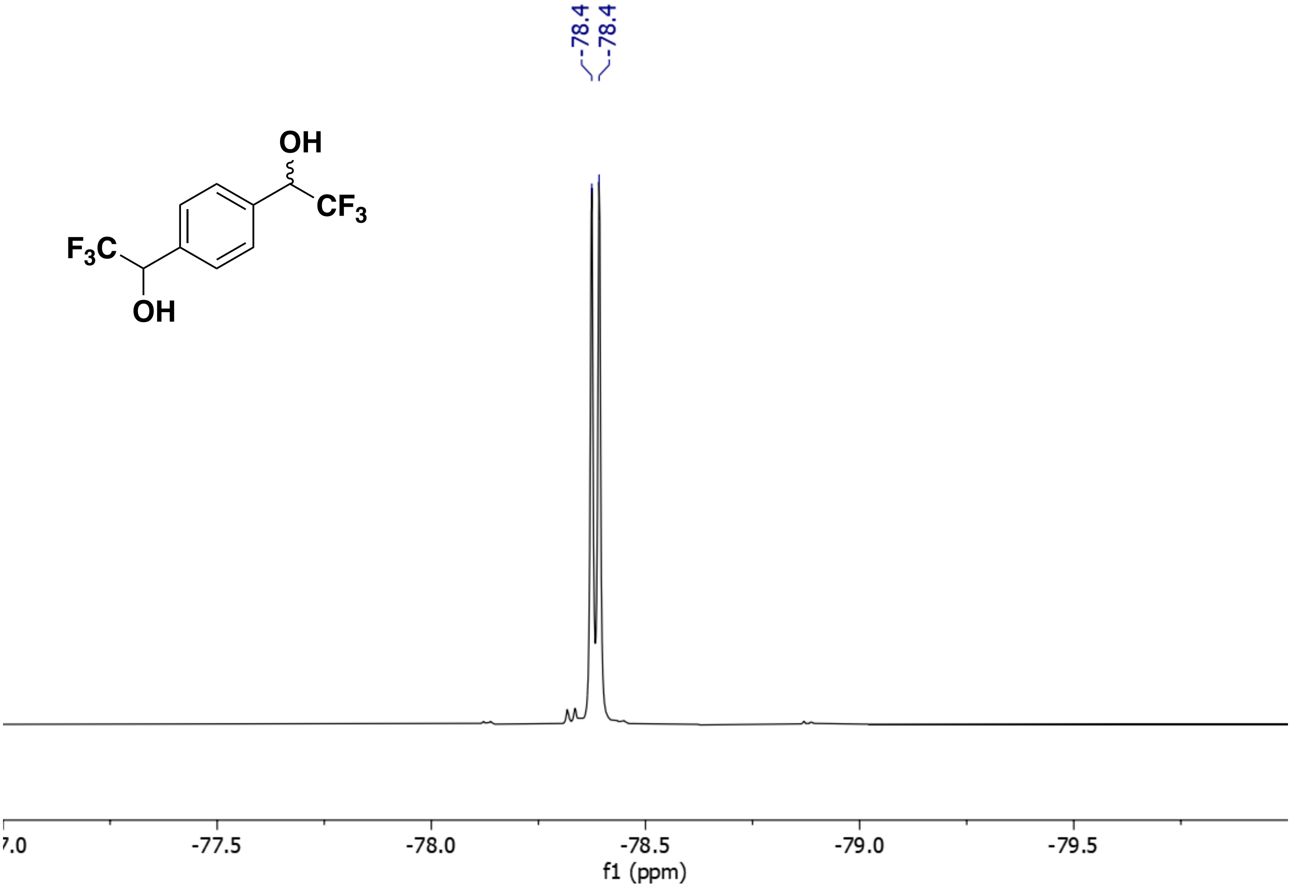

3z ^{1}H NMR (400 MHz, $CDCl_3$)

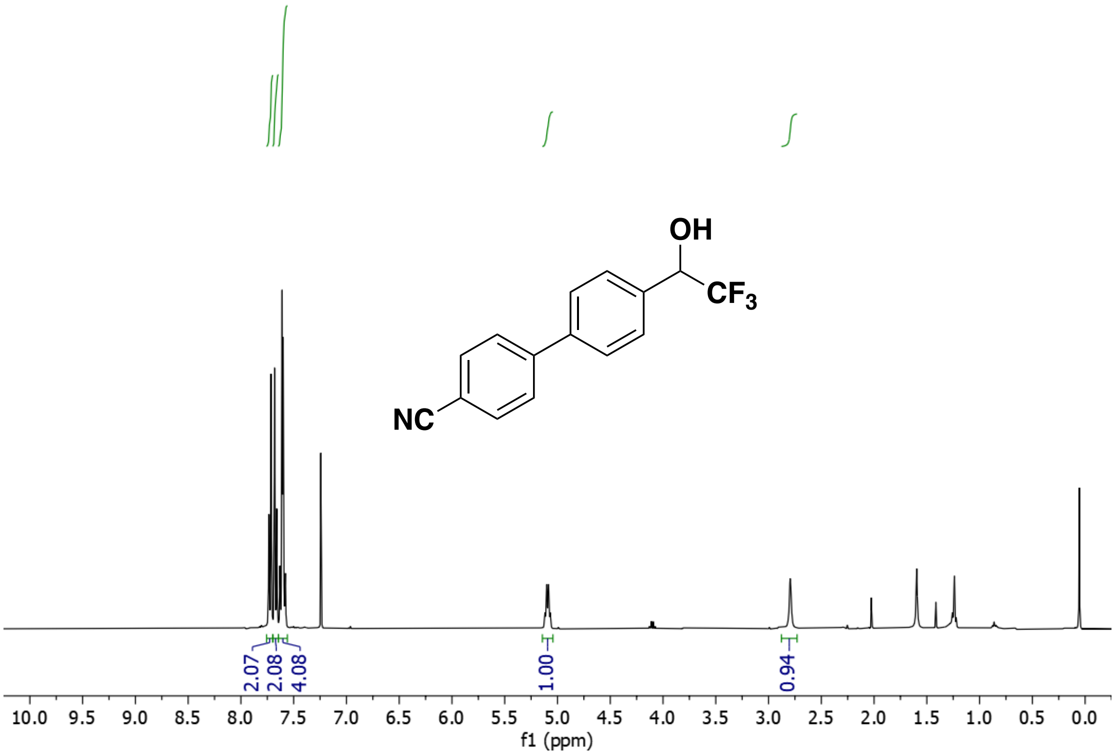

3z ^{13}C NMR (100 MHz, $CDCl_3$)

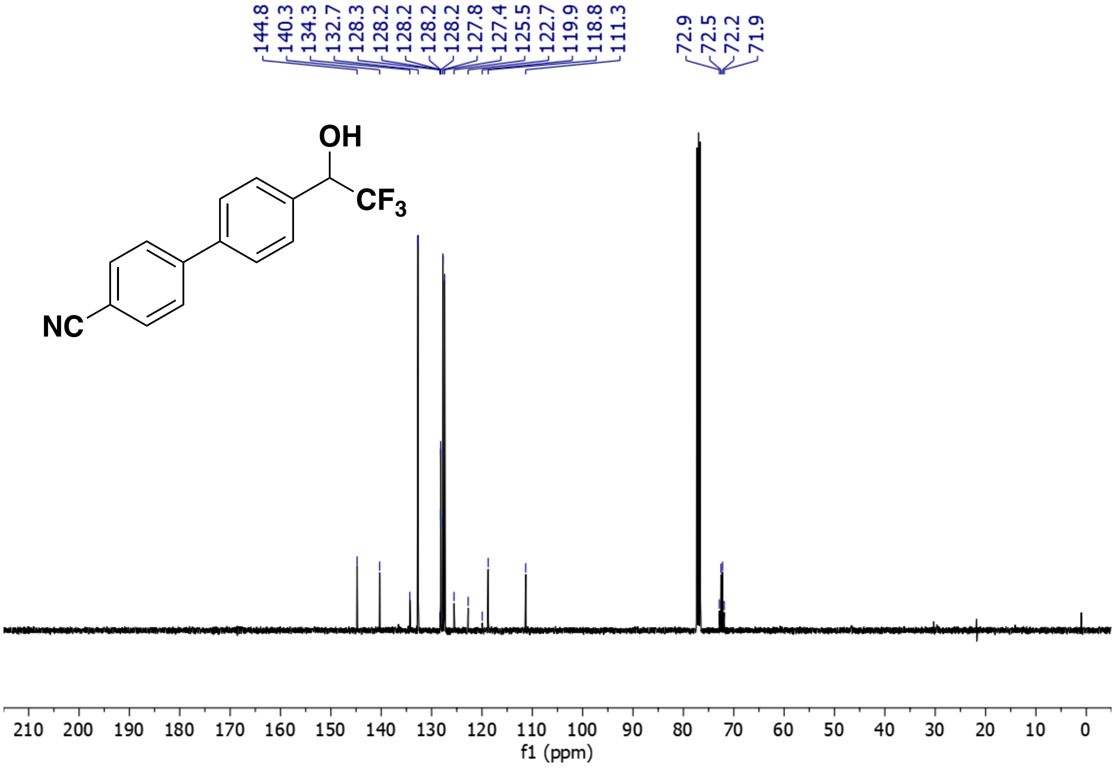

3z ^{19}F NMR (377 MHz, $CDCl_3$)

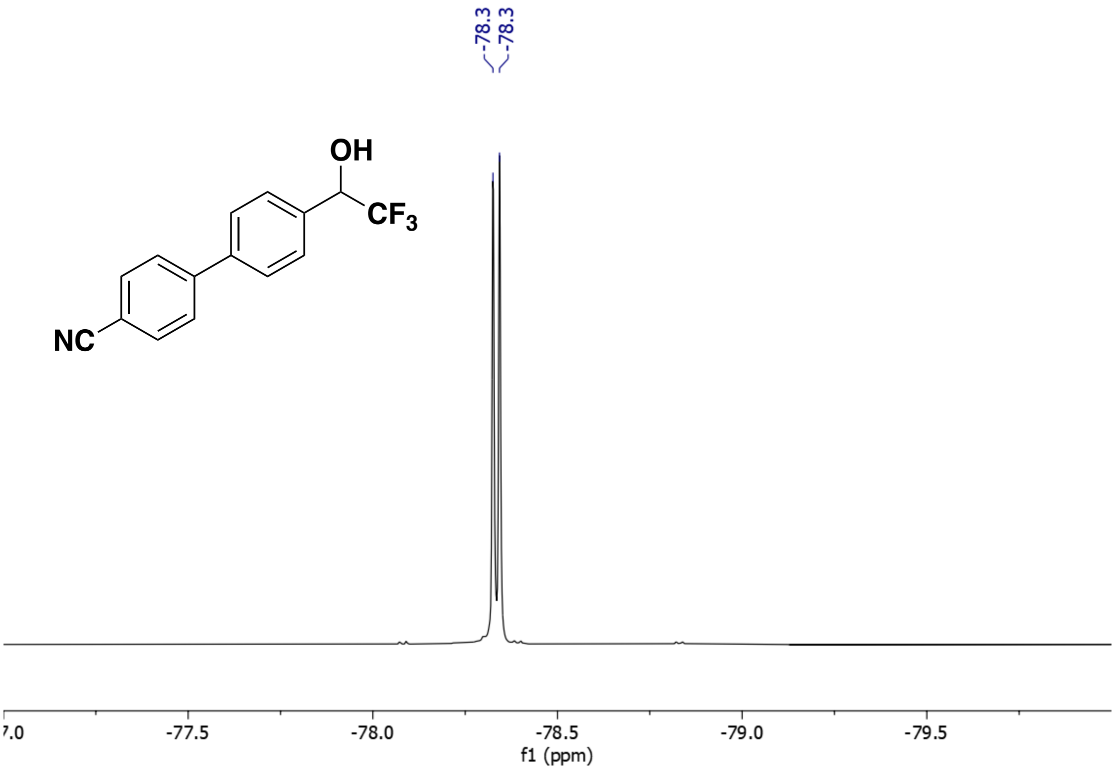

3a’ ^{1}H NMR (400 MHz, $CDCl_3$)

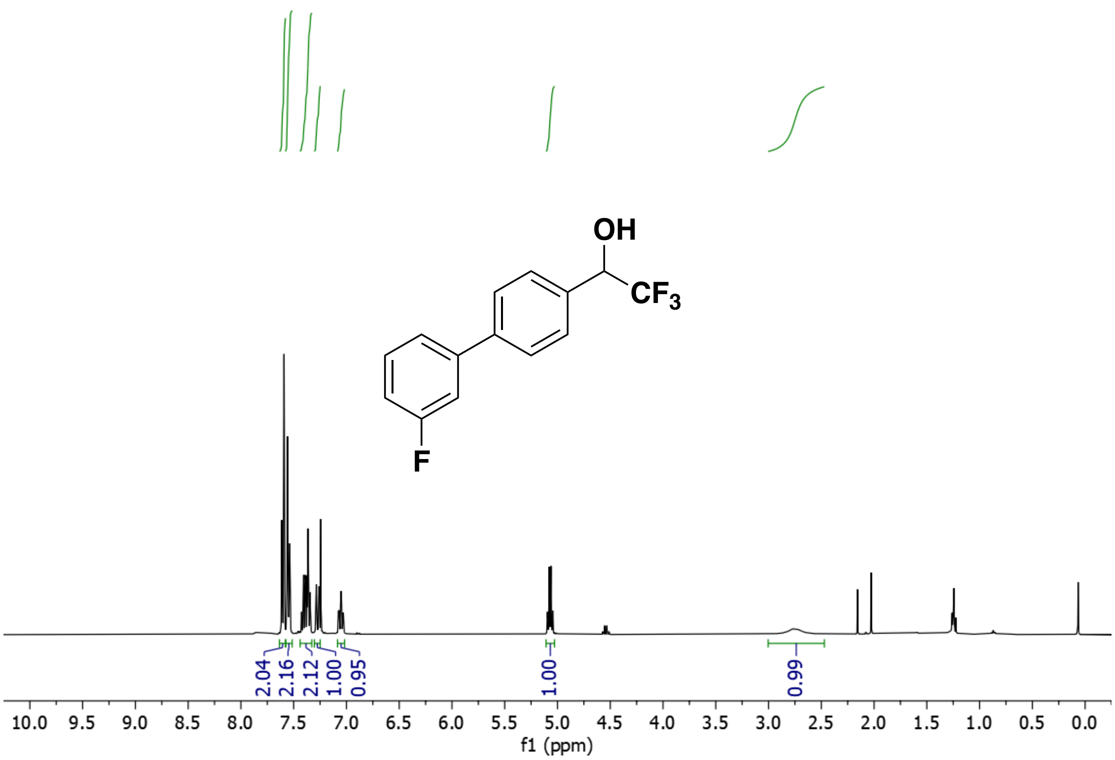

3a’ ^{13}C NMR (100 MHz, $CDCl_3$)

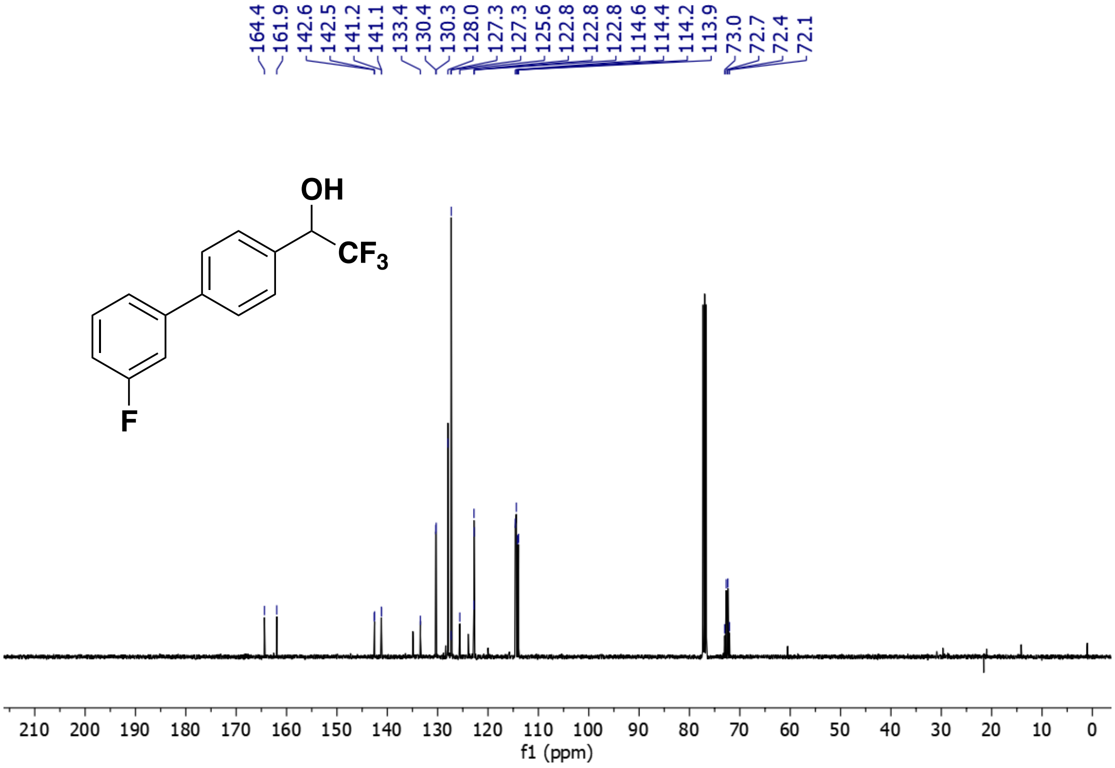

3a' ^{19}F NMR (377 MHz, $CDCl_3$)

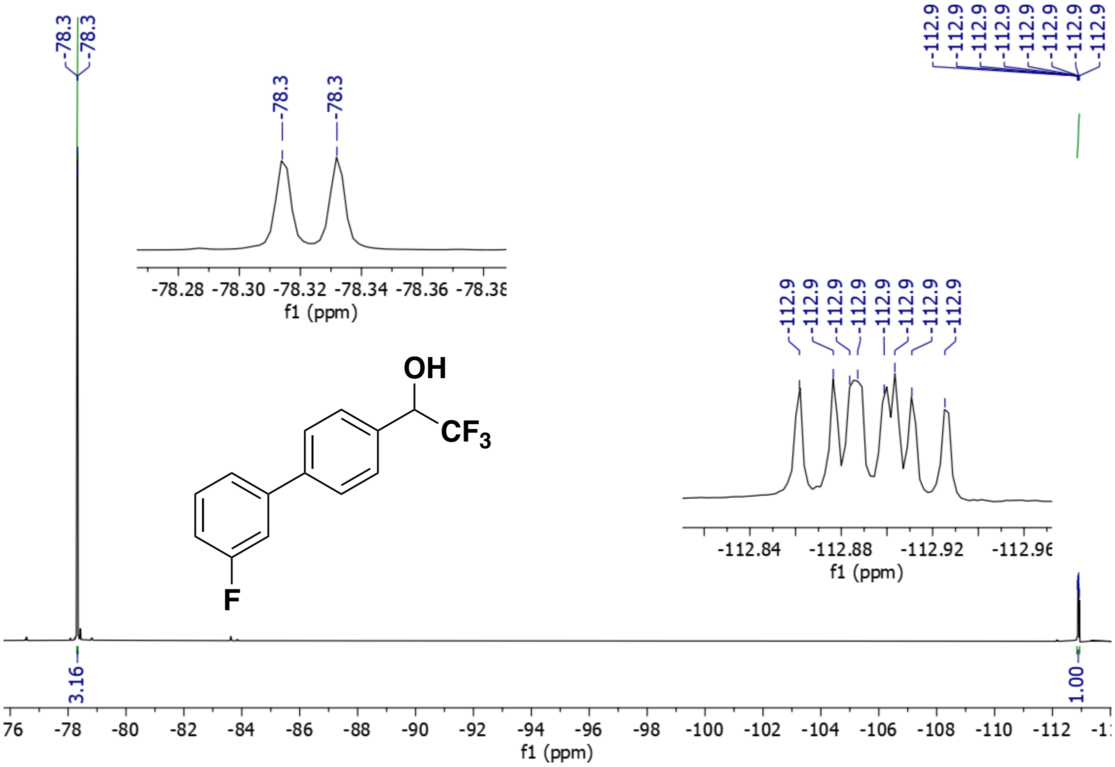

3b' ^{1}H NMR (400 MHz, $CDCl_3$)

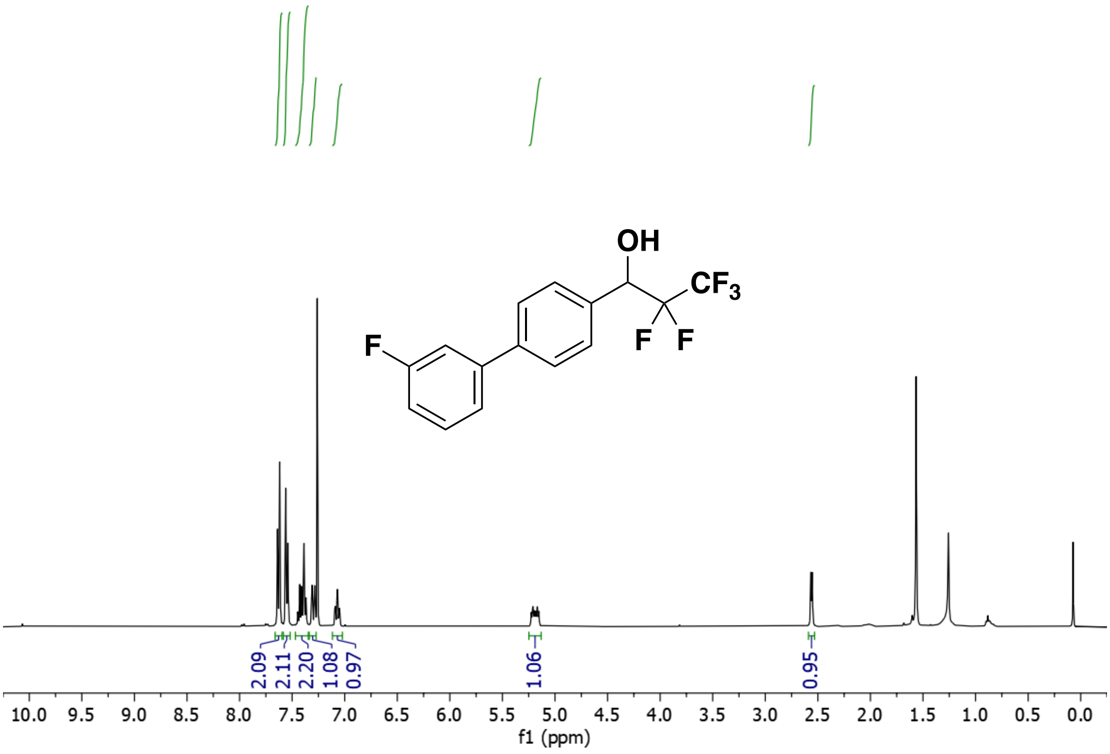

3b' ^{13}C NMR (100 MHz, $CDCl_3$)

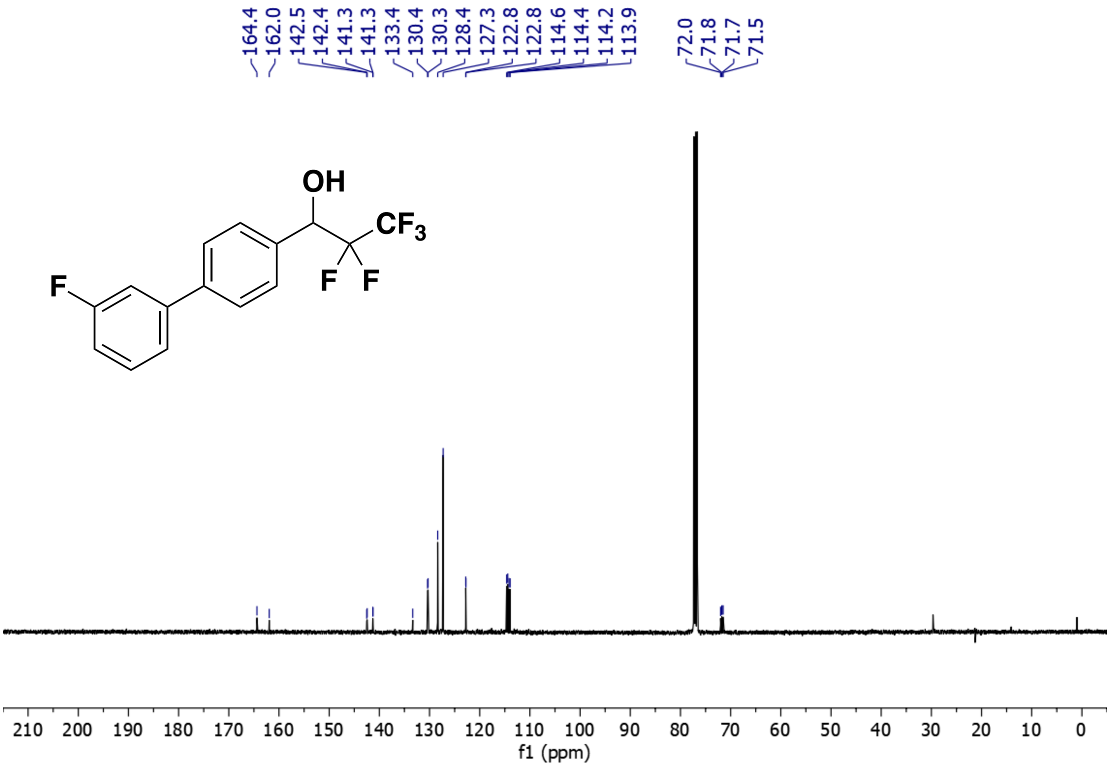

3b' ¹⁹F NMR (377 MHz, CDCl₃)

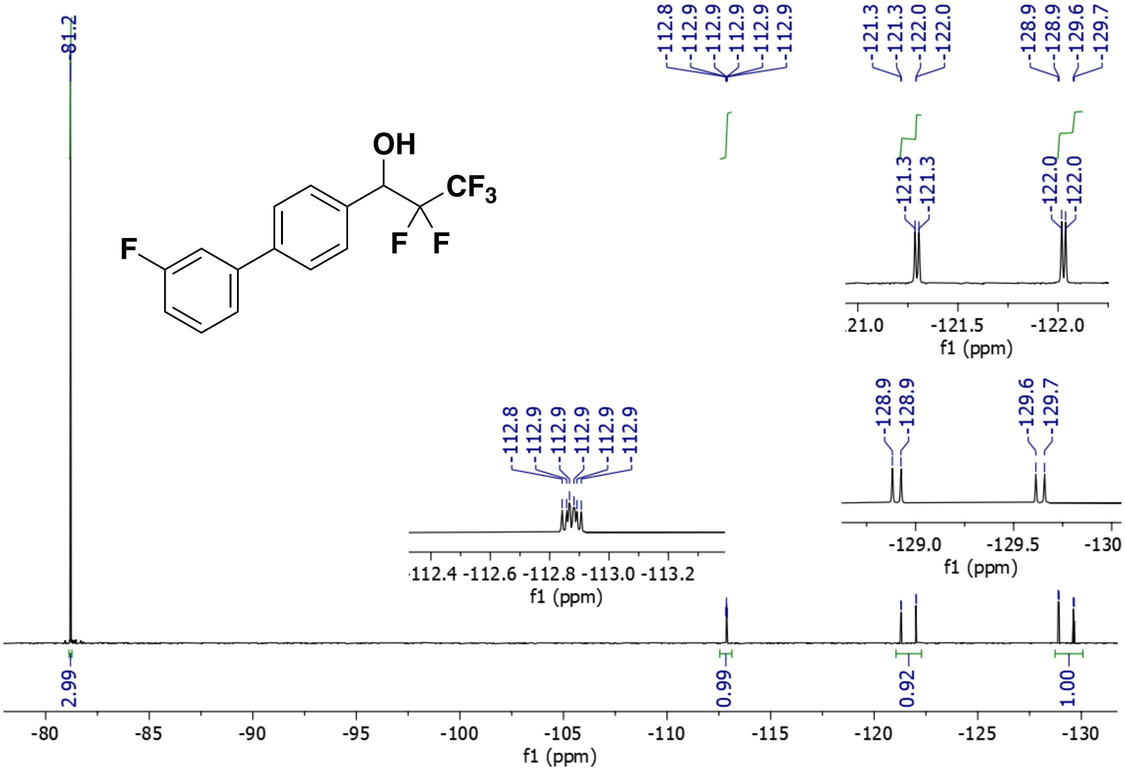

3c’ ^{1}H NMR (400 MHz, $CDCl_3$)

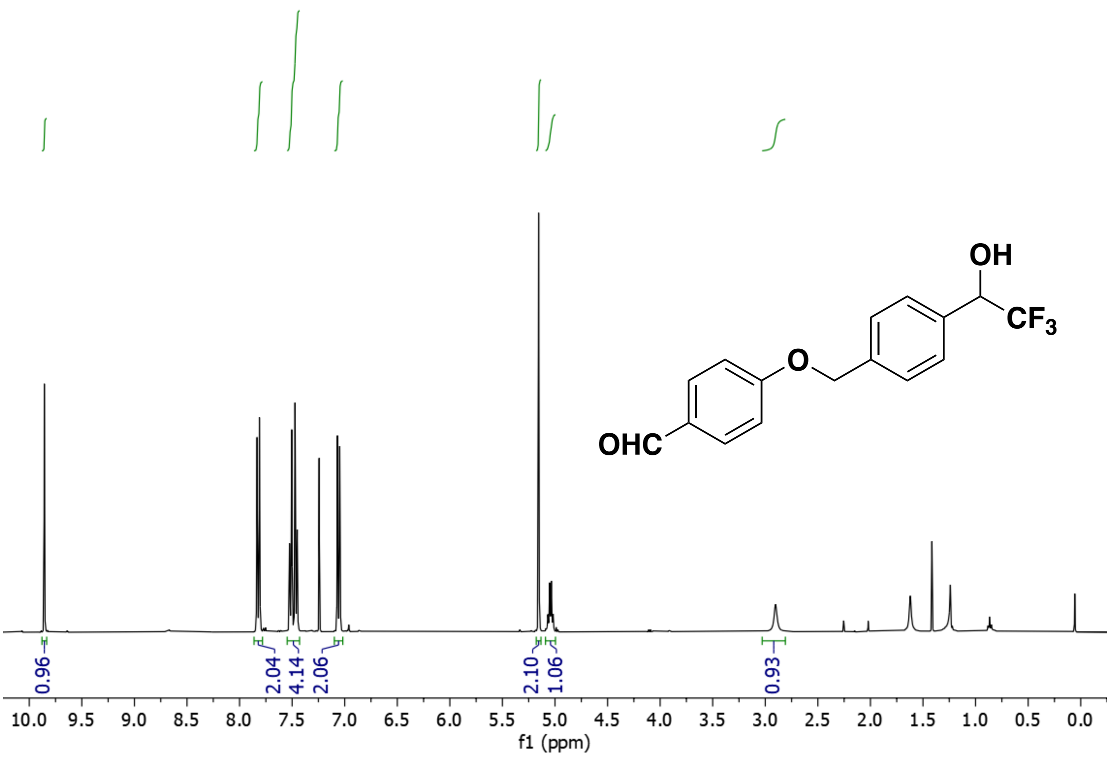

3c’ ^{13}C NMR (100 MHz, $CDCl_3$)

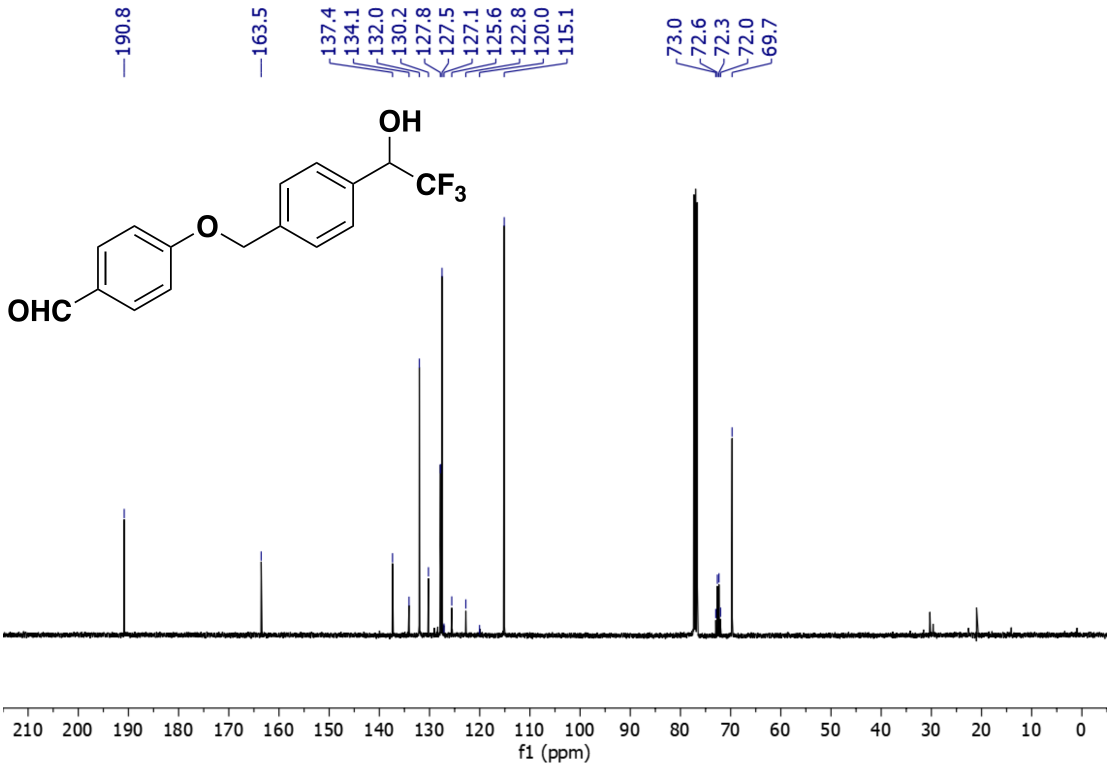

3c' ^{19}F NMR (377 MHz, $CDCl_3$)

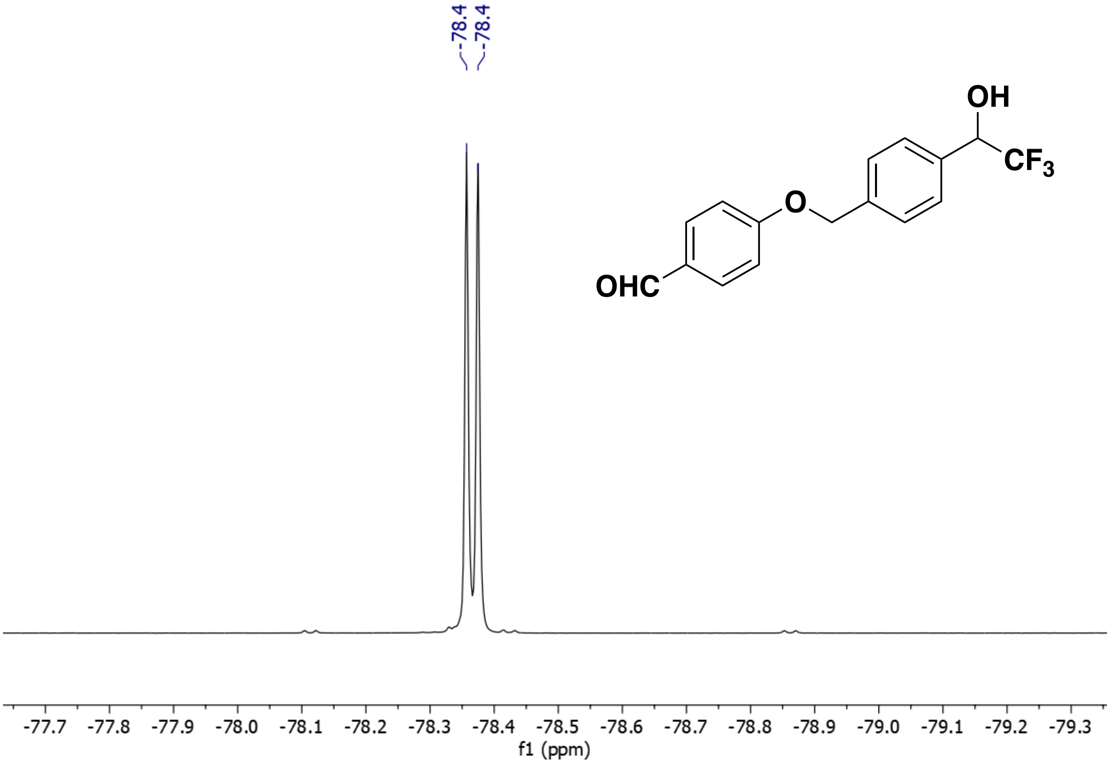

5a ¹H NMR (400 MHz, CDCl₃)

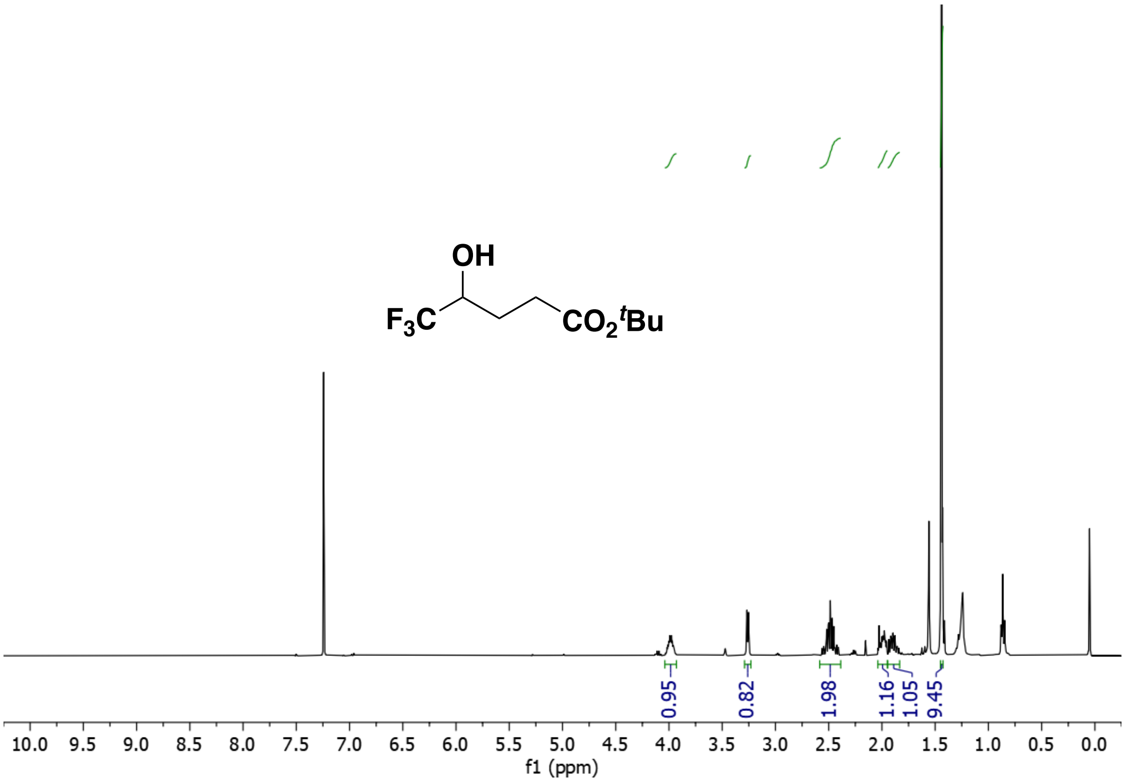

5a ¹³C NMR (100 MHz, CDCl₃)

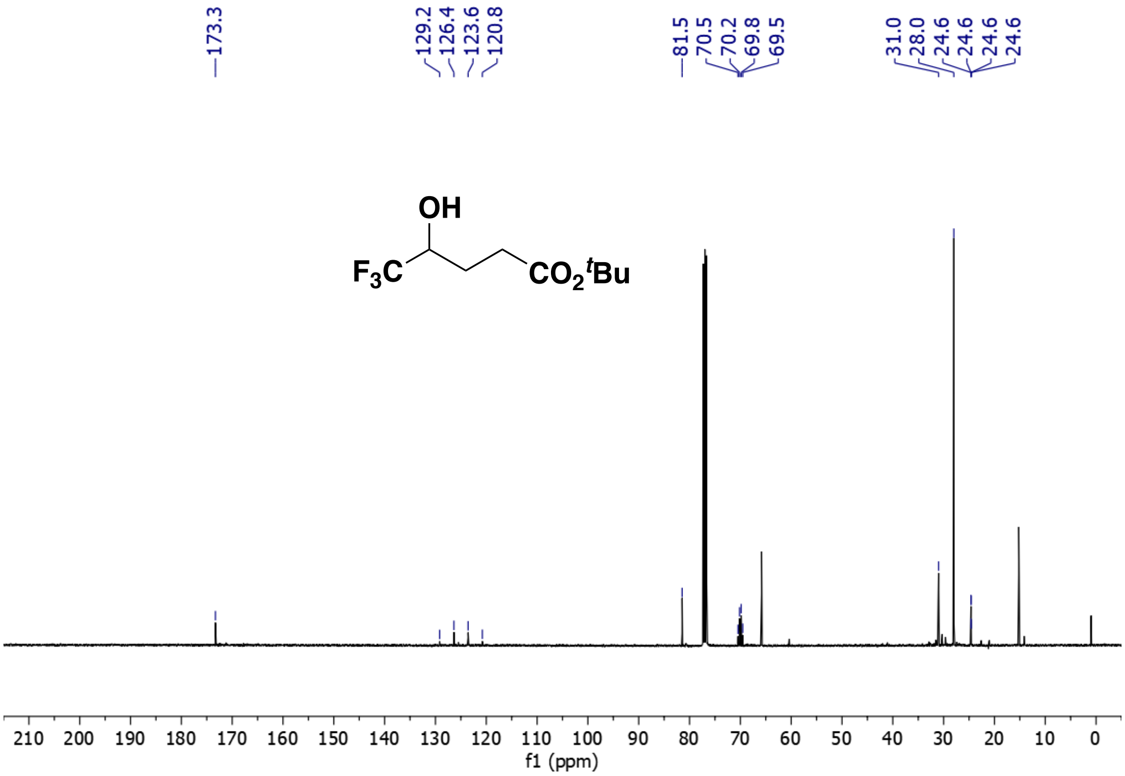

5a ^{19}F NMR (377 MHz, $CDCl_3$)

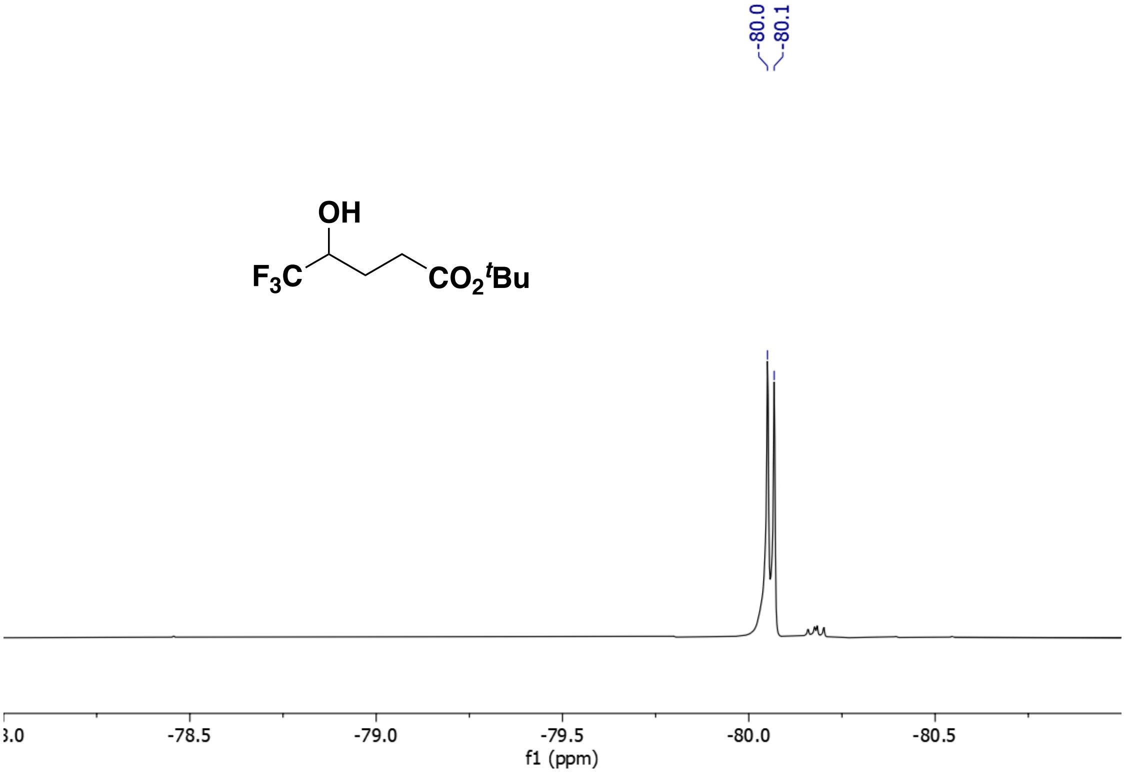

7a ^{1}H NMR (400 MHz, $CDCl_3$)

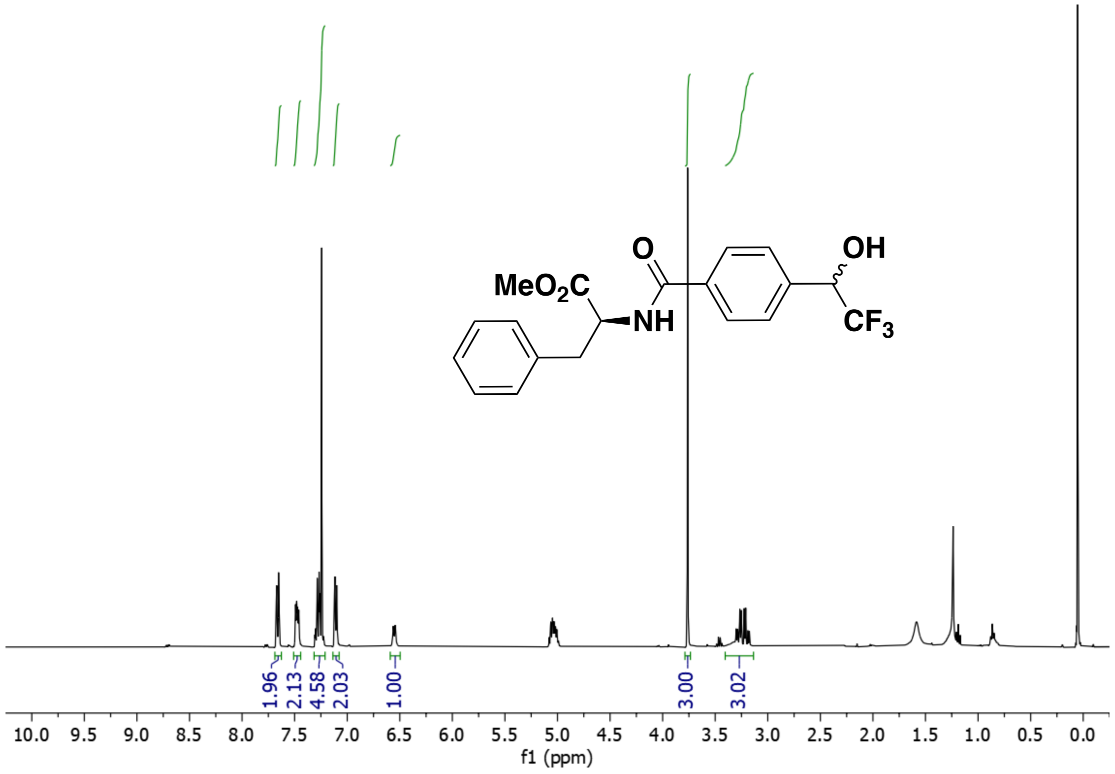

7a ^{13}C NMR (100 MHz, $CDCl_3$)

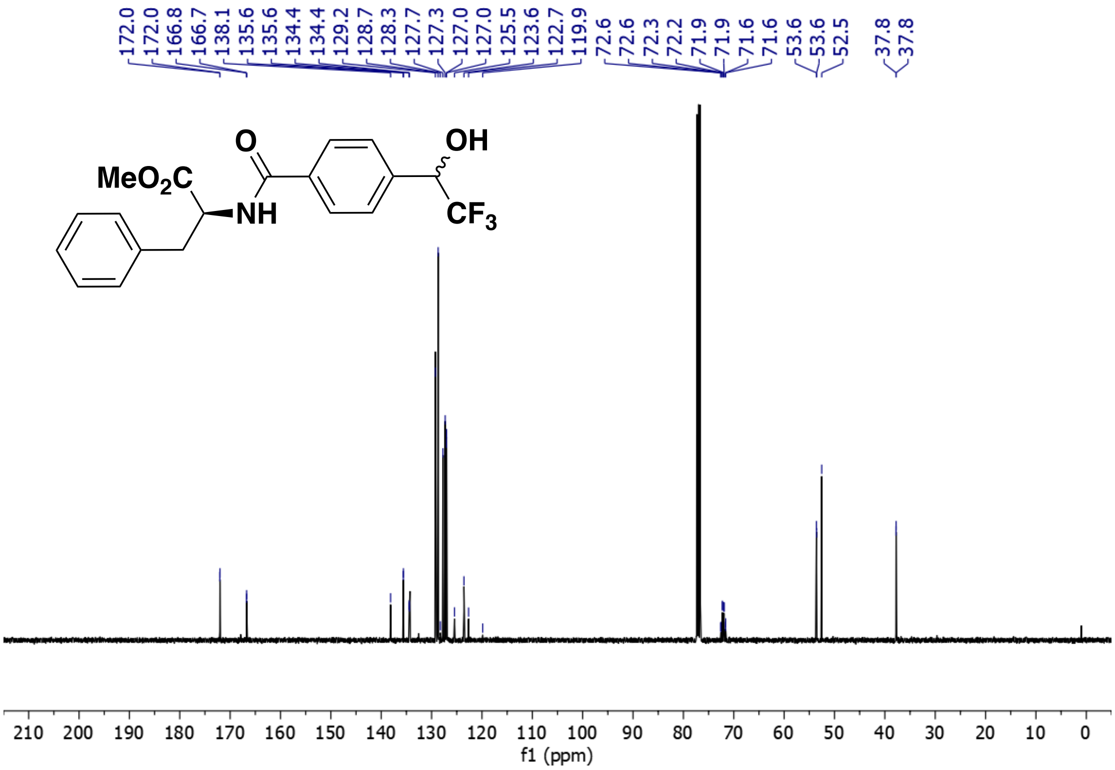

7a ^{19}F NMR (377 MHz, $CDCl_3$)

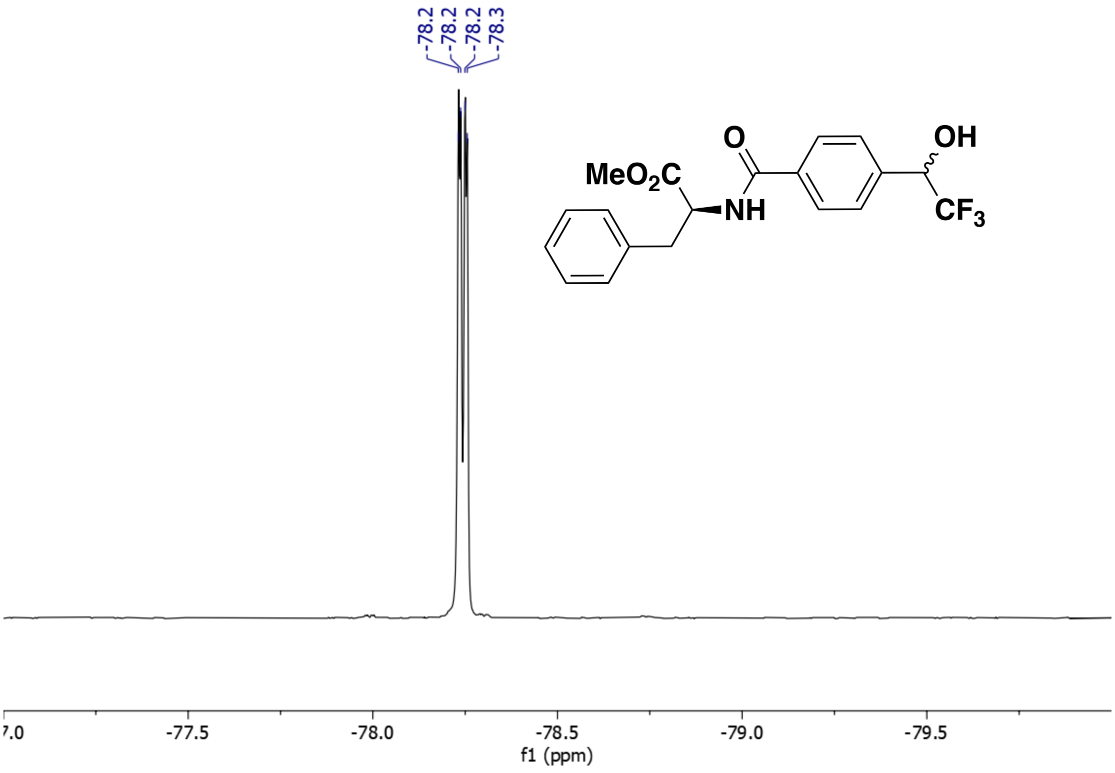

7b ^{1}H NMR (400 MHz, $CDCl_3$)

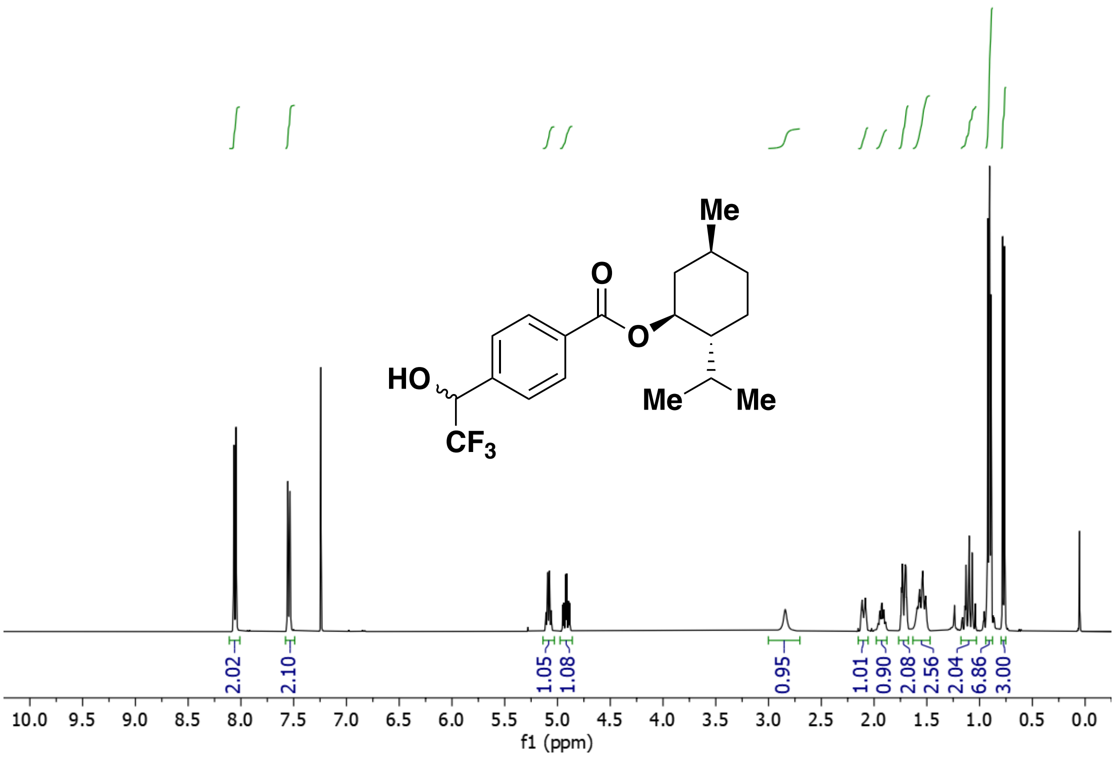

7b ^{13}C NMR (100 MHz, $CDCl_3$)

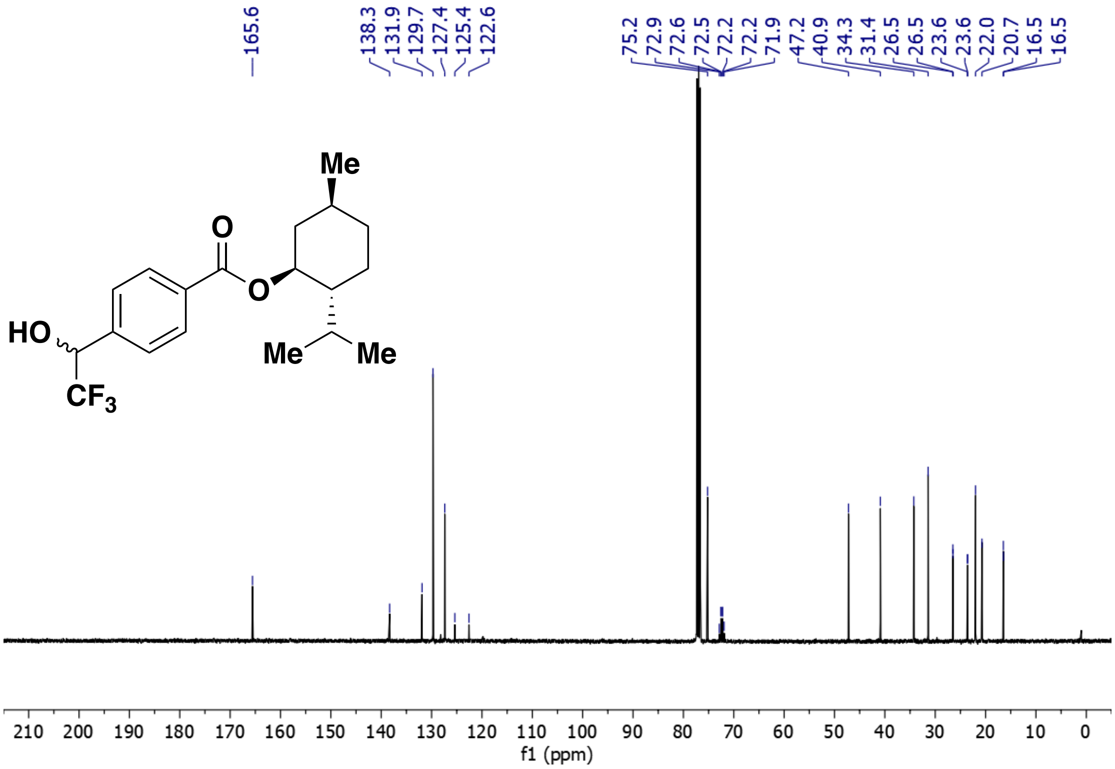

7b ^{19}F NMR (377 MHz, CDCl$_3$)

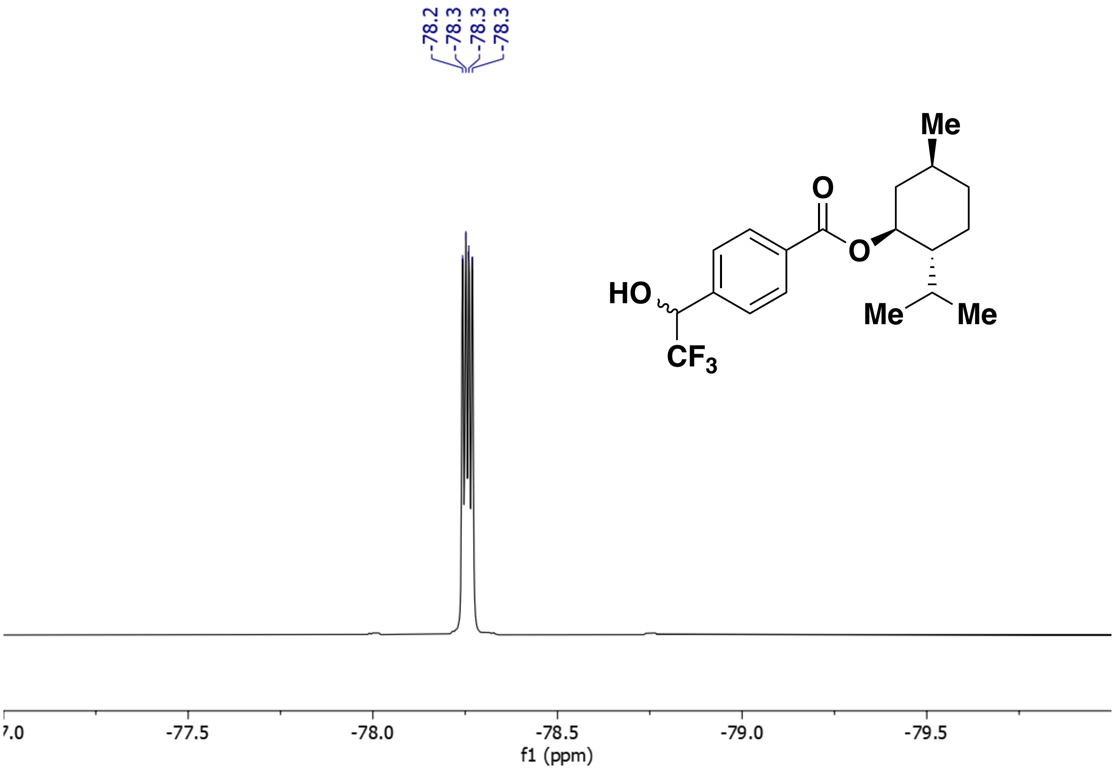

7c ^{1}H NMR (400 MHz, CDCl$_3$)

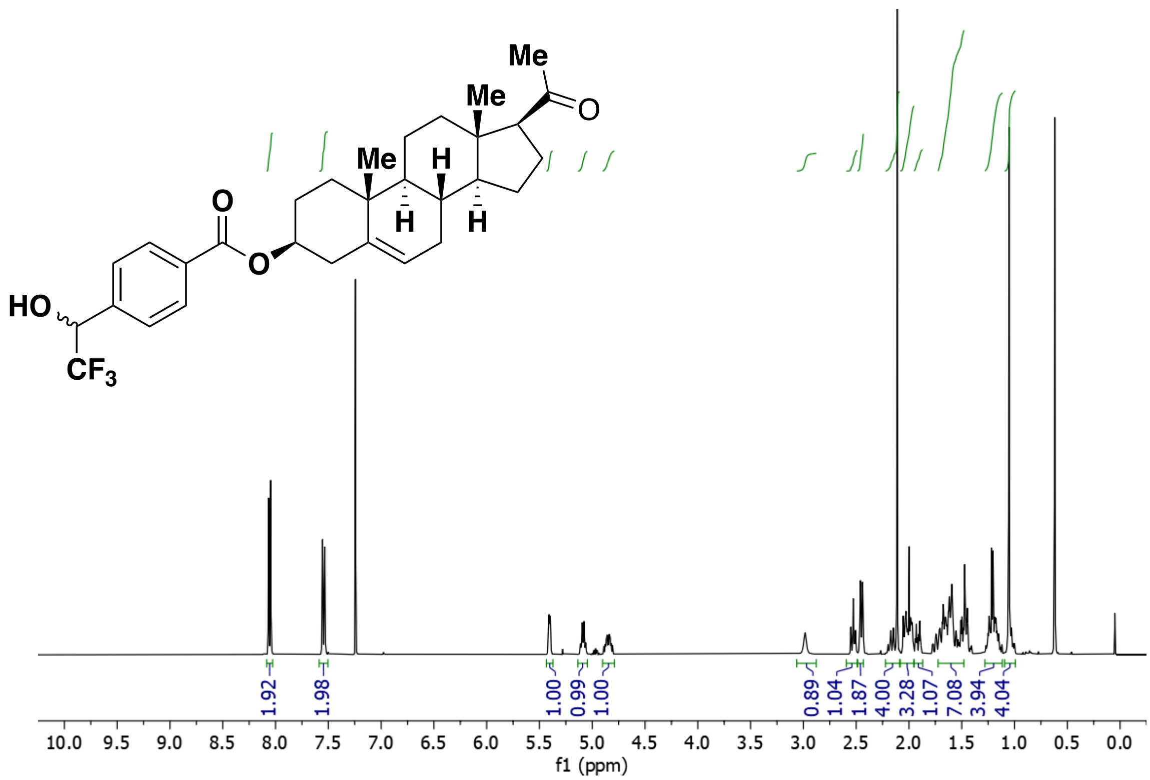

7c ^{13}C NMR (100 MHz, CDCl$_3$)

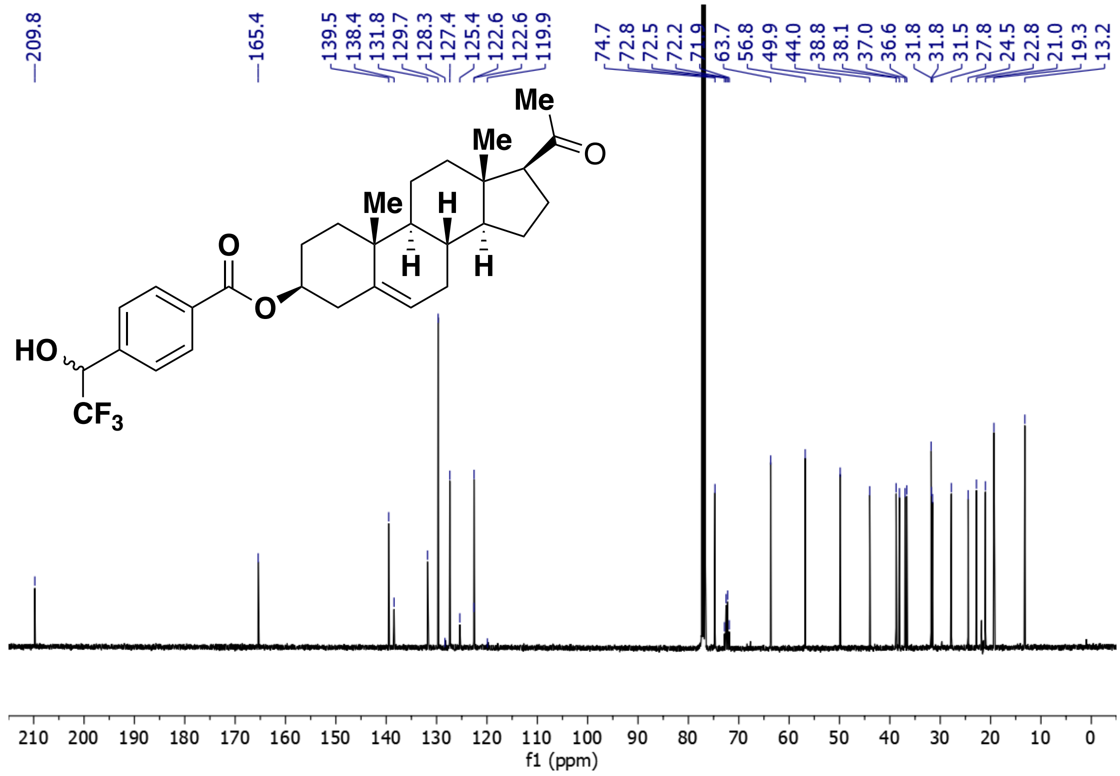

7c ^{19}F NMR (377 MHz, CDCl$_3$)

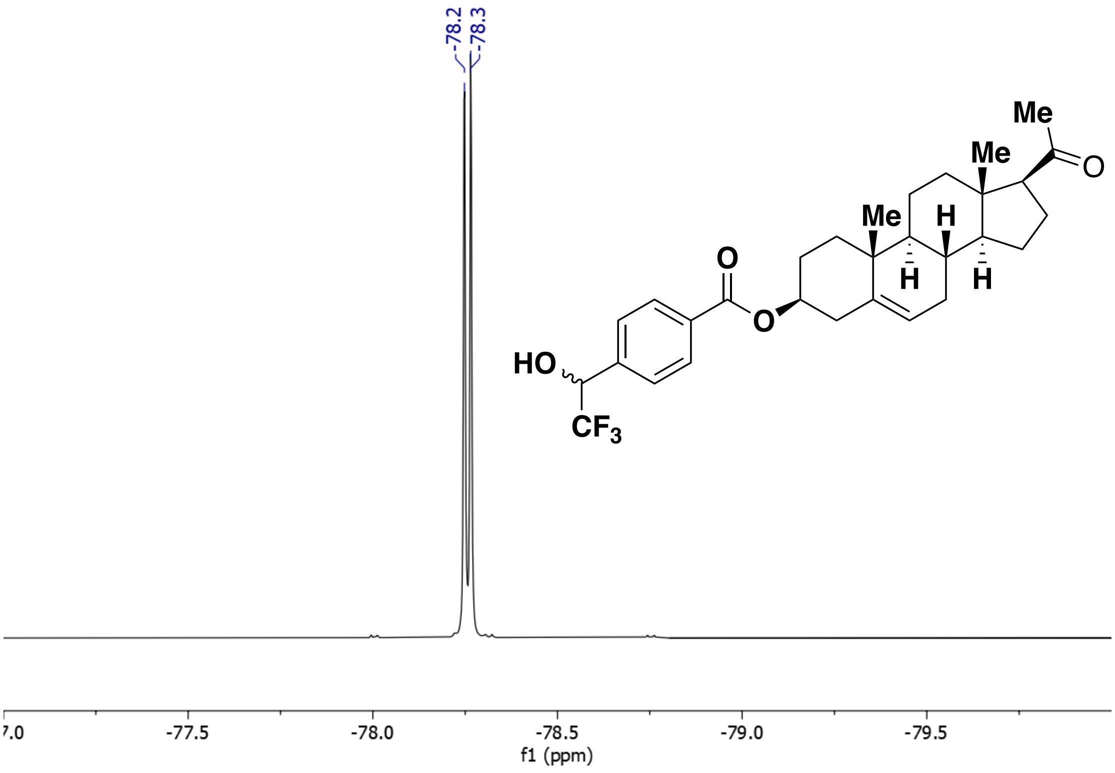

10. Cartesians

DMA (singlet)

C	-1.756810	0.820165	-0.000009
H	-1.669190	1.466670	0.891881
H	-2.754091	0.353808	-0.000045
H	-1.669149	1.466708	-0.891862
C	-0.730967	-0.288715	0.000004
O	-1.079963	-1.462007	0.000010
N	0.586935	0.075147	-0.000004
C	1.086155	1.429156	0.000007
H	1.715311	1.610389	0.894262
H	1.715211	1.610453	-0.894303
H	0.273513	2.168757	0.000084
C	1.612682	-0.941393	-0.000008
H	1.138090	-1.933167	-0.000073
H	2.257587	-0.844387	-0.895838
H	2.257519	-0.844473	0.895881

Zn (singlet)

Zn	-2.093185	0.535466	0.000000
Zn	0.000000	0.000000	0.000000
Zn	-2.093185	0.535466	0.000000
Zn	0.000000	0.000000	0.000000
Zn	-2.093185	0.535466	0.000000
Zn	0.000000	0.000000	0.000000
Zn	-2.093185	0.535466	0.000000
Zn	0.000000	0.000000	0.000000

Zn(DMA) (singlet)

Zn	1.969386	-0.070641	-0.001550
O	-1.327126	-1.025546	-1.467904
C	-1.307356	-0.679417	-0.293736
C	-1.357138	-1.700385	0.816931
N	-1.250509	0.636424	0.076369
H	-0.458541	-1.635599	1.458796

H	-1.395238	-2.700142	0.356935
H	-2.245410	-1.573813	1.462674
C	-1.132884	1.105935	1.436456
C	-1.073643	1.653628	-0.933069
H	-0.075768	1.335058	1.693518
H	-1.723367	2.033207	1.557772
H	-1.515656	0.371074	2.159857
H	-1.180241	1.196573	-1.927694
H	-1.827369	2.455585	-0.812819
H	-0.063281	2.108120	-0.853364

$Zn(DMA)_2$ (singlet)

C	3.225775	0.077436	1.549270
H	4.312654	0.016381	1.355011
H	3.028538	-0.313852	2.559621
H	2.934171	1.143953	1.513070
C	2.425795	-0.744009	0.566248
O	1.766100	-1.705802	0.940034
N	2.485947	-0.361894	-0.744361
C	3.221573	0.782513	-1.228368
H	3.609173	0.563740	-2.240357
H	2.571508	1.682365	-1.294702
H	4.084445	1.019638	-0.587973
C	1.632773	-0.995489	-1.723440
H	1.003855	-0.231168	-2.224991
H	2.233190	-1.513032	-2.498341
H	0.963403	-1.713472	-1.227169
Zn	0.018613	1.515034	0.311794
O	-1.559073	-1.744017	-0.999474
C	-2.062088	-1.166193	-0.043073
C	-1.815738	-1.639976	1.368299
N	-2.874362	-0.077107	-0.199099
H	-1.361754	-0.841295	1.984308
H	-1.116406	-2.488956	1.324151

H	-2.747418	-1.966353	1.866485
C	-3.429812	0.693491	0.888113
C	-3.034694	0.513181	-1.507036
H	-2.793205	1.574778	1.122908
H	-4.433007	1.059824	0.601714
H	-3.544371	0.091375	1.801728
H	-2.547991	-0.130081	-2.254879
H	-4.107660	0.621397	-1.758171
H	-2.566334	1.519572	-1.536560

Zn(DMA)$_3$ (singlet)

C	3.022654	2.530817	-1.418252
H	3.330193	3.585064	-1.294958
H	2.983666	2.296114	-2.493812
H	3.784905	1.890114	-0.938265
C	1.664580	2.242142	-0.828195
O	0.777854	1.742867	-1.511262
N	1.473267	2.571154	0.484301
C	2.551609	2.920028	1.380657
H	2.166625	3.582924	2.176980
H	2.986102	2.013670	1.849480
H	3.354367	3.463624	0.859728
C	0.263799	2.124047	1.136408
H	0.398570	1.105625	1.564681
H	0.006287	2.817547	1.958211
H	-0.572715	2.086741	0.420374
Zn	-0.767808	-1.266949	-0.227242
O	-2.913057	1.861563	-0.539539
C	-3.473015	0.771750	-0.554295
C	-3.901170	0.137488	-1.855005
N	-3.753917	0.078912	0.590301
H	-3.414951	-0.845940	-1.996204
H	-3.599479	0.806600	-2.675802
H	-4.994998	-0.015137	-1.904811
C	-4.302853	-1.257234	0.612760

C	-3.246925	0.556123	1.855700
H	-3.499237	-2.026000	0.594460
H	-4.888817	-1.393097	1.539607
H	-4.979957	-1.437556	-0.236064
H	-2.861162	1.578775	1.729318
H	-4.049145	0.557532	2.618203
H	-2.423706	-0.096314	2.217022
O	2.866480	-0.133286	0.534396
C	2.549950	-1.305831	0.700220
C	2.241502	-1.837004	2.078916
N	2.462516	-2.189669	-0.337375
H	1.222997	-2.266110	2.123505
H	2.308460	-0.999602	2.791642
H	2.953717	-2.622478	2.392407
C	2.054138	-3.568839	-0.207709
C	2.581323	-1.719278	-1.698108
H	0.969821	-3.690249	-0.421248
H	2.617815	-4.185128	-0.932313
H	2.260783	-3.966340	0.797072
H	2.789716	-0.639004	-1.695627
H	3.395019	-2.250898	-2.229341
H	1.632532	-1.899200	-2.244921

I (triplet)

E(UM06-singlet) = -7379.08935601

E(UM06-triplet) = -7379.14212090

C	0.518062	1.410279	-0.113572
C	-1.760420	1.809386	0.004415
C	-1.577373	3.180168	0.121898
C	-0.265786	3.674466	0.115700
C	0.794985	2.761481	-0.006759
C	1.559708	0.364658	-0.229754
C	1.951587	-1.910826	-0.386824
C	3.328191	-1.737053	-0.413915
C	3.829376	-0.429710	-0.347207

C	2.917003	0.633582	-0.246641
H	-2.768491	1.375842	-0.002152
H	-2.448740	3.832759	0.214751
H	1.816791	3.151020	-0.010488
H	1.518448	-2.918199	-0.424052
H	3.979290	-2.611698	-0.482791
H	3.311837	1.651400	-0.183560
N	-0.751115	0.949277	-0.110171
N	1.090624	-0.898688	-0.310281
O	0.063475	4.955165	0.218389
O	5.115987	-0.108055	-0.366994
C	-0.954215	5.927457	0.353870
H	-0.446939	6.900438	0.424674
H	-1.623204	5.927236	-0.526572
H	-1.546175	5.756198	1.272024
C	6.087555	-1.130048	-0.471805
H	7.066252	-0.628950	-0.478813
H	6.035285	-1.817589	0.392672
H	5.962453	-1.700965	-1.410553
Ni	-0.927409	-1.057395	-0.209311
Br	-1.025218	-1.830472	2.054769
Br	-2.896886	-1.477085	-1.456738

$ZnBr_2(DMA)_2$ (singlet)

Zn	-0.368781	-0.642243	0.218955
Br	-0.102498	-0.580224	2.620216
Br	-0.334903	-2.485901	-1.341802
O	-2.502397	-0.458506	0.337563
C	-3.289528	0.157665	-0.395361
O	-0.493727	1.263540	-0.532698
C	0.418789	1.807875	-1.188169
O	1.828036	-0.127577	-0.043980
C	2.986198	-0.461529	-0.318532
C	3.279769	-1.318222	-1.519996
H	3.795905	-0.734339	-2.305375

H	3.919536	-2.183384	-1.272254
H	2.320002	-1.683966	-1.920155
C	0.753649	1.320794	-2.565937
H	1.842282	1.227062	-2.724797
H	0.283122	0.333160	-2.706458
H	0.356804	2.012033	-3.333915
C	-3.082359	0.230921	-1.881991
H	-2.825529	1.259930	-2.194716
H	-2.248913	-0.437150	-2.152315
H	-3.985935	-0.078196	-2.437032
N	4.021257	-0.050733	0.449971
N	1.098807	2.845625	-0.682742
N	-4.369942	0.778626	0.128024
C	2.130603	3.556132	-1.409671
H	2.156762	4.602084	-1.059260
H	3.130096	3.107340	-1.237412
H	1.926647	3.571585	-2.490993
C	0.927687	3.218625	0.708159
H	1.921738	3.319367	1.181870
H	0.399798	4.187245	0.797064
H	0.354820	2.438272	1.231695
C	5.417835	-0.287463	0.157677
H	5.559869	-0.717041	-0.843560
H	5.971058	0.669973	0.197378
H	5.867121	-0.971624	0.903681
C	3.771702	0.728578	1.645068
H	4.418067	0.358741	2.462328
H	4.009224	1.799034	1.477996
H	2.716555	0.629998	1.944395
C	-5.381339	1.443461	-0.667170
H	-5.793326	2.287302	-0.087083
H	-4.962311	1.854256	-1.597739
H	-6.217339	0.761729	-0.921584
C	-4.647231	0.690258	1.547488

H	-4.809019	1.701801	1.963282
H	-5.559894	0.090248	1.730241
H	-3.795475	0.219391	2.058343

III (doublet)

E(UM06-doublet) = -4805.33031834

E(UM06-quadruplet) = -4805.27819511

C	-0.210785	1.196881	-0.000051
C	2.090083	1.385278	-0.000125
C	2.040995	2.774022	-0.000065
C	0.782173	3.389300	0.000010
C	-0.360257	2.574202	0.000015
C	-1.340050	0.241731	-0.000049
C	-1.927356	-1.991152	-0.000131
C	-3.285479	-1.700646	-0.000058
C	-3.674212	-0.354763	0.000028
C	-2.670836	0.625880	0.000028
H	3.054827	0.860881	-0.000178
H	2.972302	3.346118	-0.000074
H	-1.342677	3.055892	0.000074
H	-1.587610	-3.034038	-0.000194
H	-4.008992	-2.519881	-0.000067
H	-2.971719	1.677863	0.000094
N	1.007241	0.610457	-0.000119
N	-0.968697	-1.062063	-0.000125
O	0.576847	4.704958	0.000079
O	-4.934016	0.076671	0.000111
C	1.687193	5.576290	0.000079
H	1.282371	6.599011	0.000137
H	2.308828	5.430928	0.903581
H	2.308766	5.431007	-0.903477
C	-5.985023	-0.865293	0.000119
H	-6.921901	-0.289047	0.000192
H	-5.949955	-1.502782	-0.903444
H	-5.949867	-1.502865	0.903621

Ni	0.955448	-1.374908	-0.000126
Br	2.907484	-2.607367	0.000133

II (singlet)

E(UM06-singlet) = -681.484264046

E(UM06-triplet) = -681.356666685

C	0.242346	-1.185687	0.000126
C	1.634762	-1.152967	0.000107
C	2.321324	0.067204	-0.000024
C	1.585971	1.259701	-0.000095
C	0.196808	1.242911	-0.000100
C	-0.469875	0.014381	0.000004
H	-0.282195	-2.147390	0.000225
H	2.180100	-2.103085	0.000239
H	2.130631	2.210130	-0.000161
H	-0.365552	2.183268	-0.000169
I	-2.576620	-0.024543	-0.000004
C	3.813562	0.154140	0.000017
O	4.368414	1.236179	0.000244
C	4.600073	-1.126492	-0.000212
H	5.676199	-0.891189	-0.000560
H	4.362573	-1.739738	0.888802
H	4.361984	-1.739781	-0.889044

IV (doublet)

E(UM06-doublet) = -5486.83987779

E(UM06-quadruplet) = -5486.77828441

C	-1.062147	1.747953	0.496543
C	-2.472356	0.133774	1.345910
C	-3.606788	0.896567	1.101276
C	-3.439240	2.135218	0.470633
C	-2.133140	2.575454	0.201116
C	0.351738	2.130280	0.272567
C	2.548795	1.447873	0.447704
C	3.035098	2.682533	0.033613
C	2.107010	3.679922	-0.290286

C	0.740162	3.388846	-0.157042
H	-2.565231	-0.851422	1.820629
H	-4.589010	0.506664	1.381395
H	-2.001579	3.559831	-0.257104
H	3.241551	0.638245	0.713565
H	4.114330	2.843272	-0.027836
H	0.017615	4.177020	-0.387579
N	-1.238713	0.519749	1.029652
N	1.253522	1.165747	0.554961
O	-4.431026	2.942476	0.108644
O	2.417209	4.903012	-0.710772
C	-5.763957	2.492463	0.245958
H	-6.404064	3.273939	-0.188933
H	-5.913650	1.543938	-0.304025
H	-6.032382	2.353914	1.309980
C	3.775367	5.258746	-0.862684
H	3.787447	6.296810	-1.225866
H	4.312130	5.204937	0.102968
H	4.277427	4.607849	-1.602905
Ni	0.420401	-0.677275	0.907138
Br	1.775398	-1.725127	2.624520
C	0.375284	-1.466255	-0.991924
C	-0.291351	-2.432461	-0.183668
C	-1.695084	-2.536098	-0.268130
C	-2.435919	-1.689470	-1.085071
C	-1.754824	-0.744196	-1.895558
C	-0.384946	-0.651451	-1.880550
H	0.272140	-3.223152	0.324866
H	-2.190693	-3.306921	0.333809
H	-2.350436	-0.091927	-2.544120
H	0.130420	0.059983	-2.537926
I	2.431428	-1.778835	-1.493560
C	-3.921916	-1.706029	-1.125202
O	-4.535218	-0.864957	-1.760800

C	-4.650954	-2.763231	-0.341155
H	-4.313451	-3.779494	-0.614780
H	-4.472831	-2.644832	0.744830
H	-5.731983	-2.672590	-0.533494

V (doublet)

E(UM06-doublet) = -5486.84271337

E(UM06-quadruplet) = -5486.78633087

C	-1.358446	1.666721	-0.106567
C	-0.102752	1.925044	-2.036640
C	-0.452879	3.261170	-2.162013
C	-1.299838	3.816490	-1.192104
C	-1.757214	2.991721	-0.152353
C	-1.805429	0.709596	0.926825
C	-1.731754	-1.493992	1.623381
C	-2.553806	-1.241584	2.713816
C	-3.009924	0.069097	2.909637
C	-2.625704	1.055310	1.986841
H	0.551482	1.449941	-2.778746
H	-0.067433	3.839150	-3.005240
H	-2.429117	3.422462	0.595067
H	-1.344930	-2.505025	1.437644
H	-2.819579	-2.060705	3.386421
H	-2.987326	2.076341	2.136750
N	-0.532412	1.154084	-1.041370
N	-1.359269	-0.552229	0.761112
O	-1.715842	5.075644	-1.179357
O	-3.788651	0.462243	3.909052
C	-1.298766	5.957543	-2.203089
H	-1.758783	6.931173	-1.980847
H	-0.198384	6.066275	-2.209266
H	-1.645119	5.607484	-3.193238
C	-4.204068	-0.478828	4.878965
H	-4.828640	0.070799	5.597910
H	-4.802414	-1.286388	4.417961

H	-3.335141	-0.913392	5.407248
Ni	-0.100050	-0.776115	-0.824682
Br	-1.802986	-1.360105	-2.449973
C	1.507238	-0.227891	0.107148
C	2.565348	0.160319	-0.717056
C	3.710746	0.729930	-0.165095
C	3.817540	0.910883	1.220901
C	2.751261	0.506616	2.035188
C	1.603154	-0.060428	1.489305
H	2.516837	0.008448	-1.804717
H	4.526947	1.026328	-0.833995
H	2.841185	0.645030	3.118708
H	0.792663	-0.374029	2.158920
I	0.916433	-3.107199	-0.649620
C	5.019921	1.508400	1.867043
O	5.074147	1.649969	3.075282
C	6.164506	1.930754	0.986702
H	6.560579	1.074339	0.410167
H	5.843110	2.692964	0.252839
H	6.968730	2.348235	1.613389

ZnBrI(DMA)$_3$ (singlet)

Zn	-1.349912	-0.284682	0.198091
Br	-1.214745	-1.883927	1.956785
O	-0.231326	1.241466	0.899218
C	0.384245	2.195924	0.380835
O	2.509336	0.692479	-0.591122
C	3.609497	0.290413	-0.220763
O	-0.410454	-0.520679	-1.586359
C	0.652623	-0.809551	-2.172900
C	1.148120	0.086955	-3.268735
H	2.003071	0.683234	-2.901722
H	1.461612	-0.477015	-4.163949
H	0.334775	0.775991	-3.544876
C	4.861615	0.689773	-0.961302

H	5.440640	-0.187540	-1.303730
H	4.565299	1.287668	-1.838264
H	5.531502	1.302462	-0.329838
C	0.052348	2.685495	-0.994797
H	-0.681937	2.006393	-1.455488
H	-0.378663	3.703173	-0.955594
H	0.954575	2.720901	-1.629743
N	1.321083	-1.936628	-1.891295
N	3.755644	-0.526850	0.859358
N	1.334634	2.840946	1.075255
C	5.016950	-1.098857	1.277813
H	5.012973	-1.215784	2.376122
H	5.181897	-2.100587	0.828980
H	5.867878	-0.451807	1.017727
C	2.590774	-1.025946	1.556446
H	2.551419	-2.133081	1.498353
H	2.627735	-0.746341	2.627957
H	1.674899	-0.604217	1.111983
C	2.667010	-2.219287	-2.342779
H	3.323460	-2.378512	-1.463277
H	2.691622	-3.136632	-2.960626
H	3.081031	-1.386541	-2.928549
C	0.736253	-2.915011	-0.993956
H	1.177592	-3.900931	-1.217166
H	0.916844	-2.669966	0.068995
H	-0.353954	-2.970429	-1.148414
C	2.164055	3.875242	0.492906
H	2.567301	4.505186	1.303445
H	3.013831	3.433631	-0.067028
H	1.590041	4.527721	-0.182416
C	1.796094	2.320086	2.345660
H	2.823543	1.916280	2.237969
H	1.814296	3.125590	3.102226
H	1.126998	1.515694	2.682075

I	-3.713290	0.545916	-0.487982

XXIII (doublet)

E(UM06-doublet) = -2615.23234639

E(UM06-quadruplet) = -2615.18300890

C	1.969448	0.965175	-0.050986
C	0.024822	2.191085	-0.215821
C	0.709003	3.401639	-0.203537
C	2.105825	3.367331	-0.105247
C	2.739090	2.117039	-0.030896
C	2.536571	-0.400616	0.002230
C	2.049734	-2.655665	-0.052670
C	3.387071	-3.014459	0.058419
C	4.339495	-1.991529	0.149284
C	3.891576	-0.662274	0.119265
H	-1.070917	2.172746	-0.292151
H	0.149092	4.337936	-0.270767
H	3.830796	2.091128	0.036081
H	1.276672	-3.430818	-0.123269
H	3.659152	-4.072879	0.073489
H	4.634805	0.137314	0.190839
N	0.622399	1.005072	-0.136454
N	1.619093	-1.392942	-0.080810
O	2.895339	4.438725	-0.080050
O	5.652339	-2.177839	0.262652
C	2.316854	5.724302	-0.153926
H	3.147649	6.443921	-0.112503
H	1.764966	5.859675	-1.103121
H	1.635692	5.904958	0.698846
C	6.162801	-3.493637	0.295454
H	7.254993	-3.403069	0.388324
H	5.768691	-4.052934	1.164736
H	5.920566	-4.038886	-0.636138
Ni	-0.269754	-0.824055	-0.145560
C	-2.182389	-0.725333	-0.066421

C	-3.073422	-1.540953	-0.804760
C	-2.820830	0.215935	0.781693
C	-4.459510	-1.432608	-0.721354
H	-2.669189	-2.305145	-1.485992
C	-4.200756	0.342001	0.880524
H	-2.203192	0.885472	1.402057
C	-5.052494	-0.483039	0.126504
H	-5.085667	-2.099593	-1.328287
H	-4.656172	1.084329	1.548325
C	-6.517669	-0.317035	0.258123
O	-7.001010	0.521705	1.003336
C	-7.414622	-1.215170	-0.556329
H	-7.227121	-1.093046	-1.639246
H	-7.238599	-2.280466	-0.317953
H	-8.466480	-0.965880	-0.341552

XIV (singlet)

E(UM06-singlet) = -3066.90783218

E(UM06-triplet) = -3066.82923394

C	-0.712370	-0.055729	-0.000135
C	-2.658826	1.249599	0.000301
C	-3.506400	0.146491	0.000355
C	-2.896462	-1.134337	-0.000085
C	-1.517128	-1.229082	-0.000365
C	0.712371	-0.055728	0.000130
C	2.658826	1.249600	-0.000304
C	3.506400	0.146492	-0.000359
C	2.896463	-1.134336	0.000081
C	1.517129	-1.229080	0.000359
H	-3.092044	2.260305	0.000446
H	-4.589247	0.291320	0.000594
H	-1.066405	-2.227238	-0.000888
H	3.092045	2.260306	-0.000448
H	4.589248	0.291321	-0.000600
H	1.066406	-2.227237	0.000882

N	-1.330644	1.190543	0.000044
N	1.330644	1.190546	-0.000046
O	-3.591726	-2.291593	-0.000244
O	3.591727	-2.291592	0.000241
C	-4.994418	-2.246606	-0.000085
H	-5.345699	-3.290565	0.000171
H	-5.389197	-1.737808	0.901369
H	-5.389413	-1.738181	-0.901659
C	4.994418	-2.246606	0.000096
H	5.345699	-3.290565	-0.000145
H	5.389208	-1.737817	-0.901358
H	5.389404	-1.738173	0.901670
Ni	-0.000001	2.575647	0.000002

XV (singlet)

E(UM06-singlet) = -2806.30314479

E(UM06-triplet) = -2806.30314479

C	-0.648348	1.245032	-0.504164
C	1.672474	1.134340	-0.773755
C	1.782957	2.460994	-1.179559
C	0.577817	3.207631	-1.262504
C	-0.620699	2.602557	-0.933583
C	-1.818237	0.553973	-0.079967
C	-2.679582	-1.450705	0.760859
C	-3.984075	-0.969929	0.825391
C	-4.196218	0.367960	0.403788
C	-3.124044	1.119496	-0.041812
H	2.582228	0.515729	-0.708394
H	2.761705	2.876053	-1.430130
H	-1.538112	3.196821	-1.001823
H	-2.466540	-2.484849	1.072518
H	-4.787709	-1.615733	1.186467
H	-3.309128	2.151001	-0.359955
N	0.537788	0.529294	-0.446658
N	-1.634540	-0.753441	0.335911

O	0.528508	4.498306	-1.651753
O	-5.404873	0.967454	0.411682
C	1.721141	5.158362	-1.987824
H	1.446424	6.187977	-2.266208
H	2.424115	5.195548	-1.132558
H	2.226976	4.679137	-2.848922
C	-6.523868	0.244683	0.854986
H	-7.390237	0.919958	0.774491
H	-6.708881	-0.649739	0.228244
H	-6.415520	-0.072417	1.910827
Ni	0.288246	-1.312176	0.254289
O	1.984330	-1.339031	1.544156
O	0.257240	-3.325323	-0.037545
C	2.707648	-0.426145	1.966165
C	0.328613	-2.847342	-1.208426
C	4.202093	-0.539269	1.817299
H	4.427503	-1.512541	1.354912
H	4.606915	0.263708	1.173366
H	4.723878	-0.481321	2.789388
C	-0.905453	-2.690365	-2.062951
H	-1.793861	-2.862574	-1.436773
H	-0.986540	-1.683903	-2.512137
H	-0.902055	-3.431536	-2.886981
N	2.209590	0.679909	2.563512
N	1.538091	-2.793845	-1.871194
C	0.783900	0.871697	2.729682
H	0.448730	1.756570	2.151880
H	0.234057	-0.009085	2.364956
H	0.549327	1.038939	3.798211
C	3.006113	1.822932	2.956863
H	2.675258	2.176903	3.950230
H	4.075266	1.579696	3.024314
H	2.875892	2.655414	2.235839
C	2.761026	-2.997576	-1.128398

H	3.183236	-2.040671	-0.752559
H	2.559522	-3.640411	-0.259085
H	3.512385	-3.482637	-1.777921
C	1.669830	-2.037802	-3.096392
H	2.647482	-2.264876	-3.554596
H	0.892225	-2.307074	-3.830098
H	1.617910	-0.940948	-2.915103

XVI (singlet)

E(UM06-singlet) = -2913.02005748

E(UM06-triplet) = -2912.96580293

C	-2.432872	0.687914	-0.155256
C	-1.327631	2.561892	-0.930276
C	-2.507534	3.286466	-1.048635
C	-3.707830	2.657216	-0.695655
C	-3.655924	1.330379	-0.239340
C	-2.266619	-0.705311	0.315138
C	-0.741335	-2.391926	0.719203
C	-1.727828	-3.262315	1.166785
C	-3.053279	-2.810776	1.178381
C	-3.314858	-1.502428	0.741366
H	-0.368013	3.021860	-1.196463
H	-2.470375	4.317077	-1.410233
H	-4.593278	0.839033	0.037742
H	0.308318	-2.714044	0.688791
H	-1.448384	-4.266838	1.494319
H	-4.349316	-1.146782	0.752974
N	-1.275976	1.301663	-0.500010
N	-0.987171	-1.150233	0.302286
O	-4.910950	3.220226	-0.757155
O	-4.100561	-3.528010	1.575323
C	-5.028506	4.551884	-1.212556
H	-6.099661	4.799265	-1.181175
H	-4.474330	5.248174	-0.555636
H	-4.661808	4.651272	-2.251403

C	-3.901204	-4.851335	2.026235
H	-4.893831	-5.243844	2.291274
H	-3.460678	-5.481197	1.230764
H	-3.250066	-4.874127	2.920185
Ni	0.307706	0.170498	-0.306995
I	2.560116	-2.136730	-1.493616
C	2.062429	-0.390260	-0.225755
C	2.003832	0.927705	-0.880165
C	2.632287	-0.450511	1.099946
C	2.493906	2.054316	-0.167215
H	1.975897	1.004333	-1.979688
C	3.023747	0.680616	1.759265
H	2.725172	-1.426573	1.596780
C	2.969068	1.973019	1.130095
H	2.528964	3.036026	-0.659264
H	3.396122	0.585879	2.786336
C	3.424256	3.208794	1.792738
O	3.372526	4.301103	1.241863
C	3.967275	3.097414	3.196267
H	3.210321	2.680333	3.886729
H	4.842508	2.422240	3.235796
H	4.266122	4.097585	3.550103

TS-XVI-VI (singlet)

E(UM06-singlet) = -2913.01339538

E(UM06-triplet) = -2912.97674499

C	2.453569	-0.409424	0.368006
C	1.052823	-2.221517	0.708045
C	2.105796	-3.032513	1.111439
C	3.395590	-2.486579	1.134637
C	3.559612	-1.145178	0.750784
C	2.507152	1.007975	-0.045814
C	1.234811	2.820987	-0.711180
C	2.351560	3.636723	-0.836718
C	3.607826	3.088166	-0.546361

C	3.673394	1.744518	-0.140903
H	0.028528	-2.615785	0.653116
H	1.903490	-4.068099	1.395613
H	4.565500	-0.715986	0.770002
H	0.234011	3.214654	-0.927033
H	2.224301	4.674223	-1.155081
H	4.653943	1.315904	0.085658
N	1.209973	-0.947947	0.346481
N	1.297536	1.545394	-0.330652
O	4.493237	-3.140594	1.494941
O	4.760106	3.742092	-0.623397
C	4.395732	-4.492772	1.894791
H	3.753474	-4.597008	2.789083
H	3.998396	-5.121431	1.076274
H	5.416616	-4.819132	2.140801
C	4.766105	5.094346	-1.034867
H	5.817173	5.417344	-1.027114
H	4.359208	5.199459	-2.057804
H	4.185317	5.724838	-0.336263
Ni	-0.185038	0.287031	-0.128462
I	-2.093350	-2.570022	-1.524333
C	-1.853730	-0.326133	0.080553
C	-2.066202	0.886670	-0.653249
C	-2.623753	-0.595823	1.235932
C	-3.009860	1.822383	-0.170080
H	-1.745905	0.986912	-1.703792
C	-3.498411	0.359449	1.699655
H	-2.478980	-1.532887	1.789664
C	-3.710505	1.583344	1.004585
H	-3.216854	2.719170	-0.767293
H	-4.048958	0.202977	2.634711
C	-4.695561	2.544447	1.557702
O	-5.300117	2.300660	2.588924
C	-4.931921	3.835738	0.818055

H	-5.666086	4.439583	1.375217
H	-3.996562	4.414154	0.706106
H	-5.320528	3.647192	-0.199869

XVII (singlet)

E(UM06-singlet) = -2913.07067642

E(UM06-triplet) = -2913.02463755

C	-1.623597	1.723836	0.013979
C	0.617127	2.266075	0.044478
C	0.352012	3.627612	0.047486
C	-0.982394	4.047681	0.031078
C	-1.978878	3.061103	0.014972
C	-2.613163	0.632916	0.001728
C	-2.918661	-1.648369	0.013148
C	-4.301494	-1.528324	-0.011394
C	-4.856294	-0.243852	-0.031948
C	-3.979722	0.851483	-0.024310
H	1.656426	1.921768	0.056603
H	1.188994	4.330084	0.062271
H	-3.023878	3.382393	0.004783
H	-2.454057	-2.642557	0.028166
H	-4.914831	-2.432800	-0.014770
H	-4.408784	1.856722	-0.040720
N	-0.327443	1.318205	0.027195
N	-2.079330	-0.611007	0.019244
O	-1.387767	5.310640	0.030271
O	-6.155119	0.028994	-0.058374
C	-0.425718	6.346599	0.044315
H	-0.986435	7.292465	0.039304
H	0.198087	6.296192	0.955983
H	0.220379	6.299773	-0.851901
C	-7.085342	-1.035061	-0.070410
H	-8.084142	-0.575629	-0.094377
H	-6.952781	-1.667941	-0.967546
H	-6.990610	-1.655885	0.839873

Ni	-0.076783	-0.612608	0.022536
I	0.275486	-3.107937	0.036417
C	1.784937	-0.453636	-0.012383
C	2.461560	-0.305675	-1.236709
C	2.545144	-0.351760	1.163943
C	3.823955	-0.037791	-1.281863
H	1.905684	-0.391813	-2.181937
C	3.911368	-0.083930	1.126109
H	2.059085	-0.476262	2.142352
C	4.573835	0.082244	-0.100419
H	4.343153	0.086272	-2.239848
H	4.463881	-0.003800	2.070432
C	6.025361	0.374043	-0.202108
O	6.567949	0.517090	-1.285468
C	6.820730	0.490186	1.072296
H	6.425346	1.295204	1.719090
H	6.774177	-0.446079	1.658777
H	7.871784	0.709092	0.823936

IIa (singlet)

E(UM06-singlet) = -963.385476203

E(UM06-triplet) = -963.260252869

C	-2.194581	-0.698479	-0.032771
C	-2.187838	0.699919	-0.034525
C	-3.356133	1.428792	0.106230
C	-4.550219	0.711338	0.248097
C	-4.556958	-0.686002	0.250602
C	-3.369913	-1.415522	0.110710
C	-0.802364	-1.189018	-0.194868
C	-0.790849	1.176987	-0.195895
H	-3.341346	2.524048	0.104508
H	-5.495798	1.252804	0.359181
H	-5.507684	-1.217907	0.363946
H	-3.365778	-2.510870	0.112751
O	-0.361704	-2.305488	-0.260079

O	-0.339656	2.289105	-0.261712
N	-0.039122	-0.009828	-0.247724
O	1.275573	-0.015542	-0.547868
C	2.073828	-0.037298	0.621797
H	1.876973	0.845846	1.258369
H	1.908253	-0.963478	1.203904
C	3.507952	0.003255	0.160544
F	3.800649	-1.031829	-0.619876
F	3.769838	1.111762	-0.524347
F	4.314844	-0.032164	1.216351

IVa (doublet)

E(UM06-doublet) = -5768.73091080

E(UM06-quadruplet) = -5768.72362147

C	3.082003	-0.169344	0.060186
C	3.232490	-2.213409	-1.013607
C	4.619999	-2.201444	-0.981531
C	5.256163	-1.097305	-0.397767
C	4.460178	-0.068173	0.132493
C	2.160055	0.835001	0.642674
C	-0.036373	1.287925	1.204999
C	0.288500	2.513060	1.768353
C	1.630103	2.917478	1.729311
C	2.579094	2.042617	1.173944
H	2.695139	-3.061783	-1.455634
H	5.175446	-3.042437	-1.403503
H	4.959353	0.780573	0.608546
H	-1.079198	0.942590	1.205660
H	-0.500480	3.128528	2.207585
H	3.627682	2.353006	1.163311
N	2.481181	-1.231312	-0.520315
N	0.857154	0.479433	0.640338
O	6.569672	-0.945391	-0.292908
O	2.085861	4.076939	2.184464
C	7.428532	-1.955664	-0.783660

H	8.454855	-1.614730	-0.584990
H	7.253446	-2.913355	-0.259263
H	7.294578	-2.096662	-1.872209
C	1.173816	5.027112	2.698926
H	1.767588	5.910040	2.976332
H	0.428716	5.312331	1.932334
H	0.656880	4.638422	3.595703
Ni	0.450599	-1.139951	-0.519325
Br	-0.255282	-2.954292	0.868760
O	-1.151748	-0.630444	-1.486989
C	-1.740821	0.482768	-1.389888
C	-1.298763	1.834196	-1.546991
C	-2.368396	2.700873	-1.189223
C	-0.052588	2.362877	-1.921062
C	-3.532904	1.905046	-0.803096
C	-2.199821	4.085341	-1.200027
C	0.101797	3.740625	-1.927149
H	0.777588	1.696886	-2.190447
C	-0.960985	4.600996	-1.568081
H	-3.029782	4.744459	-0.917854
H	1.066562	4.174897	-2.214776
H	-0.803208	5.685365	-1.582646
O	-4.648517	2.214404	-0.416459
N	-3.070400	0.586641	-0.961762
O	-3.843356	-0.499992	-0.745459
C	-3.639635	-1.018943	0.554800
H	-4.036512	-0.332299	1.328205
H	-2.567999	-1.233160	0.741489
C	-4.382605	-2.326016	0.622179
F	-5.691279	-2.164311	0.439234
F	-3.953018	-3.181669	-0.299987
F	-4.199306	-2.880131	1.817744

XI/VII (doublet)

E(UM06-doublet) = -451.565313205

E(UM06-quadruplet) = -451.251311269

C	0.982677	0.677066	0.000062
H	1.060652	1.368028	-0.876036
H	1.060676	1.367928	0.876244
O	2.005413	-0.187161	-0.000023
C	-0.379214	0.010370	0.000006
F	-0.536493	-0.759434	-1.073846
F	-0.536292	-0.760034	1.073469
F	-1.347815	0.923549	0.000329

TS-XI-XI-a/ TS-X-XI (doublet)

E(UM06-doublet) = -738.974882095

E(UM06-quadruplet) = -738.793109892

C	-1.397933	0.874685	-0.024612
H	0.048960	-0.108284	0.432723
H	-1.208804	1.527463	-0.910978
O	-1.124943	1.243279	1.186466
C	-2.491638	-0.101414	-0.233993
F	-2.336649	-1.215856	0.509079
F	-3.726363	0.357837	0.096118
F	-2.575599	-0.488814	-1.515895
O	0.734484	-0.853901	0.590639
C	1.951256	-0.690993	0.205039
C	2.769150	-1.932504	0.182980
H	2.212540	-2.729024	0.698414
H	2.950001	-2.257022	-0.858618
H	3.747032	-1.787263	0.671038
N	2.462279	0.472296	-0.135007
C	3.825248	0.586208	-0.645881
H	3.853941	1.426015	-1.358088
H	4.530974	0.793716	0.178992
H	4.134447	-0.323743	-1.178549
C	1.768307	1.747581	0.025854
H	1.468834	2.136863	-0.963962
H	0.875907	1.660920	0.670898

H	2.475059	2.459375	0.486285

XI-a/VIII (doublet)

E(UM06-doublet) = -739.017867967

E(UM06-quadruplet) = -738.824341736

C	2.144040	-0.899177	0.634965
H	0.505668	-0.291358	1.518501
H	3.066921	-1.477720	0.748852
O	1.330843	-0.849647	1.687195
C	2.088876	0.145638	-0.407209
F	0.949648	0.118660	-1.130328
F	2.151438	1.393989	0.093255
F	3.099166	0.004103	-1.263142
O	-0.771639	0.620742	1.254506
C	-1.730366	0.589315	0.472165
C	-2.504063	1.850887	0.203186
H	-2.114305	2.635597	0.869065
H	-2.373276	2.184794	-0.842356
H	-3.585908	1.727745	0.385822
N	-2.109863	-0.550551	-0.143418
C	-3.144295	-0.643144	-1.152480
H	-2.720853	-1.043219	-2.093164
H	-3.950039	-1.325207	-0.821073
H	-3.587996	0.337559	-1.371046
C	-1.380797	-1.776527	0.118510
H	-0.475474	-1.854469	-0.516575
H	-1.073347	-1.825985	1.174950
H	-2.038628	-2.635362	-0.095541

XXIV (singlet)

E(UM06-singlet) = -3066.90783218

E(UM06-triplet) = -3066.82923394

C	-1.644681	1.713366	0.067792
C	0.587965	2.267652	0.152118
C	0.318892	3.628149	0.091180
C	-1.016280	4.038955	0.013674

C	-2.008415	3.048034	0.003160
C	-2.626730	0.613740	0.062864
C	-2.942275	-1.656463	0.209586
C	-4.314890	-1.546987	0.033523
C	-4.861073	-0.269740	-0.130652
C	-3.983949	0.824014	-0.114516
H	1.627037	1.924992	0.214141
H	1.151023	4.336429	0.107005
H	-3.053598	3.364451	-0.049920
H	-2.505867	-2.652012	0.335416
H	-4.925078	-2.453440	0.027237
H	-4.403175	1.823859	-0.254807
N	-0.351617	1.317267	0.140353
N	-2.098946	-0.622884	0.232629
O	-1.427255	5.300440	-0.048552
O	-6.150147	-0.004175	-0.306905
C	-0.470612	6.340337	-0.039122
H	-1.033846	7.282927	-0.099914
H	0.120717	6.328245	0.895498
H	0.208113	6.264063	-0.908970
C	-7.077080	-1.070313	-0.342011
H	-8.067511	-0.617472	-0.494716
H	-6.858245	-1.759763	-1.178673
H	-7.075196	-1.630557	0.611549
Ni	-0.083030	-0.643209	0.351001
C	1.767956	-0.503759	0.192491
C	2.654157	-0.360161	1.273400
C	2.309623	-0.338630	-1.098504
C	3.996367	-0.040993	1.085572
H	2.286407	-0.507714	2.297033
C	3.648991	-0.030629	-1.297756
H	1.658868	-0.448552	-1.979144
C	4.518877	0.133039	-0.206611
H	4.644106	0.073211	1.963974

H	4.054873	0.098211	-2.308667
C	5.937002	0.475643	-0.465195
O	6.361957	0.614944	-1.601401
C	6.855545	0.651761	0.717427
H	6.495848	1.453968	1.388097
H	6.910547	-0.272235	1.322295
H	7.864873	0.907427	0.356464
C	0.110015	-2.501852	0.823236
H	-0.875305	-2.784357	1.257567
C	0.281166	-3.403176	-0.361655
O	1.118042	-2.800395	1.769608
H	0.986813	-3.715074	2.076414
F	0.228691	-4.713599	-0.011102
F	1.442679	-3.259404	-1.001377
F	-0.693240	-3.229869	-1.269611

TS-XXIV-XV (singlet)

C	-2.043697	1.474616	-0.085546
C	0.120002	2.281326	-0.195832
C	-0.307876	3.602574	-0.229684
C	-1.684934	3.853953	-0.186830
C	-2.560235	2.758616	-0.113135
C	-2.880134	0.255232	-0.007120
C	-2.861424	-2.047725	0.072170
C	-4.245294	-2.124201	0.172747
C	-4.970997	-0.926365	0.175163
C	-4.261560	0.281934	0.084933
H	1.192089	2.039146	-0.231699
H	0.432047	4.404779	-0.289025
H	-3.635915	2.954941	-0.082297
H	-2.265449	-2.970984	0.068378
H	-4.726116	-3.102910	0.245975
H	-4.828037	1.217701	0.095142
N	-0.710646	1.241664	-0.124157
N	-2.185383	-0.905120	-0.021196

O	-2.241767	5.061404	-0.212006
O	-6.294801	-0.836454	0.260517
C	-1.414385	6.203253	-0.289880
H	-2.085339	7.074648	-0.301220
H	-0.744532	6.270939	0.587805
H	-0.811151	6.195890	-1.217054
C	-7.063443	-2.017496	0.355387
H	-8.115458	-1.701780	0.411385
H	-6.922033	-2.657370	-0.535737
H	-6.805565	-2.588745	1.266824
Ni	-0.187598	-0.620716	-0.086198
C	1.632606	-0.635744	0.077731
C	2.274959	-0.310574	1.318827
C	2.364034	-0.262034	-1.105508
C	3.502354	0.316712	1.367051
H	1.770787	-0.571185	2.257075
C	3.586822	0.364699	-1.040981
H	1.925874	-0.470523	-2.091087
C	4.209768	0.669840	0.193671
H	3.927886	0.550183	2.352057
H	4.103859	0.662937	-1.962165
C	5.507665	1.336093	0.198622
O	6.100809	1.630143	-0.838368
C	6.134502	1.671228	1.534965
H	5.485430	2.337094	2.134022
H	6.309001	0.762770	2.141349
H	7.099803	2.174998	1.361648
C	0.911206	-2.292674	0.153621
H	-0.041111	-2.511788	-0.411841
C	1.903940	-3.179604	-0.550175
O	0.810648	-2.662627	1.496942
H	0.489634	-3.580499	1.554032
F	1.587804	-4.473940	-0.330656
F	3.158816	-3.040994	-0.130730

F	1.880809	-3.001474	-1.869773

TS-XXIV-P (singlet)

E(UM06-singlet) = -3066.86696585

E(UM06-triplet) = -3066.82131334

C	-2.043697	1.474616	-0.085546
C	0.120002	2.281326	-0.195832
C	-0.307876	3.602574	-0.229684
C	-1.684934	3.853953	-0.186830
C	-2.560235	2.758616	-0.113135
C	-2.880134	0.255232	-0.007120
C	-2.861424	-2.047725	0.072170
C	-4.245294	-2.124201	0.172747
C	-4.970997	-0.926365	0.175163
C	-4.261560	0.281934	0.084933
H	1.192089	2.039146	-0.231699
H	0.432047	4.404779	-0.289025
H	-3.635915	2.954941	-0.082297
H	-2.265449	-2.970984	0.068378
H	-4.726116	-3.102910	0.245975
H	-4.828037	1.217701	0.095142
N	-0.710646	1.241664	-0.124157
N	-2.185383	-0.905120	-0.021196
O	-2.241767	5.061404	-0.212006
O	-6.294801	-0.836454	0.260517
C	-1.414385	6.203253	-0.289880
H	-2.085339	7.074648	-0.301220
H	-0.744532	6.270939	0.587805
H	-0.811151	6.195890	-1.217054
C	-7.063443	-2.017496	0.355387
H	-8.115458	-1.701780	0.411385
H	-6.922033	-2.657370	-0.535737
H	-6.805565	-2.588745	1.266824
Ni	-0.187598	-0.620716	-0.086198
C	1.632606	-0.635744	0.077731

C	2.274959	-0.310574	1.318827
C	2.364034	-0.262034	-1.105508
C	3.502354	0.316712	1.367051
H	1.770787	-0.571185	2.257075
C	3.586822	0.364699	-1.040981
H	1.925874	-0.470523	-2.091087
C	4.209768	0.669840	0.193671
H	3.927886	0.550183	2.352057
H	4.103859	0.662937	-1.962165
C	5.507665	1.336093	0.198622
O	6.100809	1.630143	-0.838368
C	6.134502	1.671228	1.534965
H	5.485430	2.337094	2.134022
H	6.309001	0.762770	2.141349
H	7.099803	2.174998	1.361648
C	0.911206	-2.292674	0.153621
H	-0.041111	-2.511788	-0.411841
C	1.903940	-3.179604	-0.550175
O	0.810648	-2.662627	1.496942
H	0.489634	-3.580499	1.554032
F	1.587804	-4.473940	-0.330656
F	3.158816	-3.040994	-0.130730
F	1.880809	-3.001474	-1.869773

P (^{1}S)

F	-4.051263	0.356627	-0.190481
C	-2.741698	0.322929	-0.414002
F	-2.363610	1.541597	-0.797198
F	-2.526621	-0.508582	-1.430323
C	-1.981092	-0.099836	0.833711
O	-2.492847	-1.352690	1.172152
H	-2.225964	0.672764	1.595781
C	-0.492403	-0.095074	0.583942
C	0.261803	1.053691	0.830893
C	0.134490	-1.234821	0.069262

C	1.628295	1.067580	0.569160
H	-0.227015	1.948049	1.236766
C	1.498750	-1.221675	-0.187610
H	-0.461525	-2.133825	-0.123742
C	2.261352	-0.072117	0.057238
H	2.199016	1.979586	0.774714
H	2.004024	-2.106681	-0.588808
C	3.726400	-0.116499	-0.239869
O	4.237641	-1.130533	-0.674556
C	4.540893	1.121119	0.011557
H	5.590004	0.926436	-0.262198
H	4.162165	1.974174	-0.581364
H	4.492261	1.419902	1.075108
H	-2.038604	-1.673170	1.969299

XXV (doublet)

E(UM06-doublet) = -3364.69645514

E(UM06-quadruplet) = -3364.60418287

C	-1.835159	1.653757	-0.330129
C	0.292862	2.384647	0.180436
C	-0.115439	3.705269	0.292792
C	-1.466829	4.000948	0.075773
C	-2.332010	2.943468	-0.240416
C	-2.685542	0.487508	-0.639255
C	-2.797968	-1.803830	-0.850380
C	-4.141191	-1.771780	-1.199210
C	-4.780477	-0.529403	-1.262929
C	-4.023319	0.614967	-0.972552
H	1.344130	2.124192	0.353120
H	0.620226	4.471588	0.548600
H	-3.388861	3.172695	-0.399276
H	-2.273272	-2.759273	-0.763264
H	-4.659565	-2.710621	-1.408770
H	-4.518937	1.587679	-1.027138
N	-0.527994	1.378691	-0.127016

N	-2.071572	-0.718036	-0.576438
O	-2.004287	5.211577	0.149306
O	-6.055074	-0.339516	-1.579148
C	-1.183689	6.316492	0.472869
H	-1.838644	7.199738	0.477438
H	-0.729303	6.193400	1.473428
H	-0.388597	6.455245	-0.283031
C	-6.867227	-1.456394	-1.881604
H	-7.867658	-1.061242	-2.110056
H	-6.479914	-2.002049	-2.762044
H	-6.934330	-2.143842	-1.018015
Ni	-0.054498	-0.600289	-0.129812
I	-0.370170	-0.625090	2.586623
C	1.784897	-0.210685	-0.344095
C	2.085516	0.222001	-1.642788
C	2.770069	-0.152085	0.640042
C	3.350487	0.705248	-1.950258
H	1.321332	0.187717	-2.433865
C	4.041265	0.331680	0.330212
H	2.551734	-0.486469	1.661010
C	4.348685	0.765320	-0.966364
H	3.592926	1.048214	-2.962653
H	4.798821	0.367399	1.122140
C	5.690532	1.287657	-1.346405
O	5.917404	1.661234	-2.483491
C	6.759959	1.340978	-0.289027
H	6.450397	1.978752	0.559479
H	6.964391	0.334984	0.121872
H	7.684849	1.747170	-0.728872
C	0.373827	-2.473884	-0.516419
H	0.107482	-2.495078	-1.602802
C	1.736289	-3.122007	-0.421866
O	-0.501386	-3.248663	0.255105
H	-0.489861	-4.169006	-0.068729

F	1.609724	-4.444926	-0.690443
F	2.616893	-2.660964	-1.304744
F	2.283168	-3.054575	0.790142

TS-XXV-P (doublet)

C	2.051243	1.242993	-0.263564
C	0.224639	2.635264	-0.085098
C	0.981921	3.770366	-0.347275
C	2.350031	3.609477	-0.594618
C	2.885710	2.313764	-0.537227
C	2.543241	-0.143140	-0.124164
C	1.972110	-2.305674	0.450586
C	3.255389	-2.777887	0.217477
C	4.225073	-1.863659	-0.216797
C	3.849068	-0.522544	-0.386550
H	-0.842425	2.731615	0.139276
H	0.495834	4.748997	-0.349441
H	3.960302	2.188380	-0.696249
H	1.181757	-2.993819	0.775788
H	3.473401	-3.837521	0.370013
H	4.604276	0.188537	-0.732754
N	0.725363	1.403216	-0.063942
N	1.619764	-1.031936	0.291320
O	3.197906	4.595214	-0.867420
O	5.489036	-2.167582	-0.488310
C	2.718722	5.922941	-0.927944
H	3.583790	6.555888	-1.173797
H	1.950327	6.032775	-1.715812
H	2.300088	6.239680	0.045570
C	5.928346	-3.502702	-0.342827
H	6.992686	-3.512431	-0.619254
H	5.819830	-3.845103	0.703161
H	5.370377	-4.180214	-1.015724
Ni	-0.301296	-0.387282	0.248242

I	-0.399097	-1.240811	-2.260688
C	-2.216704	-0.189323	0.142495
C	-2.846350	0.914790	-0.445868
C	-3.020909	-1.236685	0.611029
C	-4.231535	0.979439	-0.538177
H	-2.254417	1.733874	-0.872306
C	-4.409344	-1.180603	0.509765
H	-2.564556	-2.128288	1.064997
C	-5.037457	-0.064493	-0.060001
H	-4.720953	1.846243	-0.997664
H	-5.001344	-2.023031	0.886784
C	-6.513790	0.057795	-0.184073
O	-7.023300	1.044857	-0.686003
C	-7.367482	-1.071752	0.328185
H	-7.129956	-2.018702	-0.191026
H	-7.196355	-1.244043	1.406966
H	-8.429115	-0.826218	0.164948
C	-0.616129	0.009306	2.173446
H	-1.388838	-0.702672	2.522617
C	0.571861	-0.244221	3.066394
O	-1.001413	1.340899	2.337038
H	-1.725445	1.400955	2.982312
F	0.269268	0.063304	4.340606
F	0.921396	-1.534907	3.066837
F	1.654839	0.474401	2.763892

XVIII (doublet)

C	-0.437021	1.076321	0.457696
C	-1.824883	0.968978	0.596175
C	-2.435935	-0.263142	0.366895
C	-1.702598	-1.389942	0.002656
C	-0.312833	-1.287535	-0.131069
C	0.265414	-0.056719	0.099238
H	0.063339	2.037814	0.634996

H	-2.437648	1.831235	0.884791
H	-2.229089	-2.337065	-0.163893
H	0.284376	-2.165868	-0.410515
I	3.612938	0.048541	-0.062856
O	-3.801716	-0.412221	0.555705
C	-4.660676	0.200538	-0.296345
O	-4.300062	0.869785	-1.225344
C	-6.071965	-0.093347	0.094054
H	-6.251540	-1.182417	0.059746
H	-6.251536	0.230959	1.133944
H	-6.766388	0.421257	-0.586399

XIX (doublet)

C	0.741129	-0.062540	-0.000004
C	2.646526	1.246268	0.000052
C	3.486155	0.139919	0.000044
C	2.901251	-1.132445	-0.000005
C	1.500609	-1.220266	-0.000032
C	-0.741118	-0.062542	-0.000013
C	-2.646524	1.246252	-0.000052
C	-3.486144	0.139896	-0.000029
C	-2.901233	-1.132463	0.000008
C	-1.500590	-1.220272	0.000015
H	3.071510	2.257033	0.000076
H	4.568971	0.287930	0.000066
H	1.046922	-2.215430	-0.000081
H	-3.071518	2.257012	-0.000076
H	-4.568962	0.287900	-0.000034
H	-1.046896	-2.215434	0.000053
N	1.314599	1.166225	0.000028
N	-1.314594	1.166226	-0.000040
O	3.571065	-2.281606	-0.000028
O	-3.571037	-2.281629	0.000041
C	4.982952	-2.258987	-0.000021
H	5.312202	-3.308345	-0.000048

H	5.373768	-1.754898	-0.903829
H	5.373763	-1.754945	0.903815
C	-4.982924	-2.259023	0.000024
H	-5.312164	-3.308385	0.000051
H	-5.373753	-1.754936	0.903827
H	-5.373732	-1.754988	-0.903817
Ni	-0.000032	2.593337	0.000002

XX (doublet)

E(UM06-doublet) = -458.787540994

E(UM06-quadruplet) = -458.623098448

C	-2.449322	1.105954	0.316530
C	-1.082414	1.293314	0.077861
C	-0.298711	0.206078	-0.302399
C	-0.835219	-1.072940	-0.448828
C	-2.198221	-1.274051	-0.207342
C	-2.937490	-0.172040	0.163483
H	-3.083239	1.950909	0.611753
H	-0.614678	2.279437	0.176735
H	-0.187025	-1.902232	-0.754141
H	-2.639547	-2.272167	-0.316947
O	1.033910	0.442907	-0.589482
C	1.993552	-0.105433	0.200893
O	1.744481	-0.789816	1.154368
C	3.348613	0.279737	-0.292699
H	4.123797	-0.174807	0.341636
H	3.475985	-0.048564	-1.339059
H	3.452852	1.378983	-0.284058

XXI (doublet)

E(UM06-doublet) = -2529.34110530

E(UM06-quadruplet) = -2529.28749448

C	1.683770	0.346267	0.000005
C	1.099965	2.580307	0.000039

C	2.426081	2.993459	0.000016
C	3.424842	2.011011	-0.000016
C	3.031726	0.663960	-0.000015
C	1.168804	-1.040939	-0.000005
C	-0.737617	-2.342069	0.000069
C	-0.008725	-3.525233	0.000051
C	1.389426	-3.438170	-0.000009
C	1.979028	-2.164410	-0.000032
H	0.291485	3.321729	0.000058
H	2.654552	4.062257	0.000019
H	3.810056	-0.104943	-0.000036
H	-1.835419	-2.365888	0.000108
H	-0.535938	-4.482631	0.000078
H	3.071081	-2.097472	-0.000072
N	0.721962	1.300602	0.000042
N	-0.180402	-1.132579	0.000028
O	4.733938	2.252811	-0.000051
O	2.218454	-4.479589	-0.000034
C	5.188318	3.589184	0.000010
H	6.287359	3.546022	-0.000061
H	4.845143	4.127503	-0.903488
H	4.845252	4.127379	0.903625
C	1.685342	-5.786740	-0.000013
H	2.543759	-6.474422	-0.000039
H	1.073564	-5.968372	0.903628
H	1.073508	-5.968376	-0.903615
Ni	-1.107369	0.622091	0.000015
I	-3.594602	0.833527	-0.000020

VII (singlet)

C	-2.194581	-0.698479	-0.032771
C	-2.187838	0.699919	-0.034525
C	-3.356133	1.428792	0.106230
C	-4.550219	0.711338	0.248097
C	-4.556958	-0.686002	0.250602

C	-3.369913	-1.415522	0.110710
C	-0.802364	-1.189018	-0.194868
C	-0.790849	1.176987	-0.195895
H	-3.341346	2.524048	0.104508
H	-5.495798	1.252804	0.359181
H	-5.507684	-1.217907	0.363946
H	-3.365778	-2.510870	0.112751
O	-0.361704	-2.305488	-0.260079
O	-0.339656	2.289105	-0.261712
N	-0.039122	-0.009828	-0.247724
O	1.275573	-0.015542	-0.547868
C	2.073828	-0.037298	0.621797
H	1.876973	0.845846	1.258369
H	1.908253	-0.963478	1.203904
C	3.507952	0.003255	0.160544
F	3.800649	-1.031829	-0.619876
F	3.769838	1.111762	-0.524347
F	4.314844	-0.032164	1.216351

VIII/IX (singlet)

C	3.722870	0.445927	-0.131303
C	3.043739	-0.715987	0.252222
C	3.718782	-1.845578	0.680291
C	5.117601	-1.781049	0.712825
C	5.796666	-0.621944	0.330796
C	5.103703	0.517779	-0.098917
C	2.728654	1.469453	-0.527443
C	1.593457	-0.481921	0.119693
H	3.178192	-2.749399	0.979977
H	5.689674	-2.654063	1.043753
H	6.890919	-0.605045	0.367851
H	5.629803	1.429957	-0.399204
O	2.847561	2.586214	-0.941867
O	0.679281	-1.261979	0.322144
N	1.476937	0.830813	-0.289790

O	0.307376	1.375640	-0.676859
C	-0.221447	2.242520	0.321115
H	-0.456744	1.684462	1.248598
H	0.475434	3.073342	0.534079
C	-1.500449	2.808627	-0.240447
F	-1.280317	3.511432	-1.346770
F	-2.374364	1.852961	-0.536861
F	-2.054903	3.612608	0.660276
Zn	-1.427360	-1.284481	0.163723
Br	-2.183465	-0.492689	2.208485
Br	-1.897506	-1.852439	-2.031009

X (doublet)

C	-3.940770	-1.597883	-0.511909
C	-2.693661	-2.209617	-0.348252
C	-2.553364	-3.586898	-0.359660
C	-3.715626	-4.347527	-0.537929
C	-4.961476	-3.736752	-0.703287
C	-5.092513	-2.342274	-0.695127
C	-3.761071	-0.129324	-0.447975
C	-1.668386	-1.158944	-0.183392
H	-1.572172	-4.057030	-0.232898
H	-3.648585	-5.440476	-0.548666
H	-5.850680	-4.361132	-0.840076
H	-6.065180	-1.855857	-0.823989
O	-4.550774	0.771551	-0.474394
O	-0.486964	-1.280747	0.064220
N	-2.353352	0.037559	-0.343860
O	-1.817632	1.213667	0.035890
C	-1.542948	2.098703	-1.045939
H	-0.475207	2.045203	-1.333492
H	-2.183084	1.870652	-1.915953
C	-1.855700	3.489056	-0.555581
F	-3.151474	3.649601	-0.302825
F	-1.194936	3.778179	0.561951

F	-1.504249	4.373996	-1.485149
Zn	1.357469	-0.009707	-0.096265
Br	1.014624	-0.294920	-2.541411
Br	2.046009	2.328306	-0.005644
O	2.537514	-1.664762	0.146884
O	1.049128	0.002195	2.017304
C	0.155415	-0.585944	2.652183
C	3.710487	-1.916061	-0.195990
C	0.216551	-2.074185	2.852896
H	1.036724	-2.464709	2.230413
H	0.421029	-2.320325	3.911970
H	-0.724547	-2.577098	2.566363
N	-0.876228	0.095450	3.183830
C	-1.964800	-0.525949	3.910984
H	-1.653863	-1.465996	4.390050
H	-2.302565	0.160558	4.706650
H	-2.827244	-0.730762	3.244492
C	-0.986703	1.531785	3.005658
H	-0.202238	1.884103	2.319951
H	-1.972968	1.781652	2.573643
H	-0.887859	2.042513	3.982910
C	3.959822	-2.966199	-1.238374
H	3.007715	-3.479734	-1.441030
H	4.305083	-2.500161	-2.180435
H	4.717765	-3.705519	-0.926589
N	4.756276	-1.277660	0.349513
C	4.567441	-0.332346	1.435357
H	4.723062	0.704900	1.082649
H	3.548171	-0.418493	1.842201
H	5.301629	-0.549885	2.232525
C	6.136020	-1.500608	-0.035990
H	6.212582	-1.949984	-1.036104
H	6.660486	-0.529215	-0.061533
H	6.652307	-2.153372	0.694382

X (doublet)

C	3.752647	0.800648	-0.105374
C	3.306052	-0.520233	-0.107793
C	4.179648	-1.585630	-0.246151
C	5.540148	-1.281099	-0.383979
C	5.988967	0.043333	-0.380644
C	5.093952	1.112338	-0.238650
C	2.542409	1.672485	0.076318
C	1.829131	-0.454786	0.059797
H	3.821268	-2.620928	-0.246466
H	6.267434	-2.092759	-0.496676
H	7.060140	0.245358	-0.492164
H	5.438270	2.152683	-0.234730
O	2.508852	2.882473	0.059207
O	1.056260	-1.424174	-0.007096
N	1.462928	0.821706	0.283226
O	-0.515871	0.965677	-0.660629
C	-0.986268	2.100134	-0.034207
H	-1.103502	1.978839	1.065276
H	-0.335055	2.975488	-0.230295
C	-2.352764	2.431201	-0.593357
F	-2.312610	2.661427	-1.904458
F	-3.223155	1.441466	-0.390812
F	-2.837675	3.523496	-0.004214
Zn	-0.931919	-0.888366	0.114548
Br	-1.449281	-0.615825	2.421664
Br	-2.145804	-2.117869	-1.492985

XII (singlet)

C	2.916743	0.532619	0.419967
C	2.904689	-0.591359	-0.398420
C	4.077306	-1.174987	-0.848572
C	5.283320	-0.587391	-0.446655
C	5.295517	0.543792	0.376574
C	4.102181	1.123374	0.825265

C	1.489936	0.883550	0.706657
C	1.470308	-0.946920	-0.637836
H	4.057660	-2.061008	-1.493722
H	6.234564	-1.017317	-0.780578
H	6.256086	0.980847	0.672372
H	4.101973	2.009972	1.469969
O	1.099224	1.801736	1.401045
O	1.060688	-1.866982	-1.318723
N	0.695991	-0.032189	0.046547
Zn	-1.260286	0.001817	0.037684
Br	-2.500278	-1.953736	0.488937
Br	-2.363520	2.013286	-0.545075

XI' (doublet)

C	3.994687	1.410715	-0.571182
C	3.460875	0.135793	-0.939353
C	4.314802	-0.968272	-1.108297
C	5.673198	-0.794142	-0.908150
C	6.204307	0.473212	-0.542491
C	5.373991	1.570248	-0.374812
C	2.911940	2.355099	-0.433682
C	2.036355	0.229861	-1.029466
H	3.902417	-1.946788	-1.385332
H	6.355217	-1.643952	-1.032621
H	7.285465	0.578352	-0.392331
H	5.779725	2.549313	-0.089363
O	2.864550	3.552933	-0.149388
O	1.183242	-0.628186	-1.353363
N	1.764655	1.558147	-0.679898
O	0.520181	2.096459	-0.755152
C	0.038786	2.447852	0.525717
H	-0.144843	1.549423	1.147260
H	0.748733	3.124786	1.036935
C	-1.254535	3.192285	0.329726
F	-1.115937	4.235807	-0.478969

F	-2.215735	2.417226	-0.174211
F	-1.686651	3.644673	1.510562
Zn	-0.698541	-1.072915	-0.618431
Br	-1.853231	-0.182159	-2.597723
Br	-0.644851	-3.505642	-0.717242
O	0.369481	-0.658972	1.234259
O	-2.467937	-0.830637	0.597706
C	0.687963	-1.384923	2.192792
C	-3.066739	0.008904	1.285768
N	1.860498	-1.200786	2.831847
N	-4.286291	0.466897	0.937226
C	-0.238560	-2.455291	2.696125
H	0.185228	-3.463870	2.539532
H	-0.438732	-2.333719	3.776636
H	-1.182484	-2.390667	2.133525
C	-2.471801	0.526319	2.566374
H	-3.001850	0.102845	3.440806
H	-1.418349	0.212040	2.612087
H	-2.528790	1.625504	2.648251
C	2.747130	-0.113721	2.464639
H	3.066757	0.419529	3.379150
H	3.649350	-0.495049	1.945660
H	2.226714	0.588286	1.796145
C	2.314817	-2.004108	3.948026
H	2.164520	-1.475794	4.910183
H	1.800354	-2.974406	3.989665
H	3.395440	-2.201289	3.830485
C	-4.995736	1.475131	1.698588
H	-4.652198	2.498524	1.444378
H	-6.070565	1.405689	1.461590
H	-4.885577	1.321528	2.783595
C	-4.861980	0.124331	-0.347248
H	-5.880789	-0.284513	-0.212225
H	-4.925981	1.028405	-0.984998

H	-4.220942	-0.611374	-0.853910

TS-X-XI’ (doublet)

C	3.993747	-1.872112	-0.158222
C	3.582753	-0.754112	0.592693
C	4.532574	0.021274	1.266744
C	5.868021	-0.383336	1.201057
C	6.264935	-1.519706	0.479877
C	5.313391	-2.281949	-0.214349
C	2.775510	-2.432046	-0.778920
C	2.153970	-0.570584	0.402586
H	4.233303	0.907837	1.837927
H	6.629867	0.200013	1.732562
H	7.323007	-1.802505	0.451690
H	5.601116	-3.164768	-0.797931
O	2.641905	-3.463243	-1.393828
O	1.458209	0.356650	0.901709
N	1.721786	-1.511426	-0.504831
O	0.440001	-2.397511	0.034746
C	-0.417564	-2.499311	-1.042955
H	-0.767834	-1.507796	-1.405940
H	0.030985	-3.044207	-1.901790
C	-1.626614	-3.288364	-0.615241
F	-1.307338	-4.492614	-0.148360
F	-2.330693	-2.667009	0.334953
F	-2.450748	-3.470658	-1.656849
Zn	-0.462991	0.958759	0.629257
Br	-1.166841	0.111979	2.871867
Br	-0.335069	3.405531	0.878419
O	0.078772	1.022138	-1.509462
O	-2.422581	0.618389	-0.104009
C	0.184266	2.114248	-2.094301
C	-3.192505	-0.032137	-0.826050
N	1.398721	2.640695	-2.340359
N	-4.324147	-0.568507	-0.328563

C	-1.030889	2.857239	-2.574287
H	-0.969968	3.941336	-2.381831
H	-1.169668	2.709243	-3.663310
H	-1.908588	2.455882	-2.044582
C	-2.912021	-0.219015	-2.291700
H	-3.596428	0.406000	-2.897687
H	-1.877607	0.105472	-2.484896
H	-3.044335	-1.264682	-2.618795
C	2.551510	1.966916	-1.764106
H	3.472284	2.442196	-2.136072
H	2.524832	2.016021	-0.658667
H	2.553840	0.899480	-2.045929
C	1.573546	3.951472	-2.934072
H	2.598196	4.037695	-3.329293
H	0.881215	4.103912	-3.777857
H	1.415901	4.761873	-2.193527
C	-5.244990	-1.358992	-1.119089
H	-4.949806	-2.427895	-1.129022
H	-6.251463	-1.281830	-0.673443
H	-5.312919	-0.995408	-2.155784
C	-4.589918	-0.544671	1.096015
H	-5.517263	0.021420	1.307282
H	-4.724849	-1.580974	1.461162
H	-3.741528	-0.086974	1.629216

XI’ (doublet)

C	4.209708	-0.852300	0.720133
C	3.295402	-1.428521	-0.154028
C	3.698342	-2.062635	-1.316774
C	5.075689	-2.113766	-1.574733
C	5.996393	-1.539312	-0.692767
C	5.571011	-0.890556	0.475625
C	3.391781	-0.240919	1.825916
C	1.942891	-1.159344	0.432595
H	2.968914	-2.501136	-2.008179

H	5.440366	-2.608530	-2.482406
H	7.066456	-1.594732	-0.924518
H	6.286250	-0.429013	1.166939
O	3.842423	0.424064	2.748015
O	0.888037	-1.492765	-0.125856
N	2.064306	-0.517113	1.610645
O	0.619268	1.359638	1.255932
C	1.426072	2.365679	1.660828
H	0.833701	3.168048	2.164696
H	2.157583	2.008689	2.423888
C	2.208626	3.013580	0.538310
F	2.958876	2.137289	-0.126300
F	1.407051	3.608136	-0.347946
F	3.025298	3.955288	1.025838
Zn	-0.822133	-0.334761	-0.581932
Br	0.450180	0.795906	-2.354625
Br	-2.104742	-2.265810	-1.495485
O	-1.587754	-0.441063	1.376560
O	-2.502638	1.008955	-0.836905
C	-2.428068	-1.288079	1.731031
C	-2.930212	1.930424	-0.131017
N	-2.066353	-2.493808	2.199772
N	-4.258766	2.171674	-0.035734
C	-3.894552	-0.985348	1.637770
H	-4.481218	-1.371343	2.488388
H	-4.028029	0.106733	1.564574
H	-4.291334	-1.442411	0.709733
C	-1.996191	2.797729	0.658433
H	-2.295641	3.858658	0.678941
H	-1.937249	2.423550	1.698915
H	-0.991215	2.706744	0.216527
C	-0.664789	-2.844296	2.329294
H	-0.492250	-3.307730	3.318052
H	-0.372392	-3.566428	1.542717

H	-0.041195	-1.943230	2.222010
C	-3.037756	-3.536636	2.474690
H	-2.501979	-4.491368	2.596566
H	-3.608942	-3.345305	3.402834
H	-3.746764	-3.656689	1.636042
C	-4.812437	3.254286	0.754714
H	-5.867502	3.022969	0.976299
H	-4.288618	3.360375	1.718506
H	-4.779391	4.223708	0.219392
C	-5.199505	1.405139	-0.827937
H	-5.975335	0.961667	-0.175750
H	-5.701240	2.053136	-1.572432
H	-4.663757	0.600050	-1.351501

XI (doublet)

C	0.982677	0.677066	0.000062
H	1.060652	1.368028	-0.876036
H	1.060676	1.367928	0.876244
O	2.005413	-0.187161	-0.000023
C	-0.379214	0.010370	0.000006
F	-0.536493	-0.759434	-1.073846
F	-0.536292	-0.760034	1.073469
F	-1.347815	0.923549	0.000329

XI-a/VIII (doublet)

C	2.144040	-0.899177	0.634965
H	0.505668	-0.291358	1.518501
H	3.066921	-1.477720	0.748852
O	1.330843	-0.849647	1.687195
C	2.088876	0.145638	-0.407209
F	0.949648	0.118660	-1.130328
F	2.151438	1.393989	0.093255
F	3.099166	0.004103	-1.263142
O	-0.771639	0.620742	1.254506
C	-1.730366	0.589315	0.472165

C	-2.504063	1.850887	0.203186
H	-2.114305	2.635597	0.869065
H	-2.373276	2.184794	-0.842356
H	-3.585908	1.727745	0.385822
N	-2.109863	-0.550551	-0.143418
C	-3.144295	-0.643144	-1.152480
H	-2.720853	-1.043219	-2.093164
H	-3.950039	-1.325207	-0.821073
H	-3.587996	0.337559	-1.371046
C	-1.380797	-1.776527	0.118510
H	-0.475474	-1.854469	-0.516575
H	-1.073347	-1.825985	1.174950
H	-2.038628	-2.635362	-0.095541

XXVI

C	-3.107917	0.208323	-0.842350
C	-2.214507	-1.788139	-1.584705
C	-3.466343	-2.382181	-1.677961
C	-4.590049	-1.619161	-1.331472
C	-4.394681	-0.293045	-0.912445
C	-2.767927	1.567471	-0.388228
C	-1.049748	3.045779	0.064052
C	-1.933383	4.055173	0.418309
C	-3.306937	3.791190	0.364762
C	-3.715894	2.514488	-0.051024
H	-1.301760	-2.337593	-1.848023
H	-3.545117	-3.417165	-2.019593
H	-5.270773	0.302432	-0.640628
H	0.029272	3.227506	0.099391
H	-1.533660	5.023833	0.727870
H	-4.787063	2.300002	-0.101705
N	-2.031761	-0.535226	-1.173887
N	-1.433277	1.826248	-0.328717
O	-5.843820	-2.053174	-1.367557
O	-4.264013	4.655033	0.673657

C	-6.107523	-3.380276	-1.776402
H	-7.197995	-3.511524	-1.723474
H	-5.769210	-3.550161	-2.815492
H	-5.618956	-4.107909	-1.101949
C	-3.913185	5.957681	1.097820
H	-4.857455	6.486345	1.292351
H	-3.314703	5.923980	2.026993
H	-3.349880	6.492455	0.310797
Ni	-0.311060	0.353500	-0.817249
O	0.665524	-1.189132	-1.341233
C	1.589959	-1.630103	-0.585769
C	2.953624	-1.943744	-0.837458
C	3.556441	-2.365322	0.386814
C	3.721990	-1.868720	-2.014469
C	2.568645	-2.322482	1.451865
C	4.909031	-2.714132	0.431776
C	5.061048	-2.213137	-1.950807
H	3.263454	-1.536053	-2.953853
C	5.656372	-2.637204	-0.736034
H	5.363161	-3.034715	1.377628
H	5.678001	-2.157486	-2.855649
H	6.719687	-2.903126	-0.723220
O	2.618153	-2.573268	2.649791
N	1.403398	-1.909627	0.772017
C	1.327012	1.220689	-0.483038
C	1.876165	1.323602	0.807321
C	2.114714	1.656749	-1.559511
C	3.159472	1.820357	1.009120
H	1.292236	0.991601	1.678847
C	3.398097	2.163572	-1.365377
H	1.728194	1.584891	-2.586544
C	3.944393	2.242665	-0.075245
H	3.587859	1.885655	2.016531
H	3.980630	2.488837	-2.236077

C	5.316096	2.748369	0.189877
O	5.748902	2.835817	1.326574
C	6.168688	3.145148	-0.986906
H	6.308107	2.295365	-1.680820
H	5.697516	3.960589	-1.566247
H	7.152825	3.482479	-0.623738
O	0.275127	-1.547005	1.427332
C	-0.625393	-2.626744	1.536509
H	-0.965693	-2.974717	0.540857
H	-0.171896	-3.471299	2.089456
C	-1.818108	-2.127038	2.309960
F	-1.484555	-1.748225	3.538686
F	-2.394731	-1.083261	1.713600
F	-2.724659	-3.094861	2.404485

XXVII

C	2.872135	-0.534935	-0.548169
C	2.436938	1.157165	-2.062071
C	3.777478	1.281478	-2.404929
C	4.701042	0.436989	-1.775633
C	4.227605	-0.482445	-0.825518
C	2.283512	-1.450973	0.457562
C	0.332288	-2.117524	1.506619
C	1.003727	-3.017312	2.323625
C	2.392652	-3.131839	2.177702
C	3.034962	-2.325083	1.224431
H	1.677756	1.813053	-2.505812
H	4.076067	2.027371	-3.145655
H	4.952472	-1.132249	-0.327128
H	-0.753985	-1.990225	1.586943
H	0.440296	-3.607004	3.050808
H	4.120306	-2.409294	1.120131
N	1.989139	0.274193	-1.170876
N	0.943878	-1.361687	0.597669

O	6.010027	0.437443	-2.000984
O	3.165860	-3.951304	2.880580
C	6.552399	1.343164	-2.940242
H	7.636898	1.161738	-2.957606
H	6.136675	1.165949	-3.949628
H	6.362527	2.390211	-2.638820
C	2.582216	-4.789366	3.857333
H	3.403878	-5.371095	4.299793
H	2.088913	-4.194371	4.648378
H	1.849658	-5.480948	3.400945
Ni	0.005637	-0.053844	-0.655303
O	-0.109380	3.006795	-1.411715
C	-0.441522	2.793336	-0.254673
C	-1.031941	3.792704	0.693017
C	-1.317559	3.105903	1.868754
C	-1.302548	5.143880	0.556910
C	-0.901014	1.679598	1.653441
C	-1.884175	3.741530	2.960474
C	-1.874737	5.799652	1.655118
H	-1.076956	5.676743	-0.374269
C	-2.160759	5.109801	2.837487
H	-2.106732	3.192474	3.882985
H	-2.103880	6.869507	1.589745
H	-2.609252	5.650911	3.678594
O	-1.030073	0.770545	2.454315
N	-0.361288	1.588657	0.392677
C	-1.816514	-0.769100	-0.929271
C	-2.974570	0.030084	-0.855860
C	-2.022513	-2.131426	-1.220550
C	-4.247651	-0.487087	-1.070784
H	-2.882769	1.101661	-0.617998
C	-3.291215	-2.667308	-1.437294
H	-1.159917	-2.813455	-1.282003
C	-4.426873	-1.847126	-1.366538

H	-5.137034	0.152109	-1.011488
H	-3.390999	-3.736575	-1.662116
C	-5.805934	-2.356493	-1.587581
O	-6.771790	-1.615587	-1.517328
C	-5.983899	-3.818481	-1.903342
H	-5.437488	-4.097566	-2.823248
H	-5.588428	-4.453685	-1.089224
H	-7.056282	-4.031611	-2.040208

XXVIII

C	-2.887286	-0.919779	-0.655773
C	-1.531785	-2.741409	-0.237020
C	-2.541148	-3.644372	-0.542007
C	-3.789370	-3.136282	-0.922326
C	-3.952607	-1.743118	-0.976986
C	-2.971367	0.557249	-0.677789
C	-1.820451	2.533620	-0.372923
C	-2.936260	3.306017	-0.665445
C	-4.131560	2.646371	-0.978249
C	-4.135989	1.242528	-0.979567
H	-0.546122	-3.097775	0.080327
H	-2.339400	-4.716258	-0.476989
H	-4.926512	-1.347295	-1.277240
H	-0.868535	3.018749	-0.119876
H	-2.855635	4.395494	-0.642998
H	-5.071017	0.728798	-1.219325
N	-1.688513	-1.420859	-0.289922
N	-1.822529	1.201486	-0.377800
O	-4.847158	-3.872986	-1.240295
O	-5.276969	3.246165	-1.277297
C	-4.747153	-5.282212	-1.202093
H	-5.732240	-5.672570	-1.496236
H	-3.982870	-5.645683	-1.914002
H	-4.503765	-5.635779	-0.182854
C	-5.336798	4.658408	-1.295899

H	-6.370003	4.922711	-1.563969
H	-5.098266	5.079045	-0.301412
H	-4.645247	5.074944	-2.051753
Ni	-0.228285	-0.075115	0.112217
O	1.175393	-1.339208	-0.804065
C	2.205466	-1.815934	-0.296494
C	3.469667	-2.057501	-1.050979
C	4.366107	-2.589315	-0.129925
C	3.825752	-1.834619	-2.370452
C	3.634386	-2.674405	1.179903
C	5.659278	-2.926125	-0.488150
C	5.133417	-2.171712	-2.746163
H	3.114755	-1.412795	-3.090592
C	6.034260	-2.708851	-1.821477
H	6.360390	-3.343511	0.243936
H	5.458093	-2.013065	-3.780799
H	7.049674	-2.961347	-2.147677
O	4.088550	-3.102682	2.221602
N	2.354057	-2.182976	0.990176
C	1.050331	1.348048	0.296221
C	1.309525	2.236790	1.346222
C	1.661753	1.600195	-0.941858
C	2.141875	3.337699	1.166984
H	0.849797	2.094606	2.332537
C	2.497034	2.701046	-1.126681
H	1.489062	0.927599	-1.793606
C	2.748602	3.587639	-0.071052
H	2.335805	4.033118	1.991904
H	2.954015	2.860357	-2.110707
C	3.630311	4.780170	-0.207681
O	3.816131	5.532559	0.732020
C	4.286629	5.031059	-1.538494
H	4.932276	4.182047	-1.830139
H	3.533166	5.152756	-2.338572

H	4.898036	5.945391	-1.474635
C	-0.041049	-0.508750	2.043794
H	0.829582	0.043349	2.446400
C	-1.272543	-0.047094	2.783373
F	-1.447108	1.273423	2.681498
F	-2.396421	-0.631616	2.367544
F	-1.162150	-0.322852	4.089631
O	0.078882	-1.847293	2.134800
H	1.044146	-2.088405	1.810772

TS-VIII-P

C	-2.935684	0.361646	0.282515
C	-2.402873	1.996690	-1.254543
C	-3.685653	2.532329	-1.227627
C	-4.633256	1.930752	-0.390831
C	-4.238831	0.825803	0.378141
C	-2.426141	-0.786669	1.068551
C	-0.603993	-2.108508	1.566143
C	-1.296922	-2.816144	2.540087
C	-2.636823	-2.483297	2.768455
C	-3.196687	-1.438985	2.018943
H	-1.621507	2.440657	-1.882429
H	-3.920385	3.398939	-1.850755
H	-4.981098	0.361373	1.033234
H	0.454802	-2.321143	1.383611
H	-0.783080	-3.601232	3.100288
H	-4.234717	-1.159264	2.218521
N	-2.034799	0.944283	-0.530205
N	-1.142642	-1.138318	0.830464
O	-5.897300	2.327657	-0.258912
O	-3.429592	-3.073921	3.658249
C	-6.354668	3.436496	-1.002934
H	-7.411789	3.578605	-0.734229
H	-5.787998	4.351265	-0.745996
H	-6.278001	3.247323	-2.090223

C	-2.916391	-4.126096	4.448116
H	-3.739474	-4.460210	5.096625
H	-2.582948	-4.971554	3.817499
H	-2.075145	-3.779132	5.077015
Ni	-0.180867	0.027405	-0.580992
O	0.569736	3.133731	-0.731907
C	1.107659	2.553588	0.200656
C	2.219017	3.106640	1.042086
C	2.596467	2.093112	1.915114
C	2.850395	4.339425	1.044308
C	1.708139	0.918135	1.623866
C	3.620633	2.267036	2.830792
C	3.887749	4.530053	1.966913
H	2.548692	5.132798	0.350192
C	4.267018	3.510185	2.845762
H	3.912316	1.461080	3.514565
H	4.413315	5.491350	2.002517
H	5.083751	3.689611	3.554547
O	1.772771	-0.166063	2.178799
N	0.834838	1.279733	0.626111
C	1.373906	-0.894996	-1.272880
C	1.756674	-2.182781	-0.848639
C	2.394143	0.001965	-1.662002
C	3.097267	-2.508124	-0.704750
H	0.994029	-2.935992	-0.623339
C	3.729980	-0.328496	-1.514056
H	2.123983	0.993902	-2.050727
C	4.111202	-1.590242	-1.019446
H	3.390413	-3.500307	-0.342637
H	4.488345	0.413807	-1.789785
C	5.524385	-1.994953	-0.837008
O	5.816253	-3.105302	-0.422245
C	6.599083	-0.995548	-1.177238
H	6.549025	-0.702251	-2.242354

H	6.488303	-0.069970	-0.582320
H	7.585847	-1.441254	-0.972871
C	-0.061288	-0.834718	-2.530196
H	0.647083	-1.387945	-3.170449
C	-1.288771	-1.729246	-2.616834
F	-1.093106	-2.858385	-1.934856
F	-2.421648	-1.185111	-2.192797
F	-1.496678	-2.072020	-3.895818
O	-0.328025	0.423123	-3.068821
H	0.276484	0.617831	-3.806179

11. References

[1] a) Y. Zhao, D. G. Truhlar, The M06 suite of density functionals for main group thermochemistry, thermochemical kinetics, noncovalent interactions, excited states, and transition elements: two new functionals and systematic testing of four M06-class functionals and 12 other functionals. *Theor. Chem. Acc.* **2008**, *120*, 215–241. b) Y. Zhao, D. G. Truhlar. Density Functionals with Broad Applicability in Chemistry. *Acc. Chem. Res.* **2008**, *41*, 157–167.

[2] F. Weigend, Accurate Coulomb-fitting basis sets for H to Rn. *Phys. Chem. Chem. Phys.* **2006**, *8*, 1057–1065.

[3] F. Weigend, R. Ahlrichs, Balanced basis sets of split valence, triple zeta valence and quadruple zeta valence quality for H to Rn: Design and assessment of accuracy. *Phys. Chem. Chem. Phys.* **2005**, *7*, 3297–3305.

[4] J. Tomasi, B. Mennucci, R. Cammi, Quantum Mechanical Continuum Solvation Models. *Chem. Rev.* **2005**, *105*, 2999–3093.

[5] Gaussian 09, Revision A.02, M. J. Frisch, G. W. Trucks, H. B. Schlegel, G. E. Scuseria, M. A. Robb, J. R. Cheeseman, G. Scalmani, V. Barone, G. A. Petersson, H. Nakatsuji, X. Li, M. Caricato, A. Marenich, J. Bloino, B. G. Janesko, R. Gomperts, B. Mennucci, H. P. Hratchian, J. V. Ortiz, A. F. Izmaylov, J. L. Sonnenberg, D. Williams-Young, F. Ding, F. Lipparini, F. Egidi, J. Goings, B. Peng, A. Petrone, T. Henderson, D. Ranasinghe, V. G. Zakrzewski, J. Gao, N. Rega, G. Zheng, W. Liang, M. Hada, M. Ehara, K. Toyota, R. Fukuda, J. Hasegawa, M. Ishida, T. Nakajima, Y. Honda, O. Kitao, H. Nakai, T. Vreven, K. Throssell, J. A. Montgomery, J. E. Peralta, F. Ogliaro, M. Bearpark, J. J. Heyd, E. Brothers, K. N. Kudin, V. N. Staroverov, T. Keith, R. Kobayashi, J. Normand, K. Raghavachari, A. Rendell, J. C. Burant, S. S. Iyengar, J. Tomasi, M. Cossi, J. M. Millam, M. Klene, C. Adamo, R. Cammi, J. W. Ochterski, R. L. Martin, K. Morokuma, O. Farkas, J. B. Foresman, D. J. Fox, Gaussian, Inc., Wallingford CT.

[6] H. B. Schlegel, J. J. W. McDouall, "Do You Have SCF Stability and Convergence Problems?" in *Computational Advances in Organic Chemistry: Molecular Structure and Reactivity*, Dordrecht: Springer Netherlands, 1991, pp. 167–185.

[7] R. Bauernschmitt, R. Ahlrichs, Stability analysis for solutions of the closed shell Kohn–Sham equation. *J. Chem. Phys.* **1996**, *104*, 9047–9052.

[8] R. Seeger, J. A. Pople, Self-consistent molecular orbital methods. XVIII. Constraints and stability in Hartree–Fock theory. *J. Chem. Phys.* **1977**, 66, 3045–3050.

[9] S. Maeda, Y. Harabuchi, Y. Ono, T. Taketsugu, K. Morokuma, Intrinsic reaction coordinate: Calculation, bifurcation, and automated search. *Inter. J. Quantum Chem.* **2015**, *115*, 258–269.

[10] K. Fukui, The path of chemical reactions - the IRC approach. *Acc. Chem. Res.* **1981**, *14*, 363–368.

[11] a) D. Sheppard, R. Terrell, G. Henkelman, Optimization methods for finding minimum energy paths. *J. Chem. Phys.* **2008**, *128*, 134106. b) G. Henkelman, G. Jóhannesson, H. Jónsson, "Methods for Finding Saddle Points and Minimum Energy Paths" in *Progress on Theoretical Chemistry and Physics*, Ed. S. D. Schwartz, 269–300 (Kluwer Academic Publishers, 2000). c) G. Henkelman, B. P. Uberuaga, H. Jónsson, A climbing image nudged elastic band method for finding saddle points and minimum energy paths. *J. Chem. Phys.* **2000**, *113*, 9901. d) G. Henkelman, H. Jónsson, Improved tangent estimate in the nudged elastic band method for finding minimum energy paths and saddle points. *J. Chem. Phys.*, **2000**, *113*, 9978. e) H. Jónsson, G. Mills, K. W. Jacobsen, "Nudged Elastic Band Method for Finding Minimum Energy Paths of Transitions" in *Classical and Quantum Dynamics in Condensed Phase Simulations*, Ed. B. J. Berne, G. Ciccotti, D. F. Coker, 385 (World Scientific, 1998).

[12] G. Schaftenaar, E. Vlieg, G. Vriend, Molden 2.0: quantum chemistry meets proteins. *J. Comput. Aided. Mol. Des.* **2017**, *31*, 789–800.

[13] CYLview20; C. Y. Legault, Université de Sherbrooke, 2020 (http://www.cylview.org).

[14] C. Shu, A. Noble, V. K. Aggarwal, Metal-free photoinduced C(sp^3)–H borylation of alkanes. *Nature* **2020**, *586*, 714–719.

[15] J. C. Son, B. J. Kim, J. H. Kim, I. Y. Lee, C. S. Yun, S. H. Lee, C. K. Lee, Novel Antiviral Pyrrolopyridine Derivatives and Method for Preparing the Same. US 2014/249162 A1, September 4, 2014.

[16] K. Hayashi, K .Kunitani, S. Uehara, T. Morita, Novel Arylamidine Derivative, Salt Thereof, and Antifungal Containing These. US 2008/319016 A1, December 25, 2008.

[17] H. Yuan, K. Bi, W. Chang, R. Yue, B. Li, J. Ye, Q. Sun, H. Jin, L. Shan, W. Zhang, Total synthesis of Daphnodorin A. *Tetrahedron* **2014**, *70*, 9084–9092.

[18] R. M. Denton, J. T. Scragg, J. A Saska, concise synthesis of 4′-O-methyl honokiol. *Tetrahedron Lett.* **2011**, *52*, 2554–2556.

[19] C. S. Yeung, V. M. Dong, Beyond Aresta's Complex: Ni- and Pd-Catalyzed Organozinc Coupling with CO_2. *J. Am. Chem. Soc.* **2008**, *130*, 7826–7827.

[20] Y. H. Lee, B. Morandi, Palladium-Catalyzed Intermolecular Aryliodination of Internal Alkynes. *Angew. Chem. Int. Ed.* **2019**, *58*, 6444–6448.

[21] P. Boehm, S. Roediger, A. Bismuto, B. Morandi, Palladium-Catalyzed Chlorocarbonylation of Aryl (Pseudo)Halides Through In Situ Generation of Carbon Monoxide. *Angew. Chem. Int. Ed.* **2020**, *59*, 17887–17896.

[22] S. M. Goldup, D. A. Leigh, P. J. Lusby, R. T. McBurney, A. M. Z. Slawin, Active Template Synthesis of Rotaxanes and Molecular Shuttles with Switchable Dynamics by Four-Component Pd^{II}-Promoted Michael Additions. *Angew. Chem. Int. Ed.* **2008**, *47*, 3381–3384.

[23] A. P. Melissaris, H. M. Litt, A Simple and Economical Synthetic Route to p-Ethynylaniline and Ethynyl-Terminated Substrates. *J. Org. Chem.* **1994**, *59*, 5818–5821.

[24] T. Suzuki, Y. Ota, M. Ri, M. Bando, A. Gotoh, Y. Itoh, H. Tsumoto, P. R. Tatum, T. Mizukami, H. Nakagawa, S. Iida, R. Ueda, K. Shirahige, N. Miyata, Rapid Discovery of Highly Potent and Selective Inhibitors of Histone Deacetylase 8 Using Click Chemistry to Generate Candidate Libraries. *J. Med. Chem.* **2012**, *55*, 9562–9575.

[25] C. Montoro-García, M. J. Mayoral, R. Chamorro, D. González-Rodríguez, How Large Can We Build a Cyclic Assembly? Impact of Ring Size on Chelate Cooperativity in Noncovalent Macrocyclizations. *Angew. Chem. Int. Ed.* **2017**, *56*, 15649–15653.

[26] I. Medina-Mercado, S. Porcel, Biaryl Coupling of Aryldiazonium Salts and Arylboronic Acids Catalysed by Gold. *Synthesis* **2022**, *54*, A–L.

[27] H.-L. Lia, Y. Kuninobu, Palladium-Catalyzed Secondary C(sp^3) –H Arylation of 2-Alkylpyridines. *Adv. Synth. Catal.* **2020**, *362*, 2637–2641.

[28] H. Bölcskeia, A. Német-Hanzelika, I. Greinera, Z. Dubrovaya, V. Hádaa, G. Keglevich, The Synthesis of (Iodobenzyl)oxybenzaldehydes, Useful Intermediates for Biologically Active Targets. *Lett. Drug Des. Discov.* **2017**, *14*, 233–239.

[29] Q.-W. Zhao, Z.-F. Yang, X.-P. Fu, X. Zhang, Access to α,α-Difluoro-γ-amino Acids by Nickel-Catalyzed Reductive Aryldifluoroacetylation of N-Vinylacetamide. *Synlett* **2021**, *32*, 1565–1569.

[30] Y.-Q. Han, X. Yang, K.-X. Kong, Y.-T. Deng, L.-S. Wu, Y. Ding, B.-F. Shi, Synthesis of Acyclic Aliphatic Amides with Contiguous Stereogenic Centers via Palladium-Catalyzed Enantio-, Chemo- and Diastereoselective Methylene C(sp^3)−H arylation. *Angew. Chem. Int. Ed.* **2020**, *59*, 20455–20458.

[31] V. G. Landge, A. J. Grant, Y. Fu, A. M. Rabon, J. L. Payton, M. C. Young, Palladium-Catalyzed γ,γ′-Diarylation of Free Alkenyl Amines. *J. Am. Chem. Soc.* **2021**, *143*, 10352–10360.

[32] W.-S. Han, J.-K. Han, H.-Y. Kim, M. J. Choi, Y.-S. Kang, C. Pac, S. O. Kang, Electronic Optimization of Heteroleptic Ru(II) Bipyridine Complexes by Remote Substituents: Synthesis, Characterization, and Application to Dye-Sensitized Solar Cells. *Inorg. Chem.* **2011**, *50*, 3271–3280.

[33] C.-M. Hsu, S.-C. Lee, H.-E. Tsai, Y.-T. Tsao, C.-L. Chan, S. Miñoza, Z.-N. Tsai, L.-Y. Li, H.-H. Liao, Desulfurative Ni-Catalyzed Reductive Cross-Coupling of Benzyl Mercaptans/Mercaptoacetates with Aryl Halides. *J. Org. Chem.* **2022**, *87*, 3799–3803.

[34] Q. Lin, T. Diao, Mechanism of Ni-Catalyzed Reductive 1,2-Dicarbofunctionalization of Alkenes. *J. Am. Chem. Soc.* **2019**, *141*, 17937−17948.

[35] G. K. S. Prakash, Z. Zhang, F. Wang, S. Munoz, G. A. Olah, Nucleophilic Trifluoromethylation of Carbonyl Compounds: Trifluoroacetaldehyde Hydrate as a Trifluoromethyl Source. *J. Org. Chem.* **2013**, *78*, 3300−3305.

[36] R. Kani, T. Inuzuka, Y. Kubota, K. Funabiki, One-Pot Successive Turbo Grignard Reactions for the Facile Synthesis of α-Aryl-α-Trifluoromethyl Alcohols. *Eur. J. Org. Chem.* **2020**, 4487–4493.

[37] B. Zhang, X. Zhang, J. Hao, C. Yang, Palladium-Catalyzed Direct Approach to α-Trifluoromethyl Alcohols by Selective Hydroxylfluorination of gem- Difluoroalkenes. *Eur. J. Org. Chem.* **2018**, 5007–5015.

[38] K. Terashima, T. Kawasaki-Takasuka, I. Minami, T. Yamazaki, Synthesis and synthetic applications of (4-hydroxyphenyl) perfluoroalkylmethanols. *Tetrahedron* **2022**, *104*, 132574.

[39] L. Rushaa, S. C. Miller, Design and application of esterase-labile sulfonate protecting groups. *Chem. Commun.* **2011**, *47*, 2038–2040.

[40] K. Aikawa, W. Toya, Y. Nakamura, K. Mikami, Development of (Trifluoromethyl)zinc Reagent as Trifluoromethyl Anion and Difluorocarbene Sources. *Org. Lett.* **2015**, *17*, 4996−4999.

[41] G. E. Aspnes, R. L. Dow, Munchhof, M. J, 4-Amino-7,8-Dihydropyrido[4,3-d]pyrimidin-5(6H)-one Derivatives. US 2010/197591 A1, August 5, 2010.

[42] H. Jia, A. P. Häring, F. Berger, L. Zhang, T. Ritter, Trifluoromethyl Thianthrenium Triflate: A Readily Available Trifluoromethylating Reagent with Formal $CF3^+$, $CF3^•$, and $CF3^−$ Reactivity. *J. Am. Chem. Soc.* **2021**, *143*, 7623−7628.

[43] J. J. Song, Z. Tan, J. T. Reeves, F. Gallou, N. K. Yee, C. H. Senanayake, N-Heterocyclic Carbene Catalyzed Trifluoromethylation of Carbonyl Compounds. *Org. Lett.* **2005**, *7*, 2193-2197.

[44] M. Brambilla, M. Tredwell, Palladium-Catalyzed Suzuki–Miyaura Cross-Coupling of Secondary α-(Trifluoromethyl)benzyl Tosylates. *Angew. Chem. Int. Ed.* **2017**, *56*, 11981–11985.

[45] T. Barton, X. Gao, J. Hunter, P. R. Leplae Jr., W. C. Lo, J. Boruwa, R. Tangirala, G. B. Watson, J. Herbert, Molecules Having Pesticida Utility, and Intermediates, Compositions, and Processes, Related thereto. US 2017/208804 A1, July 27, 2017.

[46] W. J. Hoekstra, C. M. Yates, Metalloenzyme Inhibitor Compounds. WO 2013/090210 A1, June 20, 2013.

[47] A. J. Roche, Complete assignment of ^{19}F, ^{1}H and ^{13}C NMR spectra of monomers and precursors towards bridge trifluoromethylated poly(p-phenylenevinylene). *Magn. Reson. Chem.* **2004**, *42*, 944–949.

[48] M. B. Johansen, O. R. Gedde, T. S. Mayer, T. Skrydstrup, Access to Aryl and Heteroaryl Trifluoromethyl Ketones from Aryl Bromides and Fluorosulfates with Stoichiometric CO. *Org. Lett.* **2020**, *22*, 4068−4072.
